# Supplementary material for: Structure of the germline genome of Tetrahymena thermophila and relationship to the massively rearranged somatic genome
Source: eLife. 2016 Nov 28;5:e19090. doi: 10.7554/eLife.19090 (PMC5182062; doi:10.7554/eLife.19090)
Supplement: Table 3—source data 1. — DOI: http://dx.doi.org/10.7554/eLife.19090.011 [file elife-19090-table3-data1.docx]

**Source data for Table 3, Supplementary File 2E**

>Multi-Cbs_1L-1_11C, 1L-2_11C, 1L-3_cons, 1L-4_11C, 1L-5_13A; 989 bp

GATAATAAATTTACTAATAAACAAACATGTAAGAAAATATAAAATCACTTTTTACAAATAACTTAAAAATTCTTGTATAATTTTAAATTTATTTATGCTTATTAAAATTTATATTTATTTTTCAGTCTTAAAAATAATATTTTTGAATCTTTATGTGAAATGTTTTTAGTTTTTTATAATTTTTTGTTTTTATATTGAAATAAACCAACCTCATTAGTAATTTATTTAAGACTGAATTATTATAATTTTAAACTATATTTTTAAATCTTTATTTGAAATTTTTTTAGTTTTTTATAATTTTATGTTTTTACATTAAAATAAACCAACAACCTCATTAGTAATTTATTTAAGACTGAATTATTATAATTTTAAACTATATTTTTTAATCTTTATGTGAAAATTTTTAAGTTTTTTATAATTTTATGTTTTTACATTAAAATAAACCAACCCCTTTAGTAATTTATTTAAGAATGAATATTCATTTTTTTAAATTATATTTTTTAATCTTTATTTGAAATTTTTTTAGTTTTTTATAATTTTATGTTTTTACATTAAAATAAACCAACCTCTTTAGTAATTTATTTAAGACAGACTGAATATTTATTTTTTTAAATTATATTTTTGAATCTTTATTTATAATTTTTTTAGTATTTTATAATTTTATGTTTTTACATTAAAATAAACCAACCCCTTTAGTAATTTATATAAGACTGAATATTTATAATTTTTAATTTTGTTTTATTTGATTGATAATTTTTTGTTTTTAAATTGAAATAAACCAACCCCTTTGATAACTTATTTAAGACTGAATATCTATAATTTTTAATTTTATTTTTTTTGATGATTTTTTATTATTATTTTAAATAAAAGTTGTTAAAATTATGAAATAAAATTTAAATTTAATTGTTTGAAATTTTTAATTAACTTTAAAAATCAAAATATTTCCTGTTAACAATTTTTCAATTATATTATAGAAAATCATTTAAAAA

>Cbs_1L-6_cons

AGTAATTAGATTGAAAGCATTTTTTTGTGAAAATAAAAATATTTTTTATTAAATAATTCTACTAGCTTATGAAAATCTAAAATCAGTTAAAAATAATATTTTATTTTAAAAAATATAATAAATGCAAATTTTTATAATTAATTTTTCAAAATTATGATTGATTGATTGAATGATATTATTTATGCTAAAGTTTTATTTATTAAACCAACCTCTTTTAATTTTATATATTTCTTTAAACAAAATTTATTTATTTAAATATTATTTTCATTTAAGTAGTATAAAAATAAAAGAAATTCTTGATATCTTTTGGAAAAAATAAATTTCAAAATACAATCTAAAAGTTCATTTTATTTTAGAATAAATTAAAAATTAGTATAAAATCAACCAAATAATTATAAATAATTTAAAATTTTTG

>Cbs_1L-7_1A

TTTCTTCATTATAAAGATTACTCATTTTATAATAACCTATATTTAATCACTTTAATTCTTAGATTTTATTAAATTTTATATCTTTTCTAGAATTCTAATAAGATTTCAAAACATCATAAAATGTAAAATTAATTAGTTTTTGTAAAATACCAAATATTTATTATTATACACTTAATTTTTTTAAACAAATTTAATAAATAAAAACCAACCTCTTTAAAATTAAAAATATTTTGTTGAAATTTAAATTCTTCTTTTATATTTAATTAGGTTATTGAATTATAAATATTATTTTAATAGCATAATTAATATCATACTCTTTTATATGTAAATAAAGTAATTAAAATCATTATTTATTTAAATAATCCAAATAAAATTTATTATCATAATCAAGTAAATCTTAATTTTTTATATTATT

>Cbs_1L-8_15A

TTTAGATATTATTTACAATTCAAATTTTGTGTGTGTCTCTCGTTAATTAATTAATTTTTAGCAAAATTAAATAGAGCTTATAAATTTTAATTTGCAAAAAATTGAAATTTAATGCAAAGTGTAAGATAAATATAATTATATTTTTAATAATCAAAAATTAATCCTATATTTATTTTAACAATTAAAATTAGTTTTATAAATAAACCAACCTCTTAAATAATTTTAAGCGTATTACTGACACAACAAATTTATTTTAATTTATATTTAAACTGTTATTTATTTTAAATTTAAATACCGACTCAAATAAATATTATTTTTTTGTATTTCATTTTAAAAATAAAAAGAAATATTTATTTTTATAATAATTTCTTTTAAATTAAGTATCATAAAATACAATATTTAAAATAATCAAATA

>Cbs_1L-9_cons

TAAAATAAGTTTAAATTATTATTTTAATTTCAAAAAATTAGCTTTTATATTAAAAGTTAAGCATAATTTTAAATTTTACATCCATAAATGAAATAATTTTTTTAAATTAAATTTTCCATCAAATAAAATAACACTTTTAGTAAGACTTAATTTAGATTGAGAATACTAAGTAGATTTTATTTTATTAAAATTCGTTTTTATAAACCAACCTCTTTATTATATTTATTTATTCATTCATATTTTTCTTTTCAAAAAATTAATTTTGAACAAAAGTTTAATAATTTATTCTAAAAAAAATAAAGTAATAATTTATATTTAATATTAATTTTTTTAATTAAATTTTGTTATAAAATGAAATAAATTTTAGTTATTTCAAAAAATAAAAACACTTTAATAATAATTTTAACAAAATATT

>Cbs_1L-10_1A

TCTTTATAAATTAATAAAATTTTTAATTTTAAAAATAATTTATTTTTTTCTATGCTAAATATTTTAATCTAGAAAGTCAGTAATGTGTCTACAATTATTTATATTTTATTAGAAAGGACAATAAAAAGAATATTTGAAATATTTATTTTGTATTTTAAATTTAACAGAATTAATCTTAAAAGATGTAAAAATAATTAAAAAAAACCAACCTCTTTCTTAATTTTAAATTTAAAAATTTTATGATACTAATTTAAAGAAAAGTTAGCAAAGTAAATATTTATTTTTCTGATTATAATTCAAAATAAAAATGCAAATTTAAAAAAAACAAAAATACTTCTACTTTTATTTAAAATATACTCTAGAATATTCTGTTCTATGAATAAGTTAAAAATCGTTTGATAATTTTTAAAATAAA

>Cbs_1L-11_cons

AATTAATAATAGTATAGAAGTATAATCTATTAAGTGTCATAGCTCCCAAGATTCTATAATTTTTTATTTAATTAATTTAAGGCAATGATAATGAAATTTTTATTATTTTGCTTTATTTATTTTTAAATCTCTTAATACCAATTTATCTTTAAATCTTAATCAAATTTTAGCTTATTTGAAATTTGCAAAAATTATGAAATTAAACCAACCTCTTTAATAATTAAATATTTATAAGCTTACATTTTAAAATAAAATATGTGTATTCTAATAATTTAAAATATCACAAAGTAAAAAATAGATAATAAAATTTTAACTAAACCAAAATAGAATACAAAAGTTATAACAAAATAATTAAGTAAATAAAAAAATCAGTCAAACATATAAAAATTGAAATAGATTTATTTTTTAACAAATA

>Cbs_1L-12_14C

TTTAAAATTAATAAATTTTTGTTTTTAAAGAATTAGAATAAATTTAAGATATTCTTATTATGTTAATTTAAATACAGTTTTGTTAAATTTTTAATTTAATTTCTATTGTAAATTTATCAAATATTCTAAAACAAACTTTAGATTGTTCCAAATTTAATTTATAATTTATTTTTTACTTAATGAAAATGATATTAACAATATAAACCAACCTCTCTTTTGCTATTTGAATGATTTAAAAATAATGAAAATTTTAATAATGACTTAAAAGTAATTTAATATTACCAATTTTTTGAAGTAAATATTTAATCTGCTTTACTTTGCTAAATAAAATATTAACTTCACATAAAAAGGTAAAAAATTTTAATTTAACTAAATAAAAAAAGAATAATTTATTTATTTCATCTTATCTTTTGCT

>Cbs_1L-13_1A

TTATTTATTTTTTATATAATTATTCATATAATTTTTTTTATAAGCTTGCTTCATAAAAAAAAAAATTAAAAAATTTAATAGAATCTTTTAATTTTGTATTTAAAAACTTATTTGAAAAATATTTTTGTATAAAATTAAAAAGTATGTTTAAAAAATTAAAACATAAATAATCAAATAAAATAAATTTAAAAAAATAAATAAAAACCAACCTCTTTAATTTCTCTTTTATTTGCAGATTAAAAATTATAATTAAAATAAAATTTTTGGAAATTACAATCATTTCTTTACAAAAAGGTATAAATTACATTTAAATTAAAAAATAGTTAAATTAGAAAATAGTTAAATTGTTTAATTATATAATTAAACGATATTGTTGAAACACAAAAAGTTTTAAGGTAATTAATCAAATGTTAAT

>Cbs_1L-14_cons

ATTATAATTGTTTAATTGGCTATTTTTTATTGTCAATACAATTTAAAAGCAATAATTTTTAAACTTAATAAAGTTTTTAACTATGATGAATGTCATGTATTTTTATAAAATTTTTAGTATAAAATTCAATTTAATTGATATTTTGAGTGTTATTATTAAAATAATTTTGATGAAATATTGTGTTAGTTAAATACTTAAAATAAACCAACCTCTTTTGAAAATGACAAATATTTAAAATTAAAGTGAAATAATACATTTGTTTTAACTCTGTGTATTATAAATCTTTATTATTTTCATGAAAATTATTGTATCATTCAATTTTTTAAGCATATTTAATATTTAGTTTTTTTTATTTTATTGTAAAAATTAAAGTTTTATTTTAAAAATTATAGCAAATATAAACTTTTAATGCTTA

>Cbs_1L-15_cons

CTTTATAAGTCAAAGATATCTTTATTATTAATTAAATTTTACTTTTTGATCACTCATTCATTAGCTCATTAAAATTCTAATCAGTAAACTTTATAATATTAATCTAAATAAGAAGAAACCAAATTAAATAAATTATTTACTCTGGGTTAATTTTTAATTTTCATAGATAATTTTTAGTGCGAAATAGAAAAATGAGCAAATAAACCAACCTCTTTCCTACTTTTCAATATTTCTAAAACTTAAAATAAATTATTTTTTTTGTAGAAATGAAGATAGTTAGTATTTAAATAATAAAAAGATGATTTATTTTTGACTTCTCATAATTATTTAATTTTTTATAAAAAACAATAATTATTAATGAAAAATATGTTGATTTTTTAGGTATTATTTAAAAATAAAGAGAGTAAAAATATGA

>Cbs_1L-16_cons

CTAGCTTAGTCATTTTTGAGATTTAATTAATATTTTATGTTTATTCATATATAAACTATTCAAAATATTATAGAATTTAAACATTTTAACATCTTAATCATTCATAAATAACTAAAAATCAAAGTATTACATTCAATAAATAACTTTTACTCAATGTCAAAGAAATTATTAAATTGGGGTTTTAACTTATTTTTAAAAATTAAACCAACCTCTTTGTTTATTTAAATATAATTTATTTTTAATTAATTCATTTATTGATAATGCATAAGTAGCATATTTTTAATACATTATTGATAATTTTCTGTTACTAATATGACTCTTTTGAATATGAATGCTTGATTAAATCCTAATTTTGTGATATTCCTTCTTTCAGTTCAAACTATTTTTATTCAAATTCTTCAATTATAAAGATTTT

>Multi-Cbs_1L-17_cons, 1L-18_cons, 1L-19_13A; 505_bp

TAGCAGAATATAAACATCTAATTCTTATCTACTCAAATTTAAATTTTAAACCCAATTATTACTCAATTTTAAGTGATTTTCGAAGGTAAAATTCAACAACATAGTGCTGAAGGCTATAAAACTAACAACAAAAAGCAAAAAAACTAGCCTTCAGCACTATGTTGTTGAATTTTACCTTCGAAAATCGCTTTTAATATTAATAAACCAACCTCTTTCTTTAATATAAATTTAATTTTTGATTGAAATAAACCAACCTCTTTCTTTAATATAAATTTAATCATTGATTGAAATAAACCAACCTCATTTATTTTGAAAATCTAATTAAATTAGAAATTACAATATTTAATTTGTTTGTTCTCATTTTATTAAAATTATAATTTTAAATATTCTTTCTTATTTATTATCAAAATTTAAAGCCATTATATAATTTTTGAATTTATTTTTAAATTAATTTAAATAAAATAAAAGAAAAATATTCTTTTACTTAAAGTGCTTATTAATATAT

>Cbs_1L-20_cons

TGATTGTAATGGTATTCTGAAAATTTTTAATTTCAAAATTAAAGTGAGAAAGCTATAAACTTTCATAAGCCAATATTTGACATTAATAAAAAAATTATAATTTAAATGTAATAAACATTTATTTTGCTTAATGAAACTCACTTAAAAATTCAAAAGTGTCAAAAAACAATAAAAATATTTAATGAAGAATATTTAAATTATAAACCAACCTCTTTTATTTAAAAAAATAAAAAATTAGATTTGAATTATTTATTGCTTACTTTAAAAGTGTAAAAGGGATTTATCTAATTATAAAATAATTAAAATTTTTAAGTGTATTGAATAAATTGATTGATTAGTAATAATTAATATCTTTTGCAAAAAAAATAGAAATTTCTTAGATATAAAAAATAATAAATAGGATTGCATAAAATAG

>Cbs_1L-21_1A

TTTAAATTTATTTAAATCTTTTAATTAATTTAAAATGATCAAAAAAATTATAAAATGGATTTGTAGTTATTTTACTTTTGATTTTTAATTTTATATTTATAATTTTGTTAATTCTAAAATGTATTTGTTTATTTATTTATAATTGATATTTCAGTTTTATTTTATTTTTATTAATAAATAGATTCTTAAGAATTAAACATAAAACCAACCTCTTTTTTATTTGTTAAAATAAGTTTAAATAATTAATTTTTGTTCATTATAATTTATGAAATTAAAAATTTTTAATAGTTTTTACACTCCTTTGTACAATTTAAATTTAATTATTTTAAATATTTGTAAATTATTAATTTTAAAATCTAAAATTTATTAAAATAGCTAAGTTTTATCATAAACTGATAAGGAAGTAAGATAAAAT

>Cbs_1L-22_cons

AAAATATATTAATAATTCATTTTTAAAACATTTGCTTAATTATATTTCGAAGAAAGTTTAATAAATATTTTTACAATTTAAATCAATTTTTCTAAAATCATTGAAATATTTTACTTACCTATCTCAGTTTTTAATTCAAAAATATTTGATTAATAAATCTTAATATGGTTAAAAATTAATAAAAAAATAATATTAATAAATAAACCAACCTCTTTATTAAGCTTTAATTTATAAAAATTAGTTTTGACTAAAATTTTAAATATTTGATAATTTGTGAAAATTGTTCGTTAAATTTAAAAAATTATAGAATAAAAAAATATCTATATTTATGAAAAAATATGATTTATTATATCATATTTTTAAAACAAAACACGAGTTTGTTATTATTTAATTAATGAAAATATATTTAAAAATT

>Cbs_1L-23_1A

AGTCTATTACTTCAATCTCAATAGAATTTAATTTATTTTTTTGTTTAATTTTGATAGATAAATGGCATTTAAAAATGTAATATAATTTGAAAAAATTTTTGTGATTTTTTAAGGTATTTTAAAAATGATTTATTAATCTCACATATTACTAAAATTGAATTCTTTTTATTTTTGAATTGTTAATTCCTTATAAATTAAATAAAACCAACCTCTTTATTATTTAATTTAATCTTCTATTTTTTCTAAAGTTTAAAAAAAAAGATAATAATTTCTAATAATTAAAAATCTATTAAAAATATTAGATTAATGATTCTTATTTAAAATACATTAAATAAATTTAAATTCTTTTTTATTTTTAGATTAAAAAAATTTTTAAAATTGTAACAAAATGATTAAAAAATTAATTTGATTGATA

>Cbs_1L-24_1A

CTTATAATATATCTGTTATATATTCCTACAAATCTAGTTGTAATTTCCCATTTCAAAATTAAATTATGCTAATATATATTCTTTTATTTTTGATTAGGATTGATTGTTATTTTTGTGAAAAAAAATACTATTTTCTAAAAATTTTTTTTTTTCAATAAAATAGAGAAGTTTTTCTTAATTTATTTAAACTTTTATGAAATAAAACCAACCTCTTTAATTATATCTATGAAGCTGAATAACACATTAAAAGAATTTTCAATTAAATATTAAAGATATCATAACAATTAAATTTAAATAAAATTCAATTATTGGTTGTTTTAATATAATAAATTTCTATTTAATATGAAAATAATTGCATTAGAATTGTTGATTTTTAAACAATTTTAAAATAGAATTTATTGGATTAAAATTTAAA

>Cbs_1L-25_cons

TATTTAAAATTAATTTAATAAAACTTAAATTATGAATCTTTTAGTTATATTTTAAGTATTAATTGATGTCAAATTCAAATTAGATTAAGGTAAAATTTAGTAAAATAATTCAATTTTATATAAAAATTTTAGAAATTACATTTAAAAATTTATGATTTTTAAGATATAATAAAAAATACATAAATAATTTACTTGCTTATTAAACCAACCTCTTTTTTACAGAAAAATAGAGAAATATTTATTTAATTTTATATTTAAATAATTTTAAGAAGTTAAAAATGTATTTTAAATTTATTGTGAAATACCAGTAATGAAGTTTTCGTTTTTTATACTTTTATGATTATTCAATAGAAAGGTAATATTTTAATTACTTATTTTTAATTAAAAGATGGATAAAATTTTTCATTAGTGATTA

>Cbs_1L-26_1A

TCTGCTGATTTACCCTTTGCACTACTTTATTCTTTCTTTCTTTTTCTTTTTTATTATTGATTTATTTAAAAAAAAATAAAATCAACAATTTTATAATTTTTTATGATAACTTAAACTAGTGATTGAAAAATCTTTATAATCTTAAAAAAATAGGCTTACTTTTAAATTTATTTTATAAATTGCTATTTATTATTAATAAAAAAACCAACCTCTTTTAATACTTATTTTTGTATATATTTTACATAATATTTTCCTTATTTACATTTAAAATTTAAGAAAAAATATGATTTTTTGGATTTAATTTTTATATTATTTATCTAATAAATTATAAAAAGATAAATAAAATTGCAATTAATTAATTTTTAAGTATCAATCTGACAGCTTACTAAGGATTAAATTCTATTATAAAAATTAA

>Cbs_1L-27_15A

CTTATCCAAAATAAAACAATATGTTTTTTTTACTTTTTAAAAAGATTTTTATCTAAATTCGAATATAAATTTACAAGTTCCTAAAAATAAAAATATTTTTTTATTTGAGGAAATAAGTTTATTAATGAATATTTTTAATAGTTTATTATAGATTAGTTTCAGTAATTAATTAAAGATATCCTAATTATTATAAATATAAATAAACCAACCTCTTAAACTATAAAAATTAATAAATTACAAATTAAGAATCTTCCTATTATTAAAAGATTGCATCATTACCATTAAAAAAAATAATAAAATGACCATTAGTTAATAATAATTAATAAAAAGAATATTATATTTAATTCTAAATATATTTTTAGAAATACTTAATTCAAACTATTTTATTATTTAATTAAGTTAAGCAAATAAGAAT

>Cbs_1L-28_cons

AGGTACGAATAAAAAATAAGTATTATAAAAATTTTAATTTCTAAAAAATTAAATTAAAAAAATATAGAAAACAAAAAATATTTAAAAATTGTTTGATTTTGATAAAAAACAACTGTTTATTAATTTCATAAATAAATTAAAAAGAATTGTCCTAGAATATTAAATCAATTAATTAAATTTTATTAATTATTTAATCAAATTAAACCAACCTCTTTTTTGTTAATCAATAGTAAATAAATTAATAAGAACCTTTATAAACTTTAAAATTTAGAAGTTTTTATTTCATTGAGTATAAATTATTTATTATTTTATTAAATTTTAATGAAGGAATTTCTGATTATGGTAAAATAAATATTTTTATAAAATATTTAAATTAAAATAAATAGAAATACATTTATATATTTTGAGTATTGTT

>Cbs_1L-29_cons

TCAGTGATCTAATCCAAGAGATATATTTATATTTATTTATTTACTAATTAAGTATTATCTATTTATTCATTATGTTAATTATAGATTTAAAAAAAAAAACTTTGATTAAAAAAATTCTTTATTTTTAAAAAAATAAAAAGAATAGTGTTATAAATTTTAAAAAGAATATTAAGTAAGTTTTATTATTTATGTAATTTAATTAAACCAACCTCTTTTTGATTATTATTAATCTAGACTCTTAAAAATTTTAATTTCTTTTTTAAAATTTTGGAGAAAGCTAAATAAAATTTTGTTTGCTTCTAATTATTAAAAAATTAAATTGGTCAAATTGTTAAAAAACTGAGTAGATAATTTTTTATTAATAGAACAAGACCACCTTTATGGAAAATATATTAAAATTTAGTTAATTTAGTGT

>Cbs_1L-30_11C

TTAATTATATTTATTTTTTTGTTTGTTTGTTTGCTTGTTGATACTAATATTTAATTATTATAAAAAATGATCTTTCTTATCAAAATAAATTTACTTGAATTTTATCAAAAACTTTTTTGTTGTCTTTAACTATCTAATTTATTTTAAATTTAAAAATCCAAAAATTAAATACAATTTTGATTCACTGAGATATATCAAATTAAACCAACCCCTTTTAATTTAAGTTATTATTTTCTTCGCTTAAATAATATTTTATTTAAATAAATAGCTTTAACTTTCTAAAAAACAAATTATTAATAAATATTATAAAAAAAGAAATTGTTTAAAATAATAATTATGTATTTTATTTTTTTCTGCTGTCTATTTATGAATTTTTTTTGGTTTTAAAACATATCTAAACTTTCAATTTTTCAAA

>Cbs_1R-1_cons

TACAAATATATGGCTTAACTTTTTAATTAAAATTTTTTTATAAACCGCTTTTTAAATTTAGAAAAAAATTTATAATTTTATTTATTTATTTTTTTTTATTTTTGAAAAATAAAAAACTAATTAGAAATTTGTTAATTTTAAAGGGAGTATTTTTAAATTATACAATTTAAGTAATTATTTTTCAATATAATCTTAAAGCATAAACCAACCTCTTTTTTTTAATAATTTAATTTAGTTTATGAAAAAAAAATTATTAAATCAATTTATTTATTTATTTTAAGAAAATTTTTTTTAATTTTAGATTATTTTTATTAAAAATATGTAATTTAAACTTTAAAATTTTTAATATTAATTATGAAATTAATATATTTTTTAATTTTAGATATTAAACTAAATTAGATAAATAAAACTTAAT

>Cbs_1R-2_cons

AAATATATAGCTTAACTTTTTAATTAAAATTTTTTAAAAAACCGCTTTTTAACTTTAGAAAAAAATGAATAATTTTTTTTATTTACTTTTTTTAATATTTTAAAAATAAATAACTAATTTCAAATTTGTTAATTTTAAAGGGAGTATTTTTAAATTATACAATTTAAGTAATTATTTTTCAATATAATCTTAAAGTAAAATAAACCAACCTCTTTTTTTTCAAATATTTAATATTAGTTTATGAAAAAAAAATTATTAAATCAATTTATTTATTTATTTGAAGAAAATTTTACTAAATTTTAATTAATAATAAATTTTTTTATATAAAAATATATAATTTAAATACTAAAATCTTTAATATTACTTATGAAATTAAAATATTTTAAATATATGATATTAAACTAAATTATAAAAA

>Cbs_1R-3_cons

AATTTATTTTTTTTATAAACCGCTATTCAATTTTTTTTTTATAAACTGCTATTCAATTTTAGAAAATAATTTATTAATTTTATATATTTACTTTGTAATATATTTAAAAATGAATAACGAATTTATTAAATTTTTTATATTAAATAAAGTCCATAATAAAAAATAATTTAAGTAAATTTTTTAAATATTATTTTAAAGCATAAACCAACCTCTTTTTTTTCTATAACTTAATATTAATTTATGAAAATAATTAATAAATCAATTTATTTATTTATTTTAAGAAAATTATAGCTAAATTTTAATTTATAATAGATCATTTTTATATAAAAATATTTAATCTAAATTTTAAATGTTTAATATTAATTATGAAATTGAAATATTTTTAATATAAGATATTAAAATAAATTTACATATT

>Cbs_1R-4_cons

TATATAGTTCAACTTTTAAATTTGTTTTTTTTATAAACTGCTTTTCAACTTTAGAAAAAAATTATTAATTTTATATATTTACTTGATAATATATTTAAAAATGAATAACTAATTAATTAAAATTTTTTTATTAAATAAAGTAAAATTAAGTTAAAAATAAAAAAGAATTTAATCATTTTTTTAAATATAATTTAAAAGCATAAACCAACCTCTTTTTTTTCAATAACCTAATATTAATTTATGAAAATAATTAATAAATCAATTTATTTATTTATTTATTTTAAGAAAATTTTTCAAAAGTTAAATTTATAATAGATAATTTTAAAATAAAAATATGTAATCTAAAAGTTAAAATTTTTATTATTACTTATGAAATAAAATATTTTTAATATAAAATATTAAAATAAATTTTTTA

>Cbs_1R-5_cons

AGATAAAGCTAGCTTTTAAATTAATTTTTTTTTATGAACCACTTTTTAACTTTATTAATATTTTTTTAATTTATTTATTTAATTTTTATCATATTTAAAAATAAATAACTAATTTATGAAATTTTTATATTAAAGGAACTATTTTTAAATTAATTTCATAATAAAATAGAGTTTAATTTTTAAAATATAAACTTAAAGCATAAACCAACCTCTTTTTTTTCAATAATTTATTATTAGTTTATGAAAAAAAAATATTTAATCAATTTATTTTAAGAAAATTATTTTGTAAAAATATAATTTATAATAGATTATTTTGATATAAAAATATGTAATTTAAACATTTAAATTTTTAATATTACTTTTGAAATTAAAATATTTTTAATATAAGAAATGAAATTAAATTAGAAAAATAAAA

>Cbs_1R-6_cons

AAAGCTTAGATTTTAAATTTAATATTTTTTTATAAACCACTTTTTAACTTAATTAATATTTTTTTGATTTATTTATTTACTTTTTATCATATTTAAAAATAAATAACTAATTTATGAAATTTGTTAAAATTAAAGGAATTATTTTAAAATTAATTTCTTAATAAAGTAGAGTTTAATTTTTAAAATATAAACTTAAAACATAAACCAACCTCTTTTTTTTCAATAATTTAATATTAATTTATGAAAAAAAAATATTTAATTAATATATTTTAAGAAAATTATTTTGCAAAAATATAATTTTTTTTTGTTCATTTTTTTTTTTTTATATGAAAATATAAAATAAACATTTAAATTTTTAATATAAGATATTAAATTAGAAAAATAACAGTTAATTTTATTGCTTAAATAAGTTAAG

>Cbs_1R-7_cons

CTTAAATTTTAAATTAAAACTTTTTTTATAAACCGCTTTTTAACTTTAGAAAAAATTTCCATAATTTTATATATTTACTTTATATTTTATTTAAAAATAAATAAATAATTTATGAAATTTGTTTATTTTAAAGGAAGTATTTTTAATTAATTTCATAAAAAAATAGAATTTAAGTAATTTTTAAATATAATCTTAATGCATAAACCAACCTCTTTTTTTAAAATAATTTAATATTACTTTATAAAAATAAATTTTTAAATCAATTTATTTATTTTAAGAAAATTAATGCAAAAGATTAATTTTTAATAGAAAATAAAAATATAAAATTAAAATTTTTAATTTTACTTTTGAAATTAAAATATTCAAAATAGAAGATATTAAACTAAATTAGAAAAATAAAAGTTAATTTTATTAC

>Cbs_1R-8_cons

TTTTTTGATTTTATGGATAATATAATTAAATGTTTACTTTTTTAACTTTTAATTTAATTCAGAGAAAGTAAGCTCTAATATTGGTAGAAAATTTAAATTATATAATTATACTAAAAATATAAAATTTAATTTTTTAAAAAATTTTTATTATTTTTACTGGAAAATGATAGAATTTATTTATGAGATAAATTTTTTTTATTTAAACCAACCTCTTTTGAAATGTCAAAAATTTATTAAAAATCTTTTAAATTAATTAAAATTTTTATTCCTTTATTTATAATCAATTAATAATAAAATCAGCTATTTTATTTAATTAAATTAAGAATATTACTCAATTTATTGAAAAAGCTTGAAGTAATTAAAAAATAAATAGTTTTTCTATCTACTGAAATAATATTGAAAATAAATAAACATA

>Cbs_1R-9_1A

AAAATTTTTAAAAACCCCAAACAAATTTGGATAAAATATTTTTTTTCTACAATTAATTAAAAAAATAGTTTGTAAAAAATGTTTATTTTATTTTGAAAAATTATATTTTATTTGCTATTTGATAATTTTTGCAAAAAAAAGCTAACACCCTCTGCAAGCTCGCCTCCCCCACCCACCTGCCTCAGCAAATCCTAAATAAAAAAACCAACCTCTTTTAAATTTATTTAATTTCAATTTTAAAATAAAAATTCAAAAATAAATAGAAAGCCTTTACTAATAAATAAAAAAAAAATAGAAAATTGATTTAATTTTTTGATTAAAAATTAAAAATTAAGTAAATTAAAACTTTTATTTTTCTAAATTATATATTAAAGCATGTTTAAATGAAATAGAGCTAGAGCTAAAGTATCTAAAT

>Cbs_1R-10_11C

ATTAAGTAAATTAAAACTTTTATTTTTCTAAATTATATATTAAAGCATGTTTAAATGAAATAGAGCTAGAGCTAAAGTATCTAAATACCTAAGATTTTCTAAATATTAATAAATTATTATAAATAATAATCATAAATCTATTTTAATTTCTGTTAGAAATAAAATTAAATAAATATAAAATTATTTTTTATCATTAATCATAAACCAACCCCTTTCTTTTTAGATGGCTATAAGAATATTGATTATTAAAGAGAGTCAATGTATATTTATTTTTTACATTGTTTTTTATCGAGTAAAGTATTTTTTTAAAAGAAATTAGATCTTTTAGAAATATCAATATAAAATTGCTAAAAATATCAAATAAATGGAAAAAAACAATTAAATGCCTGATTCTATAAAGCAGTCCCAGATAAAA

>Cbs_1R-11_cons

AATTTAATTAAATTATCATCATAATTTTAATTTAATAGAAAATTTGTAAATTATAAATATAAATTATTTTAAATAAATAAAAAAGGATAGACATTTTTGATATTCTATTTTTTTAGTAAATAAAAAAAATAATTTAATACCATATTATTAAAAATAATAAGATATAATTTAATCACATTAGTTATAATCCTTTATTTAATTAAACCAACCTCTTTAATAAAAATTTTAATAAAATTGATTTGAAACTTTTAAAACATTATAAAATTATAAATTTAGCTTAAAATATTAATTTAATAAAATATTTTTAAATGAATAATAAAATTTCTTTAAAAAATAAAAAAATAGGTTTTTTCATTTAAGTTTACAAATAAATATTTTAATTTATTTGATAAAGAAAATATTTTGATTTTAAAAT

>Cbs_1R-12_cons

ATTTTAATATTTAACTATTTTTTTATTTCAAATTTTATGTGAAAATATTCAAGAATTTAAGTTTTTAGTATCTATAATAAATATTATTTGAATTACTCTATGACATTAAATATAAAGTATATGAAGATAAGATTTATTTAAAAAAAATTAATTTTTTAATCTTTCAGTTTGTTTCTTAAATTTATTTTATTTATCTAAAATAAACCAACCTCTTTTATAACTTATAACATATTTTTTATTATTAAATATTACATCTTTACAAACTTAATAATTTAAAACTAACAATTTATAAATACATTCAACATTAACTTAGATTTTTATCTTTGAAAATATTATATATATTTAAAAATTGTTTTTAAAAAGAAAATATAAAATTTCTCAATTCAATTTTTAATTAAATCTAAACAAATAAATG

>Cbs_1R-13_cons

AGTATATGTTTTTATTGAGATTTTCAACAAATAAATTTAAAAATTTAAAATAAACTAGTTTTAATCTTATTCTCCATAATAAATAAATACAATTTAAATTACTAAAAATTTAAATATTATAAAATAAATTTTAAAAAGAATTAAACCAATAATTTCTACTTATTAATTAAAAAGTTATTTATGTTAATTTAATATGCTATTAAACCAACCTCTTTTTATAAAAAATTTTTTAATAATTTAATTAAAAAAAAGTTGGTTGAAACAAAAAAATCAAAAATTAAATATAAATTTAATAATTTGTTAAAAACAGTATTAGATTTTAGCATCTAATTAAAAAATAAAAATAATATGGATTAATTAATTTTTTGTAAAATATTTTATATTGATATTATTAATTATACCATAAAAAAAAATA

>Cbs_1R-14_14A

TTTTCGCTACATATTCTAATATTCCATCTTCCAAATTTTATACTTTTTTCTGAAATTAGCTAAACATAATTCAATTTTTGTAATATTCTATAAATTAAATTTTTTTAATTTTTAAAAGTTGATTTTTAATTATGAAAAAGAGTCAGAAAAAGTCAGAATAAATCAGAATAAATTTTAAATAGGAAGAATTCAATAAAAATTAAACCAACCTCTATTTAAATATCTAACTAAAATAAATAAATAAATTTTTATTTAAATCATGTTATTTAATTTAAGTATTTTAAAATTTATTTTTGTATTTGTGAAATATTTTTTTTATTTTTATTTTTAATCAAATTCTTTTTTTTTATTCTTTTTAAAATAATTATAATTAGTAAAGCAAATAATAACAATTCCAAGCGTATAACTGTTAAAA

>Cbs_1R-15_cons

GAAATTGATATTAATTATTTTCAATATTTTTAAATTGAAACTTTGTTTAGATTTTAAATTTTTTATAAAACTATTAGTGATTTGACAATTTTAATTATTAGGAATAATTTATAAGTTATTTAACATCTAAATTACTTTAAAAAATTTTCAAAAATTTTTTTATAATCCTTAATTTTACTATTACTTAAACAAATATCTATTAAACCAACCTCTTTATATGCTTTTATTAAACACTTTTATTAATTTTTATAAAAAATAAACTTATAATTTTTAGATTTATATTAAATAAAAATGGTAATCTCATAAAATAGTAGCTAGGTATATGAGATTTAATCTAATATACTACAAACAAAAGATCCATAGAAATATTTAGAAATTCAGAAGATTTATTTTTTATTTATGAATTTAGAAAAAT

>Cbs_1R-16_1A,15A

ACAATTAATATTTAAGCTTCAAAATATAAATAAAAAGATTTGTAGGCAATTTATAAGTTGATTTAATTTGAAATTAATTTATTATTCTAATTAATAAAAACTTTTAAAAAAATATTACAGCTTAAAGTTAGATCAAACTTATCAATTTAAAATTGACTATAATATATATTAAAAATATTATTGATAAATTTAGGAGAAAAAAAACCAACCTCTTATTTTTTATAAAAAGTAGATTAATTTTTATACTTTTTTTAATTAATTTTTCAATGATATCTTATCAAAATTATATGCTAACATTTGATTCTAAATAAAATTTACAAAATATAAATTTAAATTAAAATAAAATAATTCATAAAAAGATAATTAATTTTTGTTTAAAAGTTTATGATTAAACGAATTAGAGCCAAAATTGTTT

>Cbs_1R-17_1A

TGTAGAACTTTTTGTTGTTAGATTAACTAAAAATAATTTATTAATTAAAACTTAGCAAAATTTTTGAATTAAATATCAATTACTAATATTCAGTTTAATTTTTTTAATTTGATAGTATCTTAAATAAATGTTATTTTAATCTTTAAAAGTAAATTTCTTATATTAAAATACCAATTAAAAATAATTATTATCTTAAATAAAAAACCAACCTCTTTTATAAATATGAAAAATGTTGTATTTATTTTTTAGAATATTTTATAATTTAGAATCTTTAAATAAATATATATTTTTAGATATCTAAAAGTCAAAAAATTTAGTAAAGATTTATGTAATATTTATTTTAAGACTAGGAAATTTATTTTTACGGTTTGATTTTTTTAATTTCATTGTTATAAAAATTTTATCAGTGTTTTTT

>Cbs_1R-18_cons

AATATATTTCGAATAAATTTATTTAAAGAAATTTCAGAAAACTTTTATTAGTTAATGACATCTATAAATATTTATCTATATTAACTAATTTGATTTAGTAAAATGTAAATTAAAAAATTCCAATATAATAAAGATTTATAAATAAATAATTACAAAAAATACTATTTAATTTTTTTAATTTAATTTTAAATTACTTCTATTAAACCAACCTCTTTATTTTTCTTTATTTTGGTTGTAAAAATATTTTTTAATTCTTTTTGTAATAAATATGAATTATTGATATTTATTAATTATTATTAGAATAAGAATTAATCAAGCTGATAATAAAAATTTTAGATTTTATTCAATATTATATCTAAAAAGTGATAAATTAGATTAGTCTATTAATGAAGAAATAAAAAATTATGAAAGATTT

>Cbs_1R-19_1A

AGAAAGAATATGAAAATCATAATTAAAAAATTAAAAAAATATTTTAAATTCAAACATTTTATAATTAAAACCATTCATAACAAGAAAATGAATAATAAATAAATTAGAAAAATTAAATAAAAGTTAAATGTTTAATTTTTAATTTTTAATATTTAATAAATACTTATTAATTATTTTTTTGCAATTAAAAGAGAAATAATAAAACCAACCTCTTTTTCTTTTTTGAAATTATAAAATTTAAATTAATGCTAAAAAAACTTTATGATATAAATTCATTTTTAATATCATTTATTAATAAGATACAAAAACAAAAAAATTAAATATTTTTAATAGAATAAAAGTATATTATATTGTAATAATTATCATTTTTTAATTCTAAAACTAATAAAATTTGAATATTTAAAAATTAGCTAAA

>Cbs_1R-20_15A

ATATTCTTCTGTTCAAAATATTTGAATTTATGAATTACGTGTTATAGAATTCTAAATTTGAAAGAATCAAACAAATATTTTTTTAATGATTTTGCTAATTAATTTAAAAATCTTTCATTATTTTTTTATTTCAATAATTAATTAAAAAAATATTTATTTAATTAAATAAGTTCTATTTTAATTTTTATTTTTATTATTAATAAACCAACCTCTTAATTTGATAATTATTAAAATTAACAATTTTATTAATTAAAAAAAAATTCTAGAAAAAAAAGATATTATTAATAATGTCTACTATTTGTTAATATAGTACCAAGAAAATTTCATTTTAAAAATAACCCAAAAATTAATTATTTATAATAATCAAAGGATTGATCTATAATTAATTTGAAAAAAAATTGATTTTGAAAAAATT

>Cbs_1R-21_1A,11C

TGTACTCTTTACAAATTTATTTTATTTTACATGTTTTCCTGTTTTTTACATTTTTAAGATATACAATTTTATTTTATTTTGATATTTATGTATGAATGAATGAATGAATAAATAAACATGTTGATATAATTTAACTCTTATTGATTATAAAATAAAAGTTTATTTTTGCATTTATAATTAGAACGATTACCACTCTCAAAAAAACCAACCCCTTTTTAATTTTAATTTCATGCTAAAATATATTTAATTAAATTAAAAAATATTTAATTTTTGAATTAAAAAGAACTCAAATAAAATAGTTAGATAGATTATTTAGTTTTCTTATCATAAATTAATTGCTACTGTTTTCAAATAATTAAATAAAAAATAACTTTTATTTTTTTTCTATTTAAGAAAGCCAATAATGAATTATAAA

>Cbs_1R-22_1A,13C

TTAAGTAAAAAATTTAATATTTACAATTATAATTAAATTTCTTCTATTACCAATAATTGTTAGCAATAATAAAAGTAATTCTTAGTAAATTCTTAAAATCTGATTAACTACTAAAAAAATACATTTAGCAATACTCTTATTAAATTATTAAAGCATTTGATTTAATTAATAATAAAAAGTAAATTTATCAATTAATATAAAAAACCAACCTCCTTTTAATCTTTATCATTTAAAACAGATAAAATATAACTATTATTCTTCTAATTTAAGTAATCTTTTTCATATAATTCTTCAAATTGTTAATAATTAAAGAAAATTTTTTGGAGTTTTTAGAATTTGATAATCTAAAATAGTCAAAAACAATATTTATTTATTGTCAATTTTAAAAATTCAATTTTATTGATTGATAACAATC

>Cbs_1R-23_13A

TTTTTTATCTAAATAAAACCGCAATTTATTTTTAAATTAATTAATATATTTTTTAATAACTATGAATATACATTAATAAAAATTTATTGTGAGATTTAATTATTAAATTCAAAGCAAATTAAAATTACAAGTCAATAAATTTTAAGATTTTTTAAAAAAATAGTTAAATTTTTTATCATGGAATAAATATTTTTATGAAATAAACCAACCTCATTATATTTCAAATGATTATCTAAATTTTAAAGATATATTTTTATTTAATTAATTTAAAATATAATTTGATATTTTTATTAAATTAAAAATAAAATTTTTTTATTTAAAGAATTAAATTATTTTTTTAGTATTAAAAATTATTATTTCTAATTAGAATTATTTAACAAAAATATCTACTTTTTATTTAAAGTAATAAATTTAT

>Cbs_1R-24_1A

TTTTAAATATGTTGTAAAAATTTTTGAAAATAAACAAAAAAGTTATAAAAATTAGAGTTATCAACTTATGGAAACACTTGAATTAGAATTTTTCTAAATAAGAAACTTAATTGATAAAAAATTTATTTATTTTAAAGTAAATAATTTTTTTCATGTTTGTAATTAAATTAAACAAAAAATTAAATCTTTGAAAAAAATTTAAAACCAACCTCTTTTCAATTTTTTTTCATTTTCTTTTTAATTTGAAATTTTTTTATTAATTTTGATAATTTAATTTATAATTTGCTTTAAAAGGAACGAATATTTAAAAAAATAAGATATTACTTAAGTGTTTTAGTTAATTTTACAAAATATAAAAAAAATTAAAAATAAAAATAATTCAGCATTTGGTGTTTTAAAACTTAAAAAAAACATT

>Cbs_1R-25_1A

ATTATCAAATAAATATATATTTTTTTAAGATATTTGTTTATTGTAGTAATAATATCTTAAGTAAAATATTTATTTATTTAAAGCTTTAGCAATCAAAGTAAAACTATATAATGGAAATGATTATAAAATTTATCATTAAATCTAAATTACATCTAATTTTATTTATAAGTTAAAATAATATTTTATTTATTATGAATTATAAAACCAACCTCTTTAATTTTTATAAATAAAAATATTTGTTTAATCTAAATATCATTGAGAAGATATATTTCAAAATTTTAAGACAGTAATATTTAAAGTTACTCATGAAAGATAAGATAAACTAAAAAATAGTACTAGGGAAGAAATAATTTAATTAGCTTAAAATTAGTGTAATTAAACTTTTTTGATAAAGTTTTTATTTAAAATATCTTGT

>Cbs_1R-26_1A

ATATAACTTGTATAATATTTTTCTGTTAAAATAAACTTTTAAATATATAAATATAACTAATATTATCATTTTAAATTAAATTTTCTTTATTTATAAATTTATTGTGTTTTCTCTAAATTTTAACAGATTTTTAAATCTTAAATTTAATTTAAATATATTATTAATTGTATTAAAAAAATTTTTATTATTTTTAGGAAATAAAAACCAACCTCTTTTTATTTTAATTAGAGTTAAATGATTTTTTTTTTTTAAATATCTTTAAAATAATAATTTAAGTTTAATAAATATTATTTTTGCTTCAATATATTAATAAAATTTATTGTAAAAATGAAAATTTTTATTTATACTAATTTTTCCATTATTTTTATTTTGTTTTATTAATTTTAATTCCAAAAAAAAATTAGATAAGTCCAGT

>Cbs_1R-27_15A

AATTTTTATGTAATAATATAAAATCTTAAAATAAATAAAAAAGTTCTATCTATCTTCTCGAATGAAGCTCGAATAAGTGAAATTGTAAATTCTGTATGAATATGTTCTTAATTTATTCTACAAAATTTCTAGATTATCTGTATTTCTATTTCATTGAAAAGCTTAAATTATTTTAATAATTTAATATTTTGTAATGCAAATAAACCAACCTCTTATCATTAAAAAAAAAAATTTTTTTATTTAAACTATTTATTTTTTTATTTTTCTTATCAATATTACACTCTTTACCAATAAAAGCTGTGGTAAAAATAAATATTTTATTTAGAGAAAACTCATTATCAATGTAATAAAAAAGCATTTGAAAGATATACTAATAACTTAATAATTAGCCATCAAAAATAAATTTATACAATTT

>Cbs_1R-28_cons

TTATTTTTTTTATTGTTCAATTTATTTAAAAAACTAACTTTTAATAATTTAAATAAACAAATAATATTTAAATTGTTTTTATTATTCAATTTAAAAAATGAATATTATCAAGTATTTTTTAACTTATCAAAATTTAATTTTCTGTTCACCACCCTAAAAAATAGTTTATTCTAAGTTTTTTCATTTTAAAAAATAACAAATAAACCAACCTCTTTATATAAGCTAAAAGAATAAATAAATATTTTTTTTAAATACTCCATTTAAAATTTATTACAGAAAATATTTTAGACTTCTTTTAAGCAAATAACCAAATTAAACATAATTTATCGACTCTTATGTAATTTTAGGATATATTAAAGTTAGCATTTAATAGGTATGAATTAATTAAATACCTATTTATACAAATTTATACTAT

>Cbs_1R-29_1A

TTAGTGGATCAAATTATATGTAATTTAAATAAAAATAAATAGCTATAAAAATTACTAATATTCTATATATACTAAAGTAAATAGGCTTTGCATCATTCAAAATCTATTTAAAAATAAATTATTTAGAAAATAAAATTTTTAAAATCCTCATAAAATATTTTTTAATTCAAAATTTATATATTTTATTTAATCTAAAGAAAAAAACCAACCTCTTTTACAAAATTATTTATTTCATTTTGTCAATTTTTAAATTAAATAATTATTTAAAATATATGAACTAAAAAATAATAGTCCAAAAATAAAAATGATTAATATTTTATTGACCTTAAAGTAAATATATTTTCCTTCATTCAATTAAAAAAAATTATTTACATAGCACATTTTTTTGTCAAACACCAGAAGTTATAACATCAGT

>Cbs_1R-30_1A

AAAATAAAAAAATTTATGAAATTTCACTTTAAATTAAAAAAATAAATTTAGAATTTATAATTTGTAAAACAAAAATTTTATGGTTTTCAAATCAAAATATTTTAAATTTTAATTTATCAAATAATAGAATATATATTTTATATTTAGAAAAAGTAATTTTTATACCTCTAAACTTAACCTTTTTTATTTTTTTAGAATAAAAAACCAACCTCTTTTACCTTTTATTTACATATATCTTTTAATTAAAAACAATTTGAATTTTTAATTTACCTTAAATTAATTTTCTATTATTTAATTTAAAAATTTTTTTAATTTTAATTTAAAGTAAAAGAAAAAAATACAATATAATATTTTGTTTAGCTAGCTAAGAATTAGTCTTTAAAATTAATTTGAGAGACGCTAATTTCAAACTCAA

>Cbs_1R-31_1A

GACCAAACAATCAAAAAAGTTCTATTCAATTCTTAATTCTTAACTTTGCTAACATTAAATTATTAACTTTGCTAACATTAAATTATTAATTAAATTTTTGTTTTATCATTTATTTTTGTAAAAATAAGATATAAAAATTATAAAGATTCCAAAAAAATATGATAAAAAATTTGTTAATAAATTTAATATTTTATTTTTAAAAAACCAACCTCTTTCTTTTACTGAATCAATAAAAATAAATAAAGATAATTTAATTTTTTTAAAATAAATAAATTATGGATGAATACCGATGAAAATTTTAGCTTAAATTTTTAACTTTTATTAAAAAAATTAAATTCTAGAAAGATTTAAGATATTTTAATTATTTTAAATACAATTATTGTAATTTTAATTCATTAATAATAATAGCTTAGGA

>Cbs_1R-32_14A

TTAATTAAAACTTAATTTTTTTCTCAAAAAAATTATTTATTATCGATTTATATTTAATTTTTAAGCTTAAAAATTTATTTTTCAATAGATCTAAAATTTTTATTTAATTCAATATTCAATTATACAAAATTTTAATTTTGTGTCAATAAAAAAGTTATTTATTTTTTCAAATGTTTTCTTAAAATCTTCTTTTCTAAAATTAAACCAACCTCTATTAAATATAAATTATATTATCATATTTGAGTTAATTTATTTAAAATATATTCAAATTAATTATATTGATTTAAATAAATTATTATTATTAATAATTTTTAAATTTGAATTTTTTATTTATTCCAAAAAAAATTCTTTAAAATCATTGCAAAAATTCACAAAACTAAACAATAATTTTACATCAATTTTATTATTTTAAAAT

>Cbs_1R-33_cons

AAGTTTTAAAAGTAATTTAGTTACTCAAATATCAAATTTCAGATTTTTAATAATTGCTCTAAAATAAAATAAATTTTCAATTTAAATAAATTTCATAGAATTTATGAGATAAAAAACAAATAAAATTTAATATTTTTTAGATTTTCTTTTTAATTTATTACATTGAAAAAATATTTAATTTAAGGAAAAAAAATTTTTTTTAAACCAACCTCTTTATCTATTTATTTATTAATAGAATACTTCAGAATATAATAAGAAATTTACTTATGCTTCTATGAATTTAGCTAGTATGTAAAAATGATTTTTACACCATAATTAACTTATTAACACTATTATAATAAAATAAAATATTCTATAATAATTTTTGATTTATGTTATATGCTGAAATTTAACAAACAATACTCAAATGAAAATG

>Cbs_1R-34_15A

TTAAATTAGAAATTTAGAACTTATTTTTATAATTATTATTTAAATATTTTATAACTTAAGATAAATAATAATAAAATTTATTATTCTTATTTTTGTAAATTGAATTAATAATTTAATCAATCATTTTTTCTAAAAAATTGGATTAATAATTTAATCAATTATTTTTTATAAAAAATTGAATTAATTTATTATAACTTTAATAAACCAACCTCTTAAAAATATTTAAGATTTTTTAAAACAAAATTTTAGTAATTAAATAAATTTAATTTATTTTTTAATAGAAAAGGAAAATCAGAAGGTAAAATATTAAATTAATGAATATTATTTAGATTGTAAATGATTAATTTTTTTCAATAAATAAATAAACAATTTAATAAGTAAATAATTAAGCTAAAAAAAAAATAGTCATTTTATA

>Cbs_1R-35_1A,11C

TAAACATGCATAGATTCTTAAGTTATTTATTTTCAAGATCACTAAAAAATATAAGAATGAAAACCTAATTAAATTTTATCATAATTTTAGCCTTTTTTTTTAGTTTTAGATACTTTATTTTTAGTTCTTAAAATAAATATTATTTTTAATAAACAATTTTTTAAAATAAAATATCAAAAAATTTTCTTAATTTGATAAAAAAAACCAACCCCTTTATTGCTTATTGAATTATTATATTACTCTTACTTATTTTAATTGTTATCAAAAAATATCAAATTTAATAAGTTTTTATTAAAATTATTTATTTGTTTTTTAATAAAATAAAATAATGTGTTGAATTTATTTTTCTTTTAAAATATCAATAAAATATTAAATAAAAATGGAAAAAAATTTATAAGTCTTTTTTTTATTTTTA

>Cbs_1R-36_1A,11C

TGCATAGATTCTTAAGTTATTTATTTATTTATAAGATCAATAAAATATAAGAATGAATACCTAATCAAATTTTATCATAATTTTAGCCTTTTTTTTAGTTTTAGATACTTTAATTTTAGATCTTAAAATAAACATTATTTTAAAATCTATTAAACAATTTTTAAAAATAAAATATCAAATTTTCTTAATTCGAAAAAAAAAAAACCAACCCCTTTATTACTTATTGAATAAAAACTATTATTATATTACTTTTACTTATATTAATTGTTATCAAAAAATACCAAATTTAATAAGTTTTTATTAATATTATTTATTTATTTTTTAATCAAATAAAATAATATTTTTAATGTAATTTTTTTATAAAACATCTATAAAATATTAAATAAAAATGGGGAAAAAATTTTATAATTCTAAA

>Cbs_1R-37_1A

TTAATTATGTAAAAATTAATTATTATTTTTCTTTTATTTTATACTTCATAAAAAATTGCTTTTCGCCTTTATTTTAACATTAATTTTTAATATAAAAATATATAAATTAATTTATTCATTTACAGTTATCAAAATTCTGAAAAAGAAAAAAACAAATTTTTATATTTCAAAAAAATTAAATCAAACTAAAATTTATTAAAAAAACCAACCTCTTTTATGAAAATAAATTAAAGAGAACAAAATATTACTTAGTTTTAATTAATTACTTAAAATAATTAAAATTTAATATATGAAAATAGATAATCACATAAACATTTTTTCTCTACCCGCTGTTCAGTACTTCTAACTACTTTATATAAAATAATTTATTAACTTTAGTTATAATTTATATTTAAAAATAAATCTTTTATTATAA

>Cbs_1R-38_1A,11C

TTTTTAAAGGTATAGTAAAATTGAATAGTATGAAATAATTAATTAAAATGTTAATTGTATGTTGATTAGATATTTTATCCTTCAAATAAATAAGAATTGTAAATTACCTTTTTGAAGAAAAAGATTTTAAAAGAATTTTAAACAAATTATAATAAAAAAATCATAAAAAGAAAATAAAAACTAAAAATGTTTGTTTAAAAAAAACCAACCCCTTTAGTGAAAATAAATTAATAGAAAAAATATTATTTTAATTTGATTAATTGATGAAAAGTTTTAATAAGTTTAATTTTTAATGAATTTCTTTTCTTCTTAAACTTTTATTTAAAGTTTTAGCAGCAGTGGTATAAAAATATGTTTGATAAGCTAGTAATAGCTGCTTTTAAATATTAACGGAAATATTATTCTGTAATGGATA

>Cbs_1R-39_1A

TGATAAAATAAATGCATATAATTTGCATTAATATACTAAAAAAAATTTAAACATTTTAGAGCAGATATGAGTGAAATAAAATAAAATAAAATTTGGATATTTCTAAAATAGCTTAAAAATTGTAAAAACTTAAATCAACATTAAAAAATAAAAATAAAGTATGATTAAGGTACAAAATATTTAAAATTAAATAATATATAAAAACCAACCTCTTTTTTTGAGACTTAATAATTATTCCTTCAACAAATAAATTAATAATAACATTTTATAAACTTTTTGTTTTGTAGAATATTCTACTTGATGTTAAAATAATTTAAAAAAATTATTATTTAATGGAGAAATTTTTAAATTTTCTATTTGAGTAATTTGATAAACTAAAGTTCAATTTACCATTAATTAATTAATTAGTGGGTCA

>Cbs_1R-40_cons

TCTTATTTCATCAAATATTTATTTTCAAGATTTTTTATTTTAAAGTAAATCTTAAAAAGAAAAAAACTTCTTTTAATTTTAGATAAATATATTTTTTAAAAATGAATTAATTAAAAATTTTTGTTTGTAAAAATAAAATACTAAATCAAATATTTTTATTATTTGCAAAATATTTAAAAAATTTTTGAAGTTATAAATTCTAAACCAACCTCTTTTTAAAATATAATATTAATTATTTTTTTCCTTAATAAAATTTAAAAAAAATAAATAGTGATATGATACAATTTTATTTAGATTAATTAATAATATTTTTATATTATATTTGAAAAAAATTTGAATTGATTTTTTATTTTATTTAGTAGCAAATATTATTTAAAATAATATTTGTTAAAAATATATTAACAACTAACTTTAA

>Cbs_1R-41_cons

AATGTATTAATTTTACTCTTTTTTAAACTAGCTAGAATTAAAATATATTTTATTTCTAATAAATATCTTATATTTATCACTCGAACTATAAAATTTTTTTTTAAGATCAATATGTCTTCCTAATTATTTTTTAAAAGAATTTTAAAGAAATAAAAATAGCAAATTTTTTTTATTAATAACTATTAATTTAAGATTATTCATAAACCAACCTCTTTAAATATGAGATTTAATGATTGATATTTCATATGATTTACTCAAAATAAACTTAAATTTAATTAATAATGTTATTTATAATTTAGAAATTTCTTTAGAAATATATTCAAAAAAAATTTTACAATAAATCAAGCTGATAAAAATATATCTTTTAATAAAAAATACATATCATAGAAAAAGTAAAGAAATATGTCAAAAGTTC

>Cbs_1R-42_cons

TTTAATATTTTATAAAAAAGTTATTAAGATAATTTTTTAATTCTTAATATTTTCAATAAGCAAAAAATGAGAAAAAAAATATATTTAGGAATTAAAAGATTCAGATATATAGCCATTGATTAGTGTGTGATAAATACTTTTAAAACTAATTTTAAAAAAATATATTTTCATTTAAACATCTAAAATTATTAATTTAAACTTAAACCAACCTCTTTTAAAAGCTTTTTTAAAAACAATATTTATTTTTAAGAATTATTTTCTATATTTCTATCTACCTTGCCAAGTAAATCTAATTTTAAATATTATATTTATATTATTCATAGCAAACTACAATAACTTTGCTTAATTTTTGACTTAACAAAAGATGTATATGCATACTTGTTTGTTAATTAATAGAAAAAGAAAAAATTTCTAA

>Cbs_2L-1_1A

TTTTTAGATAAAACCAAAAAAATATTATGCTTTATTAATTAATTTTAAATTTTATTCCTAAAAAAAACTAAGCTAATTCTATTTGTCTGTCACCAGTTATTTAGATAAAGATTTAAAAATGTAGTATTCAAATATAATTAAAATTAATATATTTTGTTTTAATTATTGTAAAAATAATAATTATTTTTTATTTATTTTTAAAAACCAACCTCTTTTTTTAATTAATAAATTTGATAGTTAATAAATCTTAAATTCTTTACAATTAATTAAAAACAATAGTTTAATTAAAAAATATGTCAAAGAAAAAAGTAAAAATGGAATTTTCAAAAAAATCATGATACACAAATTACAAATTGACAAAATAGTATTGCTATCAAAATAATATGTACTAAATAAAATTAAACTATGTTAAAAT

>Cbs_2L-2_1A

TCTAATAAGATTCAAAATAATTGATAATTTATTAGATCTGAAATATTTATTTATTTTTTTAAGAGAGATATTTAATAATTATTTATCAAATCTAAAATAGTTAATAATTTATTATATTTAAAATCAACTTATTAAAGTATTTAACTTATTTGATAAGATTATTTTTTAAATTTTAGTTTATTTTAAAATAAAGTTGATAAAAAACCAACCTCTTTTTTTTCATTTTATTTTAAAATATTTGAAAATAAGAAAAAGTTAAATTTTCTACTAACATTTTAATTTAAAATATATTTTTTTATAAAATATTTATTGATTTTTTAATAAAACTAGATAACTTATTAATAATAAATCAATTATTTTATACAAAAATTAAATTATCCATCTTTCATTTTCTATGCTTTATTCCATACATTTT

>Cbs_2L-3_cons

TAATAAGTAATAAATTATAAGCTACATTAAAAATGATAAAATAAAAATACTTTATTTGTTGTTGTTTATGTGAAATTAAAAGTAATATTTGTTACAGATTATATTTTTTAATTCAATTTAAATTAAATTGCTTTTGTAAATTTTAGTATTGTATTATTTAAAGATTTTGGAATTTATTTTTTTTAACTAAATAATTATTTTAAACCAACCTCTTTCTTTCTTTCTTGCAAAATAAATCAAATTATTTACATAAAATTTGCTCAAAATTAAAAATGCATGTTTTAAAAACCAAGAATAATTATTAATTTTATCAGAATGTAGAAGTAGATGATATTAGAGTAGGTAGGAAAAATTTTGTGCACATTTGAAGTAGACTGTTTAATAGATTTTCTAAGATAGAAAAAAAGTAAATATT

>Cbs_2L-4_1A,15A

AATAAAAAATCTAGCTTTAACTATTCCCACAAAATATTGAGATTAATTTATATAACTATCTAATGTTTATTCATAAAATATATTTTTACATTTTCCAAATGCTCCTTTTAAATTTGATATTTATCCGAAAAAATATAATAAAAAAAATAAACATTAATAAATATAATTTAATATTTAAATTAATTTATTAGGTATAATAAAAAACCAACCTCTTAAATCTTAAATGTGAAAAATTATATTTTTATATATGTAAAAAAAATATATTATTTTGGTAGATAGATATAAATTAAATGAATGACTAAAATTTGAGTAGTAAGGTTAATAAAAATAGAAGGATGTATGTATTTTCATAAAAATTTTGATATGAATGAAATATATAAAAAATACAAATTAATAAAAATTAAATTCATTGGAG

>Cbs_2L-5_cons

ATTTTTGTAATATCTGATATAGTAATAATTAGTTAATAATTATTTTTTTTAAATTACTCTAATTTTTTGATCGGATATATATATTTCAGCTTTTTCTGATTAACTTAATATTCTAAAATTGTAGTAAGTTTATTTGATATCGAATAATGCAATTTAGAACAGTAATTAAACCAAATCTTATAAATCAAATTTTATTTTACTAAACCAACCTCTTTTGAAAATTTAAAAAATATTTTAAATTTATAATTTTATTCTGTTAAATAATCAAAAAGATATTTTTTTCTTGGATTATGACAGTAGTTATGTGTTGTGATAAAAAAAAATATAAAAGCTAATTAATTAAGGGTTTTTTTATAACTAAATAAATAATTAGTTCAATAATATCATAAAAAAATTAAATCTAAACTATTTATTA

>Cbs_2L-6_1A

TTCATAATAAGTATGCTTAAAAAATATAATATTTGAAGGTAAATAGTAAATTTTTTAACTTTTTAAATTTAAGAAACCCAAATCAAATACTTGCAAGATCATAATTTAGACTAAAATTTTTTAAAGAATATCAAATAAAATTCAGATTCAATTAATATAAAAAAAACCAATTTAATTAAGAATTTGAATAGAAATCAATTAAAACCAACCTCTTTAAAATATACTTTTAACTTTATAAATAGATTAATAATTTTAATTATTTAGTAAAAACATGCTTAATATAATTAATTTCTACTGTAAATCAATATCAATATTTTAATTTTAAATATTTCATTTCATAAACAAAATTGATAATAACTTATTACTAATTTTATAAATTATTTTTATATAGCCAATTTTATAGATAGTGAATTAA

>Cbs_2L-7_cons

CAAGAAAATTTAACTTCTGAGAACAACATTTAGATAAACTAAACAAAAATTATACCCTCAAACAAATTAAAAATGTCTTGATGAACATAGGCACAAATTAAATCGTCGGTAAAAAGTAAAAAAATAAAAATTTGGAAATCCCAATTTCAGCACCAATAGCCACTACCCAGATACATTTTTTAAAAATAATAAATTATTACTAAACCAACCTCTTTTAAAAATTATAAATTTTATTAATAAATAAAAAAATATCTTTATATTTTAAAAATAATCTAAAAAATAATAATCTATCTTTATTAAATAAGAAATTAAAAATTTTCTAGAAAATAAAAAGAATAATTAAAATAAAAAAATTTATTATTTCAATATAATAATTAAATTAATAAAAAATTTTGATAAAAATTTTTTCTCAAAA

>Cbs_2R-1_14A

TAGTTTTAAAGTTAAGTTTATTTATCATAAAAAATATAATTGAACAAAAATTTTCTGTTAAAAATAATGAATAAATAATTTTTCGAAAATTTAAATTTCATTTTATCAAGAAAATAAATTAAATATTTATTTATTATTAAAAAAATGTGTAATATTTTTAAATTAAATAGGTAATTTTATAAATTAATTTTGAGTAAAATTAAACCAACCTCTATTTTATTTAATCAATTTACAAATAACAATATTAAAGTTATTTTATTTTATTAGTTTTTTATGTATTGTAATTTTTTTAAATTTTAATTATAAAAATTTTATTAATTAGTAATTTTAAAATTAATTTTAATTATTTTAAAGTAATAAAAAAATATATGCATCTTTAATATATCTACATACATAAATGATCTTTTTTTTTATT

>Cbs_2R-2_14A

AAATATAATATTTCATTAATTGATCATTTTAAAATTTATTCTTAACTCTTTTAAAATACTAAAAAGTATAGACGCATCTTAAATATACCTACCCAAATACATACATAAATGATCATTTTTTATTTAATCTATTTTGAAGACATTTTTAATATTTTTTAAATTAAATAGTTAGTTTTAGACATTTTAAGTAAAATTGCAAATAAACCAACCTCTATTTTATTTAACCAATAGTAAAAATAAAAATACTAAAATGATTTTATTTTATTAGTAGATTATGCATTGTAATTTTTTTAATTTGAAATATAAATATTTAATTAATTGATAATTTTAAAATATATTCTTAATTATTTTAAAATACTACAAAATATAGATGCATCTTAAATATACCTATCTACCTACATACATACATAAATAC

>Cbs_2R-3_1A

ACTGAAGTTTTATTTACGAAGATTGCTTATTAAATTTAATTTATTCCTTAAAGATAATTTTTTTTATACCAAAAATAAAAGTTAAAAAATTTTATATTTAAGAATAAATTTATTTACACAATAAATCATGTTTTCAAAAAAATCGATCCCTTTGGCATCGCTTCGCCTCGCCCTACCATCTCGTAATCAAAATTTGTTATAAAACCAACCTCTTTTAATTTTTTTACTTTATTTATAAAAATATATAAATTATTCCTATCAATATTTACCAGTAAATATGCTTCTGTAAAATTTTTCAATTATAAAAAATTTAGAAAATTAATTGATTAACTAAAAATAAAAATAATCAAACTTTTAAATCTTTGAAGGGTTTATATTATTATATTATGTTCTATTGATGAAAAATATTCATTGA

>Cbs_2R-4

TATAGTAATACTTCACTCAATATTTGACTGGCTAATTGAATATAATTATATTGAAAAATTTAAAAACATTTTATTTCTAATTTTAATATTAATGAGTATCTAAATTTAGTTTCAAAAAAAGAAATTTTTTAAACTGTGAATTATAGAAATAAATAAGCTAAATTTATTTGTAATTAAATAAACATATTTTCACTTATTATAAAACCAACCTCTTTTTATAAATTAAAAATTCATTCAATAAATAAATAAATAAATAAATAAATAACTTAAAATGAGAATATTTAATAAATATAAGAAAATTCAATTGCTAATTAATAAAGATTTTAATAAAAATAATTTATTAATTAATTATTAGTTTTCTAATAATTTTTTATCAAAACTCCAATTCACCATTAACAAACGTTGAATATATATA

>Cbs_2R-5_14A,15A

ATTCATTTAAAGATTTAAAACTCTAATAAATATAAATGATAAAAAATATAATTTATTGCTTTTAATTTATCTTGTTAGCATTTAATGGTTGGTAAAAAAACAACCAAAAAATGTATGATTATTTACCAAATAGATTTACTTGTTTGTTTAATAGTTAAAAAATATCTTTGAAACTATTTAATTAGTATTTTTATAAAATATAAACCAACCTCTAAATCAATTTTTTGCTCATAATTAATTAATTAATTAATTAAAAAAAATGATAATAATACTTTAATATTGTAAATTAATTATTATTTTAGTTCCAGTAAAGTATTATCGAAAAAAAGGATTTGTAAGCAGATAGAAAATAATTCAATTTTTTATGATTAACAAATGAGAAATTATTCATTTTTATGAAATTCATTCTAAGAAT

>Cbs_2R-6_cons

TTGAAAAAAAGAAGATTTAAAAAATCTATTAATCTTTAAGAATAAAAGTAAATTAATAAATGAGGTAAGAGTATTTATTTGAATTTAATTAAAACGATTAATTCTGCAATTTATTTAATTAATTATTTTATCATTTTAAGTGTATTGATTGTATTTAAAAATAAAAAGTAATTTTTTTTAAAGCTTTACAAAAATAAAAATAAACCAACCTCTTTAAACCATTTTAATATTAATTAATTAATCAAATATTTTTTAAAAATTCATAATTTAATATCAAAATTATTAAATATTTTTGAGAAAGTAAAATCTTTTAATTTTCAATAATATCTCATTAAGTATGTATTTATGTATGGATGGATGGATTTGATTTAGTTGTTGAGGTGTTTGTTTTGAAGGGATTGATTGATAAACTAAT

>Cbs_2R-7_13C

ATGTACTCTAATAAAAATTCATTTTTAAGAAATGTTTTTAATTAACAAGTACAATTATTTTATATAATAGTGTTTTTTTTAATATTTTTTTAATTATAATTTTAGATTAAAATACTTTTAAAAAGTAAAGAAAATATAATAACTAAATGAAGTCATAAATAATAAATAAAACAAATTTATTAAGCATATAAATTTATTTATAAACCAACCTCCTTTTGGTTTAATAAAAATGAAATTGCTACTAATTATTTGTTTGTTTGGCCAGGTTTGGCAGACTTTTTTTCATTTTGAGTTGCAAAAATTAAATTTAAATTTTTCGTAAAAATCTGATTTAGCAAAAAGGATTTAAATGAAGTAATTTGAGATTAAATGTGAAGATACCGATTTAAGGATTTAGAAATTAATCATAAATTTA

>Cbs_2R-8_cons

AATATTTGTCTCAATAAATGAAAAAATTAACTTAAAAAAAGATAATCTTATTATTAATTAGTTTAGCTATTTTATAGTTTTTCATTAATTAATTTATTTATTTATCTTTTGATAAATATTCAAAACTTACTTTTTATTTATATTTTTAAAAATTTTTGTGAATTTTTAATTTATTTTAATTGATAAATTTTTAATAAATATAAACCAACCTCTTTATTTTTGTTATCAATATATTATTTTTTATCAATTTAACTATTACATGGCATAAAGATAGATAAAATATATGACTTTAATTCTTTAAGAAAAATTTTAATTTAAAAAAAATTGACATATAAATGTTTAATTTTGTAAAATCTCTAATTTTTATATTGTTAATTTATAATATTAAATATTTTTTCCAAAATATTCAGTAATT

>Cbs_2R-9_1A,15A

ATATTATTTCAATTTTTAACAAAAAAATAGAGATTTTATTTTTTAGATAATTTAACAATAATTTAAATATTTGTAATCAAAAGAAGCTTATAAATATATAAGATCATCTTATTAAATATTATATGTTTAGCAATCATCGATATGAGAAATTTTATATAACTCTTTACTTTGATAATTTTAAAAACCAATTATAAAATAAAAAAACCAACCTCTTAATTATTTGTTTGTGTAAATAAAAATGTATGATATTTTAAATATTATCAAATTTAATAGTATTTTTTTTAAACTTTCCTAATTAAAATGAAATTTTAATGAATATAATTAGTTTCTAATTGATTATCAATTTTGTTTAATACTTTCAGAAATATTAATTTTTTTTTATTTCAAATTTTATAAAATACCTAAATTCTAAAAT

>Cbs_2R-10_1A

TATTTTTATTTTTAAAACTCTAATAATTAAGAAATTTTTTTAATTAATTTGGTAATAAATAATAAAATATTCAATTTTATTATTATTAATTAAATAGAAATTGATAATTATTATCAAACTAAATTAGCTTGAAAATTTCCAATCATTCAAATAAAATTTAAAAATTAAAAATCTATCTTTTTACTTCAGATTGATTCTAAAAAACCAACCTCTTTACTTATTTATTTATTTATTTATTAAAAAAGAAATTTATTATTTTTCAAGCTTTTACTTTTTGAGTTTGAAATATTTTGATTATTTTATATAGAGGAATGTTTTAAGTCAATTAACAATTTGGTTTATTAAAAAAAATTTTATATATCAAAATTGTAATTTTACTGCTTGACCATTAGTTTTAATGTGATTTTGAATAGTA

>Cbs_2R-11_cons

ATTTCATGTATTTGTAAATTAAAATCAAATTCAAATAACATCTGCATAGATTACTGATCTATCTAAAAACTGACTTATTTGGTTATGCTACAACTGTTGATAAATTATGTTTTTAATGCATCTTTATAAAATAAATTAATTGTTATTTTATCTCACTACATATTAATTAAGCATAGGATTTAATTTAATTAAAACATAAATAAACCAACCTCTTTTTGAATACAAATTTTGATATTTTATTTTAAGATATTTATGTATTAAACTCTTATATTAATTATTAATAATATGAAAATAAATTTAAAAAAATTAAAATAATAGCTGAAATAAAATTTTAGAATTTAAATGAAAATGGTTGATTTAATTATAATTTCAATATGATTTAATTGTATAATTTTAACACCAAAAATTAGAGTTT

>Cbs_3L-1_14A

TAATCTAAAAAGCAAGTATTATTTTTTAGATAAATTTGAATAATAATAAAAATATTAATTTAATTTGTTTTTTTTAATTCTAATTAGTTTGGACAATTAATTTTTTATATTTATATTTTCATAAAAATAAATAAATTTTAAATCTCATAAATAAAAATTAATTAATTTCAGCTAATTTTCTTGTTTATATAAAATGAAAATAAACCAACCTCTATTAAATTTATTTAAACATATCATGAATAATCTTTATTAGCTCAATTTTTTCAACTATTTTTGGAGTAAAAATTAATTTGTGTTTTGTCTGCTTTAGAAATATAAAATAATTAATAGAAAACAATGATAAAACTAAATATTTTTATATCTTTCAATCATTTTATTTCTTAGTTTTTTCAAATATTTTTTGAAAAACTCGTCA

>Cbs_3L-2_13A

ATTGATATAAAATAAGAATTATTTTACTTTTTCTTCAAATAGGTTTAGTAATCTAATAATATAAATATCAAAATTAAACATTTAAGCTATCAAATTTTATAAATCTAATCAATCATAATTTGATTAAGTGAAATCTTATGAAAAAAACAAACAAACTTTTTTCAATCTTTTAAATATTTTTTTTATTTTTAATAAATAAATAAACCAACCTCATTTTGGTATTTTATTTAATTAACAAAGTAAATATGATTTATTAATTTATTTATTTATTTATATTTGTTTTTATGATGCAAATATTTAGTTAATATCATAATTAAATTCATACAATCTAAAAAAAGTAAAATTAATAATTACTTTATTGATAGTTTTAATTAATGCTTAATTTGTTTTAAATTATATTTGATTTTAACAAAAG

>Cbs_3L-3_cons

TAAAATTGAACTTTTTAAAAAAATTATTAAATTTATTTTCAATTTATTTTGTCATTAATAAGTTTAATAAAAAAAGATGAAAGAAATACTTCTTCTTAAATGCTAAAATTAAAAGAAAAAATTAAAATAGTTCAAAATTTTTTATTTCAGCTTTTTGTCTGTTTGATAATAATATTGATTTAAATGCTTGGTAAATTTTTTAAACCAACCTCTTTTATGAAATTAAGTTGCACTAATTTTATAAAATTAATTCCTTATATTGATTTAAGAATAATTTAAATGTTGAAAAGAGTTTGAATTATTTATTTTATTTCTAATTAATTCGAAATTAGTCAAGAAATAATAGGAAAATAATAATTAAAAACATGAATTATTATATATTGGATAAAAAAATTAAAAAAAACATTTTGAAAAA

>Cbs_3L-4_cons

TTATATAAAAATGTTTTATGAATAAATTTTTATTATAAAAAAAATTTTAAATATTGTAAATTTATTTTATTTTTAAAGTCAGCCAATAAAAAGAGTTATTTAATTTCCCAAACTTTATTTTATTGATTAATTAACATTTAAGCTATTTTTGAGATTAAAATCAAATGAAATATTTATAAAAAAAGAAAAAAATTAAAAATTAAACCAACCTCTTTTTTATATTTCAAATAAATAAATCAAATTTGAGAGTTTTAATATTTATTTTATGATAAATAATTTTTTAACTAAAATATCATTTTGTTTGATAAAACTTGAAAATATTTCTTTTTTTAGTAATAAATTAATTAAACTTATTAATGAATCAAATAATTTTTATGTTAACATTTTAATTAATATTAAAAAGTAAAATATTTAA

>Cbs_3L-5_cons

TTTTATTTTATTCATGTTAAATATTATAAATACCAAATACGAATTATAAATCATGACTTTTAGATTTATTTATTCAATTTTTATAAATTTTTAAATAATTTAGATTTTATAATAATAAAAAGAAAAATTGAAAATAAAGTTTAGTGTAATTTTATTTTTAATTTTAATGAAAATAAATTTTTTATATTAAAAAATAAAATTAAACCAACCTCTTTAAGTATTTTAATTATAAAATAGAATTTAAATATGTTGAAAATTCATATATTTTGTATTAATAACCTAAATAAATAAATAAAAATTAATTCATGAATTAAAATTATAATTATTTTATTGCTAATTCAAATATGAATTTTCAGTAGAACAAAGCATTTAAAAATACATTTTTTTATACAATTTATTTTAAATTTGAGATAAT

>Cbs_3L-6_cons

TGATTACTGTATTCTTGCAATTTAATTTTAAAATGATTGAAATTAAATTATAATCGGATTAAATTATACTATACTAAATTAATATTGATGAAATTTTTTAACTATGAAAATAATTTTTTAATAATAATAAAATGATAATTTTTTATTTATAAGTAAAAATTTTTTTTGGTTTTTAAATACTTAAAACTTTTATAAATTAATAAACCAACCTCTTTTATTTTAGTTTGTGATTTATTAAATATATTTTTTATTTTTATTGAACTTTAATTTTTTATCAAAATTTAATTAAATAGCAAATAAATAATTTTCTGTGTATAATTTTTACTTAAATCTAAAAACGAATAAAAACATATAAAGTTAAGTTTTGAAAGAAAATAAATCAAAAATGTTTTTACATTAAGGGAGAGGAACAATT

>Cbs_3L-7_11C

AGAACAAAAGATTTTTGAGTTTTATTATCTATCATTCTTACTAATTTATTTCTTTTATAATTAAATTGATTAATTAATAAAATTAAATTGCAATCAAATTTTGTATTTTTTCATCTTCAATAATATTATTATTTAGTTTATGAAAAATTAAACATTTATCAAGATAATTTTATATTAGATTTATCAAATACACATTTTATTAAACCAACCCCTTTTTCTATTAATTCAACTTTAAAGTTAAATGTCAAATAACTATTTATATATGCTAATCAAAAAAAAGAAAGATTTTTAGAAATCAAATAAAAATAATTACTCACTAATGTTTAGCATAACATAGATTTTTCCATTTTAACATTTATTTTTTTCCTTCTTTAATTTGATATGTAAACAAACAAACAAATTTTTAAAAATATTT

>Cbs_3L-8_1A

CCTTATAATTTATCTTTGAATTACTAAACTTCATTTTTATTGAAAATATTTTTACTATTTATTTGATTTAAATCATAAAAATATGTCTATCTAAATAATTTCCTATTCAGTCAATAAAATGTAGAAATATAAATTAAAAAATCAAAACCAAACGTTTAAAATTTATTATATTTAATAAATATGAATTAACTTAAAAATAAAAAACCAACCTCTTTATCCTGACGGATTAAAATTTTAATATTTTAAATTATTATAAAATGAATTGTAAAAAAAAGTAAAATTTATTTGTTTTTTTATTAAACGCTTTATTTTATTAACTTTTAAAATAAAAAATATTTTGTTGGCTAAAATTTTTATTGTAGTATAGCAAATATACCATAAAAGCTAATTATTTTTAAAATAATCTTCAAAAAAC

>Cbs_3L-9_cons

TTTTTACTAAAATATGAACTACTTATTTTGTATTAAGGATTATAATTATTTTAAATTATCTAAATTTTTAATAAATTTTACTATTTAGTCCTTGTAGGATTTTCTATTTAAATTTTCTGATTAATTCTATTTATTTGGTTTTTACTTATATAATTACAAATATTTAGTTTATAATTTCAAATATTCTAAATTAATTTGATTAAACCAACCTCTTTAGAAAAAATTTTTCATTTATATTTTATTAAATTATTAAAAATATAAAAAATAAGTTTGTTTTTTAAGAATTTGCTAAATCAATCAATAAATATTAATATGTATTGCAAACTAATCAAACTTTACTAAAAGATAACTAATAAAATAGTTTAAATAAATAAATAAAATAAACTAAATTAATTAAAATGAATTTGATCAAATT

>Cbs_3L-10_1A,15A

TAGAGGCAACTTAAAACAAAGATATCATAAAGAAATCTCATTGCAAAAATAAATTAAGAAAAATAAATATTTCTAATAATAAAAAAATTTATTTTAAATATTTTAAATACATTTGTTGATTTTTCTTTTATTGTAAATTAATAAATTAAAAACTTAAATTGAATTAATTTACTAAATTATAAAAATAGTTTATCCAATAAAAAACCAACCTCTTAAATTAACATTGTTTATAAATAAAAATTTATTTATTTAGAAAAACAGTTAACTTAAACTAAAATTTAACTATTACTTTAATCAATTTTTATTAATTAATAATCCTTTTATTGAAATTTTGTGAGTTTTTGTTATAAATTAATTACTTTAATTAGTTATATGTTCAAATAGCTTAAGCATAGCTATTAATTCCTTCTTAAAC

>Cbs_3L-11_cons

TTGCTTCTACAATAGGAATAAATATTAATTTAATTAAGAAAGAATTATAAATACTTAATTATCTAATTAAAATAATCTTTTATCTAATATTTTAAGTATTAATCAAATAATTTAAAATTTATTTTTTTCTGTTTAGTTAAAGTATCATTTTTTAATTTAAAATTTTAATTTGTTTTTTCATATAATTAACTTTTAAAAGTTAAACCAACCTCTTTATAAATATAATTTACATTGTTAATAACTATCTTAAAAACTTTAAAATAATCTAAAAGAGATTATTATTTTTTCTTAATTAAAAATTGCGCTAAAAAATTTAATTAAAAAATTAAAAATTTAAAAATAAGTCTGACTTTATTTTCAATTTCTAAATTCTAACTTTACTAGTTTTGATTAATTACCATCAAGTAAAACATTA

>Cbs_3L-12_cons

GTATCAAATATTTGTATTAATTTTAATAGACTAAATGTTGTTAATCAAAAAAATTTAATTCAAAGATAACCATCAATTTGTTATAAAATCAAATTATTATATCCACAATTAAACATTCTCATTCTCATTCAAATTTACAAATTTATCTTATTCTTAACAAAATTTTTAAATAAAAAAATTTGAGAAATATCTAAATTGAATAAACCAACCTCTTTTACAAAATATATTTTAATAAATATAAACTGGTTTATCTAGCTCATTAATGTTTGTTTACCTTTCTAACTTAATTAATTTTTAGTAATATTTACAGGTTTAAATTCTTATTAAATAAAAGTAATTTTTAATTTTTATTAAAGAAAGCACTTCTAAATATTAAAACAAAACAAAAAATTATTTTTAAAGTTTAAACTTTTTA

>Cbs_3L-13_15A

TTATTATTTATCAAATTCCAATTTTAATTTATAATTAAAATGTATTTACTTTCAATTGATATTTAAATTTATTAAGTAATCTTTTACAAAATATTAAAAACTCATAATTATAAATAAGCTAAAAATTAACATAATAAACCTTAGTTAGTGTAGTTACTTTAAATGATTTTAATTTCAATAACTTTATTCTTTAAGAATATTAAACCAACCTCTTAAATTTATTCATCAATCATAAATAAATAAATATTTTAATTTTTATCAAAAGTAATTTAAAATTGTTGTTAATAAACATATTTTAAATTATCATCAAATATTTTTAAAAAAATTTTATGAAATAAATTAAAATTCCAAAAGAATTGATTTCATAAGGATAATATTTTGAATTTTTATTGTTTTTGGTAGAAAATAATGCAAA

>Cbs_3L-14_cons

AAATGAATTAAATAAATTTAAAAGAAGGCTATTGAATGTTAAATGAAAACAATTTTATTTTATATTTTTGTAGTTTTTCTTCTATTTTTTACAAAAAAAATCATAATAAATAATTAATCAATGCCTTTTTTTATAAGAAATTGTATTTAATTTAATTTGCAATTTATAAAATACTTGAAATAATTTATCTTCTCAAACTTTAAACCAACCTCTTTTATTTAAAGAAATGTAAAGTTATATTTAACTTATTTTAAAACAACTATCTTTAAGGAGAGGTAAGAAAATTTCTAGAAAATTAAAGAAAAGTATATGTATATTTTTTAAAATGCATAAAAAAATACTTTGAAAAAAACATAAATTCACTATTTTACAAATTTATTATAGATATTTTTATAAATTCATTGTAATGCTCACT

>Cbs_3L-15_cons

TTTAAAACTTAAATATTTTGTTTCTATAACTAGATATATTAATTGTATTTTTTAAAAACTATTTTCATTTAAAAAAAATAAATTATTTAAAATAAAATAAAATTAATTAATTAATTAAAAAGCTAAACATATTTTGTTCATAAAATCATTATTAAAATGTTAGAAAAAATAAATAAAAAAACTAAAAATTTTTATTTTTTTAAACCAACCTCTTTTATTAAATTTGAAAATTATTTTCTTAAATTAAACAAACTATTTATTGTTCATTTACCATTTTTTTTTTTTTGTGATTTATCTATCAAAAATTTAGAAAATAATATATTTTGACTAAAACAATACTTAATCAAACAAAGTGGTAAGCTCTTCTCAAAACAATTTTATTGATAGTATTTAAATCAAAAATCTATACTACAAA

>Cbs_3L-16_1A

GCGCAATTTTTAAAAAAATTAATATACAAAATACCCGTTAAATAAATTATTTTTATATTTCAGCTTTCTTAAAAATGACTACTATATTAATCAATAAAATAGATTAAAATATAAATAAAATTATTATGGAAAATATTATTATGTTTAATCATTTAATACTAAATAATCTTTCTTCATTTATTTTATTAATTTTTAATAAAAAAACCAACCTCTTTTAGATACTTTTAATAAATAAATATTGTCAGATTTATCTTGAAAAAAACAAAAAAATATATCTTATTTATAGATAACTTATGTTTGCTTATAGCTAAATATAAAAAAACTAAAAACATATTTTTAAAATAATTTATTGAAATAGAAAAAAAATTAATATACTTAATAAATTACTATAAAATATGATTTGCTTATTTTTGAA

>Cbs_3L-17

CTTTAGTGGGAAGTCAGATAAACAAAATGAAAATAATTTTTCTCAATTTTATGTAAGTTTTTTTATTCTTAACAGTTTTTATGATTTAATTTTTGCTTTTTGAAGTGTTATTATTGAATAATAATATTTTTCACATAAAGCATATTGTAAATGTTATAAAAATTTTAGGAGTTAATTAATATTTTATAAATATTAACAATTAAACCAACCTCTTAATTTTAAAAAATTAAAAACCTTATTATATAATATAAAAACTTATTATTTATAATTAATTATTTTTAAAGTTAATATCAAAATTTTTAATATAATTTAAAATTAATTATTTCTAAAGTTAATATCAAAACTTTTAATATAATTCATTATAAAATATTACTTAACTTTAAATAATAGTAAATAAATAGATTTTATTTTTTTA

>Cbs_3L-18_1A

TGTAAAATTTTTAAACTCTTTTATATTTTTATAGAACCCTCTCATTTTTAAAAAAATTGTGAAGCTCAAGGCTAAAGGTTTTACTGTAATCGTTTAAAATATCATCTCATTTTTTCTAAATTTAAATGTATTTTTATTTTTCAGATTTTAAAATAAAATCAAATAAATTAACAAGTTTTTTTTGAGATTTTAAACATTAAAAAACCAACCTCTTTTTGGTGAAATCTATTAAATATATTTTATATTTTTATTTAATTGATAAGAGTAACTTTTAAATGTAAAATATAAATTGATTATAAATTACAAAAATAACCTAGAAATTAATAAGCTTTTGATAATTCAAGATCTACCTTTTGTAGAAAATTCTAAAGTCTGATAATTAAATTAATTTATCAAATTAGTTGAACAAAAATTA

>Cbs_3L-19_cons

ATAAAATGAGATAGTTTTATATGAAATGTCTAATTTAATTTGGTGCTTCTGTTCAATTTTTGTCAATTCTTAGTTTTAAGAGTTAGATTAGGTTGCATTCATTATTTATTGAGAGCTTCTTTAGAATATCTAAGTATAGTTAAAAGCATTCATATTTATTTTAGTCTTAGATTCAAATAAACATAATATATAAATAAACTTAAACCAACCTCTTTAATAATTTAAATATAAAAATTATGAATATTTTTTTTAACTTGAAATAACAAAATAGTTTATTAAAAAAAAAATGATTTGCTTAAGTCTTATTTTAATATAAAAACATTAAAATATTTAAATTTAAATATATTAAGTTATCTATTTTAAATATTTAACATTTTTTCAGCTTGGTAAAAAATATAACTATATAAGAGGAAAG

>Cbs_3L-20_1A

AATGAGAGACTTATTATGAAAATAGAAGAAGTAAGATTTCCAAATATCTTCATAAAATTTAGTTTTAATATTTTAAATAATATTTGTTATTTTATTATAAAACTTAAATAATTTTTTAAGAATTTTATGTATTAATTATTTTTTTTTGTTTTGAAAAAATATCTTTTTAAATGGTTTCAACTATTTTTTTAAATTAAAATAAAACCAACCTCTTTTTTACCTGTTATTATATATTTCTATCTTATTAAATATTTATCAATTTATATTAATTGCTGAAATAATGTATAATTTTTAGAAGTGATTTGTTAATTATTTTAAAATATTTTTATCAATAATTTTAAGTTTTTTCATAAATTAAATCAAAAAATTTCAATTTAATTATTTTGCTCAATCTTTCTAAAATAAATGAGAAAAT

>Cbs_3L-21_cons

TAATTATTAATTTTAAAAATAAGAAAAATAAATTTTTTAAATATTTTAATGAAAGATAAAATTAACATTATCTTCTAAAAAAACATTTTAATTGGAATTTTTAATTTTAAAAGAAATATTTTTCCTAAATTATTTTAAATACTTAACTAAATATATATAGAATTTTAATCTATTAAATTTTTAACTTATTCTTGTTAAAATAAACCAACCTCTTTTAAACTTAGTTTATTTTTGTTTGGTTAAAAAATAAAATTATTACTAGATTTATAAAATATATGTATTTATTTAGTTAATACATTTAAATTTAAAAAATAAAATGACTTTTACTTAAATAGTAACTTAGTCTATCTTACTAGTTATATTTTTAGATTACTCTGAATAATATTAGATTTTTAAAAATATTAGTAACTTTGCT

>Cbs_3L-22_1A,13A

CTTCTAAAAAAAGAAGCAAATTTTATTTAACCCAGTAACTAGTATTAATTTAAAAAAATATTTGTATCATTTTTTATAAAAATAGTAATATTTAAAATCAAATAAAATTTTGAAGCTAATTTCAAAATTTATTTAAATATTTTAAAATCTACTCACTAATTTTCATTAATAAATTATTTAAAATTTAATTGTAATAAAAAAAAACCAACCTCATTTCTTAAATTTACTTTAATTTTAAGTTCAATAAATAAAATAAAAATGAATTAAAGCAAGTTATAATTTTGGAATATAGGGGTTCTTAAATAGTTAAAAACAGCTACTAAATCCTCTATTCGAACCCACCAATAAAATTTATTAATAAATAATTGAACAAGCAATAGAAAATTTGGGCTGTTATTTAAATATTTGATTCCAT

>Cbs_3L-23_cons

GTTTATTAAATTCTTTTAATGTTTTTTATTTCTATAATTAAAAATATTTTAGTTAATTAAAAAAATTGTACTCTATTACTAAAATATTATTTATTTAAAAAAGCATTTAAATCTTTTACTTTTGATAAATAATTTTGATTATTCTTCATAGTAGTTTTTAATTTATTGAATTTGTATTATTTATTTTTATCTATTTTATATAAACCAACCTCTTTTTATTTTACTGATAATGATGAATTTTAATTCATTTACATTATTTAATTTAAATAAATAAAAAATTAATTCTTTCAATACAATTAAAAAATTTTTAAGCTTTCAATCACAAAGATATATTTAATAAATATATACTTATTTATATCAATATATAAATTAAATTTTAGTAGTTTTGTATTTATTTTTTATCTTAAGAGAGTAT

>Cbs_3L-24_cons

GCCATAAATAAAAGATAATAATTTATTATAAAATAATGTAAATATTATTTAAAAGTATTTATTTTAACATATAAAATTGTAATTAATTTATAATTTTAATTAAAGCCAATTTATTAAGAATATCAATTAAATGAAAATTTTTAATATTTTTATTTAATTAAATCTAATGATACTTTTTTAATGGTTGCAATTTATTTTTGTAAACCAACCTCTTTTTAAAGTAATTAATTTGTTGTTTGTAAGAATAAATTAAAATATTTTATTTGTTTCTGATTACAAATAATAAGTATTAAATAAAGAAATTTGTTGTAAATGTTAAATTATTTTTTAATTTAAAATTTAAAAATTTAAGTTTGATTTTAATTTTAGATGAGAAATAGATAAAACAATATTCTTTATAACCAATAATTAATTG

>Cbs_3L-25_1A,11C

TATTTTTATTTTAAAGTTTAGCTTAATACTATTCATGCTTTAATTATAATTTTATTCTAGCTTGTTAAATTTATAAAAACTTTTAAAAAAATTTATTATTTATTTATTTTATTAGAAATTTTTTGAAAAGTAAATTTAAAAAATAAAAATATTTTAATTAACAAAGTTAAAGAATAAATTTAACTATTTTATAAATTAATAAAACCAACCCCTTTTTAATGTAAGTTGTTTTTATATTAATTAAAATATGGAGTTAAAAATAAAAGCTTAAGGAAAAAAAATGAGAAAAAAGGAAATTTATCAATAAATTATAGCCAATCAATATTTAAGATAATAATTTGACTGTAAAACTAACGATTTAATAAATCCACAAAAGATTTGATTATTAAATAACAAATTTAAGAAATATAGTGAT

>Cbs_3L-26_cons

TAATAAAATTTGATGTAAATGTTAAACATTTATTAATTTAAGTTTTATGCTTGTTATAAATCTATATATAAATTTTAATTAATAATTTTATTAAATTTTTACAAACTTTTGATTTTAAAACATGTTCATTTCTTTTTACATTTTATATAAAAATTTTAACAATTATTAATAAAACCTATTAAATTATTTAATTTAGTTAATAAACCAACCTCTTTTAAATTTTAATTGATTAATTTTGTTATTTTATATCGATTTCAATTTCAATTAAAATTAAAAAAAAGCAAACAAATTTGTTTTATTTAATAATATTTTTAATAAATTATAAAAATTCAAAAACTAATTTTCAATAAATAAAACTTAAATAAATTACTCAAATAAAGTTTATTTCTTGTTTATTTATATATATTTTAACATC

>Cbs_3L-27_14C

ATTAAAAATGAAATCTTTGAAAAATTATTTTTTTACTTTTCAGATTTGAAACATATTCTGAAATCATTTTTATTTGTTTAGATTAATTTCAGGAAAAATTACCATTAAACTAAATATTTATTTTGTTAATATAAGCTTATAACTTTTACAAAGGTAATAATTAATTTCCTTGAATTTACATAAATTAATTATACTTAAAATAAACCAACCTCTCTGTTTCTATCAATAAATATTAAAATCTTATTTTTTTTACTGATAAAAAACAGAGATTAGCAACCTAATAACTCAATTAGAACATTAGCTTTTGTAATTATTACATTTTTTAATGCTTGTTTTTAATTTATTTAAAAAAATTAGATTATATAATAAAAAATACAAGAAATAAAGGGTATTGAATAATAACATCTAATTTTAA

>Cbs_3L-28_cons

ATTATCAAAAACTCTATTAAATTCTATTTAACGAAGATAAAATTTGATTTTAACCATATAGTAATTCTATACATTTCGATAAAATATTATATAATACCAATAAATATGAGTTTATTTTTGAGAAATTTAGCTGTAAGAAATTTATTTTAAGTAAATTCAATATTTTAAAGTAATATTTTTATATTTTTTCTTTTATAATTTAAACCAACCTCTTTGTGTTTTGTGTGATGCAAGTAAAATAAATTCAATAAAATTTTTAATTAAATATTTATGAGATTAGTAAATAATGAATTAATTATTAGTTAGAGTTTTAGTATATATTTTTATTATTGGTTGTATTATTCATTCATTTATTTATTTATTCAAATAATTTTTTTAATATTATTAAATATTTGTTTATTATTTTATGGTGTAA

>Cbs_3L-29_cons

ATTTAATTTATATAAAATTGAACTTTTTAAAAAAATTATTAAATTTATTTTGTCATAAATAAGTTTAATAAAAAAAGATTAAAGAAATACTTCTTCTTAAATGCTAAAATTAAAAGAAAAAAATTAAAATAGTTCAAAATTTTTTTATTTCAGCTTGTTGTCTGTTTGGTAATATTGATTTAAATGCTTAGTAGATTTTTTAAACCAACCTCTTTTATGAAATTAAGTTGCACTAATTTTATAAAATTAATTCCTTATATTGATTTAAGAATAATTTAAATGTTGAAAAGAGTTTGAATTATTTATTTATATATATAATTAATTCGAAATTTGTCAAGAAATAATAGGAAAATAATAATTAAAAACATGAATTATTATATATTGGATAAAAAAATTAATGAGCAGTTTTAACAAT

>Cbs_3R-1_cons

TCTCTTTTTCAAATTTATTTTTTGTTGTTTGTTTTAACAATTAATTATTACTTTAAAAAAATTAACAAAAAATTTTATTTGATTGATACTTTTAATTTAAAATTAAGAATGAGATTTGAACTATAAAAAGTCTAGAAATAAAAAAAAATTGATTAAATTAATAATTATTGTTTCAGATGTTTGTTTATTATCTAAATTTTTAAACCAACCTCTTTCGACTTAGGAATTATTTATTTATTTAATTATTTATTTTATTTTTGAATTTTAATTTGATATCAACAGTGTTAAATTAAAGTCAAAACAAACACGAATAAAGTTTGAAATGTATATATAGTTTTTAAGAAGAGTCTGTATGAGAATATATACACAAAAAAATATTTACAATTCATAGATTTAATTTTAGTAAAAATAAATA

>Cbs_3R-2_cons

TTTTAAGTTTTAGTACTACTTAAATTATATCAAATGATACTGCCTTAATTTTTTTGATTTAAATCTGAATTATTGATTAAAAAAGAATAATCAATAGCAATATAAAGCAATATAACAAATATTTTTAATCTTCATAACTTTTAATGTATAACTCATATATTTTTATGAATTAAATTAAATTAATTTTATTTTTTCTAATCTAAACCAACCTCTTTTTAATATAAAAAATATTACAAAAAATCTTAATTTTATGATGAGAATATATTTTTAAAATAAATCTTTATATACATATGTACTTAATAACACAGAATATAAACCTCTTTCACAAAATAAGTGTAAAAATTAATAATAATAGAAATAAAAATGATATTATTATTCTTAATATTTATTTGATCTTGAATCATGGGTTTCAGAA

>Cbs_3R-3_cons

TGTTTAAATGTAAATACTTTTATATAATATAATATAAAATTTGTTATTTGTTTTATATTAAAAAATTAGTCAAATCAAAAACAATAAGAAAAAAATACTAAAAAGTAATTTACTAAACCTAAAAGGTTTTATTTAACAAAAATAAATAACAAAAAAAATTTATTTTTATTTGATAAATATATACCTTTAAGAATTAATTTTAAACCAACCTCTTTAAATAAATAAATTATTGTTTATTTGTTTAAAATATACAATAAAACAAATAAATTAAAAATTTACAAATTTGTCTATCATCTTTCAAATTAAAATCATCAAAAATTAAACAAACATAAAATAAGATTTAATTATTTTAATAATTGTATTAACAGATTCTTAATGGTAAAATAAATTAATAAATCAAAATAGAAAAAAACAG

>Cbs_3R-4_cons

TGTCTAAAAGGCTGAAAATCTTATAAAATCTTACTAGATAATTTAAAACTGCTATACTTAATTTCTATTTTAATTTCTTACTGGACAGCATATTACAATAGATAAATTTGTGTATAAATATGTATTCAATTATTTAAATTAATAAAAAAATCATCCTTAATATTACTAAAATATTTGTCACTTTTAACTAAATTTATATGTAAACCAACCTCTTTTAGTAGAATGGGATTCTTTCTTACAAATAAAATAATAGCAAAGGTATATTATAAAATTATAAATTTATTGAAGTTTTTAATAATCAATGTATATTTATTTTTAATTGAAAAGGAAATCTAACTCTAAAAATTATGATTTGTATTTTTCTTTCACTTAAAATAGTAAAAAGAATATTAAGCCATTAAAAATAGTAAATACA

>Cbs_3R-5_cons

TATTTGATTTGTATTACAAAAATTTCATCACTAATTATTTCTTATAAATTATTTTTTTTCAATACTTTAAAATAATTTTATGTTTCTTGACTAACCTGTTTAAAAGAAAAATTATAAAATATATCTGAATTGATAAAGAATTCTTTGTTTTTATTTTAGTTGATAATTACTTTTTTAATAAAGATGCCTTTTTATAAAAATAAACCAACCTCTTTTTAGAAAAAATAAATTTAATTTAAAATTATAAAGGAGAAATAAATTAAATTATCTTGAAAAATTTAATTTTTATTATTATTTTTTTTTAAATAAAAATATTTTACAAGTTTTTGTTAAAAAAAAAATAATAATTCTTTTTGTAGATTATTTATTTAATATTTAATTTGACTTAAAGTAGTGAAAATGAAATAAATTGAAA

>Cbs_3R-6_14A

TATCACAAATAGTTTTTTTGAAAATGTTAAGTGTTGAATTGTTTTGAATTTATTAAATTATTTTAAATTAGATTTAAATAATTAAATTAATTTTAGGAAGTTTGTAAATTTACAAATAAAAATTAAAATAACTAAAAAATAAATGAAATTTTACTTAAATTATTATAAAAATAAATAAAGCAAACTCTAAAATAATTAAGTAAACCAACCTCTATTTAATCATTTATTATTTTCATAAAAATTAATGAAAAGTAAAACAGTTAATTTGCATATTTTATTTTTTAGTATTTTCCTTAATCTAAAATTACTTTAACTTTTTGAAATAATATTTCAATAAATTAAATTTTTTTAAAAATTTTTTTTAACAAACAAATTTCAATATAAATACAAAATTAATCAATTAATTAGTTGTTAA

>Cbs_3R-7_1A

GTTTTTTTTATATTATTTCAATTAATATATTCATAGAACTTAAACTAATAAAGTCAAATTTACTTATATTTTGCAAAAAGACTAAATCATAAAAGTCAAATTTATTTTTCTCCAATAAATTATCCTTAACAATGAAACATAATAGACTCTTGAAAAGAATTGTTTAACTCAATATTTTATCTAATAAATAAAGAACTAAAAAAACCAACCTCTTTTAAAAAATTAATTTTTATAATGTCAAATAAACATTTTCAGCAAATGAATTTTTACTTACGAATTAATTTACTTTGTTTAATCAAAATGTTATTATTTTTTGTATTTTTTATATTTACATTAAAAAAAAAAATATGTATAATCAATTGAAAAAAAAAACTATGTAAATTCCATTTTTTTAATATTATTATATAAAATGAGG

>Cbs_3R-8_cons

TTGGGTAAGCTTTGTAATACTTTAAAAAATAATTTTTTCTACTTCAAAAAGTATTTATATAAATTATATTAAAATATTATTTTGTAGATTTTATAAGTGAGAATATAAATTATTTGATTGATTAAAATATTGCTAATATATTTTTAATAATTAAAACTATAATTTTAGTTTTATATAGAAAATTTAAATTTGTAATTATATAAACCAACCTCTTTTGTAAGAATAACTGTAAATTATAAAGATTAAATGCATTCCATCAAATCACTGTTCTTATTCAATATAAAATAAAAATATTTAGTATTCAAATTAATATTAACAATATATACAAAAATTTATTATTTTTTTCTTTCTCATATTTAACAATATCGTAATGATGATTTTTATTTTTTAAATAATACTAACTTACTGCTATGAG

>Cbs_3R-9_cons

TAATTAATTAGATATTATTAGCAATCTAAGCTTAAATTTAAAAACAACAAAAGCTAATTTATTTAATTAACATTTTTTTAATTTAATAGCTAAAAAAAAAAAAAATTTAAATAAATATCTAATAACTTAAAATAATTCCTTTTCAAAAAAATAAAAAATATTTTAAATTGATTATCAAATTTTTAAAAATCAAATAAATTTAAACCAACCTCTTTATATAATTGAAATGTTGCTAAGATTTTTAAATATAAATAAATAATTATTTATTCATTTCTGGATTTATAAATACAAAAAAATTTTTATTAAAAATATAAATTTAGAAATGATATTTTTAATTTTTTTTTTTTTTTAATATTTATTTAATAAAAAAAGATTTATTTAAAGTAGTCTATAAAAAATATATTACTATTCTTAT

>Cbs_3R-10_cons

AAAAAATACTGTTCTTACTTGATAAGTAAAAATCGTTTATTTTTGTTTAGAAAATTGTTTGTTAACACTTGTTAAAAACTTATATTAAAAATAAACTATTAATTTTAGTAAAATATCATCAACTAAATTCATAATTAAAATAAAATAAATTAATAAAAAAGAACAAATATTACTTTTTTTAATTTTTAAAAATTATCCCTTAAACCAACCTCTTTTTTGAAAAAATACTTTTATTATTTTTATTATTAAATTTTTTAGAGAATTATTTATGAAGAGCTAAGTTTTTTAAAAAATTTGTTTGTTGATCGGTATTTAATAGATAATAAATAAATTTTGACTTAAAATTTTTTTAAATATATAAATAAATAAAATAAGTGATAATAAATAAAATCGTTCAAATTAAAGCGCAAAATGT

>Cbs_3R-11_14A

ATAAAATTACATATTAAATTTTTATTTAATTTAAATTCTTTTTCAAGAAATAATATTTTTTGTATTATTTATTGTTTTATATATGTAAATTATTTTAAATTAAAAATTCTTATGTAGAATATTTAAAAAAAAGAAAATAACACTTGACTGATAAAAAACAAAATTTAATGTTTGTTAATTATAAGATTTGTTAATAAATATAAACCAACCTCTATTAATCCTAACATTAACTTGAAAAATCTAATTTTCATTTATTTAATTAATTTTTCTGCTTTAATTTGTTTAAATACATACTATTAAAAATACACTTTAATTTAATTTTAAGAAACTTAAGTTTCTTTAGAGTTTTATTCAGCTCGGGTAGCTCAGTTGGTTAGAGCGTGGGTCTAATAAACCCAAGGTCGAGGGTTCAAGC

>Cbs_3R-12_cons

TATCTTTTTCAAAGTAATTCTTTTATTAAACGTATATTATAATTTTTTGATAATAATATTTTCTCTTATTAACAAGCATATTCTTTTTTTATTCATTAGAAAAGTTTTTTTCTGATTATCAACTTGATCAGCTCATTAATTGATAAATATAATATTTTAAATTTAATATTACAATTTGCTGCATAAAATAATAATTAAATTAAACCAACCTCTTTAATCTTTTTAAATTTATTGATTATTTAAAATCTTAATAAAAAGCATTAATTAATTTAGCTGTATTTTATAAATACTAATTTCAATCTTTTTTTTTCTATAAGCAATAAATTAAAAATATGATGATTGCAGATAGTTAATTTTGAATGGTTAGTTAAACATAATTACTAAATTAAAGCTATATTTTTTTGAGATAAGGTTG

>Cbs_3R-13_1A

TTTTTATTTTAAATGATCAATTTAATTTTGATTTTTTTTAAAAAATTTGTTAATAAAAAAAATGTTAATTTAAATAAGTAAAATTCATTTTTTTATTTTACTAAAACTTTTTTAACTTGAAAAATAGAAAGCACTTTACTTCAAAATAAATTATTTATATTAATAAAAACAGCTTATTTTTTTTTATAAACTCAAAATAAAAAACCAACCTCTTTATTCTAAAACCTTTGAATATACCTTTTTATGAAAAAAAAAAACATAAAATTTAGAAATTAAATTGAAATAAACATTAAAAATACCCTTATATGTAACATAGATTTATCATAAAGTAACTAATTTTTTATTTATAAATAAAATATTTTTATAATAGCTTTAATTATTTAATTTTCAATTACTTACTCAAAAAATTAAAATA

>Cbs_3R-14_cons

TAAGTTTCAACCAATAAAATTATTTTAGTGATTAAAAAAAATATTTGTAAAACTAATAATTCAACACTTCTTTCTGCTTTTAAATAAGGCTTTATTGAATTTTTTGTTTGTTTAAAAAAATGATGTTTTATATTAAAAACTTTTTATGAATTTTGCTATTTTAATTGTCAAAGAAAAAAAATCTAAATTATTTTTAACAATAAACCAACCTCTTTCTATTTTAAAAAGCTAGTGATTATTAATAGTTTTTTTATAAATAATAAAATACTAACATATATATCTTTGAAATTAAATATTTTTTCATTTACCATTTTTTTAGATAAGAAATACTTTTTAAAGATAGAAAAAAAAATAAATATAAAATATCAATAATATCAAATAAAATTATTTGAAGTAATAAAAATTATGAATTTTT

>Cbs_3R-15_cons

GTAGTCAATAAAATTATTAAAGAATTTTGGTAATATACCCCTAGTTATCAAAAATTGGTCAACGCTTACAACATCTTATATTAAATATTTTAATAACCTAATTTCTTTTTGATTATTACTAAGTTATTTTGTTAAATTTAATTCTTCAATTTATTACTTAATTTAACTCTTGCCTAACCAAACATTCATTAATTAAAAATTAAACCAACCTCTTTTTTAAATATTAAATAAATTGAGTAATAAAATATTTACTTTTTATTTTTAAAATCTTAATAATTAAAGTGAAAAAACTATTCATAGTTTTTTTTTTTGAATAATAAACTAGCTTTAACTAACTAAATAAATTAAGAGATCTAATTAAAAATAAATACAGATATTATAAATTTTTATATATAAATCTACTTTTTTTGAATAG

>Cbs_4L-1_cons

TATTTAGTTTTATCTTAACTAATTCAATTAAACATTTTATTTGCTTATAATAAAACATATAAATTAAATAATTTCCAATGCAAATTCATACAAATATGAATTAGAGATATTTAAAATCTAAAAAAAAATGATATTAAATATTTGTTTTAAATATAACTAAATAAATAATTTTTTAACACTCAAAAAAAATACTTTGTAAATAAACCAACCTCTTTATTTATAATTATTTTTTTTATTACTAATTTTTTACTTTTATTTATCAAATCTAATTATTTTGGATTAAAATTAGTGAATTAATTATTTTCAAAGAATTGAACTTAATGGATATATTTTTAAAAACAATAAATCATTTTAAAGCTATTTATAAAATTTTAATTTTATCTTAACAAATAAAATAAATATAGTTAGTTTATAA

>Cbs_4L-2_cons

TTTAAAAATTAAATTTAATTCATAAATAAATATAAAAAAATAAAAAAAGATATTTTTATTTACTTTTAAAATCTAATAGCTATGTACTGTTTATCAGCAATAGCATTTTTTTAAATAGCAAAGTGTTATATTATTTTTTATATTATTTTGCAAATTATAAGCAAATCAAATATTTACAAAATAACAAAAAAATTAAAAATTAAACCAACCTCTTTAAAATACGTCAAGTAACCTAGTAAAATTGAGAGTTTGTATTTATTTTATGACAAATAATTCTTTAATTAAAATTGTTTTTATTTATAAAATTTTATGATCTTTCTTTTTTTTCTATTAGCTTTTTATTTAAAAAAATTAATTAAACTTTTTATGCTAAAATTTTGTTTAAATTTAAGTAAAATATTAAAAACGAAATATA

>Cbs_4L-3_cons

TAAGTATTATAAAGTTATATAAATATGCTTTTATGAAAACTTTTAAGAATAAATTAAATACTGTAAATTTATTTTGTTTTTAGAGTCAGCAAATATAGCAAGCAATCCAATTTTCTAGTTTAGATTTCATTTATTATTTTTGTTATTTTTCAAATCATAAGCAAATCAAATATTTATAAAAAAACAAAAAAACTAAAAATTAAACCAACCTCTTTAAAATATGTCAAGCTACCTAGTAAAATTGAGAGTTTTAATATTTCTTTTTTGAAAAATAATTCTTTAATTAAATTATTATTTTCATTAATAAAATTTTATGATCTTTTTTTTGTTCTATTAACTGTTTTATTTAAAAAATTAATTAAACTTATTAATAGGTCAAATAATTTTTATGCTAAAATTTTGTTTAAATTTTAAA

>Cbs_4L-4_13A

TTATTTAAAATTTATTTTGAAATAAATTAAATTTGTTATTTTGAAAGGAATTAATTTCGTTAATTAATAGATTTTTTTAGAAATAATAAACTTAGAATTTTTATGTAAAAATAACAAAATAAATAAATAAATTATCTAAAGCTTTTTATTTAAATTAAATATTTTAAAAAAATATTAATTGGATAAAGGATTGAGTTAATTAAACCAACCTCATTACAAAAAAAACAAAAATATACTAAATTTCATAATCTTTTTTGTTTAATTATTAAAATTTTAGTATTTATCATTGCTGTTAAAAACCTTACTTTAGAATACATGATATAATAAAGTGTTTTATGTTTTATAGATTTATTAAATTTTAATATCCTTTAATAATTTATTATTCAAGTTCATAATTTATATAATATTTGATATG

>Cbs_4L-5_cons

CATGTTTCTGCAGATCCAATCACACAATCTTTATCTACTCTATCCATTAGCAAAATATTATTTTTAATAATTATTCTAATTAGTATATTAATATTTTTTCTGATAGAAATTTATTTTTTAAATTATTTAAATTTTTCTTTATCTATTATTTAAAAACTAAATAATTTATAATAAAAACTTAATATGAAATGATAATTATTTAAACCAACCTCTTTATAAAATCAAATCAAAGATAGAATAAAATATTTTTTAATTCAATTTGCCATTTTAATTAAATAAACGAATACAAATTTTATTTATTTTTTTTTGTATTTAAAATAAACTCTCAATAACAAATATTGCTGGAAAACGATATTTTTATTTATTTCTTATTAATTAAACTGTTAGCTAATTTATATGACATTTATAAATAAAT

>Cbs_4L-6_cons

TATTTTGAATTTATTCTAACATACAGAGCATTCTAGTTGATTATGGATGATTGATAAAAATTTTCTTTTACAAAAATTGGCTTTATTGACAAAAAAAATACTATTTTAAGAAATTAGGAACTCATTAGATTAAAAAAATGGAGTTAATTAAATTTTTTCTTTTTTGTTAATAAGTTAAATCAATATTAATGAATTGCATATAAACCAACCTCTTTTTAAATATCGGTGATGTGTTCTGAAATAATTAAGATACAAATTTAAAAAGAATAAGTAGAATAAATTGTAAAAATATAACACTAGAATCGTGAATCATTTTAATAACTAGCTAGCTAATAAAAAGATATGATAAAATTATAATTTTTCCATCAAAAATAAATTTCATTTACTTTTTTTAACTTTTTAATAACCTTTAAAT

>Cbs_4L-7_cons

AAGAAATTAAGTAAAGGATTATTTTTCATATTGAAAATATTTTAATTTTTTGTATAGAATTATTTGGAAAATTTTTAAAGATATTCACTAATCTTTATCATTTGTTTATTAAAACAAATCATTAATATTTCTAAACTTCATATTCTGATAAAAAATTTTATTTTTGTTTTTGATTTAAAATATTATTTTGTGATTTATTTTAAACCAACCTCTTTTTTAATATAAAAAACTTTTTAATAGAATCTTAGAAAAAGTTAATGTATGTAGCAATTAAAATTACTTTTTATTTATTTATTTATTTATTAGTTAGAAATAATAAAAATTCTTTTTAAATGGTAATCAATCCTGAAAGTTCAAGAAAATAAAAAAGAAAAATAATTAAAATGAAATATAAGTAGGATAAAACCTTACTTAA

>Cbs_4L-8_15A

TTTGTTGTTTGAAAGTAAATCTTAAAATTTAGCCAAACTTGATCATTTTTTATATCCTTATTTATTCTTGTATTTCTTTTTTTTTTATTAATTTTTTGACGTATCTTTTTACAATTTAAAATTTTCCACCACAAATTAAAACTTAAAATTAAAATTCTAGAACAAGAAAGTTATTAAACATGAAATCTTTTAAAAAATTATAAACCAACCTCTTAAATTCTTAAAATCAAAATTTTCTAAATCTAAATTTGAATAAATAATTATAAAATAAAATTAATACATTTATTTAATCTTGAATTAATAATAAATTAAAAATATAATCAGTAAAAAGTATTGTGAATTTGATTTATTTTTGTTTTAATTATAATTTAATTACCGTCGAATACATAATCTGATAATTTTTAATTTTATTACT

>Cbs_4L-9_14A

ATATATTCAGAAGTTTAAGAATATTTGTCAAATAATTTGTTTCCCTTGATTTTTTTGAATAATAAATATCAAAATATTTAAAAAAATAATTTAATTAATGAAGTTTTTTAATTATAAAAAAATTTACTAATTTATAATTGCAGTTTTATTTAGATTGTTAATATTTTTAATAAATAAATAAATTATTTTTGATTTAGAAATAAACCAACCTCTATTGATAATAAACAATTATTTATAACCTTTTATTTTAATTCAAAGACTATTATAATTTATTTATTTTAATTTAATTTAAAAATTTAGATATTAAAAATGCTTAAAAAACTTAAGAATTAAAATTATAAAATAAAATTTTAGTTTAAAAAATAAATTACTCAAAGTTAAATGAATTAATTGATTACTCTTTTTTTCTCAATAT

>Cbs_4L-10_1A

ATGTAAAACAAACAATTTTGAATAACTAAGTTGTTAAAATGATATAATACTTCTTCTTCTTCTTCTTCTTAATTAAATTTTTAAGACAAACTAATTTTAGTCTCATTTTAAAAAAATAATATCAAAATATAAATCAAAACAAATTACTGTTGAAACTATTTTATTTAAATTAAAATTTTGGAATATGATATTAGTCACATAAAACCAACCTCTTTTATTGAAACACTTAAGAGTTAAAAATATTAATAAAAAATGATTTAACTGATTTAAAAAAATTTAATCATCCTATAATAATAAATCATAATGCATTAAGAAGTTATGGAAAAATACAAAAATATAGATTTATACCCTAAAATTTCACAAAATTCTTATCATAAAGTTGGTTAGATAAAATGAAGAGTTGTTAAGCTCTATC

>Cbs_4L-11_1A,14C

TAAAAATAAAAATGTGTAAACAACACACTATAGTTAATTACTACTAACTACTTTAAAAAAAATTAACTAACTTTTAAATTTGTTTAATTATGGAATGAAAAATAATATTAATTACTTTTTATCTTTTTTAAAAATATATTTTAACATATTAATTAATAATATGTAAGTAAGCATTTTATTTCAAATATTTTTTTCTAAAAAAAACCAACCTCTCTATGTAATTATTTTTATTATTATAAAAAGAATTTATTTTATAGTTTTTTAAGCCCATTATGAATTTGTTAGCAAATTCTGTAGATAAGAATTCAGAGATTAGAAGCAAAAAATTATTTTGAAAATCTAAAATTAAAAAACTTTGCTAAAAAATTCAATATCTTTAATTAATTTAAAATTAATAATATTATTAATCAAAATC

>Cbs_4L-12_cons

AAAAGACATTTAGATAAATTTTATATTCTATTAATAAAATAAAATCTTATTAAATTTTAAAAAAATATAAATATTTTATTTATCTTAATTTTACAAAATATTTAAGTTTTCAATAGAAAGAATAGTTGTTTAAACTATGTTCCTGTTTTCTTGATTGCAATAATTATCTTCAAAATAAAAAAATTTAAATAGAGATGATTTAAACCAACCTCTTTATTAATTTATCTTCTTTAAATTTAAAAATAAACCATTTAAATACTTTATGAAAAAGATTTTTTAATAATTAAATTTTAGTTATTAAAGTTATTTACAGAAAAAATCCAACTAAAGTAAATAATTATTGTTCACTAGAAAAAATAAAGTTATAATTTTGCTGCTTATTTTAAAAGAAATATTAATTTGTACTATAAATTAA

>Cbs_4L-13_1A

TATTTAGAAGAGAACAATTTTTAAAAGGCGTCTGAAATCTATCAACAATTAATAAAAATTAAAAAAAGAATGGTACCTTCTAAATGGATTCCTTTTATTGTAGAATCTTTCCAAAAGCAAAAGATCATTAAAAAAAAAATAATCAAACTGTACCATTTTTAATAATTTTTTGTTTGTTTAATTAATAAGAATTTAATAAAAAAACCAACCTCTTTTATAAATCTTACTTTAATTAATTAATTAATTAATACTATTTATTATTCACAATTATTTGAATGATAAATAGCTTAAAAATAAAAAATGAACATTTAAAAAGATTTACTTGGAGATAATTAGAATTATTTATTTCAATTTTTAATAATTTTTGATATCGTTTTAAAACTCAGAAAAATATTTTAATAAAAAACTTTGCAAT

>Cbs_4L-14_15A

AATAAAAATTATTGTCCATTTTTGGAGTTTTAAAGTTAATTAGGGTGCCCAATTTTTTTACAGATATATATTTGTTTAAGAAAAATAGTTTTATTGTTTTAAATATATGAAATATGTAATTTATCTATTTAGATATATAAATAAGATGATAAATAGGATAATTTTTAATTTATAAATTTTATATGATTAAAATTAAAAAATAAACCAACCTCTTAATAAATATAAAACTTTTAATTTTCTTTTATTTTTATTGAGTAATTTTGCTTTATTTTTTTTTTTTATTAATTCGAAGAGACTTTACTTTAAAAAACAATAAGCTGTTTCTTTTGTTATTCAAATATATTTAACATAAATTTATTATCTAAGTAAATTGATCTAATTTAAATGCATTAAAAACAATAAAAAGCTTTAACTA

>Cbs_4L-15_cons

CACAATAACTATTCTATATTTAATTTAATTTATAATAATTTTCTTTGCTAAAATAAATAAATAAGTTAATTTAATTTTCATTTCATTAATTGAAATTGATTAAAAGATATTTACGAATTAAAATATCAAAAAAAAAATTCAAAAAAAAGATTGTTGTAATTGAATAATTACTTACTTACTTACTTACCTTCTTTTATTATTAAACCAACCTCTTTTAGAAAAATCAAGCATTTTAAGTTATTTAATTTTAATCAAAAAGCAAATAAAAAATAATGAAGAAAGTTTAAATTAATTTCATAAAATAAACTTTGAATTGAAAAAAATTTTAAAAATAAAAAATAGAAAATGATATTTTATTTTTAGGTATTTCTTACCAACCACTTAATTAAAATTAGTAAAAATTAATTTAAATATT

>Cbs_4L-16_11C

ATGTTTTTCTTGAGTTTTTAATTGAATAAATAATATTTTAAAGAATATCTTACACATAAGAAATTCAGAACTTCCCTTTTTCAAATATCATGACTTTTATTAAATATTTATTTTATAAATTTATTTATGTAAAATAATATTGAGCTTTTAATAAAAAATTATCAAAATCTTATTATATTAATTAAAATTTAATAATTAATTAAACCAACCCCTTTATATTTAATAGAAAATTTTTTTTTATTGATTTTGATAATTTTTTTAATTTATTTTACAATTAACTTTTTAAAAATTTCTCAACTTTCTTTTTGAAAAAAATTATTAAATTATGATTTAAAATTCAAAAAATAATAATGATTAACTATTAAATTTCACTTTATAATTATTAGCAAAAATTATAAATAAATTTATTTAAAAA

>Cbs_4L-17_1A

ATTTAACAAATAGATAGATAGCTTCTATATATTTTGTTTCATTAGATTATAATTATTTTATGTAAATTGTTAACTTAATTTATTTTTGATCTGTTTACTAAGCTATTTTAAAGTTTAAAATTTAGATACTTAAAAAAGTTTATTTCATCAAAAATAATTAAATTTTTATAAAAATATTATAAATTTATGTTTTAAAGTAAAAAACCAACCTCTTTTATATAATGATAACTCTTAATTAATTAATAAATTAAATTAATTTAATAATTTACAAAAAAGTATTAATTTTATTTCGATAAATTAAGAAGCATTTCAAAAAAAATTCTTTATAATTAATTGAGCAATTTCTCTAATAAATTGAATTTATTTTTTTTTCTACTTTAAAGGAAAAATTCTCTTTTTTATTTATTTTCTTTTA

>Cbs_4R-1_15A

TAAAACCTAAGATACATACATACATAGACACACACAAATATGCTCTAAAAGGATATCTTTCTCACTAATAATTAAATAATTTTTTTAATACTAATTTATTCTAACATTAAAAAGTAAATAAAACTTTTTTCTATATCTTAATAAACAAATAAATTATCAATTATATTTATTATTTTTTTTTGTTAAATTATCAATCATTTTAAACCAACCTCTTAATTAATTTTAATTTATTTATGACTTTAAATAATAATAGTAAGAAAAACTAAATACCAAAAAAATTTAAATTTCTTCAATTAATATTTTTACTTATCATTATTTTATTCAAAATTACTTTTGTAAGGTTTATTGCTAAGTTCATTGTATTAATAAATCAGTCATTCAGTCAGTCTTAAATATAATAATTTTTATTATATAA

>Cbs_4R-2_14A

TTTACATTTCTAATAAATGTATCTAAAATTATATTTTAAATTCGAAATTCTTTTTAATTTATTTATGTAAGTAAATCTATCAAATATAACAACTATATATTTCTCTAAATATCAAAAAATAAATGTAAATAAATAAATAAACCAGCTCAAAATTCTAAAAAAATAAAAACTTAAACAAAAGCAAATTTTCAAAATAAAAATAAACCAACCTCTATTGAATTAAATAAATAAAAAAATTAAATAATTATTTTTAAAATTTATAAAATATTTACTACTTTATTTTAAAATAGATTTTAATGTTAATCAATCAATTAAAGTTAAATAAATTAGTTTTTACCGTTTTAGCTAATAAAAATAAAAGAAATTGAAAGTATATGAAATGCTTCTAATAAAGTTCTTAATTTTTAATAAAAGT

>Cbs_4R-3_1A

AATCTAACTTAAAGCTTATTCAGGAATTAAATTTTAATATTTACTTTCTTATTTTGAATAAACATATTCTAAAAGTTATTTTTTTTTTTAAGTAATAAAAATTTTAGTAAAAACTCAGAAAATAAATATTTGATAAAAATATTTAATTGTTTTATAGTAAAATATTATTTTTATATATATAAAAAATCGATTTGTTCTTTAAAACCAACCTCTTTTCATAGTTTTGAACTTAAATTTTAATAAATTTGATTAATAGGTTGATTGATTGATAAAAAATAATTATTAATTTCGTTTGTGTTTGTGGTGGTATTTCAAAACAAATAAATATATAATACAATTCAATATATTAAATACCCTTAATTTAGCATATGTAATTTTTATTTTTATAGAGCATAAGAATAAATGGATCACTAAA

>Cbs_4R-4_cons

TTAATCTAACTTATAGCTTATTCAGGAATAACATTTTAATATTTGCTTTCTTATTTTGAATAAACATATTTCAAAGTTTTTTTTTTTATTCAGTAATAAAAATTTTAGTAAAAACTCAGAAAATATCTGATAAAAATATTTATTTAATTGTTTTATAGTAAAATACGATTTTTATATTTATAAAAAATCAATTTGTTCTTTAAACCAACCTCTTTTCTTAGTTTTGAGCTTAAATTTTAATAAATTTTATTAAAAAATACAATTATTCAATTTTTCTAGAAATATTCACAATTTTAATTATGTAAATAAAAACTTAAGCTGATAATTAATAGATTGATTGATAAAAAATATTTAATAAATTTGTTGGTGAATAAGCAAAAATAGAAATAAATAGTAAGTAGATTATTTTTAAAGG

>Cbs_4R-5_cons

AATTAATTATTTTAATCTAACTAAAAGCTTAATGGATTTATTTTTAATATTTATTTATTTATTTACTTTGAATAAACATAGTCTAAAAGTTGGTTTTTTTAAGTAAAATTTTATAAATTCAGAAAATATCAAATAAAAATATTTAATTATTTTATAGTAAACTATGATTTTTAATATTTATAAATTATCGATTTGTTATTTAAACCAACCTCTTTGCTTAGTTTTAAAGTTAAATTTGATTAAAAAATACTATGAATTTTTATAGAAATATTCACAATTTTAATTATGTAAATAAAAACTTAAGCTGATAATTAATAGATTGATTGATAAAAAATATTAATTAAATTGGTTGGTGTATAAGCAAAAATAGAAATAAATGATAATTAGATTATTTTTAAAGGATAAAGTGTTTTAA

>Cbs_4R-6_cons

TTTTAATCTAACTAAAAGCTTATTATGGAATTAAATTTTAATATTTATTTATTTACTTTGAATAAACATAGTCTAAAAGTTTATTTTTTTTAAGAAACAAAAATAGTAATTTTATAAATTCAGAAAATATCAAATAAAAGTATTTAATTATTTTATAGTAAACTATGATTTTTATATTTATAAATTATCGATTTGTTTTTTAAACCAACCTCTTTGCTTAGTTTTAAAGTTAAATTTGATTTAAAAATTCTATGAATTTTTATAGAAATATAACAATTTTAATTATGTAAATAAAAACTTAAGCTTATAATTAATAGATTGATTGATATGAAATATTAATTAAATTGGTTGGTGTATAAGGAAAAAATAGAAATAAATGATAATTAAATTATTTTTAAAAGGATAAAATATTTTA

>Cbs_4R-7_cons

AACTAAAAGCTTATTATGGAATTAAATTTTAATATTTATTTATTTATTTACTTTGAATAAACATAGTCTAAAAGTTTGTTTTTTTAAGTAATAAAAATTTTAGTAATTTTATTAATTCAGAAAATATCAAATAAAAATATTTAATTATTATTTTATAGTAAACTATGATTTTTATATTTATAAATTATCGATTTGTTGTTTAAACCAACCTCTTTGCTTAGTTTTAAAGTTAAAATTGAAAATGAATTTTTATAGAAATATTCACAATTTTAATTATGTAAATAAAAACATAATCTAATAATTAATAGATGGTGCAAAAGCAAAAATAGAAATTAATGATAATTAGATTATTTTTAAAAGGATAAAGTATTTTAAAACAAATAAATATATTAAAAAAAGTAACTAAAATAATTAA

>Cbs_4R-8_cons

ATAAATTTCTTTGTATGTTAAAATGTAGTTTTTTATATTTGCTCGTTTTTTATAATTAATTATTTTTCTTTAAAAAAATCAATAAAAATTTTCATTTTTCTCTAAAAATAAAATGAAAAAAACTAAAAAAATTTTTTTAAAGCTTAAAGTGATATTTAACTTAAGAAAAGTTAAAAAAAATTTTATAAAATTAATTTAATTAAACCAACCTCTTTGTTTTGAAATTAAAAATTCAAATTTGTTAATATTTGCTTTATAAATTATTTCTTAGCTTATTAATTTCTCCTAAATTACATAAAATATATAAAAATATCAGAATTGTATGGATTCTTTTTAATTTTTTATTATGTAAATAAATTTATAAAAGGTTGTAAATCAAAGAAAAATAAATGAAATACAGTCTTTCTCAATCAGG

>Cbs_4R-9_11C,13A

TCTTCTTTTTAAATTTCATTTTGAAAATAGTTTCATAAATATTTGACAAAGATATTTTTTATTTTATTAAAATTTAAGCTAAAAAATTAATTATTCAGAATTTATTAGCAAATCTTTCAAATTATTTAATATATTTTATTTAAAAATAATTTCTCTTTAGTTTATTTCATTTTTATTTTATTAATTTAAAATATACCAAATAAACCAACCCCATTTTTTATATTTAAAATAATATATTTATTTTTAAAATTAATTAGAACTAAATTTTATAAATAAATTTTTAATATATATTAAATATACTCAAAAACAGAATTCGTATTTTAATAAATTTAATCATACTTTCAAAATTACTAAGTAAATATTTTGGGTAATAATTAATATATAATTATTCAATTAATGTTTTACAATACATTTT

>Cbs_4R-10_1A

ATGGATAAACTTAAAGAGAATCAGAAATCAACCTTTTTAATAATTTTTTGAAATTTTAATGAAGTCTATCTTAATGTATTAACAGTCAGAAATAAAAGTTTTAAATACTTTTAATAAATATTTCTGATATTTTAAGAATTTGTTTTAGAGATATTCTCTTAATTTTTAAAATTAGAAAGTTCTAGTAAGCTAAATAATAAAAAACCAACCTCTTTTTTATTTTTATAATATTTTCATAAGTATCCATAAATAAAAATTTTTTTTTTTTTTAAATTGAAATAATTTTTTTTTTTGAATAACAAATTACTGAAAATCCTAATAGTAAAAATTATTTTAATAGAAAAATGAAGCTTATTATTTGAAATTTTGCCATATTGAAAATATATTTTTTAATATTCCCAAATATAATTAGGGA

>Cbs_4R-11_1A,15A

AATATAAATAATAAAATAAAATTTTTAATTGATTATATAAATTAAGAGATAAAAATAAATATTAGACATGATTTATTATTTAAAATAATCAAACAAAAAACTACAAACTAAATTGCAAATATAACCTATTGTTAATAGATATTAAACTCTAAACATAAAAATATTATAAAAATTAAATCAAATCTTAAATAAAATAAAAAAAAACCAACCTCTTAACAAATTTAATTAAAAAGCATTTAAGTATATCTAAAAATATATTGATTTTTTTGTATTAAAAAATATAATTTATTATCTTTTCAATTTTTTTGAATTCATTAATTCAGTTAGTTGGTATATTTTAAAAGCTTACTATTAGCTTTCTAGAAATTATGTATCTAAAATAAAATAATAAAAAAATAAAAATTTCTTTTAGATA

>Cbs_4R-12_11C

TCGTTGTATCTATTGAATTGCTTGATTATTAATCATGAATTTAACATTATCAATTTTTCATTTTAAAAATTTTGTTATAAATTATTAATTGAACTTAATATCAAGCTTTTATCAAATTACATAAAATGGATTTTAATTTTAAATTCAATTTATTTATTATTTGACATGGATAATTTTTATTTAAATTTTTACAGTTAAATTAAACCAACCCCTTTTAAAAATGCTAAGATAAAATAAAATTAATTAGTTTCATCAATTGCTTATAAATTCTTAATAAAAGAAATTTAAAATAAACTATGTATGTATGTTATTGAATTAATAAAAGAATATCCTTAAGATTTCATTTTTTTATAGATATCAAGTACTACTTAAATTGGTTTGAAAAGAAATTTCTTTGTTCTAAAAGTTTAAAATA

>Cbs_4R-13_1A,14C

GATAAAAATGAAAGACATTTTATGCAATTAATTTAGTTTATAAGATAAAAAGTAATATAGTGAGTGATCAATATTAGAGTGATAAAAGAATTAAAATAAAAATATAACTTTTTTATAACATATTTATTATTGAACTTTGGATATATAAAAAAAGCTAGTAAAATAAATTTCTTTAAATTATTATTTATTTAAATTTTAAAAAAACCAACCTCTCTTTCTTTATATTATTCTTTACATAAAAAAATAATTGAAAAAATTTAAAAATAATTGCTTCATAATAAATTTATTTTTCAAATCTTAAAATTTAGAAAATGTATCTATTTAGTTAATATTTTACTTATCAGTTATTTATGATTTAAATTTTATTTCACTTTGTAAATTAGATTTTTATTTAAATCCATTGTAATTTTTTAAT

>Cbs_4R-14_11C,13G

TTAATTTTACTTTTTACAATATTTAATTTTTGAAAATTGCTTTTTTAATTTTAATTAAAATAACATTAATAAAAAATAATTTAATCAAACTATTTTTTAATTTATTATAATTATGTTTCTTCCCTAATTATCAATAATATTTTTTTGAAACAAAAACTATCAGTTTAATATATTTATTAATTATTATAAAATATTTAAAATAAACCAACCCCGTTTGTATTATCAATTAATTATTTTATTATTGATTTTTAAATGAAGTAGAAAGTAATTTAAAAATAAAAATTTTTAGATATTAGATAGATACATAAGACATACATACATTTTTTCTATTTTAAAGAATTATTTTAAAAATTTATTAGATTTACATTTTTGCGAAATTCAAGTATAATATAAATTTATCATAAAAATTGATAAA

>Cbs_4R-15_11C,15A

TTTCTTAAATATGTAAAAACTCACTTAAATTCTTTATTTTTAAAGAAAGCAATACCAGTTATTTCAAATTTTAAAAGGTATATTAAAATTTTTTATAACAAATTGATAAATAATTTATCTAAAAAATTTATTAAATGGTTTGTTATTAAGTTTTAAAAATATTTATTAATTTATGTTTGGTTGATGAATTAATTAATTAATAAACCAACCCCTTAATTTCATTGGTTTCTGTTTTTATTTACATTATTTTTTATTTACAATAAAACTTAGAAAAATTTGTATTCATAATTAAAGATAGTTTAAAAGAATTAAATATGATTAAATTTAAATTTCATAAATTATTTAATTAATTCATATATGAAATAGCAAATAACACTTTAAATATAGTTGAAAGTTTTGAAAATACTTTTGAAAT

>Cbs_4R-16_1A

GACTTAACCAATCAATAAATTAATAAATTGATATTGAATATTTTAAAGAATTTAGATTTTAATTTATAAAAAACTGATCAATATCATTTAATGTTAATAAGAGTAAAAAGCATTTGGAAAAATTAAAAATATACAAAAAATAATCAAATTAAACTATTTTAGAAAATTAAAATTAATCTAATTTATTTAAAGCTACTAAAAAAACCAACCTCTTTCTTTAACTAAAAATTCAGAAAAAGAAATATCAAAAAAATTAAATATCTAACTATTAAAAAGGCAATTTCTAGCTTTAATTAAACTTATATTTTTTTTAATTTAATTATTTTAATAAATAATTTTAGAAACCTCAAAATTAATTAATTTTTTCTGAACATCATTTTTTATAACAATTTACAAGATTTTTTTATTTTAAAAT

>Cbs_4R-17_1A

GTTTTTTTTTACATCAGCATTAAATACATAAATTAAATCATTTTTTAAATCCTAATTTGATTAAAATTAAAAAATTGAGTTTTTTTTATTAAGTAAGAAATCACTCAAAAAATCACATTTCTAAAAAAATTAATTTTATTTTTATTATTTAAAAGTTAAAGAATAAATTAAAAATAATTTAAATAAAATATAAAATTACAAAAACCAACCTCTTTATTTATCTTAAATACCCAAATATTATATTAAATGATTTATTTTAATTCTTTCTTAAAAAATTACATTTTCATTTAATTTTTATCCAAAATCATTGTAAAATTCTAATATTTAATTATTTGCGTTTTAAAAAAATATTTTTAAAGATAAATTAAGAATGTTTCATTTATAATAATTAAAAAAAATAATCTAAATAAATGAT

>Cbs_4R-18_cons

ATTAATTCTAAGGATTTACAATTTTGATGAGATTTTTGATAAAATTAAATCAAAAAATAGTGTTTTTTTTAATTCTTTTTTTTCAAACAGAAATATTTTAAATATTTAAGAAATGCTAATTATAATCTTTTTTTGAAGTAGAAATATTCTTTGTTTCTTTTAAATTTAGAAAACTTAAATATTTTTAACAAATCATGTTCTAAACCAACCTCTTTTAAATTAAAAAATGATACAATTTTATTAATTTTTAATATATGCTTTTTTGAATTTTGATATTTTTTGAAAATTGAATTAGCTAGTTAATAAAAAAGGAGCAGTAATAAATGAATCTTTTGGTATTTTATTTTTGAATTTATTATGAAATATATTTAAAATAAATCATATCAATTAAATCAAATCAATAAAATCTTGATTG

>Cbs_4R-19_1A

ATAAAAGAATAATTTTCATATTTAATTTCATTTTTACAAAAAAATATAAAAAAACAGACATTTCTTTTTAAATAAATTTATTAACAACAAATTAATAATTAAAAAACTTCTTTTTTTAATATAATTCATTAGATCCTTTTAAATATTACTTAATATGATAATTATCACTTAATTGTTTAAAAATGATAGAATAATTCTAAAAAACCAACCTCTTTTAAAAATGTAAATTGATAATTTATATTTTTATATTTTATCACATTAAAAAAATTTTTTAAAAAGAATCCTTCAAAGCTTTAATTAAATTTTTGTAATGTTAAATTTTGTAAACGTTTTAAATAAATAAATAATTGATAATATTTACTACATACATTAAAAGTAATAAAAAACAAATAAAAAAAATTAATAAAAAAGTAAC

>Cbs_4R-20_14C

TTTAAGCTTAATTAGAATAATAACTATTATTAGAGTTGTTTTTTGTTATATTTTTATTTGATAAAGAATGTTAATTAATTTTTATGATTTTTGTCTTATTTCAATAATATTGTTTATAATAAAATTATTCATATTCTTAAAGAAAATAACTTGGAATTAAATTAAATAAATAATTTATTTTGTATTATTATCTTAAAATATAAACCAACCTCTCTTATTCAACAATAAAAGATGAATAAAATTAATAGACACTTTTCTTATTTGTTAGAAAAAGAAAAATAATTTAATCAATTTTTTCCAAGAAAAACAATTCATTTCTTTAATATAAAATTCTTTTTTTTTTAATTAATTCGAATTCACTTAAGTGTTAATAAGGATTCTAATGTTATGTTAATTCATAAATAAATGTCTTGAA

>Cbs_4R-21_1A

TCAATCTATTTTTAAGCATTTCTAATTTTAATAAATTATTCAGTAAATTTAATTTAAATTAAGATTTAGATTTTTCAAATAATTTAGTAGACTATTGATAATTTTTTTTTAAATAAAGAAGTAAAAAATTAAATTATAAAATTCTTTTTAAGTTAAATTATTTGAATAAGTTTTTAAATATATTTTTTATTTTAAAATTAAAAACCAACCTCTTTTTTATTTTATACTTAAAAAAATCATTTTTACTAATAAAAGCAAAGCTGATTCAGTTGAAAACAAAAATTATAATTTTACTAAAATTATCACAGTTAAAATTAAATTGTCTTTGTACAAAGATATAATCAATAAAAAAATCTAAAGTATTCATAGTTAGTTAGTAAATAAATAAGATAAATTTTATATAAATTAATGACAT

>Cbs_4R-22_cons

GGATTCTTTTTATATGAAGGAGAAGAATAAAAATTGTTTTTATGAGATTAAATGTTACAAAAAATTTATATTTATTAATACATTTCAAAAAATAAATTTTTATTTTTGATTGGTTTTATTTAAAATGTTATTTTAAAATTAATTTATTTGCTTTTAATTTGATAATTTACAAAATAAATTGTTTAATATCAAATATAATTTAAACCAACCTCTTTTTTTAAATAAAAAATAATTACCAAATATTAAATTGATTGCTATTTAAATTAAATTAAATTAGTTTTTTCAATTATTAATAAAAATTTTTTTAATTATAAAAAATATTCTTAACTGTTATGAAAAAGTCACCACAAATGATTTAAAAAATTGGTGATTGAATTTTTTAATTGATGAATAGATTTTTAATAAGAAAATAATG

>Cbs_4R-23_cons

CAACAAGTTTATAGCTGATTTCATAAAAAATTTATTTATTTTTAATAATTTGTTTTAAAATTGAATAATAAATAGAAGCGTATTATAGTTAGCTTTTATTAAAAAAGTTTATCATTTTCTTTCAAAAAGTATTTATTTGCATTTTCCTTATATTATTTTTTAATTATATTGATTATATAATAGTAATTTAAAAAATTTGATAAACCAACCTCTTTTAATTAAAATTTATTTTTTAAGTTAACTTAAAACACTTTTTACTTAATAAATTTGATAGAAAAAATTTATTTAAAATTTGTTCACATAAAAATTTTTTATTTGTTTAATTTTGTTAAAAATGAATGATCTTATTTTTTATTTTGTCTTATAAATACATTAAATACAAAAATATATACGTTCATTCTTCTAATAAATATTT

>Cbs_4R-24_13A

ATTCATTAAAATGACTTGTAATAATTTATGAGTATTTTTATATAAACTTTTCAAAATATAATAGAATTTAAACAGTTTAGCATCTTAATCATAATCATTCATAAACAATCTGAAATATAAAAGTATTACTAATAAAAAAATAACTTTAATAAAATATGAATAAAAAATAAGATTTTTGGGTTTTAACTTTTTTAAAAAATTAAACCAACCTCATTATTTCATAATAATTTTTATTTTTATTTATTTTGTATTAATAATATTGAAAATAATTGAAATTTTATAAAAATTTATAATTAATAAGTTTAATTATTGCATTTCATATGTTGTAAGATAATTAGGGTGTGTCTTCATCGAAAAAAACTAGCCTTTTTTTTAAAATAAGTAAGTCTCTTAAGCTATTTAAAGCATAAGAGAA

>Cbs_4R-25_13A

TTATGAATTTTTACCCAAATTTTTTTTTCAGTAAAGATTTCTCAATTCAAAGTAAATTTCGAAGATAAGTACTGAAGACCTTAAAACTAACTACAAATAGCAAAAAAATAACCTTCAGCACTATTTTATTGAATTTATCTTCGAAAATGACTTAAAATATTAATAAATAAACTTCTTTTATATAAAATTAATTTTTGAAATAAACCAACCTCATTTTGAAAATTAAATAGAAATTTTTTAAACATCTATTGAAAAATAAAATTTAAAAATTTTAATTTGTTTGCTGGATATTAAAGTTGTAATGATAAATTTAAATGTTTATTCTTTTTATTCTAATTAAAATAATTTTTATTTATTTATAAATTTACTTTTTAAATTAATTTTTTATAAAAATTAAATAGAGAAATATCCATAT

>Cbs_4R-26_cons

TAAAATTATTTATTTTTTCACCAACTTTTGAAAATTTTGACTAATCTCTTTTGTTGTAAAATAAATGTTTAATAATATATGTTGAAAAAAGCATAAAAAATTTTGTTATTTAAATTGAGAATAAAATAAAATCAAAGCATTTATCTTAAATATTATTTAATCTTATTTTATTTTAATTTTTTAGCTTTCAAATTTTAAAATAAACCAACCTCTTTTTATTTATTATCAAAAAACCAAAAAATTAAAAAAAAATATGATTTTTTATACAATAATAAAAAGTTAAGAATGTTAGAATTATTAATATATTTATAAGCTATTTAATATTTTTGCTGGATTGTATTTTAAATTATATTATTTAACAGGTCTTTAATGTTAACTTAAAATTAAAATTAAATTTTTAGTCATTTTAACAAAT

>Cbs_4R-27_cons

TTTTACCAAGCTTAAATTTTTTAAATTTATTATTCATTTTTTTAATAATTTTACAAGAATTATTTTTTAATATTTTTCTCAAATAAAAAATTTGCTTTGATTAATTTTATTTTATTTTTTTATTTTTAAATATCTTTAATTTTTATTTAAAAAAGTTAATTATTTATTATACTTATTATATATGCCATATAAATACTAAATAAACCAACCTCTTTTATATCTTTTTAATTTGATGAAATTATCATTTAAAGTTTAATTTATTTGAAATTTTAAGCAAGAATCTTTAATCTAAAACATAAAAAAGTATTTAAATCAATAATTTCATTAAAATAAAATCAAGCTTAAATTTTAATTTATTTAATATTTTTTTCATTTTATTTTTTCATAACCAATTTAAAAAAAGAATTTTATATTT

>Cbs_4R-28_cons

ATATTTTATCTCTTAAGAATTTTCGAGATCTTATTTTAGTTTTATGCAAAAATTTGTAAATTTTTATTCATTCTATTTTCTGGTTTATATTTTAGAACCTCCAAAGCCAATGTTTTTATGGAAATTTCAGTTTATAAATATATATCAAATATCATTATTATAAGAAAAATAATTCTATTTAATAGAATAAAAATTCATTTTAAACCAACCTCTTTAAATTTTTTCATTATAATTTTTATTGAATAAATTTTTTTTGTTAATTATTTATCTTATAATTTAAAAGTTATTGTTTTAAATTCACAAAACTCAAAAAAAAAGCAAATAACAAATTTAAATAAACAAAAAAAGATAAATTTTATTTATAAAAGTATTAGTTTTTCATCAGAAATAACAATTAATTTTAATCAAAAATTAG

>Cbs_4R-29_cons

TAAATAGAATTTATACAAATAAATGTAAACAAATTTAGAAAAATAAATTTAACAATTAATTTTTTATATTTTATACATAACTTTAGAAATCTAGATTTTAGTTCTATTTTTTTTAACTTAAGTATACATAAATATTTATCTTATAAATTAAATTTATTATTCAAGTAAATTATTTAGTTTTTAATTAAAAATTTATAAAATAAACCAACCTCTTTACTTTTAATCTCTAAAATTTAACTGCCAATTAATTTTTTAAATTAATTCATATCTTTAATTTTTTGATAATATTGTCAATTTAGAAAATTTAAACTCATTTGTTATTAATCCTTAATTTATTGAAAAAATTACTATCTTCATTTTTAACTTTAAGACTTTTAAATAGAATGAAATTTTATGTTTATGTTTTATTTGTTTT

>Cbs_4R-30_1A,15A

TTTACTTTAATGAGTATTTTATAAAGTCATTTTAAAACCTATCTTAAATATAATTAAAATTTTAGATAATTTAAAAAAAGCTTAAAGCATTCAATGTAAATTCAATAATTGATCAAAATTTGACTTATTATTATTCTAAATGCATTCAATATTTGAAAATTAATTAGAATAAATTTCGTTTTTATTTGATTAATCAAAAAAAAACCAACCTCTTAAAAGCTATCAATAAATATTTTTTTTTTGAAATTAATTTACCAAAAATTATTGTCAAAGAAGAATTAATTGTTTATTTATTTTATTTCTAAAATGATTATATGCTATTTAAAGAATAAAAAACTTTTACTTTTTAAAAAATACATTTAACTTAACTGTTTATGCTTTATATTTCATTATGGTTTTAGTGATTATTTTTCTG

>Cbs_4R-31_1A,15A

TATTTATTTATTATGCCATATTTATTTAATATTTAATTAGACAATTTTTAAATATTTTTGCATTTTTTGCAAATTTTGTAAAATGCACAATTTTATTACTTTTTATTGTGTAAAGAATTATAATAAAGCATAATAAAAATAATGTTTAAATTTATTTACCCCATCTTGCCCCCGCAGACTCTTTAACATGACTTCTAAAAAAAACCAACCTCTTAAAATTAGGATCACAAATAATGAAATATTATTTATAATTTATTTATTTAATTATAAAAAATAAGTTAAATTATATAATTTATAAAATAAACTTTTATTTTTTATAATTTTAGTTTTAAATGTTATTTTATATGATTTTAAATATTTAATAAAAAAAATTTGAAAATTTGAAGCATTATTATAAATGAATCAAAGAGAAAAT

>Cbs_4R-32

TCTTTAGTACTTTTAAATAATATTTTATTTAATTAAAAAAGAAAAATTCATAAATATCTATTAAATTCTTAATATATAACAAAAGAATAATAAATACGATATGATTAGCTCTAATTAAAATTAAATATATGAATTAAAAATAAATTATAAAATTGTACATAAATTTATAAAACTAATTATTTATAGTATTTTTTAAATTGTAAACCAACCTCTTTTTTTATTTTTAATATTATTCTTTAAATTAAAGTTGTTCATTTTTAAAAAACTATTACTTTTAATTAATACAATCTTTATTTTTTCTTGAAGTTTCTAATAAAAATTTAAATTCTTATAATGTAATGTTTTTAAAATTAATGGTTAACCACTGAATTATTTTCTGCAAAATTCAAAAATTTATAAATTTTGAAAGAAAAAA

>Cbs_4R-33_cons

AAACTAACTCATTAAATCAAACTTTTGAATAAATTATTAATTCTAATTTTTGTAAGAATATCTTGAAAAACAGTATCTATCTATCTATCTATCTATCTATCTATCTATCTATCTATCTATCTATCTATCTATCTATCTATCTATCTATGAATGGCTCGCTAGCTTGCACCACCTACCAATTAAAAATAATTTAATTTTTATAAACCAACCTCTTTTTTTTTTCAATATTTAATTTATATATTAAATTAAGTTTATTGACTATATTAAAACTTTAAATTTATTTTTTTTTACTTTATATAATTTTTTAAATAAAAAAATAAAAAAATTTCTAATGATTTTTAAAAGTAAGTAAGCATTTATTTATTTATTCTGCCTTATTTTTGTTTTTGCTTTAACATATAAATTATGATACTAG

>Cbs_4R-34_1A,13A

ATTAAATAGTTCAATTTAATTAAAATAAAAGAATTCTTGAAAAATCGTAAGAAATTTGATAAAAGTTTTTAAGTTTTTTGTTATTTTAATTGAATAAAAATGTATTTTTTATCAGCAAATTAAATCTTTTATGGAAAATATATCTATTTTTTTAATAAATATCAAATTAAATTTAATTAAAACTTTTCTTTTTTACAAAAAAAACCAACCTCATTAAATAAGTTTGTTTTTAAAATATATATTTAAATAGTAAAAATGAGAATAAGGCTTGTATTTAAATAATTTAATTAATATTTTTAAATAATTTGTTTGGTTTTATAGGAAATTTAAATAAAATAATTATCTTATTTAATATAAAATAAATTAAATCATAATGCAAATTTAAATTTAAGAAAAATATTTCTGTTTAAAAATT

>Cbs_4R-35_1A

TGTAACAAAATAGCATATTAAAAAGCCCATTTGTTTTAAATAACATAAATCAATATATAAAATACAGTTTAGCTTTAAAAATAATAATAAATACATTTTAAATTAAAATCATTTTTATAAAAAAGTGAAAAGAAAAAATTAAGCAAAATAAATGTACATTATATATTATTTAAAGTAAAATATATTAAATTTCTTTTTAAAAAACCAACCTCTTTGAAATTCAATGTTTTATACTCTTCGAATAAATAGTTTATTTGTTTGTATACAATATTTATTAAGATTTTTTAAGAATTTAAATTTCTATCAAAAATTTCCCTAGATATAAAAATGCAAAAATATAAATTTGCAGAAGAAATCAATAAAAATTTCAAAAACCAACATTGGTTTGTTTATTGATTTAAAAGCTAAAAATTAA

>Cbs_4R-36_11C,14C

ATTAAACTAAAATAGTAGAAAATTCTATTCAAATTATATTTATGGAATTGTAAAACAAAATCCATTTAAATATAAAATTAATAAAAAAATGAAATCAAATAAATGATGCAAAAAACAGAAAAAAGATATCAAGAAATATCTCAATAAATAAATCTTGTTTTTAAAAAAATGTAAATAATGAAATAAATTAATTTAAAAAATAAACCAACCCCTCTTTTATTTGATTAATTTTAATTTATTTTGAGAATATTTTGATTTATTTTGATTTAGAATTAATTTTTAAAAAAGATTTACAAGCAAATATAATTTATAAATACATAAAATACAAAAAAATTAATTTAAAAATTATGATAATTTCTTCAATTTTTTTGAGTGGCATAAACAAATTTGAGCATTTAGAGACATTTATCTACCT

>Cbs_4R-37_cons

TTAATTTATTAAAAAGATAAATGATAGAAATAAAAAGCTTCTGTTTTACATAAATTAAAAAATAAAACAAATTCGACAAAGTAAATTTAAAAATAAAACAAATTTCTTTTTATTTTTTTGGTTTTTTATTTTAAATTAAACCTTAAATAATTTAATTATAAAATTTAAAGTAATTTAGAAAATATTCATTTGTTTAAAAATAAACCAACCTCTTTTTATAAACCAATTATATATTTTTTAAATTAATTCTTTAAATAATATCTTTTTTATAATTAAATAAAAATAGGAAGGAAGTCAATCATAAATATTTTGTTATTTTAATATCTGATTGAGCAAGAATTATTATTATAAATTCTCAATATCTAGACACTAGATTATTTTTATTTAATTATATCAAAAGAATATTTCTTATAAT

>Cbs_4R-38_cons

TTGTTTATCTAACTAAGAGCTTATTCAGGAATAAAATTTTAATATTTATTTATTTTGAATAAACATATTTTAAGAGTTAATTTTTTTTTAAGCAATAAAAACTTTAATAATTTTATAAACTCAGAAAATATCAGATAAAATATTTAATTGTTTTATAGTAAATTACTATTTTCATATTTATAAAAAATCAATTTGTTGTTTAAACCAACCTCTTTACTTAGTTTTAAAATAAAATTTAAATAAATTAGATTAAAAAATTCAATTATTAAATTTTTCTGGAAATACTCACAATTTTTAATTATGTAAATAAAAAGCTAATAATTAATAGATTAGTTGATAAAAAATATTTACTAGATTGGCTGGTGTAAAAGCATAAATAGAAATAAATGATTAAAAGATTATTTTTAAAGGATAG

>Cbs_5L-1_1A

AAATTTGCTTTAAATATTTATTTTTATAAATTTCTATTAAAAAATGGTCTAAGAGACATAGAATAAATTTTAGGATAATTTTTTATTTTATAAATTAAAAATTTTTTGAATAAACCAAATTAGAATACTTTTTTAAAAAGAATAAAATTAATTGTTGCTTTAATATTGTATAAAAATTCAAAAAGTGATAATTAATTAATAAAACCAACCTCTTTTAACGTTTTAAAAATATAAAATAAAAGTAAATTACAACTAGTTAATTATTTTAATTTTCTTAGGTAAATTTTAAATTTGATCTTATAAAAACCAAATTAAAAAAATAATGAAAGGCTTTTTATAATTATTTTCAAAATAATAATTTTTTTAAATTCAGCTTCGTAATAGAAATTTAAATACTATAATTAATTAAACTTTT

>Cbs_5L-2_cons

AAAATTTAATCAGTCTGATTTTAAATCTTAGCTAAGTAAAAAAATAATAAGCTGAGTAGATCAATAAATTCAAGTTTGATTGTAATTAACTGTCTTATTTTTAATAAAAATTTAATTTTTTTAATTTGTACTTTAGTCAAAAATTAACTTTTTAAATGAAATATCAATAACTAAAAATTAATTTGGGTTTTTAAAATAATTAAACCAACCTCTTTTATTTTACAAATTTTTTTATACTTTAAATTAATCTAAATCAAATAATATTAATTTAAATAAATTTTTAAAAGCTATCAATAAATGATGCTTTTATTTTGTTTAAAATTATTAGATATTTATTTTAATCGCTTTTTTAATTAATATAAATAAAACAATTACATTTTTTCTTTAACCATCAAAATAAAATTTGAAATTTTTA

>Cbs_5L-3_1A

TACATGAACTATTTAATCATCAACTTTTATTAATGAAATGAGTAAATATTGTAAATAAACAAAAATAATCATTTATTTTTAAGAATTTGTGACTATTATAATAATAATAAATAAACTCTTTTAAATTAATAAATAAGAAGTATTAGTTAATTGAAAAATTTATATTTTTATTTAAAGATTTTTAAATGAACTGAATAAAAAAAACCAACCTCTTTAATATATATTATATAGATTTACACAGAATTATAAATAGCTAAAATTATTTTGCTTAACTAATTTTTAAAATAATATAAAAAAATAAATATAAACCTTATTATTTAAACACATAATAGAAACACTAAGGCAAAATTTAAAGAAAAGTAATTATTGATTTTAAATAAAAATTTAATCTTTATTTTCTCTATGTTTCATCTAT

>Cbs_5L-4_1A

TATAATATTCCAACAATTTTTTATTCTTAGAATTAATGAATACAGTGATATTTTTAGAATAAATAAAATTTTAACTTTTAAAATAATCAAAATAAAGAAATAAATAATTGGTGATAGATAAAGATTTTGAATTAATCCTATTATATTTTAATATTTAAAAATCATTCATATGAAATAAAATTTATTATCTTATTTTAGAAAAAACCAACCTCTTTTTAAAAAAATTTAATTTTCTTAAAATTAAAATTGAAAGTTTATTTAAGTTTAATTTTTTTGAGAATTTTAATAATTTAATGAAGTTTTATTTTAATTTTAAATCATTAAAAACTACCTATTAAACATAACGATTAATGAATTTTAAAAAAATGACTTAAAAATTTTAATAAATATTTAAACTAACCAAGCTTTTATTTAA

>Cbs_5L-5_1A

TTAAAAATGATAAATCCAAATAAAAATAAGAAGAAAAATTATTAATTAATTTATTTTTACTATTTTTAATAAATAAATTACAAGTAAGTTATTAAAAAATTATTTATCTAAATAAGAAAATATTAAAATAAAAAGCATTAATTAATATTTCAATTTAATTTATTTTATTTAAACTATAAGATAAATTAAAAATGAAATAAAAAACCAACCTCTTTAAAATAGAACTGAGCTGTGTAATTTATTTCAAATTTAAATGACATAAAAAAAGTAATTACTCCTGGAATCATCTTGAAAGTTGCTATTTTGCTATTTAAAATAAATTAGAAATATTTTTTAAAAGTTTTTTTGTGATTTGTTGAGATTTAATAGCGTGTTGAGATAATTTATAAATTAAAAAAAAATTAATATTTATAAG

>Cbs_5L-6_13C

TTACTTTTTGATTGATATTTATTTTTTTTTTAAAAAATTTTTTATACTTTTTTTGAATTATTTTTGGAAATAAAAAATGTAAATAAAAATGCTTAGTATTAATTATAAGTAAAAATTAAAAAATCAAGCTCTGAAACTATTTCAAAAATGTATTTTAAACTAATGGTTTCTATATTTAAATATATAAAATTATTTTAATATAAACCAACCTCCTTTTTTTAGTGTTTTATTAATTGAAGATTAAATAAAAAAGATGAAAATTTATTATTAATTTTTTTATAAAATGTATTTGTTAATTATAGCTTAGAATATATATTAATTGATATTTAAAATCATAAACTAAAAATATTTAAATATTTATTTATTTATTGAATAAAATTAATAATGTAAGAATAAAATATATTTTTAAGAGAAT

>Cbs_5L-7_cons

CTTTTTATGAAATAAATTTATGAATTATTTAAACTTTAAAAAAAAAATGTTGTGATATGAAAAAAAATTAAAACACTTTATGCAAGAAATAAGGCAACCACTTTATATTTTTGATAAAAGTGTAAATTAAAGAAAGAATTTAAAAAAATTTTATTCTTTAGAATTTAAAAAATTTAATAAAGGAAACAAAAATAAATATCTAAACCAACCTCTTTTTATATTTGTTTATGTATTTTTATAAATTAATTAATTAATTAAAATTAATGTAAAAAAAGAATTGATAAGGATTTGATGTTTATTTAAAATTATAAACTTTAAATCTTAGTAAGTTAATTAATTAAACATTTAATGATAAATGTGGAAATTGGTAAGCAAAAAGTAATGTTTTAAAGATATTAATTAAATATCATAGATT

>Cbs_5L-8_cons

TGTAATCTGATATATTAAATACTCTTGAATAAAGTAAAACTTTATTTAAATATTTATGTATTTACTTATGTGTAAATTTAATAATATATTATTTCTTACAAAAAAAATTTTTATTAGCTAAGTAGCGATATTAAAAAGTTCATAAATGAAAAAAAGTGATGTAATTAATACATTTTTATATTAAATCAAAATTTTGTTAATAAACCAACCTCTTTGTAAAAATTAATTGATATTTTTTGGAATAAATAAATTAAATGGTCTCTTTAAAAATAAATACTTTTATTTATGTTTCTTAAAATTGTATTAAAATCAACAAAAATTGTGATAAGCTAATTATTTAAAATCGTATCATTATTTTCTAAGATTTTAATAATGAAAAAATATATTACTGCATAACCAAAAGCAACAACAATAA

>Cbs_5L-9_cons

AAAAATCATACTCACTTTATATTTAATTTATTCTTGATTAAATTAATTTAAATTTAAAAAATATATTTATTTATATTTACAAGTTGGTAATTGATTCATTTAATTATTAATTAAAAATGTTTTAAATGGTTTCTTTATTTTGATAAATTATAGAAAATTAAAAATTTTTAAAATATATATTTTCAAATGCCGAACGAAACTAAACCAACCTCTTTAAAATTAATTCTTAAAACTTTTAAAAACATTAATATAAAAATAAGATAAGATTTTAAATCTAATTAACTACAATACCAAACAAATTTTGTTTAAATTATACTTTTTACAATGTATAAAAACTTTCACTCTATTTTTTATAACTTGTTATCATGAAATTATTAAAACTCTTACAAAAATTTTATTGAAAATTATTTTAAGA

>Cbs_5L-10_cons

AAAATATTTTTATTAAAATCTCATCATAAAAAAAAATATTTTTAATTATATTTAAATCAAATAACTGCTTTGATTGGATATTATTATTATCAATTTTACAGTTTTATATGTAAAAATATAATGTTTTAAAAGGTTATTTTATTTTGATAAATAATAAAAATTAAAAAATTTTTAAAGTAAGTATTTTTAAATGTCTAAACTAAACCAACCTCTTTAAAATTAATTCTTAAAACTCTTAAAAACATAAAAATAAAAAATAAGATTTTAAATTTAATTAAATACCATATTCAAACAAGAAATTATATTTTTTACTATGTCTACTCTTAATTTTCACATTATTTTAGATAACTCATTATCATGAAATAATAAAAAATCTTACAAATATTTTATTTTAAATTATTTATGCTGTAAATAA

>Cbs_5L-11_15A

AGTCATACTCGCTTTATAATTAAAATTAATTTTTGATTAAATTAATTTGAATTTAAAAAATATATTTATTAATATCCACAATTTGATAGATTGATTCTTTTAATTATTAGTATATAATCTTTTAAACGGGTTCTTTACTTTGATAGATAAATTATTGAAAATTTTATATTTTTTAAAGTACGTACTTTTAAATGTCTGACTAAACCAACCTCTTAAAAACGTAAATATAAAAAAATAAGATTTTAAATTAAATTAACTACCATACTTCAGCAAATTTTGCTTGAAATTAAACTTTTTACTATGTCTACTCTTAACTTTCACACTGAGAACTCATTATCTTTAAATTATCAAAATTCTTACAAACATTTTATTTTAAATTATTTATTCTGTTATAGGATGATAATTAAATTTTTTA

>Cbs_5L-12_1A

ATATTTAATTTATTAAATTATTTTTTAAAAATTATTTAATTGGGAGGAGTTTTTAAAAATTTACAACAATTTATTTATTCTTTAAAAACTTAAAATTTTTTGTAAAATTTTAAAATATTAATTACTATTTAATTGATATTCCTTATTTTTTAATTTTATTTTTAATAAATATTTTTTATGTATTATTAACATTTTTTATTAAAACCAACCTCTTTTTTTACATGCTAATAAATTAATTAATGAGTTAAGTGTATTCGAAAGAGTATTTTAAATAATAACTTTATTAAAATAAATATATTGAAAGTATAAGGATGTTTGAGACTATCATTTAAATATTTTAACTAAGATAAATTTATTTTAATTTCTTTAAATATTACCTAAATTGATAAAAATAAATATTAATTAGGAAAAACAA

>Cbs_5L-13_1A

TTATTATTTTTTTTATTATTAAAAATCTTTATACTCTTTATACTAATTTAGTTAATTATTTATGATTATTAATAAATAACGTCACATTACTTTTTATACCAATCAAACTAAAAATTTTAATACTTTTCTTAATTTTTTTGTCTTTCTTTAATATTATTTTTACTATTTTTGGATTTATTTACATTTCTATGAAATTTTCTAAAACCAACCTCTTTTCATCATTAGGTTAAATATTAATCTTGTTTGTATTCTATATTTTTTTAATTATAAATCTAATTAAAGGCATAATTATTTACACCAAAATAACACAATCATCAAAATAATTTTTACTTTCCTCTTAAAAAATAAATTTTATTAATAGATAAATATTCCACTCTTTTTATTTAAGCTTTATATTCATTCACTTTTAATTTTT

>Cbs_5L-14_cons

TTACTTAATTTAAACTTGAATAAGCTTTTAACTTTTTTCAAAATTTTACAATTTTAAAAATTAGAGAATAATTATTATTAAATATTAATAAATTTGACTGTAAATTAGCAGTAGCTTATTTTATAAAAAATAAGAATGACTGATTTCAATTTTTAATATTCAATAGATAATTTTATATGATATTTTATTTTGTATAATTATAAACCAACCTCTTTTTAAATTTAAACATAAATATTGTTAAATCAAAAAATAAATTATATTGTTCATATTTATAAAAAAAATCTGAGAGACTCTTTAATTGTATTTTTGATATTTATAGTGGCGACTTGATTAATTTGAAATACTATAACACTTTTTAAGATACTTAAAATAATAATTAAATAAATATATAAATTTATGGCTAATAATAAAAATG

>Cbs_5L-15_1A

TAAAATGCTTATTTATATTAATTTAAGTAATATCAATTAATTATTTATCATCAAATTATTTTCACTTCCATATAATTTAAAGCTATTTTGACTAATAGTTTTTTTAGTTAAAAATATCTATCTATCAATCTACTTACTTTTTGAAGATTAAAATATCTTAAAATAATTATTAATTTTTAGTCTGATTAGAATTAATTTAAAAAACCAACCTCTTTTAATATTTTTCTTTTAAAAAAAATAAAAAAAAAACTCTCTTATAAAATTTTGAAAACATATTCCTTGAGAATAATAATATTAATAATTTATTAATTGTAAAATAAATTAAAGTCTATTTAAATAACTAGAATACATTAAAATAAATATAAAAAAATAAGCATTAATAAATAATTAATTAGTAAGTAAATTTATTTTATTT

>Cbs_5L-16_cons

GATTTTTTTAATTTGCAAAATTGCATAATTTAAAGTTTGCATATTATTGTAAAAATGAACTAAAGGTCAATTTCCATAAATTAATTTTAATGTTTTTTTACTTTAAAAAAATCTAAATAATTTAATAAAAATACATTAATCATAAATAATAGTAATCATCTTGAAGAAGTTGGTTAATTATGGAATAATTACAATATAAATAAACCAACCTCTTTTAAAAATGTAAATTTACTAAAGTTTAAAAATAATTAGGTTTCTAAATATTTTGTAAATAAATAATAAATTAATTTATCTTAAGAAAAAAAATATGGAAATTTATTTTATAAAAACAATATTTAAATATAACTTAAGACTAATTCAAACTTTAAATTTTAAAAATATGTAAATAACTTATCCATCAAGGAAACACATCTCT

>Cbs_5R-1_1A

CATATGAGATTAAAAATATAAAATAATTCATAGATAAAATAAATTTCTTTTAACTTTACAAATTTTTGTGGTACTCCAAAATATTAATTTTTTTGAAGGTTTAAAAAAGAAATTATGATTATTATTTAAAAAAAAGACTTAAATAATTAATTACTTCAAATTTAAGATAAATTTAATTAAAAGTATATCAAAAGATAAAAAAAACCAACCTCTTTTATTCTTTCTCTTAATATTAATTTAATTTGATTAAGTCAATTAATTAATTTTTTGGAAAATAATCTAGCTGAAATATAGGAAAATAAATACATTTATTTTAAAATTAACAATTATTTAATAAAAAAATATTAAACTAATTTTCATTTTAATTTATATTTCTAAAAAAATATATTTATTTTAACATTAGTTAAAAGTGAAC

>Cbs_5R-2_1A

ATGACCTTTACAAACTTAATTTCAAGTTTATCAAAATATAAAATCTTAAAAACATTTTCTAATTTCTATTTTTTAAAACTTTATTTAAGGTTAGCACAAAAAAATGCCATGAAAAATTGTTAATTAAATAAAATGATAAAGTCTAAATTTAGAAAGAAAAAAACCATCAAGTATTATTTTTGATTTTTTTTATTTCTCAAAAAACCAACCTCTTTAATTTTTTGGAGGATCATAAATGTTATATAAAATAAATAAAACACATTAGCATTAATTTTCTTTTGAGAAAAATTTTATTTTTTTTAATCTAAGTTTAAAATATCTTAAAAACACTTCTAATAAGAAACTCAAAATTTGTTAGAAAGAATTTTAGAATAGTTTTAAGTTTTTTATTATAAATTTATTTTTATTTGAGTTA

>Cbs_5R-3_1A

TAAATATCAAATTTTTTTTGAAAATTTTTAATAAAATTGAATTATTTATTTATATAATCTCTAAGAAATTTAACAATGATTTATTTTTATAATCTAATTTTCCATTAAATTTAAATTTTAGAATCAAATTCATATTTGTAAAAATCAAAATAAATTAATTATTAACTTATTAATCAAGAAATAAAATAAAAAAAAAATTAAAAACCAACCTCTTTTTTTATTTGATTTTAATGAATTAAGTAAAATCAATTGAGGAAAATAATGTAAAAATTTATATTCATTTTTCTTTCTTTTTAAAATATTTTATTGTTTAAAATAAAATAAATAAATAATTTTTGTTTTATAAAACATTTTTTTTTATTTTTTACAAAAAAAAATTAGTAAATTTATTGTTTTTTCTATTTTTTCTTTGTGT

>Cbs_5R-4_cons

AAGGAATATATCCTCTCTAAAATTATTTTAATAGCAATTTTTACAAAAATATTCAAAATATCTTTTCATTTTTTTCTTTTTACAATACAATTTTTGTTATCTTTAGAGTTTTCAAAAAAAATAGATTATTAAAAATTATTGATAATAAGACAAATGACTGAAATTAATTACATTAATCTTTGAAAATTTTAGCATATAAATAAACCAACCTCTTTTAAAATTAAAAAAATATTCCAAATATACTATGTTGATTGATGATTTTTTAAATATTTACGATTAACCTATTTTTAATTTTCATAAATTTTTAAATTCATTTTCAAAACCAATTAAAGAAATTAATTAATTTTTACAAATTTTATAAATAATTTGACTTAAATTTAAAAATAAACTTTTGAAATATTTTGCATTTAGAATA

>Multi_Cbs_5R-6_cons, 5R-5_cons; 468 bp

TTTTATTTTCTAAAAATAATAAGATATTAAACTAAAAAAAAGAAAAATATAGTTAAAAAAATAATATTTGAATTAAAACAAATGCTCTATTTAAATTTCAAAATATTAATTAAAAAATAATTTTTTTATATAAAATTAAAAATAATAATATTTTTAAGCAAATATTATTAAAATAAGTATATTAAACCTAATTAATATTATAAACCAACCTCTTTTGTTATTAAAATAAGTATATTAAATCTAATTAATATTATAAACCAACCTCTTTATATATTTTTTAATGATATGTTTAATAATTAATGAATTTAAAAAAAACCAATATCCTAAAGATAAATAATATTAACATTTTTATTTATCATTCTTTGATAATTAATATTAAATAAATTCGCTAAAATGATGATTAAATATCTTTTTCCATTCATAATTATGTTTATTAAAATCTATCAAAAATCAAATAAAAACAATTTA

>Cbs_5R-7_cons

TTTTTATTTTAATAAATTTATTTTAAGCATACTTAAAATATATTTTGTTATATTTATAAATAAACTTTAAAACAAATTAAAAAAATATATTTAAGCTTATTTGTTTCTTTATTTGAAGTTAAAATTTTCAATTATTAATAAATTTTCTTAAAAAATGTTTGAAATAATATTAAAAATAGTCTCGAATAATAAAGTAAAAATAAACCAACCTCTTTTAAGAAATTCCTGAAATATTAAATATAATTTTTTAAAAAATTAGAACATATTATGCATTAGTATTTTAGAAGTGAAATTCTATTAATTAAACAGTAGAAAAACATAAAATTACAAATAGAATATGATAATTTATTTTATCAATTTTTTGTAGCAATTATAATTAATCCATCCAAACCTAGAGGCAATTTATTCAATTAGA

>Cbs_5R-8_14C

TCTAAATATCTTATAAATTATAAATAAATAAACATGCATAATCATTTGTTAATAATTTAGAAAAATCAATAATTTAGATTAAATTGTTTGTGCTTCAATGCAACAAACTCCTAATCAAATAATATAATTTATTTTTTAATAATTCAACTTAAAATATAAAATCTAGTAAATAATAAAATTAAATTAATAAAATTTAAAATTAAACCAACCTCTCTCTCTTTTATTATTTATATAAAAATTGTTTTTTTTATTAATTTTTATTTTATAAATCTTAGATTTTTAATTTTTAACTTTATTATAGAAATTAATTACCGATAAAATGAATAAAGATAATTTAAATTAAAGCAAATATCTATTATCGATTTTACATATTTTGGTCATTTTGTTAATAAATATATTTTTTTTTAAAGTATTG

>Cbs_5R-9_cons

ACATTATTATTATTTATTAAAAAATAGATTGTAAATTGTTTGATTATTGAAAAATATTTTATTTCAAAAATTATTTTGGTTATTTTTTTGAATAAATTGAATGAAAAAAGCAAAATTTATTTTAAAAATGTAAATTTATTTGTTTAAAATATTGAATAAATTTAATTCTTTATTAGTTATTTTTTTAAATATTTGCTCACTAAACCAACCTCTTTGTCAAGAAATTTTTTAATTTATATTTTAAATAAGTAGATAAGATTTTTTAATTTAAAATAAGTTTATAAAAAATTTAAACAAATTACTAAATATCTTTTTTTGTTAAATAAACTAATTAAATTTTGATGTAGTAATAATAATAATAACTAAAAGACTTTAAATTTAAGAAAGTACTTTAAAACTCTGCAGAAACCTAGAC

>Cbs_5R-10_1A

AAATGTATGAATTTATGATTATGAAATGCAGATATTTTTTAATAGTTTAAATTTCATATTTGTTTTGTGAAGTTTTTTGATTAAGTAATATTAAATTTTTTTTTTTTATTTTAATAGGAAGACAATAGTAAATTTATTTAATTAACAAAAGTTAAAAATAAATACAAATAAAGATAAATCAACAGCGAATACAGGCAAAAAAAACCAACCTCTTTTTAAATTTTGCTAATTAAATTAATTGATAAATAAATTGATTAATTTTTATTCTACTTTCAAATTATATAAAAAATGCATATTGTGTCTAAAATTGAGATATCTACTAACAATTTATTTCATAAATAGTTAAGTTAATTATTTTTAATTCAATTTTTATCAAAAATTATTAAATAAATAACATAATTATATCAAATGGAGC

>Cbs_5R-11_cons

TTAGCATAGATTTCATTTATTTCAAATATTTGATTAAGTGGCTCATTAATAACAAAAATAAAAATAAATTTTAAAAAAGAATCGTAATTATCTATTAATTAATAATAAATAGTACAGTTTATTTATTTAGATATGAGTTTTGAAAAAAGTTATTAACAAATTTTTATAAGATTAAAAGATAATTCAAATTTTAATAAATATAAACCAACCTCTTTAAATTTTTTATTGATACTTTAATTAAAATATTTTTATTTCTTTTAATTTTATAATTAAGATTATTATCTATTTAAATTTTTAAGCCTAAAATTCAATTTTTTTGTAAAAAAAATATTTTAGCTTAGTAAATATTTATATTAATTATCTTTTTATATTTGTGTTTTGATTTTTCAATTAATTCAAAATAAAAAATTTTTTT

>Cbs_5R-12_cons

TACATATAAAGACTCATTATTTTATTAAATATTTATCTGTTTGTTTGATGAATTCTTTAAACAGGCAAATATAAATTTAAAAAATGCTAATTATTGATGCTTGTATTAATAAAATATATATTTTAAAATATGATTGAAACAAAATACTTTCATTTTTCAAATATTAAAAATAATTTTTATTCATTTTTTTAATTACTTTATAAACCAACCTCTTTTAATCTATTTATCTCAGCTTAAATTATATTAATAAATTAAATTCTTTTTTCTAATATTATTATGCTTAACAAAAATCAAAAAATCAAATTTTATCTTCTTTCTTTACTTAATATAAATTAAATTAATAAATACATAAAAATTAGTAATTTGCTCTACAAAAATCTAAATATTTTGAAAATAATTTTTAAAAGAAATTGAA

>Cbs_5R-13_1A

AACTTTCTCTTCAAATTCAAAATTTTTTTGTTAATTTTAAAGCCAATTTTCTCAATATAATTTCTATTTTGGATTTTTAATTACTGATAAAATATCATTCCTGATATTTTAAAAAATTTTCTTTTACAAGTTTCAACAGTTTTGTTTTATTTCAACAATTAATTATTAAATTATTAAAAAAATTTAATTTTTAAAACTTTAAAACCAACCTCTTTTATTTAAATTTTTATGTTTTGCAGATCGCCAGGCAGCCAATTTTTCATATTTGTATATGTAAAAAAATACATTTTCATTTTAGAGTAAAAAAATATTTTAAATTTGCAAATAGCATTTATTATATTTTATAAATTAATTGTAATGTTTTTTATTAAAACTTAGCTTTTATTATTACTTATTTATTCTCATATTATTTTAT

>Multi_Cbs_5R-15_1A,15A, 5R-14_1A; 499 bp

AAAATTCAAAAAAAATTTCATGTAAGTTAATCAGAGATTAATCAAAATTTTTATTGAATTAATATTTTTTTCATTAAAATAAATCAATAATAAAATAAAATTTAATTTTGTAAAAAACAAAAACCATTTAATAAAAAATTATAATTATAAAAAAATCAAAACTTTTTTTAGGAATAAAAAATTTTTTTCAATAAAAATAAAAAACCAACCTCTTATTTTAATTATGTAAAAAATCAATTAGTTTAACAAAAAATTTTAATTACATAAAAATTAATTATCAAATAAAAACCAACCTCTTTTTTTAATTAAAAAAAATCAAATCAATTTAATAAAAAATAAATAAATCTACAAATAATCTTGTAAAAAATTATTTTGCTTTTTAGCTTATTACTCATTTCTTTAAATTTTCAATTCAGTGATTTTTTAAAATTAGACAAATTAATTAAAATTTTGATTGATTTTTTTAATTTTGAGCTTATAAATATCTCTTTGCTTTATT

>Cbs_5R-16_cons

TAGATTTAGTTAAATCAAATGTTACGAATTAGAAATATACTTGAAGTGCTTATTAGCTATTAATTATTTTTTTGAATTTTTGATAAATTGATAATTATTGATTGTAATGAGAAAGTGTAAACTAGCTTTTCTTAAAATGAATAATTATATATAGTCTATATAAACAATTTAAAGAATTTTTTTTTAATTAATAAAATTATTAAACCAACCTCTTTATGAAGATTTTCTCAAAAATGTTAATTAAAAATCTAATAAAAACACTAATTTAAGTTATTACTTAAAAATTTTTTAGTTAAAATTATTTTATAAAATAATTGTTTATATAGATATATCTATCGTTGAAACATAGAGTAAGGTTTATTAAAATATAATGTAATATCTAATAATATATTTATAATTTTTATTTATTTACTTA

>Cbs_XX-1_cons

TAAAGCTTAGCTTTTAAATTAAATTTTTTTTTATAAACCACTTTTTAACTTAATTAACATTTTTTTAATTTATTTATTTACTTTTTATCATATTTAAAAAAAATAACTAATTTATGAAATTTATTTATATTAAAGGAATTATTTTTAAATTAATTTCTTAATAAAATAGAGTTTAATTTTTAAAATATAAACTTAAAACATAAACCAACCTCTTTTTTTTCAATAATTTAATATTAATTTATGAAAAAAAAATATTTAATTAATTTATTTTAAGAAAATTATTTTGGAAAAATATAATTTTTTATTGATCATTTTTAATTTTTATATGAAAATATGTAATTTAAACATTTAAATTTTTAATATAAGATATTAAATTAAATTAGAAAATAAGAGTTAATTTTATTGTTTAAATAAG

>Cbs_XX-2_cons

GTTAATCTAACTTATAGCTTATTCAGGAAAAACATTTTAATATTAGCTTCCTTATTTTGAATAAACATATTCTCAAAGTTTTTTTTTTATTCAGTAATAAAAATTTTAGTAAAAACTCAGAAATATCTAATAAAAATATTTATTTATTTGTTCGATAGTAAAATACTATTTTTATATTTATAAAAAATCAATTTATTTTTTAAACCAACCTCTTTTCTTAGTTTTGAACTTAAATTTTAATAAATTTGATTAAAAAATTCAATTATTCAATTTTTCTAGAAATATTTTTAGAAGAAAAAAAAATAGATTCTTTTGATTTTTAAAAAATTTAATTAACTCTTTATTTAAAAACATCTAAAAGCAATTTTTATCTTTATAGAGTATACGAATATATCTATATCAAAAATATATTAAT

>Cbs_XX-3_cons

TAAAGCTTAGCTTTTGAATTAAATTTTTTTTATAAACCACTTTTTAACTTAATTAACATTTTTTTAATTTATTTATTTACTTTTTATCATATTTAAAAATAAATAACTAATTTATGAAATTTGTTAAAATTAAAGGAATTATTTTAAAATTAATTTCTTAATAAAGTAGAGTTTAATTTTAAAAATATAAACTTAAAGCATAAACCAACCTCTTTTTTATCAATAATTTAATATTAGTAATGAAAAAAAATATTCAATAAATTTATTTTAAGAAAATTATTTTGCAAAAATATAATTTTTTATTGATCATTTTTATATGAAAATATGTAATTTAAACATTTAAATTTTTAATATAAGATATTAAATTAGAAAAATAAAAGTTAATTTTATTGCTTAAATAAGTTAAGTAATTAAA

>Cbs_XX-4_cons

TAACTAAAAGCTTAATGGAATTATTTTTAATATTTATTTATTTATTTATTTACTTTGAATAAACATAGTCTAAAAGTTTATTTTTTTTAAGTAATAAAAATTTTAGTAATTTTATAAATTCAGAAAATATCAAATAAAAATATTTAATTATTTTTTTGTAAACTATGATTTTTATATTTATAAATTATCGATTTGTTTTTTAAACCAACCTCTTTGCTTAGTTTTAAAGTTAAATTTGATTAAAAAATTCTATGAATTTTTATAGAAATATTCACAATTTTAATTATGTAAATAAAAATTTAAGCTGATAATTAATAGATTGATTGATAAAAAATATTAATTAAATTGGTTGGTGTATAAGCAAATATAGGAATAAATGATAATTAGATTATTTTTAAAGGATAAAGTGTTTTAA
