# Supplementary material for: Structure of the germline genome of Tetrahymena thermophila and relationship to the massively rearranged somatic genome
Source: eLife. 2016 Nov 28;5:e19090. doi: 10.7554/eLife.19090 (PMC5182062; doi:10.7554/eLife.19090)
Supplement: Supplementary file 1. — (A) Genome assembly statistics. (B) T. thermophila superscaffolds assembled by HAPPY physical mapping. (C) Chromosome super-assemblies. (D) Suspected Chimeric MIC Supercontigs. (E) Most centric MAC chromosomes are among the ten longest MAC chromosomes. DOI: http://dx.doi.org/10.7554/eLife.19090.025 [file elife-19090-supp1.docx]

**Supplementary File 1A**.

Genome Assembly Statistics

|  | MAC (Sanger) | MIC (Illumina) |
| --- | --- | --- |
| Genome Size (Mb) | 103 | ~157 |
| Sequence Coverage (fold) | 9.1 | 169 |
| Chromosomes | 181 | 5 |
| Finished Chromosomes | 123 | 0 |
| Scaffolds | 1158 | 1464 |
| Scaffold N50 (kb) | 520 | 486 |
| Contigs | 1778 | 12923 |
| Contig N50 (kb) | 367 | 25 |

The 157 Mb MIC genome assembly contains 1464 scaffolds, and 50% of its full length is contained in scaffolds greater than 486 kb (N50 = 486 kb). These statistics compare favorably with those of the Sanger-based MAC genome assembly. However, the MIC assembly contains many more intra-scaffold gaps than that of the MAC, reflected in a seven-fold greater number of contigs and fourteen-fold lower contig N50. Most (66 % by number) of the MIC assembly sequencing gaps fall within putative IESs, indicating that many of these shortcomings are due to the repetitive nature of IESs and assembly breaks caused by MAC contamination. We used nucmer (with cutoff criteria of >95% identity over >1000 bp length) to align the scaffolds of the MIC and MAC assemblies to each other. Over 99% of the MAC genome assembly length aligns to the MIC assembly under these criteria. In addition, the MIC assembly includes nearly all instances of Cbs required to account for the ends of every MAC chromosome; those not included have been shown to fall within intra-scaffold sequencing gaps. Thus, we conclude that the MIC assembly is substantially complete.

**Supplementary File 1B.**

*T. thermophila* superscaffolds assembled by HAPPY physical mapping

| New super-scaffold name | Component scaffolds |
| --- | --- |
| 8254305 | +-8254305:[7254543]:-8253957+ |
| 8254332 | +8253843:-8254332+ |
| 8254361 | +8254361:8254021+ |
| 8254381 | +8254381:-8253930+ |
| 8254412 | +8254412:8254391+ |
| 8254417 | +8254417:8253807+ |
| 8254436 | +8254049:-8254436+ |
| 8254517 | +8254415:8254517+ |
| 8254524 | +-8253973:{[8254060]:[8254247]:[8254419]:[8254499]:[8253893]:[8254508]:[8254414]:[8254443]:[8254524]:[8253934]:[8254426]:[8253829]:[8254507]:[8254003]:[8254022]:[8253845]:[8254510]:[8253918]:[8254012]}:8254482+ |
| 8254544 | +8254095:[-8253971]:-8254544+ |
| 8254552 | +-8254550:8254552+ |
| 8254579 | +-8254092:8254579+ |
| 8254587 | +-8253904:[8254134]:-(8254223):-8254587:8254369+ |
| 8254589 | +8254515:8254589+ |
| 8254617 | +-8254617:8254578:8254142:-8254528+ |
| 8254637 | +8253834:[8254701]:-8254637+ |
| 8254656 | +-8254631:-8254608:(8254238):-8254656:8254576:8254590+ |
| 8254686 | +-8254686:8253906+ |
| 8254691 | +8254634:?8253956?:8254691+ |
| 8254709 | +8254058:[8254709]:8254650+ |
| 8254716 | +8254406:-8254716:8254671:8254655:8254652+ |
| 8254759 | +8254549:[8254520]:(8254271):[8254472]:{[8254434]:[8254759]:[8254074]:(-8254678)}:[8254471]:-8253899:{[8254604]:[8254518]:[8254699]}:8254708:?8254129?:-8253994+ |
| 8254767 | +8254767:{[8254680]:[8253846]}:8254639:[8254536]:-8254484+ |
| 8254776 | +-8254776:8254702:8254653:8253902+ |
| 8254778 | +-8254481:{[8254152]:[8254613]}:-8254557:-8254751:-6254543:8254477:-8254778+ |
| 8254786 | +8254496:-8254675:8254717:8254783:8254679:-8254692:8254710:-8254786+ |
| 8254791 | +-8254670:[8254687]:-8254047:[8254553]:8254389:-8253931:8254665:8254663:-8254672:8253897:-8254465:[8254424]:[8254784]:{(8254741):[8254790]:(8254432):[8254242]}:8253978:[8254355]:[8253932]:-8254372:(8254399):{[8254445]:-8254216}:-8254302:{[8254405]:[8254562]:[8254523]:[8254738]:[8254700]:[8254739]:[8254641]:[8253853]:[8254176]:(-8254791)}:8253842:{[8254105]:[6254718]:[8254224]:[8254450]:[8254174]:[8254511]:[8254119]} |
| 8254794 | +-8254512:-8254572:8254764:[8253910]:-8254575:{[-8253818]:[8254488]:[8254554]}:8254794+ |
| 8254798 | +8253864:{(8254660):[8254421]}:(-8253989):(8254729):(8254585):[8254620]:8254685:[8254053]:[8253873]:[8253970]:[8253976]:{(-8254683):[8254789]}:-8254513:-8254573:-8254097:8254368:8254288:8254798+ |
| 8254802 | +-8254707:8254647:-8254690:{[8254694]:[8254713]:[8254531]:[8254797]}:8254802:8254788+ |
| 8254803 | +8254803:-8253900:8254159+ |
| 8254810 | {+8254662:8254612:8254633:8254490:8253997:8254715:8254775:8254805:-8253856:-8254627:8254559:8254299:-8254570}:-8254556:-8254409:-8254603:8254104:8254754:(8254280):(8254728):[-8253933]}:(-8253981):{[-8253896]:-8254466:-8254810}:{[-8254569]:8254796:[-8254516]:8254676:-8254033+ |
| 8254815 | +-8253848:{[8254101]:[8253903]:[-8254596]:[8254779]:[-8254407]:[8254519]}:-8254646:8254815+ |
| 8254817 | +8254394:{[8253838]:[8254453]:[8254052]:[8254677]:[8254175]}:8254720:[8253860]:8254732:[8254689]:[8254558]:[8254526]:[8254561]:[8254816]:{[8254703]:[8254139]:[8254755]:[8253865]:[8254771]:[8254307]:[8254108]:[8254681]:[8254817]:[8254230]:[8253985]}:-8254606:(-8254758):(-8254388):-8254711+ |
| 8254820 | +8254761:[-8254762]:(-8254478):8254521:8254380:[-8254357]:-8254623:-8254769:8254383:8253855:-7254718:-8254813:{[8254772]:[8254642]:[8254532]:[8254539]}:-8254542:[8254220]:-8254598:[8254734]:8254766:8254820:[8254215]:(-8254547):[8253851]:-8254800:{[8254364]:[8254808]:[8254726]:[8254501]:[8254493]:[8253820]:[8254287]:[8254566]:[8254614]:[8254773]:[8254533]:[8254723]:[8253868]}:8254085:[8254425]:8254500:8254760:8254591:8254179:[8254390]:-8254529:-8254007+ |
| 8254821 | +-8254375:{(-8254684):(8254765):(8254651):(8254233):(8254821)}:-8254781:8254693+ |
| 8254822 | +-8254265:8254418:-8254571:8254263:-8254463:-8254386:[8254461]:-8254438:8254661:[8254635]:-8254363:{[8254744]:[8254430]:[8254621]:[8253835]:[8254411]}:-8254822:[8253840]:-8254416:-8254586:-8254619:[8254698]:[8254439]:8254628:-8253928:8254626:-8254583:[8254696]:{[8254704]:[8254494]:[8254366]:[8254278]:[8254809]:[8254616]:[8254750]:[8254568]:[8254160]:[8253869]}:8254735:[8254740]:[8254408]:-8254705:[8253805]:8254736:[8254045]:[8253972]:[8254785]:-8254037:8253908+ |
| 8254824 | +-8254648:[8253983]:-8254682:8254077:[8254480]:-8254006:8254605:8254824:8254799:[8254727]:(8254770):8254804:-8254489:-8254502:8254485:8254222+ |
| 8254825 | +8254731:8254825:-8254213:[8254250]:-8254458:8254336:-8254657:-8254592+ |
| 8254826 | +8253870:{[8254099]:[8254624]}:(-8254795):{[8254801]:[8254113]}:[8254522]:-8254793:{[8254266]:[8254743]:[8254509]:[8254451]:[8253890]:[8254413]:[8254073]:[8254602]:[8254107]}:8254581:{[8254560]:[8254474]:[8254806]:[8254812]}:-8254722:[8254567]:8254636:8253992:8254601:{[8254625]:[8254668]:[8254695]}:8254629:{[8254462]:[8254538]:[8254826]}:8254658+ |
| 8254828 | +8254435:8254374:-8254828:8254674:-8254492:-8254376+ |

Key to symbols. “-“ = reverse scaffold orientation; “+” = telomere. Level of order and/or orientation uncertainty is indicated as follows. Scaffolds enclosed in braces, { }, have uncertain order. Scaffolds enclosed in square brackets, [ ], have uncertain orientation but with small probability of error (~90% confidence). Scaffolds enclosed in parentheses, ( ), have greater orientation uncertainty (probability of error ~ 70%).

Scaffolds 6254543 and 7254543 were obtained by splitting misassembled Celera scaffold 8254543; likewise, scaffolds 6254718 and 7254718 were split from 8254718.

**Supplementary File 1C**

**Chromosome Super-Assemblies.**

The MIC supercontigs making up each of the five chromosome super-assemblies are listed in order. Supercontig orientation is indicated by the beginning and end coordinates (columns 2 and 3). Columns 5 and 6 are the corresponding coordinates of the super-assemblies. In the gap between each supercontig, 100 Ns were inserted into the chromosome super-assembly sequences. The complete sequences are available in fasta format on the *Tetrahymena* Genome Database website: http://www.ciliate.org. Suspected chimeric supercontigs (see Supplementary Table 4) are identified with an "x" in column 7. At the end of the table, supercontigs that could not be assigned to a chromosomal location are listed, in decreasing order of size.

| **Supercontig** | **Start coord.** | **End coord.** | **Chromosome superassembly** | **Start coord.** | **End coord.** | **Chimeric Supercontig** |
| --- | --- | --- | --- | --- | --- | --- |
| supercont2.365 | 92863 | 1 | chr1 | 1 | 92863 |  |
| supercont2.469 | 1 | 65163 | chr1 | 92964 | 158126 |  |
| supercont2.252 | 140885 | 1 | chr1 | 158227 | 299111 |  |
| supercont2.338 | 1 | 102576 | chr1 | 299212 | 401787 |  |
| supercont2.274 | 1 | 130946 | chr1 | 401888 | 532833 |  |
| supercont2.64 | 1 | 529938 | chr1 | 532934 | 1062871 |  |
| supercont2.482 | 58636 | 1 | chr1 | 1062972 | 1121607 |  |
| supercont2.34 | 822476 | 1 | chr1 | 1121708 | 1944183 |  |
| supercont2.102 | 376179 | 1 | chr1 | 1944284 | 2320462 |  |
| supercont2.9 | 2149620 | 1 | chr1 | 2320563 | 4470182 |  |
| supercont2.80 | 446114 | 1 | chr1 | 4470283 | 4916396 |  |
| supercont2.2 | 3223353 | 1 | chr1 | 4916497 | 8139849 |  |
| supercont2.780 | 13635 | 1 | chr1 | 8139950 | 8153584 |  |
| supercont2.122 | 1 | 314420 | chr1 | 8153685 | 8468104 |  |
| supercont2.45 | 668904 | 1 | chr1 | 8468205 | 9137108 |  |
| supercont2.17 | 1324283 | 1 | chr1 | 9137209 | 10461491 |  |
| supercont2.439 | 65682 | 1 | chr1 | 10461592 | 10527273 |  |
| supercont2.152 | 248339 | 1 | chr1 | 10527374 | 10775712 |  |
| supercont2.472 | 1 | 60034 | chr1 | 10775813 | 10835846 |  |
| supercont2.91 | 1 | 394370 | chr1 | 10835947 | 11230316 |  |
| supercont2.186 | 191868 | 1 | chr1 | 11230417 | 11422284 |  |
| supercont2.29 | 963340 | 1 | chr1 | 11422385 | 12385724 |  |
| supercont2.377 | 1 | 86613 | chr1 | 12385825 | 12472437 |  |
| supercont2.464 | 1 | 61649 | chr1 | 12472538 | 12534186 |  |
| supercont2.295 | 1 | 119798 | chr1 | 12534287 | 12654084 |  |
| supercont2.356 | 103065 | 1 | chr1 | 12654185 | 12757249 |  |
| supercont2.197 | 174606 | 1 | chr1 | 12757350 | 12931955 |  |
| supercont2.430 | 1 | 69476 | chr1 | 12932056 | 13001531 |  |
| supercont2.149 | 1 | 257631 | chr1 | 13001632 | 13259262 |  |
| supercont2.210 | 1 | 165530 | chr1 | 13259363 | 13424892 |  |
| supercont2.46 | 1 | 663490 | chr1 | 13424993 | 14088482 |  |
| supercont2.97 | 383829 | 1 | chr1 | 14088583 | 14472411 |  |
| supercont2.421 | 1 | 75580 | chr1 | 14472512 | 14548091 |  |
| supercont2.76 | 477826 | 1 | chr1 | 14548192 | 15026017 |  |
| supercont2.776 | 1 | 14466 | chr1 | 15026118 | 15040583 |  |
| supercont2.329 | 1 | 106546 | chr1 | 15040684 | 15147229 |  |
| supercont2.696 | 1 | 21474 | chr1 | 15147330 | 15168803 |  |
| supercont2.594 | 1 | 34306 | chr1 | 15168904 | 15203209 |  |
| supercont2.1368 | 1227 | 1 | chr1 | 15203310 | 15204536 |  |
| supercont2.358 | 97461 | 1 | chr1 | 15204637 | 15302097 |  |
| supercont2.720 | 1 | 18185 | chr1 | 15302198 | 15320382 |  |
| supercont2.280 | 1 | 19220 | chr1 | 15320483 | 15339702 | x |
| supercont2.434 | 71149 | 1 | chr1 | 15339803 | 15410951 |  |
| supercont2.286 | 1 | 122689 | chr1 | 15411052 | 15533740 |  |
| supercont2.465 | 67997 | 1 | chr1 | 15533841 | 15601837 |  |
| supercont2.164 | 1 | 227571 | chr1 | 15601938 | 15829508 |  |
| supercont2.271 | 1 | 136528 | chr1 | 15829609 | 15966136 |  |
| supercont2.229 | 153389 | 1 | chr1 | 15966237 | 16119625 |  |
| supercont2.728 | 1 | 17517 | chr1 | 16119726 | 16137242 |  |
| supercont2.245 | 144948 | 1 | chr1 | 16137343 | 16282290 |  |
| supercont2.686 | 22044 | 1 | chr1 | 16282391 | 16304434 |  |
| supercont2.505 | 1 | 52557 | chr1 | 16304535 | 16357091 |  |
| supercont2.789 | 1 | 13021 | chr1 | 16357192 | 16370212 |  |
| supercont2.551 | 48801 | 1 | chr1 | 16370313 | 16419113 |  |
| supercont2.202 | 1 | 172702 | chr1 | 16419214 | 16591915 |  |
| supercont2.264 | 1 | 138016 | chr1 | 16592016 | 16730031 |  |
| supercont2.586 | 1 | 35315 | chr1 | 16730132 | 16765446 |  |
| supercont2.697 | 1 | 21139 | chr1 | 16765547 | 16786685 |  |
| supercont2.222 | 1 | 161151 | chr1 | 16786786 | 16947936 |  |
| supercont2.597 | 1 | 35154 | chr1 | 16948037 | 16983190 |  |
| supercont2.634 | 30388 | 1 | chr1 | 16983291 | 17013678 |  |
| supercont2.784 | 1 | 13356 | chr1 | 17013779 | 17027134 |  |
| supercont2.668 | 1 | 24793 | chr1 | 17027235 | 17052027 |  |
| supercont2.359 | 94335 | 1 | chr1 | 17052128 | 17146462 |  |
| supercont2.500 | 54104 | 1 | chr1 | 17146563 | 17200666 |  |
| supercont2.1108 | 1 | 2146 | chr1 | 17200767 | 17202912 |  |
| supercont2.492 | 1 | 56587 | chr1 | 17203013 | 17259599 |  |
| supercont2.462 | 1 | 64938 | chr1 | 17259700 | 17324637 |  |
| supercont2.209 | 1 | 162967 | chr1 | 17324738 | 17487704 |  |
| supercont2.418 | 74513 | 1 | chr1 | 17487805 | 17562317 |  |
| supercont2.372 | 1 | 92314 | chr1 | 17562418 | 17654731 |  |
| supercont2.478 | 61312 | 1 | chr1 | 17654832 | 17716143 |  |
| supercont2.429 | 1 | 70042 | chr1 | 17716244 | 17786285 |  |
| supercont2.577 | 1 | 37072 | chr1 | 17786386 | 17823457 |  |
| supercont2.343 | 100390 | 1 | chr1 | 17823558 | 17923947 |  |
| supercont2.84 | 1 | 432917 | chr1 | 17924048 | 18356964 |  |
| supercont2.591 | 36086 | 1 | chr1 | 18357065 | 18393150 |  |
| supercont2.216 | 160925 | 1 | chr1 | 18393251 | 18554175 |  |
| supercont2.533 | 1 | 45171 | chr1 | 18554276 | 18599446 |  |
| supercont2.360 | 1 | 97354 | chr1 | 18599547 | 18696900 |  |
| supercont2.678 | 1 | 24688 | chr1 | 18697001 | 18721688 |  |
| supercont2.99 | 1 | 378949 | chr1 | 18721789 | 19100737 |  |
| supercont2.19 | 1 | 400681 | chr1 | 19100838 | 19501518 | x |
| supercont2.414 | 75305 | 1 | chr1 | 19501619 | 19576923 |  |
| supercont2.582 | 1 | 37320 | chr1 | 19577024 | 19614343 |  |
| supercont2.112 | 351217 | 1 | chr1 | 19614444 | 19965660 |  |
| supercont2.305 | 112753 | 1 | chr1 | 19965761 | 20078513 |  |
| supercont2.521 | 1 | 48984 | chr1 | 20078614 | 20127597 |  |
| supercont2.221 | 1 | 160041 | chr1 | 20127698 | 20287738 |  |
| supercont2.190 | 183427 | 1 | chr1 | 20287839 | 20471265 |  |
| supercont2.547 | 1 | 42872 | chr1 | 20471366 | 20514237 |  |
| supercont2.1008 | 3881 | 1 | chr1 | 20514338 | 20518218 |  |
| supercont2.457 | 62492 | 1 | chr1 | 20518319 | 20580810 |  |
| supercont2.303 | 1 | 114524 | chr1 | 20580911 | 20695434 |  |
| supercont2.177 | 210971 | 1 | chr1 | 20695535 | 20906505 |  |
| supercont2.132 | 1 | 295213 | chr1 | 20906606 | 21201818 |  |
| supercont2.119 | 1 | 347086 | chr1 | 21201919 | 21549004 |  |
| supercont2.440 | 1 | 65631 | chr1 | 21549105 | 21614735 |  |
| supercont2.535 | 1 | 16128 | chr1 | 21614836 | 21630963 | x |
| supercont2.695 | 22059 | 1 | chr1 | 21631064 | 21653122 |  |
| supercont2.498 | 56416 | 1 | chr1 | 21653223 | 21709638 |  |
| supercont2.42 | 723494 | 415163 | chr1 | 21709739 | 22018070 | x |
| supercont2.632 | 29658 | 1 | chr1 | 22018171 | 22047828 |  |
| supercont2.1027 | 1 | 3302 | chr1 | 22047929 | 22051230 |  |
| supercont2.844 | 1 | 9533 | chr1 | 22051331 | 22060863 |  |
| supercont2.257 | 1 | 138569 | chr1 | 22060964 | 22199532 |  |
| supercont2.642 | 28733 | 1 | chr1 | 22199633 | 22228365 |  |
| supercont2.606 | 1 | 32481 | chr1 | 22228466 | 22260946 |  |
| supercont2.612 | 32341 | 1 | chr1 | 22261047 | 22293387 |  |
| supercont2.247 | 1 | 142907 | chr1 | 22293488 | 22436394 |  |
| supercont2.491 | 55347 | 1 | chr1 | 22436495 | 22491841 |  |
| supercont2.234 | 1 | 151049 | chr1 | 22491942 | 22642990 |  |
| supercont2.972 | 5224 | 1 | chr1 | 22643091 | 22648314 |  |
| supercont2.645 | 26792 | 1 | chr1 | 22648415 | 22675206 |  |
| supercont2.284 | 123940 | 1 | chr1 | 22675307 | 22799246 |  |
| supercont2.108 | 360554 | 1 | chr1 | 22799347 | 23159900 |  |
| supercont2.105 | 1 | 367785 | chr1 | 23160001 | 23527785 |  |
| supercont2.67 | 495392 | 1 | chr1 | 23527886 | 24023277 |  |
| supercont2.158 | 1 | 227193 | chr1 | 24023378 | 24250570 |  |
| supercont2.330 | 1 | 104361 | chr1 | 24250671 | 24355031 |  |
| supercont2.3 | 1 | 2973454 | chr1 | 24355132 | 27328585 |  |
| supercont2.57 | 573886 | 1 | chr1 | 27328686 | 27902571 |  |
| supercont2.313 | 1 | 109270 | chr1 | 27902672 | 28011941 |  |
| supercont2.8 | 1 | 2370207 | chr1 | 28012042 | 30382248 |  |
| supercont2.30 | 859612 | 1 | chr1 | 30382349 | 31241960 | x |
| supercont2.561 | 1 | 39689 | chr1 | 31242061 | 31281749 |  |
| supercont2.538 | 1 | 44286 | chr1 | 31281850 | 31326135 |  |
| supercont2.14 | 1 | 1489856 | chr1 | 31326236 | 32816091 |  |
| supercont2.845 | 9544 | 1 | chr1 | 32816192 | 32825735 |  |
| supercont2.877 | 8263 | 1 | chr1 | 32825836 | 32834098 |  |
| supercont2.327 | 1 | 106414 | chr1 | 32834199 | 32940612 |  |
| supercont2.677 | 1 | 23435 | chr1 | 32940713 | 32964147 |  |
| supercont2.579 | 40564 | 1 | chr1 | 32964248 | 33004811 |  |
| supercont2.120 | 1 | 327221 | chr1 | 33004912 | 33332132 |  |
| supercont2.16 | 1 | 258671 | chr1 | 33332233 | 33590903 | x |
| supercont2.48 | 1 | 655811 | chr1 | 33591004 | 34246814 |  |
| supercont2.637 | 1 | 30927 | chr1 | 34246915 | 34277841 |  |
| supercont2.178 | 1 | 196495 | chr1 | 34277942 | 34474436 |  |
| supercont2.384 | 1 | 84029 | chr1 | 34474537 | 34558565 |  |
| supercont2.113 | 315365 | 344693 | chr1 | 34558666 | 34587994 | x |
| supercont2.26 | 973843 | 1 | chr1 | 34588095 | 35561937 |  |
| supercont2.214 | 166493 | 1 | chr1 | 35562038 | 35728530 |  |
| supercont2.148 | 1 | 257301 | chr1 | 35728631 | 35985931 |  |
| supercont2.654 | 1 | 26898 | chr1 | 35986032 | 36012929 |  |
| supercont2.219 | 1 | 163866 | chr1 | 36013030 | 36176895 |  |
| supercont2.312 | 1 | 111140 | chr1 | 36176996 | 36288135 |  |
| supercont2.596 | 1 | 33759 | chr1 | 36288236 | 36321994 |  |
| supercont2.189 | 1 | 187711 | chr2 | 1 | 187711 |  |
| supercont2.10 | 2098439 | 1 | chr2 | 187812 | 2286250 |  |
| supercont2.145 | 268170 | 1 | chr2 | 2286351 | 2554520 |  |
| supercont2.167 | 1 | 219777 | chr2 | 2554621 | 2774397 |  |
| supercont2.41 | 737993 | 1 | chr2 | 2774498 | 3512490 |  |
| supercont2.733 | 17030 | 1 | chr2 | 3512591 | 3529620 |  |
| supercont2.726 | 1 | 19587 | chr2 | 3529721 | 3549307 |  |
| supercont2.207 | 1 | 164114 | chr2 | 3549408 | 3713521 |  |
| supercont2.736 | 17392 | 1 | chr2 | 3713622 | 3731013 |  |
| supercont2.964 | 5346 | 1 | chr2 | 3731114 | 3736459 |  |
| supercont2.58 | 571544 | 1 | chr2 | 3736560 | 4308103 |  |
| supercont2.302 | 113732 | 1 | chr2 | 4308204 | 4421935 |  |
| supercont2.336 | 104718 | 1 | chr2 | 4422036 | 4526753 |  |
| supercont2.390 | 1 | 82077 | chr2 | 4526854 | 4608930 |  |
| supercont2.113 | 1 | 304979 | chr2 | 4609031 | 4914009 | x |
| supercont2.23 | 1 | 1028507 | chr2 | 4914110 | 5942616 |  |
| supercont2.92 | 393849 | 1 | chr2 | 5942717 | 6336565 |  |
| supercont2.400 | 1 | 78136 | chr2 | 6336666 | 6414801 |  |
| supercont2.33 | 1 | 829529 | chr2 | 6414902 | 7244430 |  |
| supercont2.532 | 1 | 45247 | chr2 | 7244531 | 7289777 |  |
| supercont2.366 | 92405 | 1 | chr2 | 7289878 | 7382282 |  |
| supercont2.31 | 894385 | 1 | chr2 | 7382383 | 8276767 |  |
| supercont2.72 | 1 | 486552 | chr2 | 8276868 | 8763419 |  |
| supercont2.201 | 171369 | 1 | chr2 | 8763520 | 8934888 |  |
| supercont2.171 | 207156 | 1 | chr2 | 8934989 | 9142144 |  |
| supercont2.161 | 1 | 224884 | chr2 | 9142245 | 9367128 |  |
| supercont2.193 | 177194 | 1 | chr2 | 9367229 | 9544422 |  |
| supercont2.228 | 1 | 156886 | chr2 | 9544523 | 9701408 |  |
| supercont2.373 | 88441 | 1 | chr2 | 9701509 | 9789949 |  |
| supercont2.593 | 34157 | 1 | chr2 | 9790050 | 9824206 |  |
| supercont2.259 | 144074 | 1 | chr2 | 9824307 | 9968380 |  |
| supercont2.546 | 1 | 41668 | chr2 | 9968481 | 10010148 |  |
| supercont2.419 | 1 | 74188 | chr2 | 10010249 | 10084436 |  |
| supercont2.277 | 126536 | 1 | chr2 | 10084537 | 10211072 |  |
| supercont2.367 | 1 | 91777 | chr2 | 10211173 | 10302949 |  |
| supercont2.548 | 41472 | 1 | chr2 | 10303050 | 10344521 |  |
| supercont2.1198 | 1 | 1700 | chr2 | 10344622 | 10346321 |  |
| supercont2.961 | 5450 | 1 | chr2 | 10346422 | 10351871 |  |
| supercont2.205 | 167451 | 1 | chr2 | 10351972 | 10519422 |  |
| supercont2.212 | 1 | 165527 | chr2 | 10519523 | 10685049 |  |
| supercont2.771 | 1 | 14388 | chr2 | 10685150 | 10699537 |  |
| supercont2.564 | 39776 | 1 | chr2 | 10699638 | 10739413 |  |
| supercont2.622 | 33158 | 1 | chr2 | 10739514 | 10772671 |  |
| supercont2.725 | 17721 | 1 | chr2 | 10772772 | 10790492 |  |
| supercont2.467 | 1 | 62320 | chr2 | 10790593 | 10852912 |  |
| supercont2.396 | 82536 | 1 | chr2 | 10853013 | 10935548 |  |
| supercont2.272 | 129091 | 1 | chr2 | 10935649 | 11064739 |  |
| supercont2.656 | 25656 | 1 | chr2 | 11064840 | 11090495 |  |
| supercont2.53 | 174489 | 1 | chr2 | 11090596 | 11265084 | x |
| supercont2.278 | 1 | 125396 | chr2 | 11265185 | 11390580 |  |
| supercont2.117 | 18664 | 1 | chr2 | 11390681 | 11409344 | x |
| supercont2.232 | 1 | 157151 | chr2 | 11409445 | 11566595 |  |
| supercont2.507 | 57483 | 1 | chr2 | 11566696 | 11624178 |  |
| supercont2.142 | 274437 | 1 | chr2 | 11624279 | 11898715 |  |
| supercont2.602 | 33549 | 1 | chr2 | 11898816 | 11932364 |  |
| supercont2.448 | 64410 | 1 | chr2 | 11932465 | 11996874 |  |
| supercont2.787 | 1 | 13178 | chr2 | 11996975 | 12010152 |  |
| supercont2.705 | 19970 | 1 | chr2 | 12010253 | 12030222 |  |
| supercont2.522 | 47906 | 1 | chr2 | 12030323 | 12078228 |  |
| supercont2.291 | 1 | 119120 | chr2 | 12078329 | 12197448 |  |
| supercont2.244 | 143176 | 1 | chr2 | 12197549 | 12340724 |  |
| supercont2.523 | 1 | 51085 | chr2 | 12340825 | 12391909 |  |
| supercont2.490 | 55932 | 1 | chr2 | 12392010 | 12447941 |  |
| supercont2.506 | 52997 | 1 | chr2 | 12448042 | 12501038 |  |
| supercont2.322 | 1 | 44851 | chr2 | 12501139 | 12545989 | x |
| supercont2.623 | 32217 | 1 | chr2 | 12546090 | 12578306 |  |
| supercont2.560 | 1 | 41311 | chr2 | 12578407 | 12619717 |  |
| supercont2.510 | 1 | 51557 | chr2 | 12619818 | 12671374 |  |
| supercont2.497 | 1 | 55187 | chr2 | 12671475 | 12726661 |  |
| supercont2.565 | 38026 | 1 | chr2 | 12726762 | 12764787 |  |
| supercont2.609 | 32096 | 1 | chr2 | 12764888 | 12796983 |  |
| supercont2.381 | 89596 | 1 | chr2 | 12797084 | 12886679 |  |
| supercont2.314 | 114648 | 1 | chr2 | 12886780 | 13001427 |  |
| supercont2.509 | 51698 | 1 | chr2 | 13001528 | 13053225 |  |
| supercont2.969 | 1 | 5365 | chr2 | 13053326 | 13058690 |  |
| supercont2.834 | 1 | 10131 | chr2 | 13058791 | 13068921 |  |
| supercont2.630 | 1 | 29192 | chr2 | 13069022 | 13098213 |  |
| supercont2.665 | 26698 | 1 | chr2 | 13098314 | 13125011 |  |
| supercont2.524 | 48239 | 1 | chr2 | 13125112 | 13173350 |  |
| supercont2.913 | 1 | 6780 | chr2 | 13173451 | 13180230 |  |
| supercont2.415 | 1 | 74988 | chr2 | 13180331 | 13255318 |  |
| supercont2.688 | 28521 | 1 | chr2 | 13255419 | 13283939 |  |
| supercont2.535 | 51810 | 20016 | chr2 | 13284040 | 13315834 | x |
| supercont2.826 | 1 | 10405 | chr2 | 13315935 | 13326339 |  |
| supercont2.870 | 8528 | 1 | chr2 | 13326440 | 13334967 |  |
| supercont2.694 | 1 | 21183 | chr2 | 13335068 | 13356250 |  |
| supercont2.854 | 1 | 9090 | chr2 | 13356351 | 13365440 |  |
| supercont2.401 | 79150 | 1 | chr2 | 13365541 | 13444690 |  |
| supercont2.641 | 31564 | 1 | chr2 | 13444791 | 13476354 |  |
| supercont2.817 | 11132 | 1 | chr2 | 13476455 | 13487586 |  |
| supercont2.411 | 77708 | 1 | chr2 | 13487687 | 13565394 |  |
| supercont2.85 | 422341 | 1 | chr2 | 13565495 | 13987835 |  |
| supercont2.283 | 128383 | 1 | chr2 | 13987936 | 14116318 |  |
| supercont2.486 | 1 | 56391 | chr2 | 14116419 | 14172809 |  |
| supercont2.514 | 1 | 49634 | chr2 | 14172910 | 14222543 |  |
| supercont2.607 | 33187 | 1 | chr2 | 14222644 | 14255830 |  |
| supercont2.848 | 1 | 9300 | chr2 | 14255931 | 14265230 |  |
| supercont2.213 | 162078 | 53380 | chr2 | 14265331 | 14374029 | x |
| supercont2.614 | 1 | 31283 | chr2 | 14374130 | 14405412 |  |
| supercont2.250 | 140627 | 1 | chr2 | 14405513 | 14546139 |  |
| supercont2.385 | 83696 | 1 | chr2 | 14546240 | 14629935 |  |
| supercont2.395 | 79949 | 1 | chr2 | 14630036 | 14709984 |  |
| supercont2.631 | 28469 | 1 | chr2 | 14710085 | 14738553 |  |
| supercont2.727 | 1 | 17554 | chr2 | 14738654 | 14756207 |  |
| supercont2.536 | 1 | 47938 | chr2 | 14756308 | 14804245 |  |
| supercont2.240 | 1 | 146020 | chr2 | 14804346 | 14950365 |  |
| supercont2.636 | 28362 | 1 | chr2 | 14950466 | 14978827 |  |
| supercont2.624 | 1 | 29611 | chr2 | 14978928 | 15008538 |  |
| supercont2.734 | 17709 | 1 | chr2 | 15008639 | 15026347 |  |
| supercont2.256 | 1 | 137942 | chr2 | 15026448 | 15164389 |  |
| supercont2.296 | 1 | 117240 | chr2 | 15164490 | 15281729 |  |
| supercont2.658 | 1 | 26327 | chr2 | 15281830 | 15308156 |  |
| supercont2.619 | 31497 | 1 | chr2 | 15308257 | 15339753 |  |
| supercont2.175 | 202264 | 1 | chr2 | 15339854 | 15542117 |  |
| supercont2.646 | 26650 | 1 | chr2 | 15542218 | 15568867 |  |
| supercont2.815 | 1 | 11848 | chr2 | 15568968 | 15580815 |  |
| supercont2.723 | 1 | 19176 | chr2 | 15580916 | 15600091 |  |
| supercont2.716 | 1 | 18997 | chr2 | 15600192 | 15619188 |  |
| supercont2.402 | 1 | 93657 | chr2 | 15619289 | 15712945 |  |
| supercont2.650 | 26812 | 1 | chr2 | 15713046 | 15739857 |  |
| supercont2.116 | 338725 | 1 | chr2 | 15739958 | 16078682 |  |
| supercont2.279 | 124405 | 1 | chr2 | 16078783 | 16203187 |  |
| supercont2.549 | 42073 | 1 | chr2 | 16203288 | 16245360 |  |
| supercont2.444 | 1 | 64692 | chr2 | 16245461 | 16310152 |  |
| supercont2.270 | 1 | 127796 | chr2 | 16310253 | 16438048 |  |
| supercont2.218 | 160390 | 1 | chr2 | 16438149 | 16598538 |  |
| supercont2.133 | 1 | 292427 | chr2 | 16598639 | 16891065 |  |
| supercont2.475 | 61787 | 1 | chr2 | 16891166 | 16952952 |  |
| supercont2.299 | 1 | 117140 | chr2 | 16953053 | 17070192 |  |
| supercont2.703 | 21457 | 1 | chr2 | 17070293 | 17091749 |  |
| supercont2.488 | 56113 | 1 | chr2 | 17091850 | 17147962 |  |
| supercont2.666 | 24340 | 1 | chr2 | 17148063 | 17172402 |  |
| supercont2.255 | 142408 | 1 | chr2 | 17172503 | 17314910 |  |
| supercont2.191 | 1 | 181846 | chr2 | 17315011 | 17496856 |  |
| supercont2.59 | 572070 | 1 | chr2 | 17496957 | 18069026 |  |
| supercont2.195 | 1 | 188369 | chr2 | 18069127 | 18257495 |  |
| supercont2.459 | 65305 | 1 | chr2 | 18257596 | 18322900 |  |
| supercont2.328 | 1 | 105815 | chr2 | 18323001 | 18428815 |  |
| supercont2.326 | 1 | 106454 | chr2 | 18428916 | 18535369 |  |
| supercont2.698 | 1 | 20850 | chr2 | 18535470 | 18556319 |  |
| supercont2.629 | 28728 | 1 | chr2 | 18556420 | 18585147 |  |
| supercont2.136 | 290657 | 1 | chr2 | 18585248 | 18875904 |  |
| supercont2.315 | 1 | 108140 | chr2 | 18876005 | 18984144 |  |
| supercont2.321 | 1 | 106981 | chr2 | 18984245 | 19091225 |  |
| supercont2.101 | 1 | 382529 | chr2 | 19091326 | 19473854 |  |
| supercont2.28 | 989246 | 1 | chr2 | 19473955 | 20463200 |  |
| supercont2.409 | 79321 | 1 | chr2 | 20463301 | 20542621 |  |
| supercont2.333 | 103811 | 1 | chr2 | 20542722 | 20646532 |  |
| supercont2.833 | 11475 | 1 | chr2 | 20646633 | 20658107 |  |
| supercont2.156 | 237104 | 1 | chr2 | 20658208 | 20895311 |  |
| supercont2.39 | 764304 | 1 | chr2 | 20895412 | 21659715 |  |
| supercont2.353 | 95315 | 1 | chr2 | 21659816 | 21755130 |  |
| supercont2.740 | 17111 | 1 | chr2 | 21755231 | 21772341 |  |
| supercont2.408 | 1 | 78841 | chr2 | 21772442 | 21851282 |  |
| supercont2.182 | 1 | 197584 | chr2 | 21851383 | 22048966 |  |
| supercont2.899 | 7414 | 1 | chr2 | 22049067 | 22056480 |  |
| supercont2.226 | 1 | 163146 | chr2 | 22056581 | 22219726 |  |
| supercont2.317 | 108154 | 1 | chr2 | 22219827 | 22327980 |  |
| supercont2.227 | 155230 | 1 | chr2 | 22328081 | 22483310 |  |
| supercont2.503 | 52714 | 1 | chr2 | 22483411 | 22536124 |  |
| supercont2.742 | 16316 | 1 | chr2 | 22536225 | 22552540 |  |
| supercont2.184 | 1 | 193648 | chr2 | 22552641 | 22746288 |  |
| supercont2.261 | 135913 | 1 | chr2 | 22746389 | 22882301 |  |
| supercont2.168 | 218317 | 1 | chr2 | 22882402 | 23100718 |  |
| supercont2.1320 | 1 | 1369 | chr2 | 23100819 | 23102187 |  |
| supercont2.308 | 1 | 113530 | chr2 | 23102288 | 23215817 |  |
| supercont2.251 | 1 | 139641 | chr2 | 23215918 | 23355558 |  |
| supercont2.316 | 108797 | 1 | chr2 | 23355659 | 23464455 |  |
| supercont2.859 | 1 | 8998 | chr2 | 23464556 | 23473553 |  |
| supercont2.106 | 365570 | 1 | chr2 | 23473654 | 23839223 |  |
| supercont2.188 | 1 | 189203 | chr2 | 23839324 | 24028526 |  |
| supercont2.1055 | 1 | 2669 | chr2 | 24028627 | 24031295 |  |
| supercont2.1029 | 1 | 3226 | chr2 | 24031396 | 24034621 |  |
| supercont2.1450 | 1 | 1114 | chr2 | 24034722 | 24035835 |  |
| supercont2.458 | 1 | 62583 | chr2 | 24035936 | 24098518 |  |
| supercont2.375 | 89554 | 1 | chr2 | 24098619 | 24188172 |  |
| supercont2.481 | 61394 | 1 | chr2 | 24188273 | 24249666 |  |
| supercont2.194 | 181098 | 1 | chr2 | 24249767 | 24430864 |  |
| supercont2.978 | 5761 | 1 | chr2 | 24430965 | 24436725 |  |
| supercont2.135 | 279200 | 152670 | chr2 | 24436826 | 24563356 | x |
| supercont2.90 | 400400 | 1 | chr2 | 24563457 | 24963856 |  |
| supercont2.60 | 550865 | 1 | chr2 | 24963957 | 25514821 |  |
| supercont2.292 | 117701 | 1 | chr3 | 1 | 117701 |  |
| supercont2.294 | 117270 | 1 | chr3 | 117802 | 235071 |  |
| supercont2.805 | 11941 | 1 | chr3 | 235172 | 247112 |  |
| supercont2.897 | 7439 | 1 | chr3 | 247213 | 254651 |  |
| supercont2.422 | 1 | 72704 | chr3 | 254752 | 327455 |  |
| supercont2.6 | 1 | 2488093 | chr3 | 327556 | 2815648 |  |
| supercont2.11 | 1983256 | 1 | chr3 | 2815749 | 4799004 |  |
| supercont2.111 | 1 | 348969 | chr3 | 4799105 | 5148073 |  |
| supercont2.5 | 2526593 | 1 | chr3 | 5148174 | 7674766 |  |
| supercont2.47 | 663552 | 1 | chr3 | 7674867 | 8338418 |  |
| supercont2.82 | 1 | 436589 | chr3 | 8338519 | 8775107 |  |
| supercont2.117 | 332429 | 21288 | chr3 | 8775208 | 9086349 | x |
| supercont2.43 | 1 | 693068 | chr3 | 9086450 | 9779517 |  |
| supercont2.62 | 1 | 545918 | chr3 | 9779618 | 10325535 |  |
| supercont2.128 | 291672 | 1 | chr3 | 10325636 | 10617307 |  |
| supercont2.300 | 1 | 115185 | chr3 | 10617408 | 10732592 |  |
| supercont2.77 | 465625 | 1 | chr3 | 10732693 | 11198317 |  |
| supercont2.30 | 916602 | 876038 | chr3 | 11198418 | 11238982 | x |
| supercont2.79 | 469590 | 1 | chr3 | 11239083 | 11708672 |  |
| supercont2.613 | 31575 | 1 | chr3 | 11708773 | 11740347 |  |
| supercont2.757 | 1 | 15190 | chr3 | 11740448 | 11755637 |  |
| supercont2.147 | 1 | 255369 | chr3 | 11755738 | 12011106 |  |
| supercont2.311 | 109772 | 1 | chr3 | 12011207 | 12120978 |  |
| supercont2.196 | 1 | 173763 | chr3 | 12121079 | 12294841 |  |
| supercont2.441 | 1 | 67864 | chr3 | 12294942 | 12362805 |  |
| supercont2.262 | 1 | 134683 | chr3 | 12362906 | 12497588 |  |
| supercont2.673 | 23727 | 1 | chr3 | 12497689 | 12521415 |  |
| supercont2.640 | 28354 | 1 | chr3 | 12521516 | 12549869 |  |
| supercont2.765 | 1 | 14951 | chr3 | 12549970 | 12564920 |  |
| supercont2.914 | 6891 | 1 | chr3 | 12565021 | 12571911 |  |
| supercont2.901 | 1 | 7327 | chr3 | 12572012 | 12579338 |  |
| supercont2.683 | 22501 | 1 | chr3 | 12579439 | 12601939 |  |
| supercont2.530 | 45981 | 1 | chr3 | 12602040 | 12648020 |  |
| supercont2.181 | 1 | 191530 | chr3 | 12648121 | 12839650 |  |
| supercont2.633 | 1 | 38017 | chr3 | 12839751 | 12877767 |  |
| supercont2.923 | 6406 | 1 | chr3 | 12877868 | 12884273 |  |
| supercont2.824 | 10544 | 1 | chr3 | 12884374 | 12894917 |  |
| supercont2.426 | 72333 | 1 | chr3 | 12895018 | 12967350 |  |
| supercont2.745 | 15895 | 1 | chr3 | 12967451 | 12983345 |  |
| supercont2.573 | 39387 | 1 | chr3 | 12983446 | 13022832 |  |
| supercont2.576 | 1 | 36889 | chr3 | 13022933 | 13059821 |  |
| supercont2.215 | 1 | 162046 | chr3 | 13059922 | 13221967 |  |
| supercont2.557 | 1 | 39881 | chr3 | 13222068 | 13261948 |  |
| supercont2.729 | 1 | 17394 | chr3 | 13262049 | 13279442 |  |
| supercont2.786 | 1 | 13105 | chr3 | 13279543 | 13292647 |  |
| supercont2.379 | 87032 | 1 | chr3 | 13292748 | 13379779 |  |
| supercont2.542 | 43376 | 1 | chr3 | 13379880 | 13423255 |  |
| supercont2.808 | 1 | 11537 | chr3 | 13423356 | 13434892 |  |
| supercont2.749 | 15784 | 1 | chr3 | 13434993 | 13450776 |  |
| supercont2.667 | 24483 | 1 | chr3 | 13450877 | 13475359 |  |
| supercont2.438 | 1 | 71741 | chr3 | 13475460 | 13547200 |  |
| supercont2.1005 | 1 | 4787 | chr3 | 13547301 | 13552087 |  |
| supercont2.513 | 55909 | 1 | chr3 | 13552188 | 13608096 |  |
| supercont2.413 | 75207 | 1 | chr3 | 13608197 | 13683403 |  |
| supercont2.154 | 249939 | 1 | chr3 | 13683504 | 13933442 |  |
| supercont2.611 | 1 | 31515 | chr3 | 13933543 | 13965057 |  |
| supercont2.676 | 23554 | 1 | chr3 | 13965158 | 13988711 |  |
| supercont2.545 | 42272 | 1 | chr3 | 13988812 | 14031083 |  |
| supercont2.618 | 1 | 31010 | chr3 | 14031184 | 14062193 |  |
| supercont2.13 | 1 | 110117 | chr3 | 14062294 | 14172410 | x |
| supercont2.687 | 1 | 21943 | chr3 | 14172511 | 14194453 |  |
| supercont2.562 | 1 | 40250 | chr3 | 14194554 | 14234803 |  |
| supercont2.487 | 1 | 65576 | chr3 | 14234904 | 14300479 |  |
| supercont2.550 | 1 | 41068 | chr3 | 14300580 | 14341647 |  |
| supercont2.792 | 1 | 14558 | chr3 | 14341748 | 14356305 |  |
| supercont2.511 | 51185 | 1 | chr3 | 14356406 | 14407590 |  |
| supercont2.476 | 63526 | 1 | chr3 | 14407691 | 14471216 |  |
| supercont2.701 | 1 | 22648 | chr3 | 14471317 | 14493964 |  |
| supercont2.795 | 1 | 12775 | chr3 | 14494065 | 14506839 |  |
| supercont2.519 | 1 | 48857 | chr3 | 14506940 | 14555796 |  |
| supercont2.635 | 28309 | 1 | chr3 | 14555897 | 14584205 |  |
| supercont2.1150 | 1872 | 1 | chr3 | 14584306 | 14586177 |  |
| supercont2.620 | 31175 | 1 | chr3 | 14586278 | 14617452 |  |
| supercont2.717 | 18708 | 1 | chr3 | 14617553 | 14636260 |  |
| supercont2.541 | 44108 | 1 | chr3 | 14636361 | 14680468 |  |
| supercont2.499 | 1 | 53796 | chr3 | 14680569 | 14734364 |  |
| supercont2.243 | 1 | 145671 | chr3 | 14734465 | 14880135 |  |
| supercont2.307 | 19998 | 1 | chr3 | 14880236 | 14900233 | x |
| supercont2.335 | 1 | 17061 | chr3 | 14900334 | 14917394 | x |
| supercont2.520 | 48254 | 1 | chr3 | 14917495 | 14965748 |  |
| supercont2.693 | 21966 | 1 | chr3 | 14965849 | 14987814 |  |
| supercont2.138 | 1 | 276038 | chr3 | 14987915 | 15263952 |  |
| supercont2.474 | 58899 | 1 | chr3 | 15264053 | 15322951 |  |
| supercont2.732 | 17151 | 1 | chr3 | 15323052 | 15340202 |  |
| supercont2.425 | 1 | 72497 | chr3 | 15340303 | 15412799 |  |
| supercont2.428 | 69557 | 1 | chr3 | 15412900 | 15482456 |  |
| supercont2.453 | 1 | 63934 | chr3 | 15482557 | 15546490 |  |
| supercont2.753 | 1 | 16012 | chr3 | 15546591 | 15562602 |  |
| supercont2.355 | 1 | 95518 | chr3 | 15562703 | 15658220 |  |
| supercont2.489 | 68517 | 1 | chr3 | 15658321 | 15726837 |  |
| supercont2.718 | 18425 | 1 | chr3 | 15726938 | 15745362 |  |
| supercont2.405 | 77738 | 1 | chr3 | 15745463 | 15823200 |  |
| supercont2.838 | 9998 | 1 | chr3 | 15823301 | 15833298 |  |
| supercont2.615 | 32082 | 1 | chr3 | 15833399 | 15865480 |  |
| supercont2.655 | 1 | 25691 | chr3 | 15865581 | 15891271 |  |
| supercont2.847 | 1 | 16555 | chr3 | 15891372 | 15907926 |  |
| supercont2.265 | 135660 | 1 | chr3 | 15908027 | 16043686 |  |
| supercont2.995 | 1 | 4446 | chr3 | 16043787 | 16048232 |  |
| supercont2.263 | 1 | 138363 | chr3 | 16048333 | 16186695 |  |
| supercont2.670 | 24180 | 1 | chr3 | 16186796 | 16210975 |  |
| supercont2.671 | 1 | 41867 | chr3 | 16211076 | 16252942 |  |
| supercont2.1457 | 1 | 1089 | chr3 | 16253043 | 16254131 |  |
| supercont2.947 | 7015 | 1 | chr3 | 16254232 | 16261246 |  |
| supercont2.680 | 1 | 23488 | chr3 | 16261347 | 16284834 |  |
| supercont2.141 | 274671 | 1 | chr3 | 16284935 | 16559605 |  |
| supercont2.571 | 1 | 37360 | chr3 | 16559706 | 16597065 |  |
| supercont2.721 | 19353 | 1 | chr3 | 16597166 | 16616518 |  |
| supercont2.114 | 347298 | 1 | chr3 | 16616619 | 16963916 |  |
| supercont2.98 | 388897 | 1 | chr3 | 16964017 | 17352913 |  |
| supercont2.213 | 50069 | 1 | chr3 | 17353014 | 17403082 | x |
| supercont2.700 | 1 | 20650 | chr3 | 17403183 | 17423832 |  |
| supercont2.100 | 1 | 387666 | chr3 | 17423933 | 17811598 |  |
| supercont2.376 | 92266 | 1 | chr3 | 17811699 | 17903964 |  |
| supercont2.87 | 412915 | 1 | chr3 | 17904065 | 18316979 |  |
| supercont2.681 | 22824 | 1 | chr3 | 18317080 | 18339903 |  |
| supercont2.335 | 33721 | 102538 | chr3 | 18340004 | 18408821 | x |
| supercont2.473 | 1 | 60001 | chr3 | 18408922 | 18468922 |  |
| supercont2.307 | 113507 | 33735 | chr3 | 18469023 | 18548795 | x |
| supercont2.241 | 144345 | 1 | chr3 | 18548896 | 18693240 |  |
| supercont2.651 | 26581 | 1 | chr3 | 18693341 | 18719921 |  |
| supercont2.431 | 1 | 69506 | chr3 | 18720022 | 18789527 |  |
| supercont2.124 | 321101 | 1 | chr3 | 18789628 | 19110728 |  |
| supercont2.285 | 1 | 122897 | chr3 | 19110829 | 19233725 |  |
| supercont2.626 | 29450 | 1 | chr3 | 19233826 | 19263275 |  |
| supercont2.512 | 1 | 54902 | chr3 | 19263376 | 19318277 |  |
| supercont2.410 | 81463 | 1 | chr3 | 19318378 | 19399840 |  |
| supercont2.446 | 64752 | 1 | chr3 | 19399941 | 19464692 |  |
| supercont2.608 | 1 | 32469 | chr3 | 19464793 | 19497261 |  |
| supercont2.319 | 106722 | 1 | chr3 | 19497362 | 19604083 |  |
| supercont2.203 | 182719 | 1 | chr3 | 19604184 | 19786902 |  |
| supercont2.420 | 1 | 73320 | chr3 | 19787003 | 19860322 |  |
| supercont2.281 | 125879 | 1 | chr3 | 19860423 | 19986301 |  |
| supercont2.81 | 450431 | 1 | chr3 | 19986402 | 20436832 |  |
| supercont2.110 | 351230 | 1 | chr3 | 20436933 | 20788162 |  |
| supercont2.224 | 167045 | 1 | chr3 | 20788263 | 20955307 |  |
| supercont2.477 | 62314 | 1 | chr3 | 20955408 | 21017721 |  |
| supercont2.18 | 1 | 1262229 | chr3 | 21017822 | 22280050 |  |
| supercont2.56 | 551169 | 1 | chr3 | 22280151 | 22831319 | x |
| supercont2.53 | 597665 | 185208 | chr3 | 22831420 | 23243877 | x |
| supercont2.4 | 1 | 2639930 | chr3 | 23243978 | 25883907 |  |
| supercont2.648 | 26679 | 1 | chr3 | 25884008 | 25910686 |  |
| supercont2.301 | 115590 | 1 | chr3 | 25910787 | 26026376 |  |
| supercont2.162 | 1 | 222766 | chr3 | 26026477 | 26249242 |  |
| supercont2.289 | 1 | 118917 | chr3 | 26249343 | 26368259 |  |
| supercont2.16 | 270920 | 1328748 | chr3 | 26368360 | 27426188 | x |
| supercont2.93 | 386522 | 1 | chr3 | 27426289 | 27812810 |  |
| supercont2.37 | 771671 | 1 | chr3 | 27812911 | 28584581 |  |
| supercont2.131 | 1 | 22885 | chr3 | 28584682 | 28607566 | x |
| supercont2.71 | 1 | 488409 | chr3 | 28607667 | 29096075 |  |
| supercont2.24 | 1 | 1018835 | chr3 | 29096176 | 30115010 |  |
| supercont2.552 | 1 | 40855 | chr3 | 30115111 | 30155965 |  |
| supercont2.123 | 1 | 316944 | chr3 | 30156066 | 30473009 |  |
| supercont2.873 | 8430 | 1 | chr3 | 30473110 | 30481539 |  |
| supercont2.32 | 1 | 894853 | chr3 | 30481640 | 31376492 |  |
| supercont2.248 | 1 | 142508 | chr3 | 31376593 | 31519100 |  |
| supercont2.471 | 1 | 61167 | chr4 | 1 | 61167 |  |
| supercont2.663 | 25035 | 1 | chr4 | 61268 | 86302 |  |
| supercont2.56 | 581391 | 571971 | chr4 | 86403 | 95823 | x |
| supercont2.352 | 108108 | 1 | chr4 | 95924 | 204031 |  |
| supercont2.233 | 151978 | 1 | chr4 | 204132 | 356109 |  |
| supercont2.249 | 142671 | 1 | chr4 | 356210 | 498880 |  |
| supercont2.276 | 1 | 127932 | chr4 | 498981 | 626912 |  |
| supercont2.269 | 132202 | 1 | chr4 | 627013 | 759214 |  |
| supercont2.738 | 16685 | 1 | chr4 | 759315 | 775999 |  |
| supercont2.86 | 423674 | 1 | chr4 | 776100 | 1199773 |  |
| supercont2.416 | 75370 | 1 | chr4 | 1199874 | 1275243 |  |
| supercont2.267 | 1 | 133600 | chr4 | 1275344 | 1408943 |  |
| supercont2.617 | 33390 | 1 | chr4 | 1409044 | 1442433 |  |
| supercont2.155 | 1 | 247183 | chr4 | 1442534 | 1689716 |  |
| supercont2.325 | 1 | 111374 | chr4 | 1689817 | 1801190 |  |
| supercont2.204 | 1 | 170397 | chr4 | 1801291 | 1971687 |  |
| supercont2.135 | 1 | 137134 | chr4 | 1971788 | 2108921 | x |
| supercont2.153 | 1 | 247471 | chr4 | 2109022 | 2356492 |  |
| supercont2.192 | 1 | 177909 | chr4 | 2356593 | 2534501 |  |
| supercont2.96 | 1 | 383679 | chr4 | 2534602 | 2918280 |  |
| supercont2.36 | 1 | 817370 | chr4 | 2918381 | 3735750 |  |
| supercont2.51 | 615989 | 1 | chr4 | 3735851 | 4351839 |  |
| supercont2.35 | 817648 | 1 | chr4 | 4351940 | 5169587 |  |
| supercont2.403 | 1 | 79457 | chr4 | 5169688 | 5249144 |  |
| supercont2.831 | 1 | 10250 | chr4 | 5249245 | 5259494 |  |
| supercont2.127 | 1 | 291938 | chr4 | 5259595 | 5551532 |  |
| supercont2.139 | 275319 | 1 | chr4 | 5551633 | 5826951 |  |
| supercont2.1 | 3543150 | 1 | chr4 | 5827052 | 9370201 |  |
| supercont2.865 | 8762 | 1 | chr4 | 9370302 | 9379063 |  |
| supercont2.40 | 1 | 747119 | chr4 | 9379164 | 10126282 |  |
| supercont2.50 | 1 | 638533 | chr4 | 10126383 | 10764915 |  |
| supercont2.140 | 1 | 282375 | chr4 | 10765016 | 11047390 |  |
| supercont2.449 | 63442 | 1 | chr4 | 11047491 | 11110932 |  |
| supercont2.437 | 66326 | 1 | chr4 | 11111033 | 11177358 |  |
| supercont2.322 | 123982 | 67495 | chr4 | 11177459 | 11233946 | x |
| supercont2.531 | 1 | 45377 | chr4 | 11234047 | 11279423 |  |
| supercont2.310 | 1 | 110525 | chr4 | 11279524 | 11390048 |  |
| supercont2.466 | 1 | 61506 | chr4 | 11390149 | 11451654 |  |
| supercont2.391 | 1 | 81363 | chr4 | 11451755 | 11533117 |  |
| supercont2.406 | 78035 | 1 | chr4 | 11533218 | 11611252 |  |
| supercont2.339 | 101556 | 1 | chr4 | 11611353 | 11712908 |  |
| supercont2.331 | 1 | 104264 | chr4 | 11713009 | 11817272 |  |
| supercont2.501 | 59219 | 1 | chr4 | 11817373 | 11876591 |  |
| supercont2.643 | 26929 | 1 | chr4 | 11876692 | 11903620 |  |
| supercont2.627 | 1 | 36274 | chr4 | 11903721 | 11939994 |  |
| supercont2.559 | 1 | 39718 | chr4 | 11940095 | 11979812 |  |
| supercont2.743 | 1 | 16124 | chr4 | 11979913 | 11996036 |  |
| supercont2.258 | 1 | 137697 | chr4 | 11996137 | 12133833 |  |
| supercont2.657 | 1 | 25704 | chr4 | 12133934 | 12159637 |  |
| supercont2.711 | 1 | 19289 | chr4 | 12159738 | 12179026 |  |
| supercont2.567 | 37907 | 1 | chr4 | 12179127 | 12217033 |  |
| supercont2.173 | 1 | 208008 | chr4 | 12217134 | 12425141 |  |
| supercont2.682 | 1 | 22630 | chr4 | 12425242 | 12447871 |  |
| supercont2.442 | 1 | 66080 | chr4 | 12447972 | 12514051 |  |
| supercont2.945 | 1 | 5903 | chr4 | 12514152 | 12520054 |  |
| supercont2.709 | 21659 | 1 | chr4 | 12520155 | 12541813 |  |
| supercont2.813 | 1 | 13446 | chr4 | 12541914 | 12555359 |  |
| supercont2.555 | 46297 | 34271 | chr4 | 12555460 | 12567486 | x |
| supercont2.774 | 14174 | 1 | chr4 | 12567587 | 12581760 |  |
| supercont2.374 | 88684 | 1 | chr4 | 12581861 | 12670544 |  |
| supercont2.320 | 1 | 107192 | chr4 | 12670645 | 12777836 |  |
| supercont2.450 | 1 | 63234 | chr4 | 12777937 | 12841170 |  |
| supercont2.332 | 1 | 103317 | chr4 | 12841271 | 12944587 |  |
| supercont2.160 | 235934 | 1 | chr4 | 12944688 | 13180621 |  |
| supercont2.436 | 1 | 67291 | chr4 | 13180722 | 13248012 |  |
| supercont2.235 | 1 | 153654 | chr4 | 13248113 | 13401766 |  |
| supercont2.288 | 1 | 119704 | chr4 | 13401867 | 13521570 |  |
| supercont2.568 | 37883 | 1 | chr4 | 13521671 | 13559553 |  |
| supercont2.344 | 107307 | 1 | chr4 | 13559654 | 13666960 |  |
| supercont2.159 | 1 | 230795 | chr4 | 13667061 | 13897855 |  |
| supercont2.702 | 1 | 20804 | chr4 | 13897956 | 13918759 |  |
| supercont2.452 | 63973 | 1 | chr4 | 13918860 | 13982832 |  |
| supercont2.639 | 27925 | 1 | chr4 | 13982933 | 14010857 |  |
| supercont2.540 | 1 | 49383 | chr4 | 14010958 | 14060340 |  |
| supercont2.220 | 1 | 166191 | chr4 | 14060441 | 14226631 |  |
| supercont2.644 | 27061 | 1 | chr4 | 14226732 | 14253792 |  |
| supercont2.398 | 62418 | 86345 | chr4 | 14253893 | 14277820 | x |
| supercont2.584 | 37498 | 1 | chr4 | 14277921 | 14315418 |  |
| supercont2.515 | 1 | 58217 | chr4 | 14315519 | 14373735 |  |
| supercont2.393 | 1 | 82398 | chr4 | 14373836 | 14456233 |  |
| supercont2.527 | 1 | 47539 | chr4 | 14456334 | 14503872 |  |
| supercont2.180 | 200058 | 1 | chr4 | 14503973 | 14704030 |  |
| supercont2.751 | 15715 | 1 | chr4 | 14704131 | 14719845 |  |
| supercont2.605 | 32908 | 1 | chr4 | 14719946 | 14752853 |  |
| supercont2.282 | 124641 | 1 | chr4 | 14752954 | 14877594 |  |
| supercont2.362 | 1 | 99167 | chr4 | 14877695 | 14976861 |  |
| supercont2.858 | 9013 | 1 | chr4 | 14976962 | 14985974 |  |
| supercont2.231 | 1 | 168121 | chr4 | 14986075 | 15154195 |  |
| supercont2.198 | 1 | 173312 | chr4 | 15154296 | 15327607 |  |
| supercont2.581 | 36546 | 1 | chr4 | 15327708 | 15364253 |  |
| supercont2.389 | 1 | 84380 | chr4 | 15364354 | 15448733 |  |
| supercont2.346 | 1 | 107012 | chr4 | 15448834 | 15555845 |  |
| supercont2.534 | 1 | 44752 | chr4 | 15555946 | 15600697 |  |
| supercont2.563 | 1 | 38938 | chr4 | 15600798 | 15639735 |  |
| supercont2.504 | 1 | 54000 | chr4 | 15639836 | 15693835 |  |
| supercont2.174 | 1 | 206935 | chr4 | 15693936 | 15900870 |  |
| supercont2.368 | 1 | 90994 | chr4 | 15900971 | 15991964 |  |
| supercont2.849 | 9266 | 1 | chr4 | 15992065 | 16001330 |  |
| supercont2.555 | 1 | 32602 | chr4 | 16001431 | 16034032 | x |
| supercont2.578 | 1 | 37162 | chr4 | 16034133 | 16071294 |  |
| supercont2.423 | 72017 | 1 | chr4 | 16071395 | 16143411 |  |
| supercont2.361 | 1 | 95857 | chr4 | 16143512 | 16239368 |  |
| supercont2.456 | 1 | 67307 | chr4 | 16239469 | 16306775 |  |
| supercont2.238 | 151136 | 1 | chr4 | 16306876 | 16458011 |  |
| supercont2.118 | 1 | 333073 | chr4 | 16458112 | 16791184 |  |
| supercont2.595 | 1 | 34040 | chr4 | 16791285 | 16825324 |  |
| supercont2.537 | 1 | 44339 | chr4 | 16825425 | 16869763 |  |
| supercont2.280 | 137783 | 30253 | chr4 | 16869864 | 16977394 | x |
| supercont2.246 | 144071 | 1 | chr4 | 16977495 | 17121565 |  |
| supercont2.383 | 85433 | 1 | chr4 | 17121666 | 17207098 |  |
| supercont2.447 | 26492 | 64018 | chr4 | 17207199 | 17244725 | x |
| supercont2.455 | 1 | 65282 | chr4 | 17244826 | 17310107 |  |
| supercont2.386 | 1 | 83445 | chr4 | 17310208 | 17393652 |  |
| supercont2.208 | 165610 | 1 | chr4 | 17393753 | 17559362 |  |
| supercont2.364 | 1 | 92454 | chr4 | 17559463 | 17651916 |  |
| supercont2.762 | 1 | 15055 | chr4 | 17652017 | 17667071 |  |
| supercont2.526 | 50149 | 1 | chr4 | 17667172 | 17717320 |  |
| supercont2.334 | 1 | 104921 | chr4 | 17717421 | 17822341 |  |
| supercont2.268 | 133121 | 1 | chr4 | 17822442 | 17955562 |  |
| supercont2.275 | 1 | 126539 | chr4 | 17955663 | 18082201 |  |
| supercont2.517 | 1 | 52582 | chr4 | 18082302 | 18134883 |  |
| supercont2.369 | 1 | 90191 | chr4 | 18134984 | 18225174 |  |
| supercont2.569 | 1 | 38350 | chr4 | 18225275 | 18263624 |  |
| supercont2.937 | 6072 | 1 | chr4 | 18263725 | 18269796 |  |
| supercont2.143 | 1 | 276454 | chr4 | 18269897 | 18546350 |  |
| supercont2.621 | 1 | 30215 | chr4 | 18546451 | 18576665 |  |
| supercont2.585 | 1 | 34931 | chr4 | 18576766 | 18611696 |  |
| supercont2.566 | 1 | 39040 | chr4 | 18611797 | 18650836 |  |
| supercont2.131 | 63832 | 29599 | chr4 | 18650937 | 18685170 | x |
| supercont2.461 | 63145 | 1 | chr4 | 18685271 | 18748415 |  |
| supercont2.447 | 20504 | 1 | chr4 | 18748516 | 18769019 | x |
| supercont2.125 | 299695 | 1 | chr4 | 18769120 | 19068814 |  |
| supercont2.371 | 1 | 93859 | chr4 | 19068915 | 19162773 |  |
| supercont2.674 | 23631 | 1 | chr4 | 19162874 | 19186504 |  |
| supercont2.337 | 1 | 101506 | chr4 | 19186605 | 19288110 |  |
| supercont2.273 | 128200 | 1 | chr4 | 19288211 | 19416410 |  |
| supercont2.121 | 1 | 319872 | chr4 | 19416511 | 19736382 |  |
| supercont2.404 | 1 | 77556 | chr4 | 19736483 | 19814038 |  |
| supercont2.89 | 1 | 400604 | chr4 | 19814139 | 20214742 |  |
| supercont2.137 | 283424 | 1 | chr4 | 20214843 | 20498266 |  |
| supercont2.388 | 1 | 82864 | chr4 | 20498367 | 20581230 |  |
| supercont2.287 | 121475 | 1 | chr4 | 20581331 | 20702805 |  |
| supercont2.445 | 1 | 65584 | chr4 | 20702906 | 20768489 |  |
| supercont2.144 | 269096 | 1 | chr4 | 20768590 | 21037685 |  |
| supercont2.54 | 1 | 595342 | chr4 | 21037786 | 21633127 |  |
| supercont2.15 | 1427049 | 1 | chr4 | 21633228 | 23060276 |  |
| supercont2.12 | 1746771 | 1 | chr4 | 23060377 | 24807147 |  |
| supercont2.807 | 11628 | 1 | chr4 | 24807248 | 24818875 |  |
| supercont2.7 | 1 | 2405115 | chr4 | 24818976 | 27224090 |  |
| supercont2.894 | 1 | 8094 | chr4 | 27224191 | 27232284 |  |
| supercont2.27 | 1 | 959775 | chr4 | 27232385 | 28192159 |  |
| supercont2.211 | 165427 | 1 | chr4 | 28192260 | 28357686 |  |
| supercont2.75 | 1 | 474735 | chr4 | 28357787 | 28832521 |  |
| supercont2.20 | 1156447 | 1 | chr4 | 28832622 | 29989068 |  |
| supercont2.13 | 909947 | 1694315 | chr4 | 29989169 | 30773537 | x |
| supercont2.885 | 7914 | 1 | chr4 | 30773638 | 30781551 |  |
| supercont2.592 | 34158 | 1 | chr4 | 30781652 | 30815809 |  |
| supercont2.129 | 1 | 296070 | chr4 | 30815910 | 31111979 |  |
| supercont2.66 | 507097 | 1 | chr4 | 31112080 | 31619176 |  |
| supercont2.341 | 1 | 103290 | chr4 | 31619277 | 31722566 |  |
| supercont2.669 | 1 | 24381 | chr5 | 1 | 24381 |  |
| supercont2.179 | 1 | 194338 | chr5 | 24482 | 218819 |  |
| supercont2.73 | 495848 | 1 | chr5 | 218920 | 714767 |  |
| supercont2.387 | 1 | 83521 | chr5 | 714868 | 798388 |  |
| supercont2.454 | 1 | 64309 | chr5 | 798489 | 862797 |  |
| supercont2.575 | 36749 | 1 | chr5 | 862898 | 899646 |  |
| supercont2.399 | 79021 | 1 | chr5 | 899747 | 978767 |  |
| supercont2.363 | 93762 | 1 | chr5 | 978868 | 1072629 |  |
| supercont2.394 | 1 | 81727 | chr5 | 1072730 | 1154456 |  |
| supercont2.83 | 1 | 440415 | chr5 | 1154557 | 1594971 |  |
| supercont2.49 | 660388 | 1 | chr5 | 1595072 | 2255459 |  |
| supercont2.200 | 171496 | 1 | chr5 | 2255560 | 2427055 |  |
| supercont2.706 | 19874 | 1 | chr5 | 2427156 | 2447029 |  |
| supercont2.443 | 70682 | 1 | chr5 | 2447130 | 2517811 |  |
| supercont2.78 | 460173 | 1 | chr5 | 2517912 | 2978084 |  |
| supercont2.647 | 26621 | 1 | chr5 | 2978185 | 3004805 |  |
| supercont2.22 | 1020721 | 1 | chr5 | 3004906 | 4025626 |  |
| supercont2.589 | 35853 | 1 | chr5 | 4025727 | 4061579 |  |
| supercont2.242 | 144797 | 1 | chr5 | 4061680 | 4206476 |  |
| supercont2.25 | 1005823 | 1 | chr5 | 4206577 | 5212399 |  |
| supercont2.38 | 1 | 749966 | chr5 | 5212500 | 5962465 |  |
| supercont2.61 | 549444 | 1 | chr5 | 5962566 | 6512009 |  |
| supercont2.63 | 1 | 532934 | chr5 | 6512110 | 7045043 |  |
| supercont2.21 | 1 | 1056769 | chr5 | 7045144 | 8101912 |  |
| supercont2.74 | 1 | 487639 | chr5 | 8102013 | 8589651 |  |
| supercont2.107 | 1 | 372518 | chr5 | 8589752 | 8962269 |  |
| supercont2.959 | 1 | 5459 | chr5 | 8962370 | 8967828 |  |
| supercont2.187 | 195024 | 1 | chr5 | 8967929 | 9162952 |  |
| supercont2.468 | 1 | 62692 | chr5 | 9163053 | 9225744 |  |
| supercont2.378 | 1 | 91589 | chr5 | 9225845 | 9317433 |  |
| supercont2.254 | 1 | 141788 | chr5 | 9317534 | 9459321 |  |
| supercont2.493 | 55082 | 1 | chr5 | 9459422 | 9514503 |  |
| supercont2.223 | 1 | 160805 | chr5 | 9514604 | 9675408 |  |
| supercont2.304 | 112693 | 1 | chr5 | 9675509 | 9788201 |  |
| supercont2.397 | 1 | 81221 | chr5 | 9788302 | 9869522 |  |
| supercont2.185 | 197910 | 1 | chr5 | 9869623 | 10067532 |  |
| supercont2.134 | 282191 | 1 | chr5 | 10067633 | 10349823 |  |
| supercont2.350 | 97654 | 1 | chr5 | 10349924 | 10447577 |  |
| supercont2.574 | 1 | 37268 | chr5 | 10447678 | 10484945 |  |
| supercont2.380 | 89104 | 1 | chr5 | 10485046 | 10574149 |  |
| supercont2.864 | 8733 | 1 | chr5 | 10574250 | 10582982 |  |
| supercont2.354 | 1 | 95307 | chr5 | 10583083 | 10678389 |  |
| supercont2.172 | 208659 | 1 | chr5 | 10678490 | 10887148 |  |
| supercont2.157 | 232354 | 1 | chr5 | 10887249 | 11119602 |  |
| supercont2.599 | 33229 | 1 | chr5 | 11119703 | 11152931 |  |
| supercont2.348 | 100699 | 1 | chr5 | 11153032 | 11253730 |  |
| supercont2.342 | 1 | 101619 | chr5 | 11253831 | 11355449 |  |
| supercont2.797 | 1 | 13099 | chr5 | 11355550 | 11368648 |  |
| supercont2.730 | 1 | 17462 | chr5 | 11368749 | 11386210 |  |
| supercont2.340 | 102746 | 1 | chr5 | 11386311 | 11489056 |  |
| supercont2.318 | 107561 | 1 | chr5 | 11489157 | 11596717 |  |
| supercont2.790 | 13512 | 1 | chr5 | 11596818 | 11610329 |  |
| supercont2.370 | 90118 | 1 | chr5 | 11610430 | 11700547 |  |
| supercont2.556 | 1 | 39904 | chr5 | 11700648 | 11740551 |  |
| supercont2.230 | 1 | 157709 | chr5 | 11740652 | 11898360 |  |
| supercont2.199 | 1 | 178391 | chr5 | 11898461 | 12076851 |  |
| supercont2.508 | 52229 | 1 | chr5 | 12076952 | 12129180 |  |
| supercont2.955 | 5545 | 1 | chr5 | 12129281 | 12134825 |  |
| supercont2.544 | 1 | 42852 | chr5 | 12134926 | 12177777 |  |
| supercont2.518 | 1 | 58531 | chr5 | 12177878 | 12236408 |  |
| supercont2.484 | 1 | 58269 | chr5 | 12236509 | 12294777 |  |
| supercont2.590 | 1 | 41676 | chr5 | 12294878 | 12336553 |  |
| supercont2.451 | 1 | 63720 | chr5 | 12336654 | 12400373 |  |
| supercont2.755 | 1 | 15283 | chr5 | 12400474 | 12415756 |  |
| supercont2.324 | 1 | 107114 | chr5 | 12415857 | 12522970 |  |
| supercont2.496 | 1 | 54584 | chr5 | 12523071 | 12577654 |  |
| supercont2.470 | 75888 | 1 | chr5 | 12577755 | 12653642 |  |
| supercont2.169 | 1 | 215319 | chr5 | 12653743 | 12869061 |  |
| supercont2.42 | 1 | 410652 | chr5 | 12869162 | 13279813 | x |
| supercont2.345 | 97470 | 1 | chr5 | 13279914 | 13377383 |  |
| supercont2.943 | 1 | 5973 | chr5 | 13377484 | 13383456 |  |
| supercont2.382 | 1 | 85054 | chr5 | 13383557 | 13468610 |  |
| supercont2.480 | 57893 | 1 | chr5 | 13468711 | 13526603 |  |
| supercont2.88 | 1 | 406804 | chr5 | 13526704 | 13933507 |  |
| supercont2.516 | 1 | 49381 | chr5 | 13933608 | 13982988 |  |
| supercont2.432 | 1 | 69871 | chr5 | 13983089 | 14052959 |  |
| supercont2.170 | 211882 | 1 | chr5 | 14053060 | 14264941 |  |
| supercont2.427 | 72255 | 1 | chr5 | 14265042 | 14337296 |  |
| supercont2.768 | 1 | 14545 | chr5 | 14337397 | 14351941 |  |
| supercont2.659 | 1 | 25154 | chr5 | 14352042 | 14377195 |  |
| supercont2.239 | 150329 | 1 | chr5 | 14377296 | 14527624 |  |
| supercont2.763 | 1 | 14959 | chr5 | 14527725 | 14542683 |  |
| supercont2.685 | 1 | 22742 | chr5 | 14542784 | 14565525 |  |
| supercont2.151 | 247235 | 1 | chr5 | 14565626 | 14812860 |  |
| supercont2.236 | 152229 | 1 | chr5 | 14812961 | 14965189 |  |
| supercont2.293 | 120040 | 1 | chr5 | 14965290 | 15085329 |  |
| supercont2.55 | 588576 | 1 | chr5 | 15085430 | 15674005 |  |
| supercont2.398 | 1 | 58653 | chr5 | 15674106 | 15732758 | x |
| supercont2.104 | 383438 | 1 | chr5 | 15732859 | 16116296 |  |
| supercont2.809 | 1 | 11474 | chr5 | 16116397 | 16127870 |  |
| supercont2.115 | 341823 | 1 | chr5 | 16127971 | 16469793 |  |
| supercont2.237 | 1 | 150481 | chr5 | 16469894 | 16620374 |  |
| supercont2.625 | 1 | 29623 | chr5 | 16620475 | 16650097 |  |
| supercont2.424 | 85383 | 1 | chr5 | 16650198 | 16735580 |  |
| supercont2.146 | 266016 | 1 | chr5 | 16735681 | 17001696 |  |
| supercont2.843 | 9610 | 1 | chr5 | 17001797 | 17011406 |  |
| supercont2.604 | 1 | 35283 | chr5 | 17011507 | 17046789 |  |
| supercont2.558 | 1 | 39727 | chr5 | 17046890 | 17086616 |  |
| supercont2.94 | 385758 | 1 | chr5 | 17086717 | 17472474 |  |
| supercont2.323 | 1 | 106665 | chr5 | 17472575 | 17579239 |  |
| supercont2.684 | 1 | 22487 | chr5 | 17579340 | 17601826 |  |
| supercont2.297 | 1 | 115852 | chr5 | 17601927 | 17717778 |  |
| supercont2.485 | 56160 | 1 | chr5 | 17717879 | 17774038 |  |
| supercont2.603 | 33114 | 1 | chr5 | 17774139 | 17807252 |  |
| supercont2.131 | 89626 | 292733 | chr5 | 17807353 | 18010460 | x |
| supercont2.412 | 75929 | 1 | chr5 | 18010561 | 18086489 |  |
| supercont2.306 | 111454 | 1 | chr5 | 18086590 | 18198043 |  |
| supercont2.103 | 1 | 383389 | chr5 | 18198144 | 18581532 |  |
| supercont2.163 | 1 | 224539 | chr5 | 18581633 | 18806171 |  |
| supercont2.69 | 495202 | 1 | chr5 | 18806272 | 19301473 |  |
| supercont2.539 | 1 | 44805 | chr5 | 19301574 | 19346378 |  |
| supercont2.407 | 1 | 77355 | chr5 | 19346479 | 19423833 |  |
| supercont2.217 | 1 | 162663 | chr5 | 19423934 | 19586596 |  |
| supercont2.862 | 1 | 8895 | chr5 | 19586697 | 19595591 |  |
| supercont2.183 | 1 | 192070 | chr5 | 19595692 | 19787761 |  |
| supercont2.347 | 105928 | 1 | chr5 | 19787862 | 19893789 |  |
| supercont2.206 | 173567 | 1 | chr5 | 19893890 | 20067456 |  |
| supercont2.165 | 222249 | 1 | chr5 | 20067557 | 20289805 |  |
| supercont2.463 | 1 | 66225 | chr5 | 20289906 | 20356130 |  |
| supercont2.822 | 10632 | 1 | chr5 | 20356231 | 20366862 |  |
| supercont2.525 | 57807 | 1 | chr5 | 20366963 | 20424769 |  |
| supercont2.166 | 222149 | 1 | chr5 | 20424870 | 20647018 |  |
| supercont2.554 | 1 | 40326 | chr5 | 20647119 | 20687444 |  |
| supercont2.19 | 479455 | 1216767 | chr5 | 20687545 | 21424857 | x |
| supercont2.494 | 1 | 54381 | chr5 | 21424958 | 21479338 |  |
| supercont2.19 | 479454 | 402449 | chr5 | 21479439 | 21556444 | x |
| supercont2.68 | 495722 | 1 | chr5 | 21556545 | 22052266 |  |
| supercont2.13 | 153665 | 900971 | chr5 | 22052367 | 22799673 | x |
| supercont2.928 | 8837 | 1 | chr5 | 22799774 | 22808610 |  |
| supercont2.460 | 1 | 67882 | chr5 | 22808711 | 22876592 |  |
| supercont2.176 | 1 | 200988 | chr5 | 22876693 | 23077680 |  |
| supercont2.126 | 1 | 298207 | chr5 | 23077781 | 23375987 |  |
| supercont2.44 | 1 | 668738 | chr5 | 23376088 | 24044825 |  |
| supercont2.52 | 622908 | 1 | chr5 | 24044926 | 24667833 |  |
| supercont2.435 | 1 | 67758 | chr5 | 24667934 | 24735691 |  |
| supercont2.392 | 81149 | 1 | chr5 | 24735792 | 24816940 |  |
| supercont2.290 | 120448 | 1 | chr5 | 24817041 | 24937488 |  |
| supercont2.130 | 291376 | 1 | chr5 | 24937589 | 25228964 |  |
| supercont2.95 | 382643 | 1 | chr5 | 25229065 | 25611707 |  |
| supercont2.150 | 249777 | 1 | chr5 | 25611808 | 25861584 |  |
| supercont2.580 | 36102 | 1 | chr5 | 25861685 | 25897786 |  |
| supercont2.109 | 353224 | 1 | chr5 | 25897887 | 26251110 |  |
| supercont2.495 | 54360 | 1 | chr5 | 26251211 | 26305570 |  |
| supercont2.65 | 1 | 518654 | chr5 | 26305671 | 26824324 |  |
| supercont2.253 | 136781 | 1 | chr5 | 26824425 | 26961205 |  |
| supercont2.70 | 1 | 505245 | chr5 | 26961306 | 27466550 |  |
| supercont2.225 | 1 | 158369 | unassigned |  |  |  |
| supercont2.260 | 1 | 140465 | unassigned |  |  |  |
| supercont2.266 | 1 | 133799 | unassigned |  |  |  |
| supercont2.298 | 1 | 114988 | unassigned |  |  |  |
| supercont2.309 | 1 | 113855 | unassigned |  |  |  |
| supercont2.351 | 1 | 103950 | unassigned |  |  |  |
| supercont2.349 | 1 | 98020 | unassigned |  |  |  |
| supercont2.357 | 1 | 94581 | unassigned |  |  |  |
| supercont2.417 | 1 | 74222 | unassigned |  |  |  |
| supercont2.433 | 1 | 68700 | unassigned |  |  |  |
| supercont2.479 | 1 | 57970 | unassigned |  |  |  |
| supercont2.483 | 1 | 56685 | unassigned |  |  |  |
| supercont2.494 | 1 | 54381 | unassigned |  |  |  |
| supercont2.502 | 1 | 52538 | unassigned |  |  |  |
| supercont2.529 | 1 | 46277 | unassigned |  |  |  |
| supercont2.528 | 1 | 46060 | unassigned |  |  |  |
| supercont2.598 | 1 | 43829 | unassigned |  |  |  |
| supercont2.543 | 1 | 43098 | unassigned |  |  |  |
| supercont2.553 | 1 | 40547 | unassigned |  |  |  |
| supercont2.572 | 1 | 37598 | unassigned |  |  |  |
| supercont2.570 | 1 | 37249 | unassigned |  |  |  |
| supercont2.583 | 1 | 35820 | unassigned |  |  |  |
| supercont2.588 | 1 | 34902 | unassigned |  |  |  |
| supercont2.587 | 1 | 34844 | unassigned |  |  |  |
| supercont2.601 | 1 | 33606 | unassigned |  |  |  |
| supercont2.610 | 1 | 33247 | unassigned |  |  |  |
| supercont2.600 | 1 | 33124 | unassigned |  |  |  |
| supercont2.616 | 1 | 30980 | unassigned |  |  |  |
| supercont2.628 | 1 | 30003 | unassigned |  |  |  |
| supercont2.638 | 1 | 27852 | unassigned |  |  |  |
| supercont2.652 | 1 | 26273 | unassigned |  |  |  |
| supercont2.649 | 1 | 26263 | unassigned |  |  |  |
| supercont2.704 | 1 | 26206 | unassigned |  |  |  |
| supercont2.653 | 1 | 26056 | unassigned |  |  |  |
| supercont2.661 | 1 | 25973 | unassigned |  |  |  |
| supercont2.672 | 1 | 25849 | unassigned |  |  |  |
| supercont2.662 | 1 | 25465 | unassigned |  |  |  |
| supercont2.660 | 1 | 25388 | unassigned |  |  |  |
| supercont2.664 | 1 | 24798 | unassigned |  |  |  |
| supercont2.679 | 1 | 23253 | unassigned |  |  |  |
| supercont2.675 | 1 | 23205 | unassigned |  |  |  |
| supercont2.690 | 1 | 22055 | unassigned |  |  |  |
| supercont2.699 | 1 | 21794 | unassigned |  |  |  |
| supercont2.689 | 1 | 21564 | unassigned |  |  |  |
| supercont2.691 | 1 | 21418 | unassigned |  |  |  |
| supercont2.692 | 1 | 21309 | unassigned |  |  |  |
| supercont2.707 | 1 | 20410 | unassigned |  |  |  |
| supercont2.714 | 1 | 20155 | unassigned |  |  |  |
| supercont2.708 | 1 | 19755 | unassigned |  |  |  |
| supercont2.713 | 1 | 19545 | unassigned |  |  |  |
| supercont2.710 | 1 | 19376 | unassigned |  |  |  |
| supercont2.737 | 1 | 19346 | unassigned |  |  |  |
| supercont2.712 | 1 | 19080 | unassigned |  |  |  |
| supercont2.715 | 1 | 19014 | unassigned |  |  |  |
| supercont2.722 | 1 | 18361 | unassigned |  |  |  |
| supercont2.719 | 1 | 18270 | unassigned |  |  |  |
| supercont2.724 | 1 | 17964 | unassigned |  |  |  |
| supercont2.731 | 1 | 17260 | unassigned |  |  |  |
| supercont2.735 | 1 | 16854 | unassigned |  |  |  |
| supercont2.752 | 1 | 16650 | unassigned |  |  |  |
| supercont2.739 | 1 | 16605 | unassigned |  |  |  |
| supercont2.741 | 1 | 16304 | unassigned |  |  |  |
| supercont2.793 | 1 | 16177 | unassigned |  |  |  |
| supercont2.748 | 1 | 15999 | unassigned |  |  |  |
| supercont2.744 | 1 | 15955 | unassigned |  |  |  |
| supercont2.747 | 1 | 15815 | unassigned |  |  |  |
| supercont2.750 | 1 | 15788 | unassigned |  |  |  |
| supercont2.746 | 1 | 15656 | unassigned |  |  |  |
| supercont2.764 | 1 | 15574 | unassigned |  |  |  |
| supercont2.754 | 1 | 15395 | unassigned |  |  |  |
| supercont2.760 | 1 | 15218 | unassigned |  |  |  |
| supercont2.756 | 1 | 15204 | unassigned |  |  |  |
| supercont2.758 | 1 | 15154 | unassigned |  |  |  |
| supercont2.759 | 1 | 15118 | unassigned |  |  |  |
| supercont2.761 | 1 | 15012 | unassigned |  |  |  |
| supercont2.837 | 1 | 14973 | unassigned |  |  |  |
| supercont2.783 | 1 | 14908 | unassigned |  |  |  |
| supercont2.766 | 1 | 14769 | unassigned |  |  |  |
| supercont2.767 | 1 | 14637 | unassigned |  |  |  |
| supercont2.770 | 1 | 14435 | unassigned |  |  |  |
| supercont2.769 | 1 | 14423 | unassigned |  |  |  |
| supercont2.772 | 1 | 14317 | unassigned |  |  |  |
| supercont2.773 | 1 | 14274 | unassigned |  |  |  |
| supercont2.775 | 1 | 14145 | unassigned |  |  |  |
| supercont2.801 | 1 | 14009 | unassigned |  |  |  |
| supercont2.791 | 1 | 13939 | unassigned |  |  |  |
| supercont2.777 | 1 | 13820 | unassigned |  |  |  |
| supercont2.778 | 1 | 13690 | unassigned |  |  |  |
| supercont2.779 | 1 | 13688 | unassigned |  |  |  |
| supercont2.781 | 1 | 13625 | unassigned |  |  |  |
| supercont2.782 | 1 | 13496 | unassigned |  |  |  |
| supercont2.785 | 1 | 13212 | unassigned |  |  |  |
| supercont2.794 | 1 | 13004 | unassigned |  |  |  |
| supercont2.798 | 1 | 12626 | unassigned |  |  |  |
| supercont2.796 | 1 | 12625 | unassigned |  |  |  |
| supercont2.800 | 1 | 12525 | unassigned |  |  |  |
| supercont2.799 | 1 | 12471 | unassigned |  |  |  |
| supercont2.802 | 1 | 12422 | unassigned |  |  |  |
| supercont2.828 | 1 | 12406 | unassigned |  |  |  |
| supercont2.804 | 1 | 12121 | unassigned |  |  |  |
| supercont2.803 | 1 | 12097 | unassigned |  |  |  |
| supercont2.811 | 1 | 11685 | unassigned |  |  |  |
| supercont2.806 | 1 | 11604 | unassigned |  |  |  |
| supercont2.810 | 1 | 11594 | unassigned |  |  |  |
| supercont2.820 | 1 | 11555 | unassigned |  |  |  |
| supercont2.812 | 1 | 11204 | unassigned |  |  |  |
| supercont2.814 | 1 | 11098 | unassigned |  |  |  |
| supercont2.816 | 1 | 10807 | unassigned |  |  |  |
| supercont2.818 | 1 | 10784 | unassigned |  |  |  |
| supercont2.819 | 1 | 10711 | unassigned |  |  |  |
| supercont2.821 | 1 | 10687 | unassigned |  |  |  |
| supercont2.827 | 1 | 10625 | unassigned |  |  |  |
| supercont2.823 | 1 | 10611 | unassigned |  |  |  |
| supercont2.825 | 1 | 10555 | unassigned |  |  |  |
| supercont2.829 | 1 | 10229 | unassigned |  |  |  |
| supercont2.830 | 1 | 10207 | unassigned |  |  |  |
| supercont2.832 | 1 | 10186 | unassigned |  |  |  |
| supercont2.835 | 1 | 10056 | unassigned |  |  |  |
| supercont2.882 | 1 | 10040 | unassigned |  |  |  |
| supercont2.975 | 1 | 10009 | unassigned |  |  |  |
| supercont2.840 | 1 | 9947 | unassigned |  |  |  |
| supercont2.839 | 1 | 9941 | unassigned |  |  |  |
| supercont2.841 | 1 | 9826 | unassigned |  |  |  |
| supercont2.842 | 1 | 9761 | unassigned |  |  |  |
| supercont2.851 | 1 | 9476 | unassigned |  |  |  |
| supercont2.846 | 1 | 9453 | unassigned |  |  |  |
| supercont2.850 | 1 | 9301 | unassigned |  |  |  |
| supercont2.852 | 1 | 9215 | unassigned |  |  |  |
| supercont2.853 | 1 | 9182 | unassigned |  |  |  |
| supercont2.855 | 1 | 9113 | unassigned |  |  |  |
| supercont2.866 | 1 | 9098 | unassigned |  |  |  |
| supercont2.916 | 1 | 9072 | unassigned |  |  |  |
| supercont2.856 | 1 | 9069 | unassigned |  |  |  |
| supercont2.857 | 1 | 9027 | unassigned |  |  |  |
| supercont2.860 | 1 | 8972 | unassigned |  |  |  |
| supercont2.863 | 1 | 8871 | unassigned |  |  |  |
| supercont2.861 | 1 | 8837 | unassigned |  |  |  |
| supercont2.867 | 1 | 8636 | unassigned |  |  |  |
| supercont2.868 | 1 | 8630 | unassigned |  |  |  |
| supercont2.869 | 1 | 8543 | unassigned |  |  |  |
| supercont2.871 | 1 | 8520 | unassigned |  |  |  |
| supercont2.872 | 1 | 8514 | unassigned |  |  |  |
| supercont2.898 | 1 | 8513 | unassigned |  |  |  |
| supercont2.875 | 1 | 8375 | unassigned |  |  |  |
| supercont2.874 | 1 | 8366 | unassigned |  |  |  |
| supercont2.878 | 1 | 8321 | unassigned |  |  |  |
| supercont2.905 | 1 | 8258 | unassigned |  |  |  |
| supercont2.879 | 1 | 8207 | unassigned |  |  |  |
| supercont2.876 | 1 | 8185 | unassigned |  |  |  |
| supercont2.881 | 1 | 8141 | unassigned |  |  |  |
| supercont2.883 | 1 | 8138 | unassigned |  |  |  |
| supercont2.884 | 1 | 7955 | unassigned |  |  |  |
| supercont2.887 | 1 | 7909 | unassigned |  |  |  |
| supercont2.888 | 1 | 7883 | unassigned |  |  |  |
| supercont2.889 | 1 | 7868 | unassigned |  |  |  |
| supercont2.886 | 1 | 7858 | unassigned |  |  |  |
| supercont2.890 | 1 | 7821 | unassigned |  |  |  |
| supercont2.892 | 1 | 7744 | unassigned |  |  |  |
| supercont2.893 | 1 | 7725 | unassigned |  |  |  |
| supercont2.891 | 1 | 7717 | unassigned |  |  |  |
| supercont2.895 | 1 | 7597 | unassigned |  |  |  |
| supercont2.896 | 1 | 7530 | unassigned |  |  |  |
| supercont2.900 | 1 | 7297 | unassigned |  |  |  |
| supercont2.1464 | 1 | 7158 | unassigned |  |  |  |
| supercont2.904 | 1 | 7148 | unassigned |  |  |  |
| supercont2.903 | 1 | 7122 | unassigned |  |  |  |
| supercont2.902 | 1 | 7059 | unassigned |  |  |  |
| supercont2.906 | 1 | 7049 | unassigned |  |  |  |
| supercont2.907 | 1 | 7029 | unassigned |  |  |  |
| supercont2.908 | 1 | 6945 | unassigned |  |  |  |
| supercont2.909 | 1 | 6941 | unassigned |  |  |  |
| supercont2.910 | 1 | 6913 | unassigned |  |  |  |
| supercont2.912 | 1 | 6782 | unassigned |  |  |  |
| supercont2.1354 | 1 | 6749 | unassigned |  |  |  |
| supercont2.915 | 1 | 6744 | unassigned |  |  |  |
| supercont2.911 | 1 | 6744 | unassigned |  |  |  |
| supercont2.917 | 1 | 6710 | unassigned |  |  |  |
| supercont2.918 | 1 | 6672 | unassigned |  |  |  |
| supercont2.919 | 1 | 6645 | unassigned |  |  |  |
| supercont2.836 | 1 | 6622 | unassigned |  |  |  |
| supercont2.934 | 1 | 6532 | unassigned |  |  |  |
| supercont2.920 | 1 | 6458 | unassigned |  |  |  |
| supercont2.924 | 1 | 6428 | unassigned |  |  |  |
| supercont2.921 | 1 | 6403 | unassigned |  |  |  |
| supercont2.922 | 1 | 6394 | unassigned |  |  |  |
| supercont2.925 | 1 | 6380 | unassigned |  |  |  |
| supercont2.926 | 1 | 6378 | unassigned |  |  |  |
| supercont2.927 | 1 | 6333 | unassigned |  |  |  |
| supercont2.930 | 1 | 6262 | unassigned |  |  |  |
| supercont2.929 | 1 | 6245 | unassigned |  |  |  |
| supercont2.931 | 1 | 6243 | unassigned |  |  |  |
| supercont2.788 | 1 | 6210 | unassigned |  |  |  |
| supercont2.932 | 1 | 6184 | unassigned |  |  |  |
| supercont2.935 | 1 | 6166 | unassigned |  |  |  |
| supercont2.936 | 1 | 6133 | unassigned |  |  |  |
| supercont2.933 | 1 | 6037 | unassigned |  |  |  |
| supercont2.938 | 1 | 6026 | unassigned |  |  |  |
| supercont2.939 | 1 | 6006 | unassigned |  |  |  |
| supercont2.942 | 1 | 5983 | unassigned |  |  |  |
| supercont2.940 | 1 | 5972 | unassigned |  |  |  |
| supercont2.944 | 1 | 5948 | unassigned |  |  |  |
| supercont2.941 | 1 | 5936 | unassigned |  |  |  |
| supercont2.948 | 1 | 5890 | unassigned |  |  |  |
| supercont2.946 | 1 | 5854 | unassigned |  |  |  |
| supercont2.949 | 1 | 5737 | unassigned |  |  |  |
| supercont2.950 | 1 | 5715 | unassigned |  |  |  |
| supercont2.952 | 1 | 5665 | unassigned |  |  |  |
| supercont2.951 | 1 | 5633 | unassigned |  |  |  |
| supercont2.957 | 1 | 5519 | unassigned |  |  |  |
| supercont2.954 | 1 | 5518 | unassigned |  |  |  |
| supercont2.958 | 1 | 5516 | unassigned |  |  |  |
| supercont2.956 | 1 | 5486 | unassigned |  |  |  |
| supercont2.960 | 1 | 5461 | unassigned |  |  |  |
| supercont2.963 | 1 | 5425 | unassigned |  |  |  |
| supercont2.962 | 1 | 5422 | unassigned |  |  |  |
| supercont2.965 | 1 | 5403 | unassigned |  |  |  |
| supercont2.966 | 1 | 5397 | unassigned |  |  |  |
| supercont2.967 | 1 | 5380 | unassigned |  |  |  |
| supercont2.968 | 1 | 5377 | unassigned |  |  |  |
| supercont2.996 | 1 | 5332 | unassigned |  |  |  |
| supercont2.970 | 1 | 5328 | unassigned |  |  |  |
| supercont2.971 | 1 | 5324 | unassigned |  |  |  |
| supercont2.973 | 1 | 5286 | unassigned |  |  |  |
| supercont2.974 | 1 | 5286 | unassigned |  |  |  |
| supercont2.976 | 1 | 5273 | unassigned |  |  |  |
| supercont2.979 | 1 | 5197 | unassigned |  |  |  |
| supercont2.977 | 1 | 5188 | unassigned |  |  |  |
| supercont2.980 | 1 | 5135 | unassigned |  |  |  |
| supercont2.982 | 1 | 5087 | unassigned |  |  |  |
| supercont2.983 | 1 | 5072 | unassigned |  |  |  |
| supercont2.981 | 1 | 5063 | unassigned |  |  |  |
| supercont2.984 | 1 | 5016 | unassigned |  |  |  |
| supercont2.985 | 1 | 4838 | unassigned |  |  |  |
| supercont2.986 | 1 | 4814 | unassigned |  |  |  |
| supercont2.987 | 1 | 4801 | unassigned |  |  |  |
| supercont2.989 | 1 | 4653 | unassigned |  |  |  |
| supercont2.990 | 1 | 4652 | unassigned |  |  |  |
| supercont2.991 | 1 | 4649 | unassigned |  |  |  |
| supercont2.1061 | 1 | 4648 | unassigned |  |  |  |
| supercont2.988 | 1 | 4636 | unassigned |  |  |  |
| supercont2.1002 | 1 | 4561 | unassigned |  |  |  |
| supercont2.993 | 1 | 4540 | unassigned |  |  |  |
| supercont2.992 | 1 | 4514 | unassigned |  |  |  |
| supercont2.994 | 1 | 4472 | unassigned |  |  |  |
| supercont2.997 | 1 | 4407 | unassigned |  |  |  |
| supercont2.998 | 1 | 4354 | unassigned |  |  |  |
| supercont2.999 | 1 | 4353 | unassigned |  |  |  |
| supercont2.1000 | 1 | 4353 | unassigned |  |  |  |
| supercont2.1001 | 1 | 4318 | unassigned |  |  |  |
| supercont2.1003 | 1 | 4173 | unassigned |  |  |  |
| supercont2.1004 | 1 | 4170 | unassigned |  |  |  |
| supercont2.1006 | 1 | 3980 | unassigned |  |  |  |
| supercont2.1007 | 1 | 3898 | unassigned |  |  |  |
| supercont2.1009 | 1 | 3823 | unassigned |  |  |  |
| supercont2.1011 | 1 | 3814 | unassigned |  |  |  |
| supercont2.1010 | 1 | 3814 | unassigned |  |  |  |
| supercont2.1014 | 1 | 3743 | unassigned |  |  |  |
| supercont2.1013 | 1 | 3726 | unassigned |  |  |  |
| supercont2.1012 | 1 | 3715 | unassigned |  |  |  |
| supercont2.1015 | 1 | 3694 | unassigned |  |  |  |
| supercont2.1016 | 1 | 3660 | unassigned |  |  |  |
| supercont2.1017 | 1 | 3652 | unassigned |  |  |  |
| supercont2.1018 | 1 | 3603 | unassigned |  |  |  |
| supercont2.1047 | 1 | 3599 | unassigned |  |  |  |
| supercont2.1019 | 1 | 3544 | unassigned |  |  |  |
| supercont2.1020 | 1 | 3536 | unassigned |  |  |  |
| supercont2.1021 | 1 | 3514 | unassigned |  |  |  |
| supercont2.1023 | 1 | 3496 | unassigned |  |  |  |
| supercont2.1022 | 1 | 3491 | unassigned |  |  |  |
| supercont2.1025 | 1 | 3464 | unassigned |  |  |  |
| supercont2.1024 | 1 | 3429 | unassigned |  |  |  |
| supercont2.1026 | 1 | 3405 | unassigned |  |  |  |
| supercont2.1028 | 1 | 3296 | unassigned |  |  |  |
| supercont2.1030 | 1 | 3142 | unassigned |  |  |  |
| supercont2.1031 | 1 | 3120 | unassigned |  |  |  |
| supercont2.1032 | 1 | 3101 | unassigned |  |  |  |
| supercont2.1059 | 1 | 3045 | unassigned |  |  |  |
| supercont2.1034 | 1 | 3014 | unassigned |  |  |  |
| supercont2.1033 | 1 | 2986 | unassigned |  |  |  |
| supercont2.1038 | 1 | 2984 | unassigned |  |  |  |
| supercont2.1035 | 1 | 2978 | unassigned |  |  |  |
| supercont2.1039 | 1 | 2969 | unassigned |  |  |  |
| supercont2.1040 | 1 | 2960 | unassigned |  |  |  |
| supercont2.1037 | 1 | 2940 | unassigned |  |  |  |
| supercont2.1041 | 1 | 2931 | unassigned |  |  |  |
| supercont2.1036 | 1 | 2929 | unassigned |  |  |  |
| supercont2.1043 | 1 | 2889 | unassigned |  |  |  |
| supercont2.1042 | 1 | 2875 | unassigned |  |  |  |
| supercont2.1044 | 1 | 2870 | unassigned |  |  |  |
| supercont2.1046 | 1 | 2834 | unassigned |  |  |  |
| supercont2.1045 | 1 | 2810 | unassigned |  |  |  |
| supercont2.1049 | 1 | 2770 | unassigned |  |  |  |
| supercont2.1050 | 1 | 2764 | unassigned |  |  |  |
| supercont2.1048 | 1 | 2735 | unassigned |  |  |  |
| supercont2.1051 | 1 | 2733 | unassigned |  |  |  |
| supercont2.1052 | 1 | 2723 | unassigned |  |  |  |
| supercont2.1053 | 1 | 2703 | unassigned |  |  |  |
| supercont2.1054 | 1 | 2687 | unassigned |  |  |  |
| supercont2.1056 | 1 | 2666 | unassigned |  |  |  |
| supercont2.1057 | 1 | 2666 | unassigned |  |  |  |
| supercont2.1058 | 1 | 2665 | unassigned |  |  |  |
| supercont2.1060 | 1 | 2652 | unassigned |  |  |  |
| supercont2.1062 | 1 | 2617 | unassigned |  |  |  |
| supercont2.1063 | 1 | 2615 | unassigned |  |  |  |
| supercont2.1064 | 1 | 2609 | unassigned |  |  |  |
| supercont2.1067 | 1 | 2587 | unassigned |  |  |  |
| supercont2.1066 | 1 | 2558 | unassigned |  |  |  |
| supercont2.1068 | 1 | 2554 | unassigned |  |  |  |
| supercont2.1069 | 1 | 2553 | unassigned |  |  |  |
| supercont2.1065 | 1 | 2540 | unassigned |  |  |  |
| supercont2.1071 | 1 | 2531 | unassigned |  |  |  |
| supercont2.1072 | 1 | 2528 | unassigned |  |  |  |
| supercont2.1070 | 1 | 2502 | unassigned |  |  |  |
| supercont2.1076 | 1 | 2500 | unassigned |  |  |  |
| supercont2.1074 | 1 | 2489 | unassigned |  |  |  |
| supercont2.1073 | 1 | 2488 | unassigned |  |  |  |
| supercont2.1077 | 1 | 2479 | unassigned |  |  |  |
| supercont2.1078 | 1 | 2472 | unassigned |  |  |  |
| supercont2.1079 | 1 | 2471 | unassigned |  |  |  |
| supercont2.1080 | 1 | 2471 | unassigned |  |  |  |
| supercont2.1075 | 1 | 2460 | unassigned |  |  |  |
| supercont2.1081 | 1 | 2444 | unassigned |  |  |  |
| supercont2.1082 | 1 | 2440 | unassigned |  |  |  |
| supercont2.1083 | 1 | 2386 | unassigned |  |  |  |
| supercont2.1084 | 1 | 2378 | unassigned |  |  |  |
| supercont2.1085 | 1 | 2338 | unassigned |  |  |  |
| supercont2.1086 | 1 | 2325 | unassigned |  |  |  |
| supercont2.1087 | 1 | 2321 | unassigned |  |  |  |
| supercont2.1088 | 1 | 2319 | unassigned |  |  |  |
| supercont2.1089 | 1 | 2296 | unassigned |  |  |  |
| supercont2.1090 | 1 | 2286 | unassigned |  |  |  |
| supercont2.1091 | 1 | 2282 | unassigned |  |  |  |
| supercont2.1093 | 1 | 2277 | unassigned |  |  |  |
| supercont2.1094 | 1 | 2277 | unassigned |  |  |  |
| supercont2.1092 | 1 | 2270 | unassigned |  |  |  |
| supercont2.1096 | 1 | 2269 | unassigned |  |  |  |
| supercont2.1095 | 1 | 2265 | unassigned |  |  |  |
| supercont2.953 | 1 | 2258 | unassigned |  |  |  |
| supercont2.1098 | 1 | 2250 | unassigned |  |  |  |
| supercont2.1097 | 1 | 2245 | unassigned |  |  |  |
| supercont2.1099 | 1 | 2244 | unassigned |  |  |  |
| supercont2.1100 | 1 | 2243 | unassigned |  |  |  |
| supercont2.1103 | 1 | 2190 | unassigned |  |  |  |
| supercont2.1105 | 1 | 2181 | unassigned |  |  |  |
| supercont2.1101 | 1 | 2179 | unassigned |  |  |  |
| supercont2.1102 | 1 | 2174 | unassigned |  |  |  |
| supercont2.1109 | 1 | 2137 | unassigned |  |  |  |
| supercont2.1110 | 1 | 2131 | unassigned |  |  |  |
| supercont2.1107 | 1 | 2127 | unassigned |  |  |  |
| supercont2.1112 | 1 | 2123 | unassigned |  |  |  |
| supercont2.1111 | 1 | 2123 | unassigned |  |  |  |
| supercont2.1104 | 1 | 2120 | unassigned |  |  |  |
| supercont2.1106 | 1 | 2107 | unassigned |  |  |  |
| supercont2.1113 | 1 | 2072 | unassigned |  |  |  |
| supercont2.1119 | 1 | 2068 | unassigned |  |  |  |
| supercont2.1118 | 1 | 2067 | unassigned |  |  |  |
| supercont2.1121 | 1 | 2066 | unassigned |  |  |  |
| supercont2.1122 | 1 | 2062 | unassigned |  |  |  |
| supercont2.1116 | 1 | 2062 | unassigned |  |  |  |
| supercont2.1117 | 1 | 2061 | unassigned |  |  |  |
| supercont2.1123 | 1 | 2059 | unassigned |  |  |  |
| supercont2.1125 | 1 | 2055 | unassigned |  |  |  |
| supercont2.1126 | 1 | 2054 | unassigned |  |  |  |
| supercont2.1115 | 1 | 2051 | unassigned |  |  |  |
| supercont2.1129 | 1 | 2027 | unassigned |  |  |  |
| supercont2.1130 | 1 | 2021 | unassigned |  |  |  |
| supercont2.1114 | 1 | 2014 | unassigned |  |  |  |
| supercont2.1131 | 1 | 2013 | unassigned |  |  |  |
| supercont2.1120 | 1 | 2011 | unassigned |  |  |  |
| supercont2.1128 | 1 | 2011 | unassigned |  |  |  |
| supercont2.1124 | 1 | 2010 | unassigned |  |  |  |
| supercont2.1133 | 1 | 2000 | unassigned |  |  |  |
| supercont2.1134 | 1 | 1996 | unassigned |  |  |  |
| supercont2.1127 | 1 | 1992 | unassigned |  |  |  |
| supercont2.1135 | 1 | 1992 | unassigned |  |  |  |
| supercont2.1136 | 1 | 1988 | unassigned |  |  |  |
| supercont2.1137 | 1 | 1987 | unassigned |  |  |  |
| supercont2.1138 | 1 | 1982 | unassigned |  |  |  |
| supercont2.1139 | 1 | 1978 | unassigned |  |  |  |
| supercont2.1140 | 1 | 1972 | unassigned |  |  |  |
| supercont2.1141 | 1 | 1959 | unassigned |  |  |  |
| supercont2.1144 | 1 | 1947 | unassigned |  |  |  |
| supercont2.1143 | 1 | 1947 | unassigned |  |  |  |
| supercont2.1145 | 1 | 1936 | unassigned |  |  |  |
| supercont2.1148 | 1 | 1932 | unassigned |  |  |  |
| supercont2.1149 | 1 | 1932 | unassigned |  |  |  |
| supercont2.1151 | 1 | 1921 | unassigned |  |  |  |
| supercont2.1132 | 1 | 1918 | unassigned |  |  |  |
| supercont2.1152 | 1 | 1915 | unassigned |  |  |  |
| supercont2.1147 | 1 | 1900 | unassigned |  |  |  |
| supercont2.1153 | 1 | 1900 | unassigned |  |  |  |
| supercont2.1154 | 1 | 1896 | unassigned |  |  |  |
| supercont2.1157 | 1 | 1887 | unassigned |  |  |  |
| supercont2.1158 | 1 | 1883 | unassigned |  |  |  |
| supercont2.1146 | 1 | 1878 | unassigned |  |  |  |
| supercont2.1161 | 1 | 1873 | unassigned |  |  |  |
| supercont2.1162 | 1 | 1870 | unassigned |  |  |  |
| supercont2.1160 | 1 | 1870 | unassigned |  |  |  |
| supercont2.1163 | 1 | 1863 | unassigned |  |  |  |
| supercont2.1156 | 1 | 1859 | unassigned |  |  |  |
| supercont2.1166 | 1 | 1853 | unassigned |  |  |  |
| supercont2.1164 | 1 | 1849 | unassigned |  |  |  |
| supercont2.1167 | 1 | 1848 | unassigned |  |  |  |
| supercont2.1169 | 1 | 1843 | unassigned |  |  |  |
| supercont2.1155 | 1 | 1841 | unassigned |  |  |  |
| supercont2.1142 | 1 | 1834 | unassigned |  |  |  |
| supercont2.1171 | 1 | 1832 | unassigned |  |  |  |
| supercont2.1173 | 1 | 1827 | unassigned |  |  |  |
| supercont2.1172 | 1 | 1827 | unassigned |  |  |  |
| supercont2.1174 | 1 | 1823 | unassigned |  |  |  |
| supercont2.1159 | 1 | 1819 | unassigned |  |  |  |
| supercont2.1176 | 1 | 1814 | unassigned |  |  |  |
| supercont2.1177 | 1 | 1811 | unassigned |  |  |  |
| supercont2.1165 | 1 | 1809 | unassigned |  |  |  |
| supercont2.1179 | 1 | 1808 | unassigned |  |  |  |
| supercont2.1182 | 1 | 1793 | unassigned |  |  |  |
| supercont2.1183 | 1 | 1792 | unassigned |  |  |  |
| supercont2.1170 | 1 | 1788 | unassigned |  |  |  |
| supercont2.1168 | 1 | 1786 | unassigned |  |  |  |
| supercont2.1185 | 1 | 1782 | unassigned |  |  |  |
| supercont2.1180 | 1 | 1778 | unassigned |  |  |  |
| supercont2.1187 | 1 | 1766 | unassigned |  |  |  |
| supercont2.1188 | 1 | 1754 | unassigned |  |  |  |
| supercont2.1178 | 1 | 1753 | unassigned |  |  |  |
| supercont2.1189 | 1 | 1749 | unassigned |  |  |  |
| supercont2.1190 | 1 | 1745 | unassigned |  |  |  |
| supercont2.1186 | 1 | 1741 | unassigned |  |  |  |
| supercont2.1184 | 1 | 1731 | unassigned |  |  |  |
| supercont2.1191 | 1 | 1729 | unassigned |  |  |  |
| supercont2.1193 | 1 | 1728 | unassigned |  |  |  |
| supercont2.1194 | 1 | 1721 | unassigned |  |  |  |
| supercont2.1175 | 1 | 1718 | unassigned |  |  |  |
| supercont2.1195 | 1 | 1713 | unassigned |  |  |  |
| supercont2.1196 | 1 | 1707 | unassigned |  |  |  |
| supercont2.1181 | 1 | 1701 | unassigned |  |  |  |
| supercont2.1201 | 1 | 1691 | unassigned |  |  |  |
| supercont2.1203 | 1 | 1689 | unassigned |  |  |  |
| supercont2.1202 | 1 | 1689 | unassigned |  |  |  |
| supercont2.1200 | 1 | 1685 | unassigned |  |  |  |
| supercont2.1205 | 1 | 1679 | unassigned |  |  |  |
| supercont2.1206 | 1 | 1674 | unassigned |  |  |  |
| supercont2.1208 | 1 | 1671 | unassigned |  |  |  |
| supercont2.1204 | 1 | 1671 | unassigned |  |  |  |
| supercont2.1209 | 1 | 1671 | unassigned |  |  |  |
| supercont2.1197 | 1 | 1670 | unassigned |  |  |  |
| supercont2.1210 | 1 | 1669 | unassigned |  |  |  |
| supercont2.1211 | 1 | 1662 | unassigned |  |  |  |
| supercont2.1212 | 1 | 1657 | unassigned |  |  |  |
| supercont2.1199 | 1 | 1656 | unassigned |  |  |  |
| supercont2.1214 | 1 | 1652 | unassigned |  |  |  |
| supercont2.880 | 1 | 1652 | unassigned |  |  |  |
| supercont2.1215 | 1 | 1651 | unassigned |  |  |  |
| supercont2.1216 | 1 | 1650 | unassigned |  |  |  |
| supercont2.1217 | 1 | 1643 | unassigned |  |  |  |
| supercont2.1218 | 1 | 1642 | unassigned |  |  |  |
| supercont2.1220 | 1 | 1639 | unassigned |  |  |  |
| supercont2.1221 | 1 | 1638 | unassigned |  |  |  |
| supercont2.1222 | 1 | 1633 | unassigned |  |  |  |
| supercont2.1226 | 1 | 1630 | unassigned |  |  |  |
| supercont2.1224 | 1 | 1630 | unassigned |  |  |  |
| supercont2.1225 | 1 | 1630 | unassigned |  |  |  |
| supercont2.1223 | 1 | 1629 | unassigned |  |  |  |
| supercont2.1227 | 1 | 1628 | unassigned |  |  |  |
| supercont2.1228 | 1 | 1626 | unassigned |  |  |  |
| supercont2.1192 | 1 | 1622 | unassigned |  |  |  |
| supercont2.1231 | 1 | 1619 | unassigned |  |  |  |
| supercont2.1233 | 1 | 1617 | unassigned |  |  |  |
| supercont2.1213 | 1 | 1615 | unassigned |  |  |  |
| supercont2.1232 | 1 | 1612 | unassigned |  |  |  |
| supercont2.1207 | 1 | 1611 | unassigned |  |  |  |
| supercont2.1235 | 1 | 1608 | unassigned |  |  |  |
| supercont2.1234 | 1 | 1606 | unassigned |  |  |  |
| supercont2.1236 | 1 | 1599 | unassigned |  |  |  |
| supercont2.1237 | 1 | 1596 | unassigned |  |  |  |
| supercont2.1239 | 1 | 1591 | unassigned |  |  |  |
| supercont2.1230 | 1 | 1587 | unassigned |  |  |  |
| supercont2.1241 | 1 | 1581 | unassigned |  |  |  |
| supercont2.1242 | 1 | 1580 | unassigned |  |  |  |
| supercont2.1219 | 1 | 1579 | unassigned |  |  |  |
| supercont2.1238 | 1 | 1577 | unassigned |  |  |  |
| supercont2.1229 | 1 | 1576 | unassigned |  |  |  |
| supercont2.1243 | 1 | 1572 | unassigned |  |  |  |
| supercont2.1244 | 1 | 1568 | unassigned |  |  |  |
| supercont2.1245 | 1 | 1568 | unassigned |  |  |  |
| supercont2.1246 | 1 | 1566 | unassigned |  |  |  |
| supercont2.1248 | 1 | 1562 | unassigned |  |  |  |
| supercont2.1249 | 1 | 1561 | unassigned |  |  |  |
| supercont2.1250 | 1 | 1558 | unassigned |  |  |  |
| supercont2.1253 | 1 | 1554 | unassigned |  |  |  |
| supercont2.1254 | 1 | 1550 | unassigned |  |  |  |
| supercont2.1252 | 1 | 1549 | unassigned |  |  |  |
| supercont2.1256 | 1 | 1546 | unassigned |  |  |  |
| supercont2.1259 | 1 | 1535 | unassigned |  |  |  |
| supercont2.1260 | 1 | 1534 | unassigned |  |  |  |
| supercont2.1240 | 1 | 1528 | unassigned |  |  |  |
| supercont2.1261 | 1 | 1527 | unassigned |  |  |  |
| supercont2.1257 | 1 | 1523 | unassigned |  |  |  |
| supercont2.1263 | 1 | 1521 | unassigned |  |  |  |
| supercont2.1266 | 1 | 1510 | unassigned |  |  |  |
| supercont2.1268 | 1 | 1509 | unassigned |  |  |  |
| supercont2.1267 | 1 | 1509 | unassigned |  |  |  |
| supercont2.1247 | 1 | 1505 | unassigned |  |  |  |
| supercont2.1255 | 1 | 1503 | unassigned |  |  |  |
| supercont2.1262 | 1 | 1502 | unassigned |  |  |  |
| supercont2.1264 | 1 | 1499 | unassigned |  |  |  |
| supercont2.1269 | 1 | 1498 | unassigned |  |  |  |
| supercont2.1265 | 1 | 1497 | unassigned |  |  |  |
| supercont2.1271 | 1 | 1496 | unassigned |  |  |  |
| supercont2.1251 | 1 | 1496 | unassigned |  |  |  |
| supercont2.1274 | 1 | 1488 | unassigned |  |  |  |
| supercont2.1273 | 1 | 1486 | unassigned |  |  |  |
| supercont2.1275 | 1 | 1482 | unassigned |  |  |  |
| supercont2.1276 | 1 | 1482 | unassigned |  |  |  |
| supercont2.1277 | 1 | 1478 | unassigned |  |  |  |
| supercont2.1270 | 1 | 1469 | unassigned |  |  |  |
| supercont2.1279 | 1 | 1469 | unassigned |  |  |  |
| supercont2.1284 | 1 | 1463 | unassigned |  |  |  |
| supercont2.1281 | 1 | 1461 | unassigned |  |  |  |
| supercont2.1286 | 1 | 1449 | unassigned |  |  |  |
| supercont2.1287 | 1 | 1446 | unassigned |  |  |  |
| supercont2.1288 | 1 | 1442 | unassigned |  |  |  |
| supercont2.1258 | 1 | 1439 | unassigned |  |  |  |
| supercont2.1289 | 1 | 1439 | unassigned |  |  |  |
| supercont2.1285 | 1 | 1436 | unassigned |  |  |  |
| supercont2.1290 | 1 | 1430 | unassigned |  |  |  |
| supercont2.1272 | 1 | 1430 | unassigned |  |  |  |
| supercont2.1291 | 1 | 1428 | unassigned |  |  |  |
| supercont2.1293 | 1 | 1426 | unassigned |  |  |  |
| supercont2.1292 | 1 | 1426 | unassigned |  |  |  |
| supercont2.1295 | 1 | 1425 | unassigned |  |  |  |
| supercont2.1294 | 1 | 1425 | unassigned |  |  |  |
| supercont2.1296 | 1 | 1423 | unassigned |  |  |  |
| supercont2.1298 | 1 | 1420 | unassigned |  |  |  |
| supercont2.1297 | 1 | 1420 | unassigned |  |  |  |
| supercont2.1299 | 1 | 1414 | unassigned |  |  |  |
| supercont2.1300 | 1 | 1410 | unassigned |  |  |  |
| supercont2.1283 | 1 | 1409 | unassigned |  |  |  |
| supercont2.1301 | 1 | 1408 | unassigned |  |  |  |
| supercont2.1302 | 1 | 1406 | unassigned |  |  |  |
| supercont2.1282 | 1 | 1406 | unassigned |  |  |  |
| supercont2.1304 | 1 | 1402 | unassigned |  |  |  |
| supercont2.1305 | 1 | 1400 | unassigned |  |  |  |
| supercont2.1306 | 1 | 1398 | unassigned |  |  |  |
| supercont2.1309 | 1 | 1392 | unassigned |  |  |  |
| supercont2.1308 | 1 | 1392 | unassigned |  |  |  |
| supercont2.1280 | 1 | 1391 | unassigned |  |  |  |
| supercont2.1312 | 1 | 1387 | unassigned |  |  |  |
| supercont2.1313 | 1 | 1386 | unassigned |  |  |  |
| supercont2.1316 | 1 | 1382 | unassigned |  |  |  |
| supercont2.1278 | 1 | 1378 | unassigned |  |  |  |
| supercont2.1314 | 1 | 1378 | unassigned |  |  |  |
| supercont2.1315 | 1 | 1373 | unassigned |  |  |  |
| supercont2.1319 | 1 | 1371 | unassigned |  |  |  |
| supercont2.1322 | 1 | 1367 | unassigned |  |  |  |
| supercont2.1317 | 1 | 1366 | unassigned |  |  |  |
| supercont2.1323 | 1 | 1366 | unassigned |  |  |  |
| supercont2.1310 | 1 | 1365 | unassigned |  |  |  |
| supercont2.1326 | 1 | 1364 | unassigned |  |  |  |
| supercont2.1327 | 1 | 1361 | unassigned |  |  |  |
| supercont2.1318 | 1 | 1355 | unassigned |  |  |  |
| supercont2.1329 | 1 | 1351 | unassigned |  |  |  |
| supercont2.1330 | 1 | 1349 | unassigned |  |  |  |
| supercont2.1303 | 1 | 1346 | unassigned |  |  |  |
| supercont2.1336 | 1 | 1342 | unassigned |  |  |  |
| supercont2.1333 | 1 | 1342 | unassigned |  |  |  |
| supercont2.1334 | 1 | 1342 | unassigned |  |  |  |
| supercont2.1332 | 1 | 1341 | unassigned |  |  |  |
| supercont2.1335 | 1 | 1341 | unassigned |  |  |  |
| supercont2.1337 | 1 | 1338 | unassigned |  |  |  |
| supercont2.1339 | 1 | 1337 | unassigned |  |  |  |
| supercont2.1338 | 1 | 1337 | unassigned |  |  |  |
| supercont2.1325 | 1 | 1335 | unassigned |  |  |  |
| supercont2.1342 | 1 | 1329 | unassigned |  |  |  |
| supercont2.1343 | 1 | 1329 | unassigned |  |  |  |
| supercont2.1331 | 1 | 1324 | unassigned |  |  |  |
| supercont2.1345 | 1 | 1322 | unassigned |  |  |  |
| supercont2.1346 | 1 | 1319 | unassigned |  |  |  |
| supercont2.1349 | 1 | 1318 | unassigned |  |  |  |
| supercont2.1321 | 1 | 1318 | unassigned |  |  |  |
| supercont2.1353 | 1 | 1315 | unassigned |  |  |  |
| supercont2.1311 | 1 | 1315 | unassigned |  |  |  |
| supercont2.1352 | 1 | 1315 | unassigned |  |  |  |
| supercont2.1347 | 1 | 1312 | unassigned |  |  |  |
| supercont2.1351 | 1 | 1311 | unassigned |  |  |  |
| supercont2.1350 | 1 | 1309 | unassigned |  |  |  |
| supercont2.1358 | 1 | 1306 | unassigned |  |  |  |
| supercont2.1355 | 1 | 1304 | unassigned |  |  |  |
| supercont2.1360 | 1 | 1299 | unassigned |  |  |  |
| supercont2.1361 | 1 | 1297 | unassigned |  |  |  |
| supercont2.1362 | 1 | 1297 | unassigned |  |  |  |
| supercont2.1328 | 1 | 1296 | unassigned |  |  |  |
| supercont2.1363 | 1 | 1294 | unassigned |  |  |  |
| supercont2.1364 | 1 | 1292 | unassigned |  |  |  |
| supercont2.1307 | 1 | 1291 | unassigned |  |  |  |
| supercont2.1324 | 1 | 1290 | unassigned |  |  |  |
| supercont2.1365 | 1 | 1290 | unassigned |  |  |  |
| supercont2.1340 | 1 | 1279 | unassigned |  |  |  |
| supercont2.1344 | 1 | 1279 | unassigned |  |  |  |
| supercont2.1369 | 1 | 1278 | unassigned |  |  |  |
| supercont2.1370 | 1 | 1277 | unassigned |  |  |  |
| supercont2.1372 | 1 | 1276 | unassigned |  |  |  |
| supercont2.1373 | 1 | 1275 | unassigned |  |  |  |
| supercont2.1357 | 1 | 1272 | unassigned |  |  |  |
| supercont2.1375 | 1 | 1271 | unassigned |  |  |  |
| supercont2.1341 | 1 | 1271 | unassigned |  |  |  |
| supercont2.1367 | 1 | 1267 | unassigned |  |  |  |
| supercont2.1348 | 1 | 1262 | unassigned |  |  |  |
| supercont2.1359 | 1 | 1259 | unassigned |  |  |  |
| supercont2.1376 | 1 | 1259 | unassigned |  |  |  |
| supercont2.1378 | 1 | 1257 | unassigned |  |  |  |
| supercont2.1380 | 1 | 1251 | unassigned |  |  |  |
| supercont2.1382 | 1 | 1247 | unassigned |  |  |  |
| supercont2.1381 | 1 | 1247 | unassigned |  |  |  |
| supercont2.1384 | 1 | 1240 | unassigned |  |  |  |
| supercont2.1374 | 1 | 1236 | unassigned |  |  |  |
| supercont2.1386 | 1 | 1236 | unassigned |  |  |  |
| supercont2.1387 | 1 | 1231 | unassigned |  |  |  |
| supercont2.1388 | 1 | 1229 | unassigned |  |  |  |
| supercont2.1389 | 1 | 1228 | unassigned |  |  |  |
| supercont2.1390 | 1 | 1228 | unassigned |  |  |  |
| supercont2.1366 | 1 | 1227 | unassigned |  |  |  |
| supercont2.1391 | 1 | 1227 | unassigned |  |  |  |
| supercont2.1394 | 1 | 1223 | unassigned |  |  |  |
| supercont2.1395 | 1 | 1220 | unassigned |  |  |  |
| supercont2.1356 | 1 | 1219 | unassigned |  |  |  |
| supercont2.1396 | 1 | 1218 | unassigned |  |  |  |
| supercont2.1377 | 1 | 1216 | unassigned |  |  |  |
| supercont2.1397 | 1 | 1216 | unassigned |  |  |  |
| supercont2.1371 | 1 | 1216 | unassigned |  |  |  |
| supercont2.1393 | 1 | 1212 | unassigned |  |  |  |
| supercont2.1385 | 1 | 1211 | unassigned |  |  |  |
| supercont2.1400 | 1 | 1210 | unassigned |  |  |  |
| supercont2.1399 | 1 | 1210 | unassigned |  |  |  |
| supercont2.1401 | 1 | 1209 | unassigned |  |  |  |
| supercont2.1398 | 1 | 1205 | unassigned |  |  |  |
| supercont2.1405 | 1 | 1198 | unassigned |  |  |  |
| supercont2.1402 | 1 | 1198 | unassigned |  |  |  |
| supercont2.1410 | 1 | 1190 | unassigned |  |  |  |
| supercont2.1411 | 1 | 1188 | unassigned |  |  |  |
| supercont2.1412 | 1 | 1185 | unassigned |  |  |  |
| supercont2.1415 | 1 | 1181 | unassigned |  |  |  |
| supercont2.1416 | 1 | 1180 | unassigned |  |  |  |
| supercont2.1417 | 1 | 1178 | unassigned |  |  |  |
| supercont2.1421 | 1 | 1177 | unassigned |  |  |  |
| supercont2.1409 | 1 | 1177 | unassigned |  |  |  |
| supercont2.1423 | 1 | 1175 | unassigned |  |  |  |
| supercont2.1418 | 1 | 1173 | unassigned |  |  |  |
| supercont2.1424 | 1 | 1172 | unassigned |  |  |  |
| supercont2.1419 | 1 | 1168 | unassigned |  |  |  |
| supercont2.1383 | 1 | 1167 | unassigned |  |  |  |
| supercont2.1425 | 1 | 1166 | unassigned |  |  |  |
| supercont2.1404 | 1 | 1160 | unassigned |  |  |  |
| supercont2.1379 | 1 | 1160 | unassigned |  |  |  |
| supercont2.1427 | 1 | 1156 | unassigned |  |  |  |
| supercont2.1428 | 1 | 1156 | unassigned |  |  |  |
| supercont2.1432 | 1 | 1150 | unassigned |  |  |  |
| supercont2.1435 | 1 | 1143 | unassigned |  |  |  |
| supercont2.1413 | 1 | 1142 | unassigned |  |  |  |
| supercont2.1403 | 1 | 1139 | unassigned |  |  |  |
| supercont2.1407 | 1 | 1139 | unassigned |  |  |  |
| supercont2.1436 | 1 | 1138 | unassigned |  |  |  |
| supercont2.1438 | 1 | 1136 | unassigned |  |  |  |
| supercont2.1437 | 1 | 1136 | unassigned |  |  |  |
| supercont2.1408 | 1 | 1133 | unassigned |  |  |  |
| supercont2.1441 | 1 | 1131 | unassigned |  |  |  |
| supercont2.1392 | 1 | 1131 | unassigned |  |  |  |
| supercont2.1430 | 1 | 1130 | unassigned |  |  |  |
| supercont2.1442 | 1 | 1129 | unassigned |  |  |  |
| supercont2.1440 | 1 | 1128 | unassigned |  |  |  |
| supercont2.1414 | 1 | 1125 | unassigned |  |  |  |
| supercont2.1443 | 1 | 1125 | unassigned |  |  |  |
| supercont2.1420 | 1 | 1124 | unassigned |  |  |  |
| supercont2.1446 | 1 | 1121 | unassigned |  |  |  |
| supercont2.1444 | 1 | 1121 | unassigned |  |  |  |
| supercont2.1445 | 1 | 1121 | unassigned |  |  |  |
| supercont2.1448 | 1 | 1120 | unassigned |  |  |  |
| supercont2.1451 | 1 | 1116 | unassigned |  |  |  |
| supercont2.1422 | 1 | 1114 | unassigned |  |  |  |
| supercont2.1447 | 1 | 1109 | unassigned |  |  |  |
| supercont2.1452 | 1 | 1103 | unassigned |  |  |  |
| supercont2.1431 | 1 | 1103 | unassigned |  |  |  |
| supercont2.1453 | 1 | 1102 | unassigned |  |  |  |
| supercont2.1406 | 1 | 1099 | unassigned |  |  |  |
| supercont2.1449 | 1 | 1094 | unassigned |  |  |  |
| supercont2.1434 | 1 | 1094 | unassigned |  |  |  |
| supercont2.1429 | 1 | 1091 | unassigned |  |  |  |
| supercont2.1459 | 1 | 1084 | unassigned |  |  |  |
| supercont2.1460 | 1 | 1079 | unassigned |  |  |  |
| supercont2.1455 | 1 | 1072 | unassigned |  |  |  |
| supercont2.1461 | 1 | 1072 | unassigned |  |  |  |
| supercont2.1426 | 1 | 1068 | unassigned |  |  |  |
| supercont2.1462 | 1 | 1058 | unassigned |  |  |  |
| supercont2.1439 | 1 | 1056 | unassigned |  |  |  |
| supercont2.1433 | 1 | 1053 | unassigned |  |  |  |
| supercont2.1454 | 1 | 1043 | unassigned |  |  |  |
| supercont2.1458 | 1 | 1040 | unassigned |  |  |  |
| supercont2.1456 | 1 | 1033 | unassigned |  |  |  |
| supercont2.1463 | 1 | 1028 | unassigned |  |  |  |

**Supplementary File 1D**

**Suspected Chimeric MIC Supercontigs.**

Twenty MIC supercontigs are suspected to have been mis-assembled, based on inconsistencies in the alignment of different parts to MAC scaffolds. For the purpose of constructing MIC chromosome super-assemblies, each of these MIC scaffolds was broken into two or three parts, listed here. Sequences between the endpoints of each fragment could not be definitively assigned to one or the other.

| **Supercontig** | **Fragment** | **Start** | **End** | **Chromosome** |
| --- | --- | --- | --- | --- |
| 2.13 | 5' | 1 | 110117 | 3 |
| 2.13 | middle | 153665 | 900971 | 5 |
| 2.13 | 3' | 909947 | 1694315 | 4 |
| 2.16 | 5' | 1 | 258671 | 1 |
| 2.16 | 3' | 270920 | 1328748 | 3 |
| 2.19 | 5' | 1 | 400681 | 1 |
| 2.19 | middle | 402449 | 479454 | 5 |
| 2.19 | 3' | 479455 | 1216767 | 5 |
| 2.30 | 5' | 1 | 859612 | 1 |
| 2.30 | 3' | 876038 | 916602 | 3 |
| 2.42 | 5' | 1 | 410652 | 5 |
| 2.42 | 3' | 415163 | 723494 | 1 |
| 2.53 | 5' | 1 | 174489 | 2 |
| 2.53 | 3' | 185208 | 597665 | 3 |
| 2.56 | 5' | 1 | 551169 | 3 |
| 2.56 | 3' | 571971 | 581391 | 4 |
| 2.113 | 5' | 1 | 304979 | 1 |
| 2.113 | 3' | 315365 | 344693 | 1 |
| 2.117 | 5' | 1 | 18664 | 2 |
| 2.117 | 3' | 21288 | 332429 | 3 |
| 2.131 | 5' | 1 | 22885 | 3 |
| 2.131 | middle | 29599 | 63832 | 4 |
| 2.131 | 3' | 89626 | 292733 | 5 |
| 2.135 | 5' | 1 | 137134 | 4 |
| 2.135 | 3' | 152670 | 279200 | 2 |
| 2.213 | 5' | 1 | 50069 | 3 |
| 2.213 | 3' | 53380 | 162078 | 2 |
| 2.280 | 5' | 1 | 19220 | 1 |
| 2.280 | 3' | 30253 | 137783 | 4 |
| 2.307 | 5' | 1 | 19998 | 3 |
| 2.307 | 3' | 33735 | 113507 | 3 |
| 2.322 | 5' | 1 | 44851 | 2 |
| 2.322 | 3' | 67495 | 123982 | 4 |
| 2.335 | 5' | 1 | 17061 | 3 |
| 2.335 | 3' | 33721 | 102538 | 3 |
| 2.398 | 5' | 1 | 58653 | 5 |
| 2.398 | 3' | 62418 | 86345 | 4 |
| 2.447 | 5' | 1 | 20504 | 4 |
| 2.447 | 3' | 26492 | 64018 | 4 |
| 2.535 | 5' | 1 | 16128 | 1 |
| 2.535 | 3' | 20016 | 51810 | 2 |
| 2.555 | 5' | 1 | 32602 | 4 |
| 2.555 | 3' | 34271 | 46297 | 4 |

**Supplementary File 1E**

**Most centric MAC chromosomes are among the ten longest MAC chromosomes**

| **MAC chromosome ID** | **MAC chromosome length (kb)** | **Number of scaffolds** | **Average scaffold size (kb)** | **MIC chromo-**  **some** | **Location on MIC chromosome** |
| --- | --- | --- | --- | --- | --- |
| 8254716 | 3,243 | 5 | 648.6 | 2 | 2L-7 – 2L-6 |
| 8254820 | 3,211 | 48 | 66.9 | 5 | 5L-1 – 5R-1 |
| 8254791* | 2,652 | 43 | 61.7 | 3 | 3L-1 – 3R-1 |
| 8254810 | 2,473 | 30 | 82.4 | 4 | 4L-1 – 4R-1 |
| 8254803 | 2,412 | 3 | 804.1 | 2 | 2L-2 – 2L-1 |
| 8254467 | 2,170 | 3 | 723.4 | 3 | 3L-6 - 3L-5 |
| 8254824 | 1,908 | 16 | 119.2 | 5 | 5R-1 – 5R-2 |
| 8254822 | 1,878 | 50 | 37.6 | 2 | 2L-1 – 2R-1 |
| 8254798* | 1,583 | 20 | 79.2 | 3 | 3L-3 – 3L-2 |
| 8254826/4759** | 1,579*** | 38 | 41.6 | 1 | 1L-1 – 1R-1 |

Centric MAC chromosomes, derived from the centromeric regions of MIC chromosomes, tend to be large; most (shaded rows) are among the ten largest MAC chromosomes. They also retain more repetitive sequences, making them more difficult to assemble by the whole-genome shotgun approach; as a result, they are composed of numerous small HAPPY-linked scaffolds (column 3; see Supplementary Table 2 for details). The centromeric regions of chromosomes 2, 4, and 5 each engender a single, large, centric MAC chromosome (8254822, 8254810, and 8254820, respectively). Three centric MAC chromosomes (two of them – 8254791 and 8254798 – exceptionally large) derive from the putative centromere region of chromosome 3 (although, because of the low number of 3L deletions available for mapping, the actual number may be lower). The centromere region of chromosome 1 is complex, containing 15 Cbs’s associated with 5 centric MAC chromosomes and 11 NMCs, as well as several sites involved in the joining of non-contiguous MIC regions into MAC chromosomes, as described in the main text. One of the resulting MAC chromosomes (8254826/4759) is among the ten largest.

If the MAC retains any centromeric activity, it should reside on the centric MAC chromosomes, although two lines of cytological evidence argue against this possibility. First, centromeric histone H3 disappears from the differentiating MACs of *Tetrahymena* [27, 40] and *Paramecium* [88] suggesting programmed elimination of centromere-specific sequences. Second, the MAC divides amitotically with no discernable mitotic apparatus. We further tested the possibility of centromeric activity on centric MAC chromosomes using a genetic strategy. The random partitioning of MAC alleles to daughter nuclei during vegetative growth leads to a phenomenon known as "phenotypic assortment" where heterozygous cells produce clonal lineages in which the MAC has become pure for one of the two alleles [110]. We found that DNA polymorphisms on centric MAC chromosomes show normal, random assortment (data not shown). Thus, all available evidence is consistent with the complete absence of centromeric activity in the MAC.

* The putative chromosome 3 centromere spans three MAC chromosomes, including these two.

** This nascent centric MAC chromosome is exceptional, in that it consists of two segments, each of which becomes incorporated into a different mature MAC chromosome, 8254826 and 8254759, by programmed recombination during MAC differentiation (Hamilton & Orias, unpublished information).

*** The length is that of the nascent chromosome; the contributions of each mature MAC chromosome are 875.5 and 703.5 kb, respectively.
