# Supplementary material for: Structure of the germline genome of Tetrahymena thermophila and relationship to the massively rearranged somatic genome
Source: eLife. 2016 Nov 28;5:e19090. doi: 10.7554/eLife.19090 (PMC5182062; doi:10.7554/eLife.19090)
Supplement: Supplementary file 2. — (A) The 225 Cbs identified in the T. thermophila MIC genome. (B) The 181 T. thermophila MAC chromosomes: Lengths and flanking Cbs's. (C) Conservation of chromosome breakage sites 3L-15 to 26 relative to flanking genes in all four examined Tetrahymena species. (D) Identity and lengths of Tetrahymena species scaffolds and supercontigs defined by Cbs's homologous to T. thermophila 3L-26 to 15. (E) T. thermophila Cbs clade alignments. (F) Properties of predicted non-maintained chromosomes. (G) Predicted genes on non-maintained chromosomes. DOI: http://dx.doi.org/10.7554/eLife.19090.026 [file elife-19090-supp2.docx]

**Supplementary File 2A**

**The 225 Cbs identified in the *T. thermophila* MIC genome**

| **Cbs Name ^a^** | **Previous published* Names for Cbs** | **MIC super-contig location** | **Super-contig length** | **Cbs Variant** | **Cbs Orientation relative to** | |
| --- | --- | --- | --- | --- | --- | --- |
|  |  |  |  |  | **MIC super-contig** | **MIC chromosome super-assembly** |
| 1L-1 |  | 2.264:54962 | 83055 | 11C | Fwd | Fwd |
| 1L-2 |  | 2.264:54867 |  | 11C | Fwd | Fwd |
| 1L-3 |  | 2.264:54745 |  | consensus | Fwd | Fwd |
| 1L-4 |  | 2.264:54627 |  | 11C | Fwd | Fwd |
| 1L-5 |  | 2.264:54388 |  | 13A | Fwd | Fwd |
| 1L-6 | 1L-9 | 2.46:550486 | 113005 | consensus | Fwd | Fwd |
| 1L-7 | 1L-6 | 2.46:66465 |  | 1A | Fwd | Fwd |
| 1L-8 |  | 2.91:313246 | 81125 | 15A | Fwd | Fwd |
| 1L-9 | 1L-4 | 2.91:204787 |  | consensus | Fwd | Fwd |
| 1L-10 |  | 2.17:79263 |  | 1A | Rev | Fwd |
| 1L-11 | 1L-5 | 2.17:160611 |  | consensus | Rev | Fwd |
| 1L-12 |  | 2.17:911037 |  | 14C | Rev | Fwd |
| 1L-13 |  | 2.17:989150 | 335134 | 1A | Fwd | Rev |
| 1L-14 |  | 2.45:294889 |  | consensus | Fwd | Rev |
| 1L-15 |  | 2.45:641614 | 27291 | consensus | Fwd | Rev |
| 1L-16 | 1L-2 | 2.780:12856 | 780 | consensus | Rev | Fwd |
| 1L-17 | 1L-1 | 2.2:790 |  | consensus | Fwd | Rev |
| 1L-18 | 1L-1 | 2.2:835 |  | consensus | Fwd | Rev |
| 1L-19 | 1L-1 | 2.2:880 |  | 13A | Fwd | Rev |
| 1L-20 | 1L-7 | 2.2:568734 |  | consensus | Fwd | Rev |
| 1L-21 |  | 2.2:1063990 |  | 1A | Rev | Fwd |
| 1L-22 | 1L-3 | 2.2:1946536 |  | consensus | Rev | Fwd |
| 1L-23 |  | 2.2:2472680 |  | 1A | Rev | Fwd |
| 1L-24 |  | 2.2:2961105 | 262249 | 1A | Fwd | Rev |
| 1L-25 |  | 2.9:357753 |  | consensus | Rev | Fwd |
| 1L-26 | 1L-8 | 2.9:1229909 |  | 1A | Fwd | Rev |
| 1L-27 |  | 2.9:1363814 |  | 15A | Rev | Fwd |
| 1L-28 |  | 2.102:127945 |  | consensus | Rev | Fwd |
| 1L-29 |  | 2.102:128475 | 247705 | consensus | Rev | Fwd |
| 1L-30 |  | 2.365:18515 | 74349 | 11C | Fwd | Rev |
| 1R-1 |  | 2.221:91868 |  | consensus | Rev | Rev |
| 1R-2 |  | 2.221:103322 |  | consensus | Rev | Rev |
| 1R-3 |  | 2.221:115992 |  | consensus | Rev | Rev |
| 1R-4 |  | 2.221:135411 |  | consensus | Rev | Rev |
| 1R-5 |  | 2.221:141052 |  | consensus | Rev | Rev |
| 1R-6 | 1R-6 | 2.221:159996 | 46 | consensus | Rev | Rev |
| 1R-7 | 1R-2 | 2.190:131239 | 52189 | consensus | Fwd | Rev |
| 1R-8 |  | 2.257:94160 | 44410 | consensus | Fwd | Fwd |
| 1R-9 |  | 2.247:135646 |  | 1A | Fwd | Fwd |
| 1R-10 |  | 2.247:135975 | 6933 | 11C | Fwd | Fwd |
| 1R-11 |  | 2.105:79816 |  | consensus | Rev | Rev |
| 1R-12 | 1R-7 | 2.105:163618 | 204168 | consensus | Rev | Rev |
| 1R-13 |  | 2.67:58835 | 436558 | consensus | Rev | Fwd |
| 1R-14 |  | 2.3:39305 |  | 14A | Rev | Rev |
| 1R-15 | 1R-8 | 2.3:385919 |  | consensus | Rev | Rev |
| 1R-16 | 1R-9 | 2.3:1556258 |  | 1A,15A | Fwd | Fwd |
| 1R-17 |  | 2.3:2726246 |  | 1A | Rev | Rev |
| 1R-18 | 1R-1 | 2.3:2843525 |  | consensus | Rev | Rev |
| 1R-19 |  | 2.3:2895431 | 78024 | 1A | Rev | Rev |
| 1R-20 |  | 2.57:127032 | 446855 | 15A | Rev | Fwd |
| 1R-21 |  | 2.313:85419 | 23852 | 1A,11C | Fwd | Fwd |
| 1R-22 |  | 2.8:553372 |  | 1A,13C | Fwd | Fwd |
| 1R-23 |  | 2.8:796901 |  | 13A | Rev | Rev |
| 1R-24 |  | 2.8:974074 |  | 1A | Fwd | Fwd |
| 1R-25 |  | 2.8:1094941 |  | 1A | Rev | Rev |
| 1R-26 |  | 2.8:1642998 |  | 1A | Fwd | Fwd |
| 1R-27 |  | 2.8:2211640 |  | 15A | Rev | Rev |
| 1R-28 |  | 2.8:2343932 | 26276 | consensus | Rev | Rev |
| 1R-29 |  | 2.30:679862 | 236741 | 1A | Rev | Fwd |
| 1R-30 |  | 2.30:676480 |  | 1A | Rev | Fwd |
| 1R-31 |  | 2.30:564783 |  | 1A | Fwd | Rev |
| 1R-32 |  | 2.14:63050 |  | 14A | Fwd | Fwd |
| 1R-33 |  | 2.14:127295 |  | consensus | Fwd | Fwd |
| 1R-34 | 1R-3 | 2.14: 365429 ^b^ |  | 15A | Fwd | Rev |
| 1R-35 |  | 2.14:506339 |  | 1A,11C | Fwd | Fwd |
| 1R-36 |  | 2.14:506808 |  | 1A,11C | Fwd | Fwd |
| 1R-37 |  | 2.14:965437 |  | 1A | Fwd | Fwd |
| 1R-38 |  | 2.14:966560 |  | 1A,11C | Fwd | Fwd |
| 1R-39 |  | 2.14:1355849 |  | 1A | Fwd | Fwd |
| 1R-40 |  | 2.14:1443993 | 45864 | consensus | Fwd | Fwd |
| 1R-41 | 1R-5 | 2.48:386652 |  | consensus | Fwd | Fwd |
| 1R-42 | 1R-4 | 2.48:459056 | 196756 | consensus | Rev | Rev |
| 2L-1 |  | 2.593:17880 | 16278 | 1A | Rev | Fwd |
| 2L-2 |  | 2.92:204495 | 189355 | 1A | Fwd | Rev |
| 2L-3 | 2R-1 | 2.23:833181 | 195327 | consensus | Fwd | Fwd |
| 2L-4 |  | 2.23:552953 |  | 1A,15A | Fwd | Fwd |
| 2L-5 |  | 2.23:176196 |  | consensus | Fwd | Fwd |
| 2L-6 |  | 2.336:62078 | 42641 | 1A | Rev | Fwd |
| 2L-7 |  | 2.189:20008 | 167704 | consensus | Fwd | Fwd |
| 2R-1 |  | 2.240:40779 |  | 14A | Fwd | Fwd |
| 2R-2 |  | 2.240:41384 | 104637 | 14A | Fwd | Fwd |
| 2R-3 |  | 2.279:8224 | 116182 | 1A | Rev | Fwd |
| 2R-4 |  | 2.195:60714 ^b^ |  | 1A | Rev | Fwd |
| 2R-5 | 2R-2 | 2.28:383445 | 605802 | 14A,15A | Rev | Fwd |
| 2R-6 |  | 2.226:22545 | 140602 | consensus | Fwd | Fwd |
| 2R-7 |  | 2.168:177860 | 40458 | 13C | Fwd | Rev |
| 2R-8 |  | 2.308:16580 | 96951 | consensus | Rev | Rev |
| 2R-9 |  | 2.90:143214 | 257187 | 1A,15A | Rev | Fwd |
| 2R-10 |  | 2.90:22128 |  | 1A | Fwd | Rev |
| 2R-11 |  | 2.60:33826 | 517040 | consensus | Fwd | Rev |
| 3L-1 |  | 2.499:36783 | 17014 | 14A | Rev | Rev |
| 3L-2 |  | 2.154:18676 | 231264 | 13A | Fwd | Rev |
| 3L-3 | 3L-6 | 2.62:203451 | 342468 | consensus | Rev | Rev |
| 3L-4 | 3L-4 | 2.62:196687 |  | consensus | Fwd | Fwd |
| 3L-5 |  | 2.62:25744 |  | consensus | Rev | Rev |
| 3L-6 | 3L-8 | 2.5:568687 |  | consensus | Rev | Fwd |
| 3L-7 |  | 2.5:845939 |  | 11C | Fwd | Rev |
| 3L-8 |  | 2.5:1322772 |  | 1A | Fwd | Rev |
| 3L-9 | 3L-2 | 2.5:1644915 |  | consensus | Rev | Fwd |
| 3L-10 |  | 2.5:1900037 |  | 1A,15A | Rev | Fwd |
| 3L-11 | 3L-7 | 2.5:2001381 |  | consensus | Rev | Fwd |
| 3L-12 |  | 2.5:2258872 |  | consensus | Fwd | Rev |
| 3L-13 |  | 2.5:2317012 | 209582 | 15A | Rev | Fwd |
| 3L-14 |  | 2.111:272602 | 76368 | consensus | Fwd | Fwd |
| 3L-15 | 3L-1 | 2.11:461987 |  | consensus | Rev | Fwd |
| 3L-16 |  | 2.11:698735 |  | 1A | Rev | Fwd |
| 3L-17 |  | See footnote b |  | consensus | Rev | Fwd |
| 3L-18 |  | 2.11:1449320 |  | 1A | Fwd | Rev |
| 3L-19 |  | 2.11:1552096 |  | consensus | Rev | Fwd |
| 3L-20 |  | 2.11:1677873 |  | 1A | Rev | Fwd |
| 3L-21 |  | 2.11:1908264 | 74993 | consensus | Rev | Fwd |
| 3L-22 | 3L-11 | 2.6:1355781 | 1132313 | 1A,13A | Rev | Rev |
| 3L-23 |  | 2.6:1036417 |  | consensus | Rev | Rev |
| 3L-24 | 3L-5 | 2.6:742918 |  | consensus | Fwd | Fwd |
| 3L-25 |  | 2.6:542061 |  | 1A,11C | Rev | Rev |
| 3L-26 |  | 2.6:400775 |  | consensus | Fwd | Fwd |
| 3L-27 |  | 2.294:96050 |  | 14C | Rev | Fwd |
| 3L-28 |  | 2.294:103015 | 14256 | consensus | Fwd | Rev |
| 3L-29 | 3L-3 | 2.292:110863 | 6839 | consensus | Rev | Fwd |
| 3R-1 | 3R-2 | 2.81:118760 | 331672 | consensus | Rev | Fwd |
| 3R-2 |  | 2.110:156628 | 194603 | consensus | Fwd | Rev |
| 3R-3 |  | 2.18:729722 |  | consensus | Fwd | Fwd |
| 3R-4 | 3R-3 | 2.18:828858 |  | consensus | Fwd | Fwd |
| 3R-5 |  | 2.18:926961 | 335269 | consensus | Rev | Rev |
| 3R-6 |  | 2.56:371052 | 210340 | 14A | Rev | Fwd |
| 3R-7 | 3R-4 | 2.53:591384 | 6282 | 1A | Rev | Fwd |
| 3R-8 |  | 2.4:1429592 |  | consensus | Rev | Rev |
| 3R-9 |  | 2.4:1518837 | 1121094 | consensus | Fwd | Fwd |
| 3R-10 |  | 2.289:104960 | 13958 | consensus | Rev | Rev |
| 3R-11 | 3R-5 | 2.16:641608 | 687141 | 14A | Fwd | Fwd |
| 3R-12 |  | 2.37:649460 | 122212 | consensus | Rev | Fwd |
| 3R-13 |  | 2.37:386995 |  | 1A | Rev | Fwd |
| 3R-14 | 3R-1 | 2.71:299693 | 188717 | consensus | Fwd | Fwd |
| 3R-15 |  | 2.248:110965 | 31544 | consensus | Rev | Rev |
| 4L-1 | 4L-2 | 2.173:15039 | 192970 | consensus | Rev | Rev |
| 4L-2 |  | 2.310:109444 | 1082 | consensus | Fwd | Fwd |
| 4L-3 |  | 2.310:105624 |  | consensus | Fwd | Fwd |
| 4L-4 |  | 2.140:49269 | 233107 | 13A | Rev | Rev |
| 4L-5 | 4L-4 | 2.40:681950 | 65170 | consensus | Fwd | Fwd |
| 4L-6 | 4L-6 | 2.1:309341 |  | consensus | Fwd | Rev |
| 4L-7 |  | 2.1:1103998 |  | consensus | Rev | Fwd |
| 4L-8 |  | 2.1:1262639 |  | 15A | Rev | Fwd |
| 4L-9 | 4L-10 | 2.1:1934265 |  | 14A | Rev | Fwd |
| 4L-10 |  | 2.1:3008984 |  | 1A | Rev | Fwd |
| 4L-11 | 4L-3 | 2.1:3011421 | 531730 | 1A,14C | Rev | Fwd |
| 4L-12 |  | 2.139:191967 | 83353 | consensus | Rev | Fwd |
| 4L-13 | 4L-5 | 2.403:52172 | 27286 | 1A | Fwd | Fwd |
| 4L-14 |  | 2.51:557007 | 58983 | 15A | Rev | Fwd |
| 4L-15 |  | 2.96:300808 | 82872 | consensus | Fwd | Fwd |
| 4L-16 |  | 2.96:35360 |  | 11C | Rev | Fwd |
| 4L-17 |  | 2.471:880 | 60288 | 1A | Rev | Rev |
| 4R-1 |  | 2.143:74227 | 202228 | 15A | Fwd | Fwd |
| 4R-2 |  | 2.125:246045 | 53651 | 14A | Fwd | Rev |
| 4R-3 |  | 2.337:80043 |  | 1A | Rev | Rev |
| 4R-4 |  | 2.337:96313 | 5194 | consensus | Rev | Rev |
| 4R-5 |  | 2.273:125932 | 2269 | consensus | Fwd | Rev |
| 4R-6 |  | 2.273:110096 |  | consensus | Fwd | Rev |
| 4R-7 |  | 2.273:90981 |  | consensus | Fwd | Rev |
| 4R-8 |  | 2.15:1358421 | 68629 | consensus | Rev | Fwd |
| 4R-9 | 4R-6 | 2.15:889655 |  | 11C,13A | Fwd | Rev |
| 4R-10 |  | 2.15:780776 |  | 1A | Fwd | Rev |
| 4R-11 |  | 2.15:155531 |  | 1A,15A | Rev | Fwd |
| 4R-12 |  | 2.12:1487336 | 259436 | 11C | Fwd | Rev |
| 4R-13 |  | 2.12:1194978 |  | 1A,14C | Rev | Fwd |
| 4R-14 | 4R-5 | 2.12:1140061 |  | 11C,13G | Rev | Fwd |
| 4R-15 | 4R-2 | 2.12:937345 |  | 11C,15A | Fwd | Rev |
| 4R-16 |  | 2.12:722246 |  | 1A | Rev | Fwd |
| 4R-17 |  | 2.12:631684 |  | 1A | Fwd | Rev |
| 4R-18 | 4R-4 | 2.12:51758 |  | consensus | Rev | Fwd |
| 4R-19 |  | 2.7:37887 |  | 1A | Rev | Rev |
| 4R-20 |  | 2.7:294411 |  | 14C | Rev | Rev |
| 4R-21 |  | 2.7:849346 |  | 1A | Fwd | Fwd |
| 4R-22 |  | 2.7:891652 |  | consensus | Rev | Rev |
| 4R-23 |  | 2.7:2096148 |  | consensus | Fwd | Fwd |
| 4R-24 |  | 2.7:2230707 |  | 13A | Rev | Rev |
| 4R-25 |  | 2.7:2231363 | 173753 | 13A | Fwd | Fwd |
| 4R-26 |  | 2.27:11865 |  | consensus | Fwd | Fwd |
| 4R-27 |  | 2.27:679756 | 280020 | consensus | Rev | Rev |
| 4R-28 |  | 2.27:821594 | 138182 | consensus | Fwd | Fwd |
| 4R-29 |  | 2.75:261965 |  | consensus | Rev | Rev |
| 4R-30 |  | 2.75:273702 | 201034 | 1A,15A | Rev | Rev |
| 4R-31 |  | 2.20:1136185 | 20263 | 1A,15A | Fwd | Rev |
| 4R-32 | 4R-1 | 2.20:1081157 ^b^ |  | Consensus | Rev | Fwd |
| 4R-33 | 4R-7 | 2.20:292513 |  | consensus | Rev | Fwd |
| 4R-34 |  | 2.20:146847 |  | 1A,13A | Fwd | Rev |
| 4R-35 |  | 2.20:146052 |  | 1A | Fwd | Rev |
| 4R-36 | 4R-3 | 2.13:1102450 | 591866 | 11C,14C | Fwd | Fwd |
| 4R-37 |  | 2.66:430980 | 76118 | consensus | Rev | Fwd |
| 4R-38 |  | 2.341:49463 | 53828 | consensus | Fwd | Fwd |
| 5L-1 | 5-6 | 2.350:79503 | 18152 | 1A | Rev | Fwd |
| 5L-2 |  | 2.378:9329 | 82261 | consensus | Fwd | Fwd |
| 5L-3 | 5-5 | 2.21:392683 | 664087 | 1A | Rev | Rev |
| 5L-4 |  | 2.61:155625 |  | 1A | Fwd | Rev |
| 5L-5 |  | 2.61:336262 | 213183 | 1A | Rev | Fwd |
| 5L-6 |  | 2.25:760307 | 245517 | 13C | Fwd | Rev |
| 5L-7 |  | 2.22:592984 |  | consensus | Rev | Fwd |
| 5L-8 | 5-3 | 2.22:942525 | 78197 | consensus | Rev | Fwd |
| 5L-9 |  | 2.647:4280 | 22342 | consensus | Rev | Fwd |
| 5L-10 |  | 2.78:14300 |  | consensus | Fwd | Rev |
| 5L-11 | 5-4 | 2.78:24675 | 435499 | 15A | Rev | Fwd |
| 5L-12 |  | 2.49:411679 | 248710 | 1A | Rev | Fwd |
| 5L-13 |  | 2.399:46268 | 32754 | 1A | Rev | Fwd |
| 5L-14 | 5-7 | 2.73:327415 | 168434 | consensus | Rev | Fwd |
| 5L-15 |  | 2.179:40056 | 154283 | 1A | Fwd | Fwd |
| 5L-16 |  | 2.669:18033 | 6349 | consensus | Fwd | Fwd |
| 5R-1 |  | 2.94:85242 | 300517 | 1A | Fwd | Rev |
| 5R-2 |  | 2.166:165278 | 56872 | 1A | Fwd | Rev |
| 5R-3 |  | 2.19:1030175 | 186593 | 1A | Fwd | Fwd |
| 5R-4 | 5-8 | 2.68:119473 | 376250 | consensus | Rev | Fwd |
| 5R-5 | 5-1 | 2.13:288352 ^b^ |  | consensus | Rev | Rev |
| 5R-6 | 5-1 | 2.13: 288658 ^b^ |  | consensus | Rev | Rev |
| 5R-7 |  | 2.13:842931 | 851385 | consensus | Rev | Rev |
| 5R-8 |  | 2.126:18795 | 279413 | 14C | Rev | Rev |
| 5R-9 | 5-2 | 2.44:324650 | 344089 | consensus | Rev | Rev |
| 5R-10 |  | 2.52:621995 | 914 | 1A | Rev | Fwd |
| 5R-11 |  | 2.52:479924 |  | consensus | Fwd | Rev |
| 5R-12 |  | 2.392:42353 | 38797 | consensus | Rev | Fwd |
| 5R-13 |  | 2.130:103752 | 187625 | 1A | Fwd | Rev |
| 5R-14 |  | 2.95:166288 | 216356 | 1A | Fwd | Rev |
| 5R-15 |  | 2.95:166204 |  | 1A,15A | Fwd | Rev |
| 5R-16 |  | 2.109:295716 | 57509 | consensus | Rev | Fwd |
| XX-1 |  | 2.713:3219 | 16327 | consensus | Fwd | ND |
| XX-2 |  | 2.769:8849 | 5575 | consensus | Rev | ND |
| XX-3 |  | 2.796:2989 |  | consensus | Fwd | ND |
| XX-4 |  | 2.800:12070 | 456 | consensus | Fwd | ND |

Explanation of headings

**MIC supercontig location**: Position of the first Cbs nucleotide in the supercontig, based on its orientation as assembled at the Broad Institute.

**Distance to the other supercontig end**. Distance of the first Cbs nucleotide to the opposite end of the supercontig, if only contains a single Cbs. Otherwise, distance of the last (i.e., highest numbered) Cbs to the end of the supercontig.

**Cbs variant**: relative to the consensus Cbs sequence, TAAACCAACCTCTTT

**Cbs Orientation**: orientation of the C-strand of the Cbs sequence relative to the supercontig orientation as assembled at the Broad Institute (left column) and relative to the MIC chromosome super assemblies, as shown at the JBrowser at JCVI (right column). For each MIC chromosome, the number of forward- and reverse-oriented Cbs’s does not significantly differ from a 1:1 ratio, nor does the orientation of consecutive Cbs with respect to one another differ significantly from random, when tandemly repeated Cbs’s are excluded (probability of chi-square > 0.05 in each case).

^a^ Cbs’s XX-1 to 4 are in small MIC2 supercontigs not yet incorporated into MIC chromosome superassemblies but their adjacent sequences show high identity to two extensive tandem repeat clusters. For this reason it is likely that the Cbs’s XX1 and XX-3 pair and the Cbs XX-2 and XX-4 pair are likely to be on MIC chromosome 1R near the Cbs 1R-1 cluster and 4R near the 4R-3 cluster, respectively. Orientation with respect to MIC chromosome superassemblies (ND) cannot yet be determined.

^b^ Cbs missing from MIC assembly 2, assembled using additional information as indicated in main text. In four cases, Cbs 2R-4, 3L-17, 5R-5, and 5R-6, the Cbs’s were located within short sequence gaps. In the other two cases, Cbs 1R-34 and 4R-32, the Cbs region was misassembled in assembly 2 due to incorporation of contaminating MAC telomere sequence.

*[18,51]. Accession numbers for missing Cbs’s: 1R-34 (AY653010, previous name 1R-3); 2R-4 (KU521359); 3L-17 (KU521360); 4R-32 (DQ395115, previous name 4R-1); 5R-5 and 5R-6 (AY653023, previous name 5-1).

**Supplementary File 2B**

**The 181 *T. thermophila* MAC chromosomes: Lengths and flanking Cbs's**

| **MAC Superscaf** | **Cbs pair** | **Approximate length (kb)** |
| --- | --- | --- |
| 8253811 | 5R-8 & 5R-9 | 495 |
| 8253815 | 4R-28 & 4R-29 | 515 |
| 8253817 | 1R-28 & 1R-29 | 193 |
| 8253823 | 2L-5 & 2L-4 | 332 |
| 8253880 | 4R-33 & 4R-34 | 122 |
| 8253886 | 1R-23 & 1R-24 | 150 |
| 8253887 | 4R-37 & 4R-38 | 364 |
| 8253891 | 5L-8 & 5L-7 | 331 |
| 8253894 | 3L-21 & 3L-20 | 215 |
| 8253915 | 1L-15 & 1L-14 | 330 |
| 8253964 | 4R-9 & 4R-10 | 99 |
| 8253974 | 3L-13 & 3L-12 | 55 |
| 8253984 | 1R-33 & 1R-34 | 205 |
| 8254002 | 4R-16 & 4R-17 | 88 |
| 8254010 ^a b^ | 4R-32 & 4R-32 | 682 |
| 8254028 | 1R-38 & 1R-39 | 344 |
| 8254034 | 3L-9 & 3L-8 | 303 |
| 8254043 | 4R-19 & 4R-20 | 250 |
| 8254051 | 4R-14 & 4R-15 | 162 |
| 8254062 | 1R-18 & 1R-19 | 51 |
| 8254072 | 5L-16 & 5L-15 | 37 |
| 8254075 | 1R-32 & 1R-33 | 57 |
| 8254106 | 1R-30 & 1R-31 | 107 |
| 8254122 | 1R-41 & 1R-42 | 58 |
| 8254167 | 3L-25 & 3L-24 | 150 |
| 8254181 | 4R-30 & 4R-31 | 185 |
| 8254185 | 1R-17 & 1R-18 | 114 |
| 8254187 | 4R-11 & 4R-12 | 311 |
| 8254194 | 4R-23 & 4R-24 | 134 |
| 8254199 | 1R-27 & 1R-28 | 126 |
| 8254241 | 3L-29 & 3L-28 | 107 |
| 8254260 | 4R-21 & 4R-22 | 41 |
| 8254284 | 3L-12 & 3L-11 | 256 |
| 8254305 | 4R-2 & 4R-3 | 137 |
| 8254332 | 4R-15 & 4R-16 | 180 |
| 8254359 | 4R-35 & 4R-36 | 222 |
| 8254360 | 1R-39 & 1R-40 | 80 |
| 8254361 | 3R-3 & 3R-4 | 95 |
| 8254362 | 1R-14 & 1R-15 | 275 |
| 8254365 | 3R-6 & 3R-7 | 330 |
| 8254367 | 3L-14 & 3L-13 | 262 |
| 8254370 | 3R-1 & 3R-2 | 256 |
| 8254371 | 1R-12 & 1R-13 | 530 |
| 8254373 | 4L-10 & 4L-9 | 876 |
| 8254377 | 5R-13 & 5R-14 | 298 |
| 8254378 | 3R-5 & 3R-6 | 385 |
| 8254379 | 1L-26 & 1L-25 | 757 |
| 8254381 | 5R-7 & 5R-8 | 229 |
| 8254382 | 5L-5 & 5L-4 | 167 |
| 8254385 | 4R-10 & 4R-11 | 480 |
| 8254392 | 4R-18 & 4R-19 | 83 |
| 8254393 | 5L-9 & 5L-8 | 53 |
| 8254395 | 4R-26 & 4R-27 | 541 |
| 8254396 | 1L-9 & 1L-8 | 99 |
| 8254397 | 3R-12 & 3R-13 | 231 |
| 8254401 | 3L-5 & 3L-4 | 113 |
| 8254402 | 4L-13 & 4L-12 | 355 |
| 8254403 | 4R-17 & 4R-18 | 526 |
| 8254410 | 3L-18 & 3L-17 | 136 |
| 8254412 | 5R-15 & 5R-16 | 427 |
| 8254417 | 1L-16 & 1L-15 | 289 |
| 8254420 | 5R-9 & 5R-10 | 313 |
| 8254422 | 3R-10 & 3R-11 | 319 |
| 8254428 | 1L-24 & 1L-23 | 470 |
| 8254429 | 1R-34 & 1R-35 | 128 |
| 8254431 | 4L-14 & 4L-13 | 1,141 |
| 8254433 | 3L-26 & 3L-25 | 111 |
| 8254436 | 3L-23 & 3L-22 | 265 |
| 8254437 | 3L-17 & 3L-16 | 548 |
| 8254440 | 1R-22 & 1R-23 | 230 |
| 8254444 | 4R-8 & 4R-9 | 400 |
| 8254446 | 1L-20 & 1L-19 | 516 |
| 8254448 | 4L-16 & 4L-15 | 189 |
| 8254449 | 3L-7 & 3L-6 | 252 |
| 8254454 | 3L-11 & 3L-10 | 100 |
| 8254456 | 1L-13 & 1L-12 | 76 |
| 8254457 | 4R-27 & 4R-28 | 137 |
| 8254459 | 1L-23 & 1L-22 | 487 |
| 8254460 | 1L-12 & 1L-11 | 638 |
| 8254464 | 2R-10 & 2R-11 | 406 |
| 8254468 | 4R-25 & 4R-26 | 126 |
| 8254469 | 1R-24 & 1R-25 | 112 |
| 8254470 | 2R-3 & 2R-4 | 1,199 |
| 8254475 | 1L-14 & 1L-13 | 588 |
| 8254476 | 4R-12 & 4R-13 | 275 |
| 8254479 | 1R-19 & 1R-20 | 458 |
| 8254483 | 2L-4 & 2L-3 | 231 |
| 8254486 | 5R-11 & 5R-12 | 431 |
| 8254487 | 1R-25 & 1R-26 | 514 |
| 8254491 | 3L-24 & 3L-23 | 250 |
| 8254495 | 1L-21 & 1L-20 | 427 |
| 8254497 | 1L-11 & 1L-10 | 70 |
| 8254498 | 4R-13 & 4R-14 | 49 |
| 8254503 | 1R-20 & 1R-21 | 182 |
| 8254504 | 3L-19 & 3L-18 | 98 |
| 8254505 | 1L-27 & 1L-26 | 130 |
| 8254517 | 3L-20 & 3L-19 | 116 |
| 8254524 | 3L-2 & 3L-1 | 270 |
| 8254527 | 4R-20 & 4R-21 | 520 |
| 8254544 | 3L-10 & 3L-9 | 212 |
| 8254545 | 1R-15 & 1R-16 | 1,045 |
| 8254548 | 4L-7 & 4L-6 | 714 |
| 8254551 | 1L-28 & 1L-27 | 719 |
| 8254552 | 3L-8 & 3L-7 | 437 |
| 8254555 | 3R-13 & 3R-14 | 596 |
| 8254563 | 1R-36 & 1R-37 | 396 |
| 8254564 | 1L-25 & 1L-24 | 890 |
| 8254565 | 1R-26 & 1R-27 | 527 |
| 8254574 | 3R-4 & 3R-5 | 73 |
| 8254577 ^a^ | 5R-5 & 5R-13 | 348 |
| 8254579 | 5R-6 & 5R-7 | 447 |
| 8254580 | 1L-10 & 1L-9 | 400 |
| 8254582 | 3L-22 & 3L-21 | 1,075 |
| 8254584 | 3L-27 & 3L-26 | 451 |
| 8254587 | 4R-22 & 4R-23 | 1,053 |
| 8254588 | 3L-16 & 3L-15 | 212 |
| 8254589 | 4L-4 & 4L-3 | 429 |
| 8254593 | 4R-36 & 4R-37 | 682 |
| 8254594 | 4L-6 & 4L-5 | 762 |
| 8254595 | 2R-7 & 2R-8 | 153 |
| 8254597 | 5L-13 & 5L-12 | 645 |
| 8254600 | 3R-2 & 3R-3 | 817 |
| 8254607 | 4L-9 & 4L-8 | 558 |
| 8254609 | 1R-31 & 1R-32 | 555 |
| 8254610 | 3L-15 & 3L-14 | 665 |
| 8254611 | 2R-6 & 2R-7 | 522 |
| 8254617 | 3R-9 & 3R-10 | 1,320 |
| 8254622 | 3R-8 & 3R-9 | 73 |
| 8254630 | 1L-7 & 1L-6 | 348 |
| 8254632 | 2R-9 & 2R-10 | 78 |
| 8254637 | 1R-13 & 1R-14 | 275 |
| 8254638 | 3R-11 & 3R-12 | 979 |
| 8254644 | 5L-14 & 5L-13 | 357 |
| 8254645 ^a^ | 5R-4 & 5R-16 | 1,076 |
| 8254649 | 5L-4 & 5L-3 | 820 |
| 8254654 | 1R-10 & 1R-11 | 510 |
| 8254656 | 1L-8 & 1L-7 | 1,423 |
| 8254659 | 3R-7 & 3R-8 | 1,644 |
| 8254664 | 1R-21 & 1R-22 | 502 |
| 8254666 | 4L-15 & 4L-14 | 597 |
| 8254667 | 3L-6 & 3L-5 | 2,170 |
| 8254686 | 5R-2 & 5R-3 | 551 |
| 8254688 | 1L-22 & 1L-21 | 758 |
| 8254691 | 5L-12 & 5L-11 | 852 |
| 8254697 | 3R-14 & 3R-15 | 1,863 |
| 8254709 | 5L-3 & 5L-2 | 1,265 |
| 8254712 | 5L-15 & 5L-14 | 241 |
| 8254716 | 2L-7 & 2L-6 | 3,243 |
| 8254719 | 1R-40 & 1R-41 | 849 |
| 8254747 | 1R-16 & 1R-17 | 1,052 |
| 8254748 | 2L-3 & 2L-2 | 306 |
| 8254749 | 5R-10 & 5R-11 | 103 |
| 8254752 | 4R-7 & 4R-8 | 1,594 |
| 8254757 | 4L-8 & 4L-7 | 149 |
| 8254759 ^a^ | 1R-1 & 1R-9 | 801 |
| 8254763 | 2L-6 & 2L-5 | 427 |
| 8254767 | 4R-1 & 4R-2 | 199 |
| 8254776 | 1L-30 & 1L-29 | 1,454 |
| 8254777 | 4L-12 & 4L-11 | 613 |
| 8254778 | 5L-2 & 5L-1 | 601 |
| 8254786 | 2R-8 & 2R-9 | 1,156 |
| 8254791 | 3L-1 & 3R-1 | 2,652 |
| 8254794 | 1R-7 & 1R-8 | 894 |
| 8254798 | 3L-3 & 3L-2 | 1,583 |
| 8254802 | 2R-5 & 2R-6 | 1,221 |
| 8254803 | 2L-2 & 2L-1 | 2,412 |
| 8254807 | 4L-5 & 4L-4 | 464 |
| 8254810 | 4L-1 & 4R-1 | 2,473 |
| 8254811 | 5L-6 & 5L-5 | 1,389 |
| 8254814 | 5L-7 & 5L-6 | 791 |
| 8254815 | 4L-17 & 4L-16 | 1,387 |
| 8254817 ^a^ | 1L-30 & 1L-5 | 823 |
| 8254820 | 5L-1 & 5R-1 | 3,211 |
| 8254821 | 4L-2 & 4L-1 | 228 |
| 8254822 | 2L-1 & 2R-1 | 1,878 |
| 8254823 | 5R-3 & 5R-4 | 462 |
| 8254824 | 5R-1 & 5R-2 | 1,908 |
| 8254825 | 2R-4 & 2R-5 | 1,210 |
| 8254826 ^a^ | 1L-1 & 1R-42 | 2,166 |
| 8254828 ^a^ | 1L-6 & 2R-2 | 851 |
| rDNA | 1L-17 & 1L-16 | 21 |

^a^ MAC chromosome scrambled in the MIC

^b^ Chromosome breakage at Cbs 4R-32 generates both ends of MAC scaffold 8254010

**Supplementary File 2C**

Conservation of Chromosome Breakage sites 3L-15 to 26 relative to flanking genes in all four examined *Tetrahymena* species.

| **Tetrahymena thermophila** | | | **Other Species Supercontigs** | | |
| --- | --- | --- | --- | --- | --- |
| **Cbs** | **Genes** | **Scaffold** | **T.mal** | **T.ell** | **T.bor** |
|  | 01212840 | 4584 - 5' | 79 - 5' | 46A-3' | 108 - 3' |
|  | 001212840 | 4584 - 5' | 79 - 5' | 46A-3' | 108 - 3' |
| 3L-26 |  |  |  |  |  |
|  | 01085712 | 4433 - 3' | 233 - 3' | 46B-5' | 174 - 3' ^a^ |
|  |  |  |  |  |  |
|  | 01085410 | 4433 - 5' | 233 - 5' | 46B-3' | 174 - 5' |
| 3L-25 |  |  |  |  |  |
|  | 01002750 | 4167 - 5' | 169 - 5' | 46C-5' | 152 - 3' |
|  |  |  |  |  |  |
|  | 001002910 | 4167 - 3' | 231 - 5' | 116 - 3' | 152 - 5' |
| 3L-24 |  |  |  |  |  |
|  | 000787440 | 4491 - 3' | 139 - 3' | 95 - 5' | 130 - 5' |
|  |  |  |  |  |  |
|  | 000785819 | 4491 - 5' | 139 - 5' | 95 - 3' | 130 - 3' |
| 3L-23 |  |  |  |  |  |
|  | 01143830 | 4049 - 5' | 141 - 3' | 89 - 5' ^b^ | 141 - 5' |
|  |  |  |  |  |  |
|  | 00938730 | 4436 - 5' | 141 - 5' | 89 - 3' | 141 - 3' |
| 3L-22 |  |  |  |  |  |
|  | 00131070 | 4582 - 3' | 16 - 3' | 14C - 5' | 18 - 5' |
|  |  |  |  |  |  |
|  | 000139729 | 4582 - 5' | 16 - 5' | 14C - 3' | 18 - 3' |
| 3L-21 |  |  |  |  |  |
|  | 00841180 | 3894 - 5' | 160 - 3' | 14B- 5' | 133 - 3' |
|  |  |  |  |  |  |
|  | 00842720 | 3894 - 3' | 160 - 5' | 14B - 3' | 133 - 5' |
| 3L-20 |  |  |  |  |  |
|  | 001205230 | 4415 - 5' | 276 - 3' | 14 -A1 ^c^ | 172 - 3' |
|  |  |  |  |  |  |
|  | 01326880 | 4517 - 3' | 299 - 3' | 61R - 5' | 172 - 5' |
|  | 001326880 | 4517 - 3' | 299 - 3' | 61R - 5' | 172 - 5' |
| 3L-19 |  |  |  |  |  |
|  | 01109760 | 4504 - 5' | 250 - 3' | 119 - 5' | 171 - 3' |
|  |  |  |  |  |  |
|  | 001109990 | 4504 - 3' | 250 - 5' | 119 - 3' | 171 - 5' |
|  | 01109990 | 4504 - 3' | 250 - 5' | 119 - 3' | 171 - 5' |
| 3L-18 |  |  |  |  |  |
|  | 01015860 | 4410 - 5' | 208 - 3' | 30C - 3' | 160 - 3' |
|  |  |  |  |  |  |
|  | 01016240 | 4410 - 3' | 208 - 5' | 30C - 5' | 160 - 5' |
| 3L-17 |  |  |  |  |  |
|  | 00446569 | 4437 - 3' | 49 - 3' | 30B- 3' | 47 - 5' |
|  | 00446570 | 4437 - 3' | 49 - 3' | 30B- 3' | 47 - 5' |
|  |  |  |  |  |  |
|  | 00444120 | 4437 - 5' | 49 - 5' | 30B - 5' | 47 - 3' |
| 3L-16 |  |  |  |  |  |
|  | 00849190 | 4588 - 5' | 166 - 3' | 30A- 3' | 129 - 5' |
|  |  |  |  |  |  |
|  | 000850632 | 4588 - 3' | 166 - 5' | 30A- 5' | 129 - 3' |
|  | 000850631 | 4588 - 3' | 166 - 5' | 30A- 5' | 129 - 3' |
| 3L-15 |  |  |  |  |  |
|  | 00333210 | 4610 - 5' | 35 - 3' | 14 A-2 ^c^ | 92 - 5' |

^a^ *T. borealis* 174 3' end: Just the *T. borealis* gene immediately adjacent to this CB site has been translocated to a telomere-adjacent location in *T. borealis* supercontig 162. There is no translocation in the three other species.

^b^ *T. elliotti* 89 5' end: ~1.6 kb unrelated DNA sequence has been inserted between the telomere and the nearest conserved flanking gene.

^c^ *T. elliotti* 14A-1 and 14A-2 represent two segments of MAC supercontig 14A which been split by an inversion relative to the three other species (data not shown).

The 5 kb terminal DNA segment of the *T. thermophila* MAC chromosome homologs in three other species were BlastX-aligned with the *Tetrahymena* protein database at the Broad Institute or the *Tetrahymena* Genome Database (TGD; www.ciliate.org), as described under Materials and Methods. BlastX alignment was used to circumvent any potential gene annotation errors in the three other species. Unless qualified in a footnote, the *T. thermophila* genes shown were the best matches in each case.

The column headed by “Genes” gives the matching TTHERM gene identifier. Abbreviations under “Other species”: T.mal, T.ell and T.bor are *Tetrahymena malaccensis*, *elliotti* and *borealis*, respectively. Under “Scaffold” (for T.the) and “supercontig” (for the other species) telomere-adjacent ends matching the *T. thermophila* genes are shown; 5’ and 3’ ends relate to the orientation of assemblies at NCBI or the Broad Institute.

Note that *T. elliotti* supercontigs 14, 30 and 46 each represents a multi-supercontig assembly, linked by blocks of N’s which should have assembled. Individual supercontigs, named with a capital letter suffix (A, B, C, etc.), were dissected by cloning and sequencing the MIC Cbs defining their ends or by finding telomere sequence-containing MAC sequence reads.

As stated in the main text, the retention of a functional Cbs sequence at each of the studied sites, in contrast to the divergence of immediately adjacent sequence, provides evidence that Cbs’s, and therefore the positioning of chromosome breakage sites, are under purifying selection. In other words, it is functionally relevant to maintain the structure of specific MAC chromosomes to preserve particular lengths and/or compositions. With regard to length, selective pressure might conceivably relate to a need for MAC chromosome condensation in preparation for amitotic karyokinesis, to avoid chromatin entanglement and damage (a danger perhaps exacerbated by the exceptionally short doubling time of tetrahymenine ciliates). Due to technical limitations imposed by the polyploidy of MACs, it has not been possible to determine whether or not such condensation actually occurs, but *Tetrahymena* does require condensin proteins for MAC division [26]. It may be possible that those chromosomes that achieve a high condensation ratio can afford greater lengths, while those with lower condensation ratios may need to remain short. Optimal lengths would be conserved by the overall conservation of Cbs number and placement. Loss of a Cbs, generating a longer than optimal chromosome, would be selected against, while there would be no natural selection to protect a new Cbs that splits a chromosome of optimal length in two against future loss by mutational disabling. Indeed, the generation of an additional chromosome species would increase the probability that vegetative cells missing most or every copy of any chromosome type would be generated at cell division [79] and this could provide selection for loss of the new Cbs. Variables affecting the condensation ratio could include the number of genes on the chromosome and their state of expression during amitotic segregation. In this respect, it may be relevant that centric chromosomes are among the longest and also have unusually low gene density (Figure 2, Supplementary Table 5). With regard to chromosome composition, there is some evidence in other eukaryotes for clustering of genes with related expression, regulation, and/or functionality characteristics [73, 100]. Further study may reveal whether any such clustering is evident in *Tetrahymena*. In any case, this organism offers several advantages as a model system in this regard, including the feasibility of coupling or uncoupling MAC linkage by experimentally adding or deleting Cbs sites in the germline genome.

**Supplementary File 2D**

Identity and lengths of *Tetrahymena* species scaffolds and supercontigs defined by Cbs's homologous to *T. thermophila* 3L-26 to 15.

| Cbs | T.the | T.the | T.mal | T.mal | T.ell | T.ell | T.bor | T.bor |
| --- | --- | --- | --- | --- | --- | --- | --- | --- |
| 3L- | Scaf ID | Length (kb) | Scaf ID | Length (kb) | Scaf ID | Length (kb) | Scaf ID | Length (kb) |
| 26 |  |  |  |  |  |  |  |  |
|  | <4433R> | 115 | <233R> | 116 | <46B.. | 107 | <174R> | 105 |
| 25 |  |  |  |  |  |  |  |  |
|  | <4167> | 151 | <169> | 197 | ..46C.. | 11 | <152R> | 173 |
|  |  |  | <231R> | 120 | ..116> | 117 |  |  |
| 24 |  |  |  |  |  |  |  |  |
|  | <4491R> | 251 | <139R> | 261 | <95> | 275 | <130> | 269 |
| 23 |  |  |  |  |  |  |  |  |
|  | <4049.. | 87 | <141R> | 258 | <89> | 287 | <141> | 226 |
|  | ..4436R> | 178 |  |  |  |  |  |  |
| 22 |  |  |  |  |  |  |  |  |
|  | <4582R> | 1,074 | <16R> | 1,047 | <14C> | 1,123 | <18> | 1,016 |
| 21 |  |  |  |  |  |  |  |  |
|  | <3894> | 217 | ..160R> | 216 | <14B.. | 238 | <133R> | 249 |
| 20 |  |  |  |  |  |  |  |  |
|  | <4515.. | 71 | <276R.. | 73 | NA | NA | <172R> | 107 |
|  | ..4517> | 45 | ..299> | 52 | NA | NA |  |  |
| 19 |  |  |  |  |  |  |  |  |
|  | <4504> | 100 | <250R> | 97 | ..119> | 100 | <171R> | 107 |
| 18 |  |  |  |  |  |  |  |  |
|  | <4410> | 139 | <208R> | 140 | <30C R.. | 140 | <160R.. | 144 |
| 17 |  |  |  |  |  |  |  |  |
|  | <4437R> | 548 | <49R> | 556 | ..30B R> | 598 | <47> | 622 |
| 16 |  |  |  |  |  |  |  |  |
|  | <4588> | 214 | ..166R> | 202 | <30A R> | 207 | <129> | 269K |
| 15 |  |  |  |  |  |  |  |  |

MAC chromosome lengths have been rounded off to the nearest 1 kb. NA: not applicable; this chromosome has undergone a rearrangement, most likely due to a MIC inversion (not shown).

Symbols flanking a supercontig name: R: reverse orientation, i.e. match to the minus strand, as shown at the Broad Institute or *Tetrahymena* Genome Database (www.ciliate.org). Telomere-related symbols: angle brackets: telomere sequence exists, whether assembled, or found in unassembled sequence reads or experimentally determined. “..”: telomere not identified.

Note that T. elliotti supercontigs 14, 30 and 46 each represents a multi- supercontig assembly, linked by blocks of N’s where telomeres should have assembled. Individual supercontigs, named with a capital letter suffix (A, B, C, etc.), were dissected by cloning and sequencing the MIC Cbs defining their ends or by finding telomere sequence-containing MAC sequence reads.

**Supplementary File 2E**

*T. thermophila* Cbs clade alignments

| **Cbs ID, variant ^1^** | **bp to next Cbs** | **Query length (bp) ^2^** | **Cbs and alignment statistics** | | | | | | | | | |  |
| --- | --- | --- | --- | --- | --- | --- | --- | --- | --- | --- | --- | --- | --- |
| **Clade 1L-1** |  |  | **1L-1** | **1L-2** | **1L-3** | **1L-4** | **1L-5** |  |  |  |  |  | |
| 1L-1, 11C | 95 | 94 | 170 bt  3E-47 | 109 bt  9E-29 | 88 bt  4E-22 | 90 bt  8E-23 | 97 bt  6E-25 |  |  |  |  |  | |
| 1L-2, 11C | 122 | 120 | 83 bp  89%,0% | 217 bt  3E-61 | 161 bt  2E-44 | 154 bt  3E-42 | 147 bt  5E-40 |  |  |  |  |  | |
| 1L-3, cons | 118 | 122 | 119 bp  77%,8% | 122 bp  89%,3% | 221 bt  3E-62 | 174 bt  3E-48 | 149 bt  1E-40 |  |  |  |  |  | |
| 1L-4, 11C ^3^ | 239 | 118 | 82 bp  84%,0% | 118 bp  99% bp  0% | 122 bp  92%,3% | 214 bt  4E-60 | 141 bt  2E-38 |  |  |  |  |  | |
| 1L-5, 13A |  | 118 | 90 bp  84%,1% | 116 bp  88%,0% | 120 bp  88%,3% | 116 bp  87%,0% | 214 bt  4E-60 |  |  |  |  |  | |
|  |  |  |  |  |  |  |  |  |  |  |  |  | |
| **Clade 1L-16** |  |  | **1L-16** | **4R-24** |  |  |  |  |  |  |  |  | |
| 1L-16, cons | NA | 415 | 749 bt  0.0 | 172 bt  4E-47 |  |  |  |  |  |  |  |  | |
| 4R-24, 13A |  |  | 245 bp  78%,6% | 749 bt  0.0 |  |  |  |  |  |  |  |  | |
|  |  |  |  |  |  |  |  |  |  |  |  |  | |
| **Clade 1L-17 ^4^** |  |  | **1L-17** | **1L-18** | **1L-19** |  |  |  |  |  |  |  | |
| 1L-17, cons | 45 | 45 | 90 bt  4E-23 | 74 bt  2E-18 | 28 bt  1E-04 |  |  |  |  |  |  |  | |
| 1L-18, cons | 45 | 45 | 45 bp  96%,0% | 90 bt  4E-23 | 28 bt  1E-04 |  | **Cbs** | **Query length** | **1L-17 to 1L-19** | **4R-25** |  |  | |
| 1L-19, 13A |  | 21 | 14  100%,0% | 14  100%,0% | 42 bt  3E-09 |  | 1L-17 to 1L-19 | 989 bp | 1784 bt  0.0 | 186 bt  6E-46 |  |  | |
| 4R-25, 13A |  | 415 |  |  |  |  | 4R-25 | 415 bp | 403 bp  71%,13% | 749 bt  0.0 |  |  | |
|  |  |  |  |  |  |  |  |  |  |  |  |  | |
| **Clade 1L-20 ^5^** |  |  | **1L-20** | **3L-14** |  |  |  |  |  |  |  |  | |
| 1L-20, cons | NA | 415 | 749 bt  0.0 | 63,6E-14 |  |  |  |  |  |  |  |  | |
| 3L-14, cons |  | 415 | 57 bp  84%,0% | 749 bt  0.0 |  |  |  |  |  |  |  |  | |
|  |  |  |  |  |  |  |  |  |  |  |  |  | |
| **Clade 1L-28** |  |  | **1L-28** | **1L-29** |  |  |  |  |  |  |  |  | |
| 1L-28, cons | 530 | 415 | 749 bt  0.0 | 77 bt  3E-18 |  |  |  |  |  |  |  |  | |
| 1L-29, cons |  | 415 | 124 bp  76%,9% | 749 bt  0.0 |  |  |  |  |  |  |  |  | |
|  |  |  |  |  |  |  |  |  |  |  |  |  | |
| **Clade 1R-1** |  |  | **1R-1** | **1R-2** | **1R-3** | **1R-4** | **1R-5** | **1R-6** | **1R-7** | **XX-1** | **XX-3** | **2L-2** | |
| 1R-1, cons | 11,454 | 415 | 749 bt  0.0 | 502 bt  2E-146 | 315 bt  6E-90 | 295 bt  5E-84 | 340 bt  1E-97 | 242 bt  3E-68 | 351 bt  8E-101 | 262 bt  3E-74 | 282 bt  3E-80 |  | |
| 1R-2, cons | 12,670 | 415 | 417 bp  88%,4% | 749 bt  0.0 | 331 bt  7E-95 | 315 bt  6E-90 | 324 bt  1E-92 | 226 bt  2E-63 | 331 bt  7E-95 | 248 bt  7E-70 | 255 bt  5E-72 |  | |
| 1R-3, cons | 19,419 | 415 | 382 bp  81%,7% | 386 bp  81%,5% | 749 bt  0.0 | 448 bt  4E-130 | 309 bt  2E-88 | 210 bt  2E-58 | 300 bt  1E-85 | 230 bt  2E-64 | 230 bt  2E-64 |  | |
| 1R-4, cons | 5,641 | 415 | 418 bp  78%,8% | 421 bp  79%,6% | 403 bp  86%,5% | 749 bt  0.0 | 327 bt  9E-94 | 199 bt  3E-55 | 309 bt  2E-88 | 248 bt  7E-70 | 223 bt  3E-62 |  | |
| 1R-5, cons | 18,944 | 415 | 413 bp  81%,6% | 422 bp  79%,6% | 393 bp  78%,8% | 408 bp  79%,7% | 749 bt  0.0 | 461 bt  6E-134 | 365 bt  4E-105 | 522 bt  2E-152 | 497 bt  8E-145 |  | |
| 1R-6, cons | >52,200 | 415 | 378 bp  76%,8% | 387 bp  74%,7% | 372 bp  74%,10% | 382 bp  73%,10% | 381 bp  87%,4% | 749 bt  0.0 | 284 bt  9E-81 | 609 bt  2E-178 | 601 bt  3E-176 | 66 bt  5E-15 | |
| 1R-7, cons | NA | 415 | 423 bp  81%,9% | 423 bp  78%,11% | 390 bp  77%,9% | 411 bp  78%,9% | 414 bp  80%,7% | 417 bp  76%,6% | 749 bt  0.0 | 338 bt  5E-97 | 302 bt  4E-86 |  | |
| XX-1, cons | NA | 415 | 378 bp  78%,8% | 393 bp  76%,7% | 372 bp  75%,8% | 387 bp  75%,9% | 383 bp  91%,3% | 416 bp  93%,2% | 419 bp  79%,6% | 749 bt  0.0 | 592 bt  1E-173 | 88 bt  1E-21 | |
| XX-3, cons | NA | 415 | 420 bp  77%,8% | 384 bp  76%,6% | 380 bp  75%,9% | 374 bp  74%,7% | 373 bp  90%,1% | 417 bp  93%,3% | 416 bp  77%,8% | 416 bp  92%,4% | 749 bt  0.0 |  | |
| 2L-2, 1A |  |  |  |  |  |  |  | 346 bp  67%,13% | 340 bp  69%,11% | 749 bt  0.0 |  |  | |
|  |  |  |  |  |  |  |  |  |  |  |  |  | |
| **Clade 1R-35** |  |  | **1R-35** | **1R-36** |  |  |  |  |  |  |  |  | |
| 1R-35, 1A,11C | 469 | 415 | 749 bt  0.0 | 479 bt  2E-139 |  |  |  |  |  |  |  |  | |
| 1R-36, 1A,11C |  | 415 | 418 bp  86%,7% | 749 bt  0.0 |  |  |  |  |  |  |  |  | |
|  |  |  |  |  |  |  |  |  |  |  |  |  | |
| **Clade 1R-37** |  |  | **1R-37** | **1R-38** |  |  |  |  |  |  |  |  | |
| 1R-37, 1A | 1,123 | 415 | 749 bt  0.0 | 77 bt  3E-18 |  |  |  |  |  |  |  |  | |
| 1R-38, 1A,11C |  | 415 | 150 bp  74%,9% | 749 bt  0.0 |  |  |  |  |  |  |  |  | |
|  |  |  |  |  |  |  |  |  |  |  |  |  | |
| **Clade 2R-1** |  |  | **2R-1** | **2R-2** |  |  |  |  |  |  |  |  | |
| 2R-1, 14A | 605 | 415 | 749 bt  0.0 | 237 bt  1E-66 |  |  |  |  |  |  |  |  | |
| 2R-2, 14A |  | 415 | 291 bp  80%,7% | 749 bt  0.0 |  |  |  |  |  |  |  |  | |
|  |  |  |  |  |  |  |  |  |  |  |  |  | |
| **Clade 3L-3** |  |  | **3L-3** | **3L-29** |  |  |  |  |  |  |  |  | |
| 3L-3, cons | NA | 415 | 749 bt  0.0 | 598 bt  3E-175 |  |  |  |  |  |  |  |  | |
| 3L-29, cons |  | 415 | 400 bp  94%,4% | 749 bt  0.0 |  |  |  |  |  |  |  |  | |
|  |  |  |  |  |  |  |  |  |  |  |  |  | |
| **Clade 3L-4** |  |  | **3L-4** | **4L-2** | **4L-3** |  |  |  |  |  |  |  | |
| 3L-4, cons | NA | 415 | 749 bt  0.0 | 178 bt  1E-48 | 259 bt  4E-73 |  |  |  |  |  |  |  | |
| 4L-2, cons | 3,820 | 415 | 275 bp  76%,6% | 749 bt  0.0 | 300 bt  1E-85 |  |  |  |  |  |  |  | |
| 4L-3, cons |  | 415 | 417 bp  75%,8% | 283 bp  84%,9% | 749 bt  0.0 |  |  |  |  |  |  |  | |
|  |  |  |  |  |  |  |  |  |  |  |  |  | |
| **Clade 4R-3** |  |  | **4R-3** | **4R-4** | **4R-5** | **4R-6** | **4R-7** | **4R-38** | **XX-2** | **XX-4** |  |  | |
| 4R-3, 1A | 16,270 | 415 | 749 bt  0.0 | 338 bt  5E-97 | 199 bt  3E-55 | 239 bt  4E-67 | 228 bt  6E-64 | 264 bt  9E-75 | 311 bt  7E-89 | 219 bt  3E-61 |  |  | |
| 4R-4, cons | >74,600 | 415 | 257 bp  90%,4% | 749 bt  0.0 | 401 bt  5E-116 | 381 bt  5E-110 | 280 bt  1E-79 | 446 bt  1E-129 | 435 bt  2E-126 | 394 bt  7E-114 |  |  | |
| 4R-5, cons | 15,836 | 415 | 249 bp  79%,9% | 421 bp  82%,9% | 749 bt  0.0 | 563 bt  7E-165 | 430 bt  1E-124 | 347 bt  9E-100 | 179 bt  3E-49 | 619 bt  0.0 |  |  | |
| 4R-6, cons | 19,115 | 415 | 240 bp  83%,4% | 418 bp  81%,6% | 421 bp  91%,5% | 749 bt  0.0 | 441 bt  6E-128 | 381 bt  5E-110 | 221 bt  9E-62 | 583 bt  7E-171 |  |  | |
| 4R-7, cons | NA | 415 | 248 bp  81%,8% | 338 bp  78%,12% | 417 bp  82%,15% | 417 bp  83%,13% | 749 bt  0.0 | 280 bt  1E-79 | 205 bt  7E-57 | 455 bt  3E-132 |  |  | |
| 4R-38, cons | NA | 415 | 258 bp  84%,7% | 423 bp  84%,5% | 426 bp  78%,11% | 423 bp  81%,8% | 417 bp  75%,16% | 749 bt  0.0 | 297 bt  1E-84 | 381 bt  5E-110 |  |  | |
| XX-2, cons | NA | 415 | 257 bp  88%,4% | 287 bp  94%,1% | 291 bp  75%,12% | 288 bp  77%,8% | 267 bp  78%,6% | 291 bp  83%,5% | 749 bt  0.0 | 212 bt  5E-59 |  |  | |
| XX-4, cons |  | 415 | 254 bp  80%,9% | 424 bp  81%,9% | 416 bp  93%,5% | 418 bp  92%,4% | 421 bp  84%,13% | 378 bp  83%,6% | 242 bp  80%,11% | 749 bt  0.0 |  |  | |
|  |  |  |  |  |  |  |  |  |  |  |  |  | |
| **Clade 5L-9** |  |  | **5L-9** | **5L-10** | **5L-11** |  |  |  |  |  |  |  | |
| 5L-9, cons | >18,600 | 415 | 749 bt  0.0 | 269 bt  2E-76 | 262 bt  3E-74 |  |  |  |  |  |  |  | |
| 5L-10, cons | 10,375 | 415 | 299 bp  80%,7% | 749 bt  0.0 | 210 bt  2E-58 |  |  |  |  |  |  |  | |
| 5L-11, 15A |  | 415 | 418 bp  74%,12% | 309 bp  74%,15% | 749 bt  0.0 |  |  |  |  |  |  |  | |
|  |  |  |  |  |  |  |  |  |  |  |  |  | |
| **Clade 5R-5** |  |  | **5R-5** | **5R-6** |  |  |  |  |  |  |  |  | |
| 5R-5, cons |  | 53 | 97 bt  3E-25 | 88 bt  2E-22 |  |  |  |  |  |  |  |  | |
| 5R-6, cons |  | 53 | 51 bp  91%,0% | 97 bt  3E-25 |  |  |  |  |  |  |  |  | |
|  |  |  |  |  |  |  |  |  |  |  |  |  | |
| **Clade 5R-14 ^6^** |  |  | **5R-14** | **5R-15** |  |  |  |  |  |  |  |  | |
| 5R-14, 1A | 84 | 58 | 105 bt  6E-28 | 61 bt  2E-14 |  |  |  |  |  |  |  |  | |
| 5R-15, 1A,15A |  | 60 | 61 bp  86%,7% | 109 bt  5E-29 |  |  |  |  |  |  |  |  | |
|  |  |  |  |  |  |  |  |  |  |  |  |  | |

Table organization: For every clade table, self-alignments are represented by yellow-highlighted cells in the main diagonal. Every non-self alignment is represented by two cells, located symmetrically with respect to the main diagonal. The cell in the upper triangle shows bit score (bt) and expect value, while the cell in the lower triangle lists the corresponding alignment length (bp), followed by the % identity and % gaps. For self-alignments, only the bit score and expect value are explicitly shown; the alignment length equals the query length, and % identity and %gaps are 100 and 0, respectively. Methods are described in the Materials and Methods section.

^1^ Variant indicates the nucleotide and position at which the Cbs differs from the consensus

(cons) Cbs TAAACCAACCTCTTT. Coordinates of every Cbs are listed in Suppl. Table 6.

Each clade is named by the first listed Cbs.

^2^ Query lengths are less than 415 bp when consecutive Cbs are less than 400 bp apart (cells highlighted in light pink). For those clades, repeat units were parsed and aligned and query length represents the length of the parsed repeat unit.

^3^ An extra repeat unit occurs between Cbs’s 1L-4 and 1L-5, which contains a mutationally disabled Cbs (see Supplementary Text).

^4^ Left sub-table: parsed Cbs 1L-17 to 1L-19 repeat units aligned with one another. Right sub-table: a 995-bp segment, containing all three closely spaced 1L-17 to 1L-19 Cbs’s plus 200 bp of flanking DNA sequence on each side, aligned with the 415-bp Cbs 4R-25 segment. The Cbs 1L-19-adjacent sequence is unrelated to the sequence adjacent to Cbs 1L-17 and 1L-18. However both the sequences adjacent to Cbs 1L-17 5’ and Cbs-19 3’ align with the 4R-25-adjacent sequences. (See the more detailed analysis of 1L-16 and 1L-17 clades in Suppl. Text 1.)

^5^ Clade 1L-20 is less strongly supported. The 6E-14 expect value is well below E-18 threshold for acceptable alignment but at this expect value, real alignments are ~ 3 times more frequent than random ones. This clade was also robustly identified by the Markov clustering algorithm (see Materials and Methods).

^6^ Cluster 5R-14 is less strongly supported. The 2E-14 expect value falls short of the E-18 threshold arrived at using 415 bp segments, but the parsed repeat units are quite short (58 and 60 bp, respectively). The probability of being a legitimate clade is likely strengthened by the Cbs’s being consecutive and sharing the 1A Cbs variant. A 25-bp segment of unrelated sequence separates the two Cbs-containing repeat units.

**Supplementary File 2F**

**Properties of predicted non-maintained chromosomes**

| NMC name | MIC super-contig | Locations of flanking Cbs | NMC length ^a^  (bp) | Clade of Flanking Cbs ^b^ | # genes^c^ | | Gene density ^d^ | |
| --- | --- | --- | --- | --- | --- | --- | --- | --- |
| 1L-2/1 | 264 | 54,867-54,962 | 41 | 1L-1 |  | |  | |
| 1L-3/2 | 264 | 54,745-54,867 | 68 | 1L-1 |  | |  | |
| 1L-4/3 | 264 | 54,627-54,745 | 64 | 1L-1 |  | |  | |
| 1L-5/4 | 264 | 54,388-54,627 | 185 | 1L-1 |  | |  | |
| 1L-18/17 | 2 | 790-835 | 0 | 1L-17 |  | |  | |
| 1L-19/18 | 2 | 835-880 | 0 | 1L-17 |  | |  | |
| 1L-29/28 | 102 | 127,945-128,475 | 476 | 1L-28 |  | |  | |
| 1R-1/2 | 221 | 91,868-103,322 | 11,400 | 1R-1 | 1 | | 0.13 | |
| 1R-2/3 | 221 | 103,322-115,992 | 12,616 | 1R-1 | 1 | | 0.12 | |
| 1R-3/4 | 221 | 115,992-135,411 | 19,365 | 1R-1 | 1 | | 0.08 | |
| 1R-4/5 | 221 | 135,411-141,052 | 5,587 | 1R-1 |  | |  | |
| 1R-5/6 | 221 | 141,052-159,996 | 18,890 | 1R-1 | 1 | | 0.08 | |
| 1R-6/7 ^e^ | 221  190 | 160,010-end (160,041)  1-52,175 | > 52,135 | 1R-1 |  | |  | |
| 1R-9/10 | 247 | 135,646-135,975 | 275 | None |  | |  | |
| 1R-11/12 ^f^ | 105 | 79,816-163,618 | 83,748 | None | 20 | | 0.36 | |
| 1R-29/30 ^g^ | 30 | 676,480-679,862 | 3,328 | None | 1 | | 0.45 | |
| 1R-35/36 | 14 | 506,339-506,808 | 415 | 1R-35 |  | |  | |
| 1R-37/38 | 14 | 965,437-966,560 | 1,069 | 1R-37 |  | |  | |
| 2R-1/2 | 240 | 40,779-41,384 | 551 | 2R-1 |  | |  | |
| 3L-4/3 | 62 | 196,687-203,451 | 6,710 | 3L-4  & 3L-3 |  | |  | |
| 3L-28/27 | 294 | 96,050-103,015 | 6,911 | None | 3 | | 0.65 | |
| 4L-3/2 | 310 | 105,624-109,444 | 3,766 | 3L-4 | 1 | | 0.39 | |
| 4L-11/10 | 1 | 3,008,984-3,011,421 | 2,383 | None |  | |  | |
| 4R-3/4 | 337 | 80,043-96,313 | 16,216 | 4R-3 | 4 | | 0.37 | |
| 4R-4/5 ^e^ | 337  273 | 96,313 – end (101,507)  125,932 – end (128,201) | > 5159 | 4R-3 |  | |  | |
| 4R-5/6 | 273 | 110,096-125,932 | 15,782 | 4R-3 | 5 | | 0.47 | |
| 4R-6/7 | 273 | 90,981-110,096 | 19,061 | 4R-3 | 2 | | 0.16 | |
| 4R-24/25 | 7 | 2,230,707-2,231,363 | 602 | 1L-16 & 1L17 |  | |  | |
| 4R-29/30 | 75 | 261,965-273,702 | 11,683 | None | 2 | | 0.26 | |
| 4R-34/35 | 20 | 146,052-146,847 | 741 | None |  | |  | |
| L-10/9 ^e^ | 78  647 | 1-14,300  4,280-end (26,621) | > 36,566 | 5L-9 | 3 | ≤ 0.12 | |  |
| 5L-11/10 | 78 | 14,300-24,675 | 10,321 | 5L-9 | 2 | 0.29 | |  |
| 5R-14/15 | 95 | 166,204-166,288 | 30 | 5R-14 |  |  | |  |

^a^ Potential NMC lengths are approximate. 40 bp have been subtracted from the distance between flanking Cbs, to correct for the approximate number of base pairs lost on each side of Cbs during chromosome breakage and telomere addition.Thus, potential NMCs that are very short may not be telomerized and therefore immediately degraded. In addition, MAC NMC lengths could be smaller due to IES excision.

^b^ Clade of flanking Cbs: clade membership of the two flanking Cbs’s, as described in Table 4 and Supplementary Table 10. NMCs flanked by tandemly repeated Cbs duplications likely result from recent repeat expansions, as random mutations have not yet obliterated Cbs-adjacent sequence similarity. A few NMCs have flanking Cbs’s belonging to different Cbs clades. Because of the chromosome breakage site conservation described in main text, some of these breakage sites could be old and might be conserved in other *Tetrahymena* species. Alternatively, they may represent cases where a long-range duplication was targeted to the vicinity of a preexisting Cbs.

^c^ NMC predicted genes are described in Supplementary Table 12.

^d^ Gene densities are estimated as # genes / NMC length in kb x 1.5. The 1.5 factor corrects for the average IES fraction in the maintained MAC genome. (IES’s in NMCs remain to be identified, as NMC DNA is absent from the assembled MAC genome).

^e^ NMCs 1R-6/7, 4R-4/5 and 5L-10/9 are composed of segments in two supercontigs that have been joined in the corresponding MIC chromosome super-assemblies. Their MIC lengths are thus minimum estimates. The gaps between 1R-6/7 (MIC 2.221 and MIC 2.190) and 5L-10/9 (MIC 2.78 and MIC 2.647) are bridged by MIC scaffolds from an earlier MIC genome assembly and their length estimates have been corrected accordingly.

^f^ NMC 1R-11/12 is the largest NMC. It includes three (and a partial fourth) tandem copies of an approximately 20 kb sequence (data not shown) each containing predicted genes.

^g^ NMC 1R-29/30 is the smallest predicted gene-containing NMC.

**Supplementary File 2G**

**Predicted Genes on Non-Maintained Chromosomes**

| **Gene Name** | **NMC** | **MIC**  **supercont** | **Start** | **Stop** | **Gene Product Name** | **nr blastp top hit** | **InterProScan Hits** | **RNAseq support** | **Notes** |
| --- | --- | --- | --- | --- | --- | --- | --- | --- | --- |
| TTHMIC_00001 | 1R-29/30 | 2.30  GenBank: JH659619.1 | 678348 | 678530 | NUMOD4 motif-containing homing endonuclease | ref\|YP_008318244.1\| putative HNH endonuclease [Lactococcus phage phi7]gb\|AGI11221.1\| putative HNH endonuclease [Lactococcus phage phi7] | IPR010902- PF07436 NUMOD4, a putative DNA-binding motif found in homing endonucleases and related proteins | N | Homology to *T. thermophila* Tlr element |
| TTHMIC_00002 | 4R-29/30 | 2.75  GenBank: JH659664.1 | 263293 | 270059 | piggyBac transposase | dbj\|BAI68043.1\| piggyBac-like protein Tpb1p [Tetrahymena thermophila] | IPR024196- Ku80, IPR016194- SPOC-like C-terminal Domain, IPR006164Ku70/Ku80 beta barrel, IPR14893- Ku, C-terminal, IPR029526- PiggyBac transposable element derived protein | Y | TPB3 |
| TTHMIC_00003 | 4R-29/30 | 2.75  GenBank: JH659664.1 | 271760 | 272607 | kinase domain protein | gb\|EWS73736.1\| kinase domain protein [Tetrahymena thermophila SB210] | No IPR hits | Y |  |
| TTHMIC_00004 | 5L-10/9 | 2.78  GenBank: JH659667.1 | 3904 | 4597 | hypothetical protein | no significant hits | No IPR hits | N |  |
| TTHMIC_00005 | 5L-10/9 | 2.78  GenBank: JH659667.1 | 4983 | 5617 | hypothetical protein | na | na | na |  |
| TTHMIC_00006 | 5L-11/10 | 2.78  GenBank: JH659667.1 | 21387 | 22115 | Tc1-like transposase | gb\|EXX52255.1\| hypothetical protein RirG_254680 [Rhizophagus irregularis DAOM 197198w] | IPR002492- Transposase, Tc1-like | N |  |
| TTHMIC_00007 | 5L-11/10 | 2.78  GenBank: JH659667.1 | 22921 | 23663 | hypothetical protein | gb\|EAR95642.2\| hypothetical protein TTHERM_00266520 [T. thermophila SB210] | No IPR hits | Y |  |
| TTHMIC_00008 | 1R-11/12 | 2.105  GenBank: JH659694.1 | 82080 | 92353 | DUF3638 family protein | no significant hits | IPR022099-DUF3638 | Y | Duplicated Region |
| TTHMIC_00009 | 1R-11/12 | 2.105  GenBank: JH659694.1 | 94082 | 94863 | hypothetical protein | ref\|XP_002407133.1\|GTP-binding protein CRFG/NOG1, putative [Ixodes scapularis] | No IPR hits | Y |  |
| TTHMIC_00010 | 1R-11/12 | 2.105  GenBank: JH659694.1 | 95272 | 95806 | ubiquitin-conjugating enzyme | ref\|XP_001013377.1\| Ubiquitin-conjugating enzyme family protein [Tetrahymena thermophila]gb\|EAR93132.1\| ubiquitin-conjugating enzyme [Tetrahymena thermophila SB210] | IPR000608, IPR016135- ubiquitin conjucating enzymes RWD-like and E2 | Y |  |
| TTHMIC_00011 | 1R-11/12 | 2.105  GenBank: JH659694.1 | 96769 | 98338 | hypothetical protein | no significant hits | PS00972- peptidase c19 ubiquitin carboxyl-terminal hydrolase, conserved site. | Y |  |
| TTHMIC_00012 | 1R-11/12 | 2.105  GenBank: JH659694.1 | 101536 | 111473 | DUF3638 family protein | gb\|EXK76993.1\| hypothetical protein FOQG_18281, partial [Fusarium oxysporum f. sp. raphani 54005] | IPR022099-DUF3638 | Y | Duplicated Region |
| TTHMIC_00013 | 1R-11/12 | 2.105  GenBank: JH659694.1 | 112536 | 113077 | hypothetical protein | na | na | na |  |
| TTHMIC_00014 | 1R-11/12 | 2.105  GenBank: JH659694.1 | 114207 | 116066 | hypothetical protein | no significant hits | IPR018200- peptidase C19, ubiquitin carboxyl-terminal hydrolase conserved site | Y |  |
| TTHMIC_00015 | 1R-11/12 | 2.105  GenBank: JH659694.1 | 116481 | 117210 | ubiquitin-conjugating enzyme | ref\|XP_001013377.1\| Ubiquitin-conjugating enzyme family protein [Tetrahymena thermophila]gb\|EAR93132.1\| ubiquitin-conjugating enzyme [T. thermophila SB210] | IPR000608, IPR016135- ubiquitin conjucating enzymes RWD-like and E2 | Y |  |
| TTHMIC_00016 | 1R-11/12 | 2.105  GenBank: JH659694.1 | 118218 | 119779 | hypothetical protein | no significant hits | No IPR hits | Y |  |
| TTHMIC_00017 | 1R-11/12 | 2.105  GenBank: JH659694.1 | 121365 | 121584 | transmembrane protein, putative | no significant hits | No IPR hits | Y | Internal duplication |
| TTHMIC_00018 | 1R-11/12 | 2.105  GenBank: JH659694.1 | 125237 | 126221 | hypothetical protein | no significant hits | No IPR hits | Y |  |
| TTHMIC_00019 | 1R-11/12 | 2.105  GenBank: JH659694.1 | 127278 | 127761 | hypothetical protein | no significant hits | No IPR hits | Y |  |
| TTHMIC_00020 | 1R-11/12 | 2.105  GenBank: JH659694.1 | 131369 | 141256 | DUF3638 family protein | no significant hits | IPR022099-DUF3638 | Y | Duplicated Region |
| TTHMIC_00021 | 1R-11/12 | 2.105  GenBank: JH659694.1 | 142022 | 143069 | transmembrane protein, putative | no significant hits | No IPR hits | Y |  |
| TTHMIC_00022 | 1R-11/12 | 2.105  GenBank: JH659694.1 | 144727 | 146584 | hypothetical protein | ref\|XP_001013379.1\|hypothetical protein TTHERM_00450830 [Tetrahymena thermophila] | IPR018200- peptidase C19, ubiquitin carboxyl-terminal hydrolase conserved site | Y |  |
| TTHMIC_00023 | 1R-11/12 | 2.105  GenBank: JH659694.1 | 146986 | 147713 | ubiquitin-conjugating enzyme | ref\|XP_001013383.1\| Ubiquitin-conjugating enzyme family protein [Tetrahymena thermophila]gb\|EAR93138.1\| ubiquitin-conjugating enzyme [T. thermophila SB210] | IPR000608, IPR016135- ubiquitin conjucating enzymes RWD-like and E2 | Y |  |
| TTHMIC_00024 | 1R-11/12 | 2.105  GenBank: JH659694.1 | 148723 | 150289 | ubiquitin carboxyl-terminal hydrolase | no significant hits | IPR001394 and IPR018200, Peptidase C19, ubiquitin carboxyl terminal hydrolase | Y |  |
| TTHMIC_00025 | 1R-11/12 | 2.105  GenBank: JH659694.1 | 155566 | 156216 | hypothetical protein | no significant hits | No IPR hits | Y |  |
| TTHMIC_00026 | 1R-11/12 | 2.105  GenBank: JH659694.1 | 157321 | 159034 | hypothetical protein | gb\|EWS74123.1\|hypothetical protein TTHERM_001469343 [T. thermophila SB210] | IPR018200- peptidase C19, ubiquitin carboxyl-terminal hydrolase conserved site | Y |  |
| TTHMIC_00027 | 1R-11/12 | 2.105  GenBank: JH659694.1 | 160099 | 163235 | ubiquitin activating enzyme | ref\|XP_003579050.1\| PREDICTED: ubiquitin-activating enzyme E1 2-like [Brachypodium distachyon] | IPR016040 NAD(P)-binding domain, IPR009036 Molybdenum cofactor biosynthesis, MoeB, IPR UBA/THIF-type NAD/FAD binding fold, IPR000127 (PF00899) Ubiquitin-activating enzyme repeat,, Panther- ubiquitin activating enzyme E1 | Y |  |
| TTHMIC_00028 | 1R-1/2 | 2.221  GenBank: JH659810.1 | 92418 | 101771 | hypothetical protein | gb\|EWS74416.1\| translation initiation factor eIF-5A family protein [Tetrahymena thermophila SB210] | IPR003590- Leucine rich repeat, ribonuclease inhibitor subtype | Y | Internal Duplications |
| TTHMIC_00029 | 1R-2/3 | 2.221  GenBank: JH659810.1 | 103928 | 109692 | hypothetical protein | gb\|EAS01534.2\| translation initiation factor eIF-5A family protein [Tetrahymena thermophila SB210] | IPR003590- Leucine rich repeat, ribonuclease inhibitor subtype | Y |  |
| TTHMIC_00030 | 1R-3/4 | 2.221  GenBank: JH659810.1 | 125172 | 131076 | hypothetical protein | gb\|EAR86164.3\| translation initiation factor eIF-5A family protein [Tetrahymena thermophila SB210] | IPR003590- Leucine rich repeat, ribonuclease inhibitor subtype | Y |  |
| TTHMIC_00031 | 1R-5/6 | 2.221  GenBank: JH659810.1 | 141788 | 151191 | hypothetical protein | ref\|XP_976759.2\| hypothetical protein TTHERM_00874790 [Tetrahymena thermophila] | IPR003590- Leucine rich repeat, ribonuclease inhibitor subtype | Y |  |
| TTHMIC_00032 | 4R-6-7 | 2.273  GenBank: JH659862.1 | 99861 | 101738 | hypothetical protein | ref\|XP_001010903.1\| hypothetical protein TTHERM_00124080 [Tetrahymena thermophila] | No IPR hits | Y |  |
| TTHMIC_00033 | 4R-6-7 | 2.273  GenBank: JH659862.1 | 102138 | 103541 | hypothetical protein | ref\|XP_001026830.1\| hypothetical protein TTHERM_01070320 [Tetrahymena thermophila] | No IPR hits | Y |  |
| TTHMIC_00034 | 4R-5/6 | 2.273  GenBank: JH659862.1 | 114180 | 115475 | ORF2 reverse transcriptase | gb\|AAQ82023.1\| ORF2 [Tetrahymena thermophila] | IPR000477- reverse transcriptase | N | *T. thermophila* REP element ORF2 |
| TTHMIC_00035 | 4R-5/6 | 2.273  GenBank: JH659862.1 | 118870 | 119403 | hypothetical protein | gb\|AAQ82025.1\| ORF1 [Tetrahymena thermophila] | No IPR hits | N | Possible *T. thermophila* REP element ORF1 homology |
| TTHMIC_00036 | 4R-5/6 | 2.273  GenBank: JH659862.1 | 119570 | 119965 | hypothetical protein | gb\|EWS72299.1\| hypothetical protein TTHERM_001085463 [T. thermophila SB210] | No IPR hits CATH superfamily 1.10.240.10- Tyrosyl-transfer RNA Synthetase | N | Possible *T. thermophila* REP element ORF1 homology |
| TTHMIC_00037 | 4R-5/6 | 2.273  GenBank: JH659862.1 | 121559 | 121867 | hypothetical protein | ref\|XP_001026829.1\| hypothetical protein TTHERM_01070310 [T. thermophila]gb\|EAS06584.1\| hypothetical protein TTHERM_01070310 [Tetrahymena thermophila SB210] | No IPR hits | Y |  |
| TTHMIC_00038 | 4R-5/6 | 2.273  GenBank: JH659862.1 | 123406 | 124442 | hypothetical protein | gb\|EAR90658.2\| hypothetical protein TTHERM_00124080, partial [T. thermophila SB210] | No IPR hits | N |  |
| TTHMIC_00039 | 3L-28/27 | 2.294 | 97083 | 98464 | helicase, putative | gb\|AAL73453.1\|AF451860_1 putative helicase [Tetrahymena thermophila] | No IPR hits | N |  |
| TTHMIC_00040 | 3L-28/27 | 2.294 | 98805 | 100236 | helicase, putative | gb\|AAL73453.1\|AF451860_1 putative helicase [Tetrahymena thermophila] | No IPR hits | N |  |
| TTHMIC_00041 | 3L-28/27 | 2.294 | 100291 | 100569 | NUMOD4 motif-containing homing endonuclease | ref\|YP_008318244.1\| putative HNH endonuclease [Lactococcus phage phi7]gb\|AGI11221.1\| putative HNH endonuclease [Lactococcus phage phi7] | IPR010902- PF07436 NUMOD4, a putative DNA-binding motif found in homing endonucleases and related proteins | N | Homology to T. thermophila Tlr element |
| TTHMIC_00042 | 4L-3/2 | 2.310 | 106365 | 107561 | DDE family transposase | emb\|CCQ48560.1\| 42 kDa transposase [Tetrahymena thermophila] | PF13358- DDE_3 | N |  |
| TTHMIC_00043 | 4R-3/4 | 2.337 | 80496 | 80909 | hypothetical protein | no significant hits | No IPR hits | Y |  |
| TTHMIC_00044 | 4R-3/4 | 2.337 | 85571 | 86382 | BED zinc finger protein | ref\|XP_008548025.1\|PREDICTED: zinc finger BED domain-containing protein 1-like [Microplitis demolitor] | IPR003656- Zn finger, BED type | N |  |
| TTHMIC_00045 | 4R-3/4 | 2.337 | 91572 | 92180 | DDE family transposase | gb\|KCZ78035.1\| hypothetical protein H311_00945 [Anncaliia algerae PRA109] | IPR024445- Transposase, ISXO2-like | N |  |
| TTHMIC_00046 | 4R-3/4 | 2.337 | 92630 | 93990 | DDE family transposase | ref\|XP_001021762.1\| hypothetical protein TTHERM_00898170 [Tetrahymena thermophila]gb\|EAS01517.1\| hypothetical protein TTHERM_00898170 [T. thermophila SB210] | IPR024445- Transposase, ISXO2-like | Y |  |
| TTHMIC_00047 | 5L-10/9 | 2.647 | 9463 | 10063 | hypothetical protein | ref\|XP_001470785.1\| hypothetical protein TTHERM_00266529 [Tetrahymena thermophila] | No IPR hits | N |  |
