# Supplementary material for: Structure of the germline genome of Tetrahymena thermophila and relationship to the massively rearranged somatic genome
Source: eLife. 2016 Nov 28;5:e19090. doi: 10.7554/eLife.19090 (PMC5182062; doi:10.7554/eLife.19090)
Supplement: Supplementary file 4. — DOI: http://dx.doi.org/10.7554/eLife.19090.028 [file elife-19090-supp4.docx]

**Appendix Supplementary File 1. Cbs segment sequences and alignments**

**Section A 1.**

**Sequence Cbs_1L-5_to_1 (supercontig 2.264, 54,384 – 55,049, 726 bp)**

ATATTTTTGAATCTTTATGTGAAATGTTTTTAGTTTTTTATAATTTTTTGTTTTTATATTGAAATAAACCAACCTCATTAGTAATTTATTTAAGACTGAATTATTATAATTTTAAACT

ATATTTTTAAATCTTTATTTGAAATTTTTTTAGTTTTTTATAATTTTATGTTTTTACATTAAAATAAACCAACAACCTCATTAGTAATTTATTTAAGACTGAATTATTATAATTTTAAACT

ATATTTTTTAATCTTTATGTGAAAATTTTTAAGTTTTTTATAATTTTATGTTTTTACATTAAAATAAACCAACCCCTTTAGTAATTTATTTAAGAATGAATATTCATTTTTTTAAATT

ATATTTTTTAATCTTTATTTGAAATTTTTTTAGTTTTTTATAATTTTATGTTTTTACATTAAAATAAACCAACCTCTTTAGTAATTTATTTAAGACAGACTGAATATTTATTTTTTTAAATT

ATATTTTTGAATCTTTATTTATAATTTTTTTAGTATTTTATAATTTTATGTTTTTACATTAAAATAAACCAACCCCTTTAGTAATTTATATAAGACTGAATATTTATAATTTTTAATT

TTGTTTTATTTGATTGATAATTTTTTGTTTTTAAATTGAAATAAACCAACCCCTTTGATAACTTATTTAAGACTGAATATCTATAATTTTTAATTTTATTTTTTTTGATGATTTTTTATTATTATTTTA

A continuous segment is shown, but repeat units are in separate “paragraphs”. The repeats containing Cbs 1L-5 and 1L-1 are at the top and bottom, respectively. Cbs’s are highlighted aqua, variant nucleotides highlighted yellow give a functional Cbs, while those highlighted fuchsia are predicted to be nonfunctional.

**Clustal Omega multiple sequence alignment of the six tandem repeat units**

Cbs_1L-AAC ATATTTTTAAATCTTTATTTGAAATTTTTTTAGTTTTTTATAATTTTATGTTTTTACATT

Cbs_1L-2 ATATTTTTGAATCTTTATTTATAATTTTTTTAGTATTTTATAATTTTATGTTTTTACATT

Cbs_1L-5 ATATTTTTGAATCTTTATGTGAAATGTTTTTAGTTTTTTATAATTTTTTGTTTTTATATT

Cbs_1L-3 ATATTTTTTAATCTTTATTTGAAATTTTTTTAGTTTTTTATAATTTTATGTTTTTACATT

Cbs_1L-4 ATATTTTTTAATCTTTATGTGAAAATTTTTAAGTTTTTTATAATTTTATGTTTTTACATT

Cbs_1L-1 ------------------------TGTTTTATTTGATTGATAATTTTTTGTTTTTAAATT

:::::::: ::::::::: :: :: **** ::* :** ******** ******** ***

Cbs_1L-AAC AAAATAAACCAACAACCTCATTAGTAATTTATTTAAGACTGAATTATTATAATT----TT

Cbs_1L-2 AAAATAAACCAAC---CCCTTTAGTAATTTATATAAGACTGAATATTTATAATT----TT

Cbs_1L-5 GAAATAAACCAAC---CTCATTAGTAATTTATTTAAGACTGAATTATTATAATT----TT

Cbs_1L-3 AAAATAAACCAAC---CTCTTTAGTAATTTATTTAAGACAGACTGAATATTTATTTTTTT

Cbs_1L-4 AAAATAAACCAAC---CCCTTTAGTAATTTATTTAAGAATGAATATTCAT----TTTTTT

Cbs_1L-1 GAAATAAACCAAC---CCCTTTGATAACTTATTTAAGACTGAATATCTAT----AATTTT

************ * * **::***:****:*****::**:* :** **

Cbs_1L-AAC AAACT-

Cbs_1L-2 TAATTT

Cbs_1L-5 AAACT-

Cbs_1L-3 AAATT-

Cbs_1L-4 AAATT-

Cbs_1L-1 TAATTT

** *

Asterisks: all nucleotides aligned at that position are identical; colons: all but one of the six nucleotides are identical.

**Section A 2.**

**MIC DNA segment including Cbs 1R-35&36 (Supercontig 2.14: 805,840 – 507,322, 1483 bp)**

ATTTTTTCCTTAATTAAAAAATCTTTCTTAATAAATTCAATTATATTAGCTGTCAGCATTAATTGCCAAAATTAAATATTTGCTAATAAATGATCATAGATACTAAATAATATGTATTATCTTTTGTAATATCAATAGGTAAACACAATCCTTAAAGGAAAATAATTCTTTTTATGTATCTTTATAGCGAATGCTAAACAAATAGAATCACTAGAATTTCAAAATTTATAAGTCTACATTTTTAATTTTGATAAAAAATTTGTACTTTTTAATTTTGCTTTTCGAAAATTCTAAACTTTTAAACATGCATAGATTCTTAAGTTATTTATTTTCAAGATCACTAAAAAATATAAGAATGAAAACCTAATTAAATTTTATCATAATTTTAGCCTTTTTTTTTAGTTTTAGATACTTTATTTTTAGTTCTTAAAATAAATATTATTTTTAATAAACAATTTTTTAAAATAAAATATCAAAAAATTTTCTTAATTTGATAAAAAAAACCAACCCCTTTATTGCTTATTGAATTATTATATTACTCTTACTTATTTTAATTGTTATCAAAAAATATCAAATTTAATAAGTTTTTATTAAAATTATTTATTTGTTTTTTAATAAAATAAAATAATGTGTTGAATTTATTTTTCTTTTAAAATATCAATAAAATATTAAATAAAAATGGAAAAAAATTTATAAGTCTTTTTTTTATTTTTATAAAAAAATGTGTACTTTTTAATTTTGCTTTTAGAAAATTCTAAACTTTTATAATGCATAGATTCTTAAGTTATTTATTTATTTATAAGATCAATAAAATATAAGAATGAATACCTAATCAAATTTTATCATAATTTTAGCCTTTTTTTTAGTTTTAGATACTTTAATTTTAGATCTTAAAATAAACATTATTTTAAAATCTATTAAACAATTTTTAAAAATAAAATATCAAATTTTCTTAATTCGAAAAAAAAAAAACCAACCCCTTTATTACTTATTGAATAAAAACTATTATTATATTACTTTTACTTATATTAATTGTTATCAAAAAATACCAAATTTAATAAGTTTTTATTAATATTATTTATTTATTTTTTAATCAAATAAAATAATATTTTTAATGTAATTTTTTTATAAAACATCTATAAAATATTAAATAAAAATGGGGAAAAAATTTTATAATTCTAAATTTTACTAGAAATAATTAGAATTATTAAAAAGATCTTAGATTAATTAAATTCTAACTAAGTTTTTTAAGATTTAAAAAGTATTTCTTTTATACACTTAAAAGAAAATTAAAACAATTATTGCGTTTTTTGAATAATTTTTTATTTGATCAAACTTGAAAGTAAGTAAATATACATAGATATAAAATTAAATGATTCTTATAAATATATATAATATATATAAAAGTAGTTTTAATTTAATATTTGAGTGCTGGTTAGTTGTTTGTTTTTATAATTATATTATTGTATTTGTTTTACATTTA

**MIC DNA segment including Cbs 1R-37&38 (Supercontig 2.14: 964,938- 967,074, 2137 bp)**

GGTTATTAATCTATCATAATATATATTATTTTGATTTTGTTAAATTCGCAAGGAATAATTATTTATTATTACATTAATTATTTTTTAATTTCTAGCAAGATAATTTATGTTTTATATAACTAAAATGATTTTATATCATCTAATACACAAAATAATTCAAGTTTGTTATAATTTATAATTACCATCTCTTTGTATTCAATAATATTTTTTAATGGCTTATTTAAAAATTGTTAAAATTTTATTTTTTGAAACTTATATTTTTATATTCGAAACAACTCTATTAATAAATAAAATATATTTTAATTATGTAAAAATTAATTATTATTTTTCTTTTATTTTATACTTCATAAAAAATTGCTTTTCGCCTTTATTTTAACATTAATTTTTAATATAAAAATATATAAATTAATTTATTCATTTACAGTTATCAAAATTCTGAAAAAGAAAAAAACAAATTTTTATATTTCAAAAAAATTAAATCAAACTAAAATTTATTAAAAAAACCAACCTCTTTTATGAAAATAAATTAAAGAGAACAAAATATTACTTAGTTTTAATTAATTACTTAAAATAATTAAAATTTAATATATGAAAATAGATAATCACATAAACATTTTTTCTCTACCCGCTGTTCAGTACTTCTAACTACTTTATATAAAATAATTTATTAACTTTAGTTATAATTTATATTTAAAAATAAATCTTTTATTATAATAATTGTTAAAATAAAAACATAATAAAGTGATTTAAATAACATAGTAAAAATAAAAACTTTATCTTTGATTGAGTTTTTATTTTTTATTAATTCTTTTAATTAATGTAATATAATAAGATATAATAAAATGATTTGAATGCTATTGTAATAAAGTGTATATTGTTTTTATTTAATAATTTTATTTAATTGATGTAAGATAATAGATAAATAAACACTTATTTTATCTAAATAGTTTTTTATTATTAGTTAAATAATATACAAATTGTAGATTTATTATTTATTTGAATTAAAAGCTTTTATTTTATTGAACCCTAAAAAAATGATGAACGAACAGTAATGAAAAAAAATAAATAACAACTTAGTTTTTAAATTAATTTTTTATCTTTTAAACAAAATTTGATAATAAGAGAATAAATTTAATATTTGTAATAATTTTGAAAATAAAATAAAATCTAAGATGATAATATTTAATTATTTTTAAATAAAAGAATAAATCAAAATAAGATTAAAATATAAATTAACTTTTTTATATATAAATAAAAGTTTAATTTTTTAATTTTAATTTGAAAGTATGAAAAAAAATAAGAATAATTCTAAATTAGATTAAATAAATTATTTAAATAATTTAAAAAAAACTTTAATGTAAAGATTAAAATAAAAAGAAAATGTTTTAAATAAGTAATTACCAATTCTTTATTAAAATTTATTTTTTAAAGGTATAGTAAAATTGAATAGTATGAAATAATTAATTAAAATGTTAATTGTATGTTGATTAGATATTTTATCCTTCAAATAAATAAGAATTGTAAATTACCTTTTTGAAGAAAAAGATTTTAAAAGAATTTTAAACAAATTATAATAAAAAAATCATAAAAAGAAAATAAAAACTAAAAATGTTTGTTTAAAAAAAACCAACCCCTTTAGTGAAAATAAATTAATAGAAAAAATATTATTTTAATTTGATTAATTGATGAAAAGTTTTAATAAGTTTAATTTTTAATGAATTTCTTTTCTTCTTAAACTTTTATTTAAAGTTTTAGCAGCAGTGGTATAAAAATATGTTTGATAAGCTAGTAATAGCTGCTTTTAAATATTAACGGAAATATTATTCTGTAATGGATAAATATTAATTTAAGCAAAATTTACTTATTAAAAATATTTGATTGATTATTATTAATTAATTATTTAGATCAATAATTTAAATATGTAATATTGATTATTTTTTAATAATTAATTTTATCAAGTGTAAATTGAGAATCAGGTAATGAATAACATTGAAATCGATGAAAATGAAGAAACTAAGGAACAAATTGAAATTTAGTAAAATCCTAATAGTATGATACACTATGCTTAGCAATTAGGAAATTTAGATGAAACAAATTAAATATCTTAGATTTAGTAAAACAATTAAAATGAAATTTC

Within-clade Blast alignments are significant but between-clade alignments are not (see below). Nevertheless, the following features tend to link the two clades into a possible superclade, 1R-35/1R-37.

a) Three of the four Cbs’s involved have the rare 1A,11C double substitution. Only two additional incidences of this variant Cbs are found among the 225 Cbs’s (Table 2, main text).

b) The four Cbs’s are consecutive, as reflected by their numbering, and are similarly oriented within supercontig MIC2.14. The two pairs of Cbs’s are 460 Kb apart and define the ends of maintained MAC chromosome scaffold 8254563 (Supplementary Table 7).

**Within clade alignments:**

**Alignment of Cbs 1R-35 & 36**

Query= Cbs 1R-35&36 1493 bp

>lcl|50294 Cbs 1R-35&36 1493 bp Length=1483

Score = 580 bits (642), Expect = 5e-169

Identities = 440/512 (86%), Gaps = 33/512 (6%)

Strand=Plus/Plus

Query 686 AAAATTTATAAGTCTT--TTTTTTATTTTTATAAAAAAATGTGTACTTTTTAATTTTGCT 743

||||||||||||||| ||||| ||||| |||||||| | |||||||||||||||||||

Sbjct 221 AAAATTTATAAGTCTACATTTTTAATTTTGATAAAAAATT-TGTACTTTTTAATTTTGCT 279

Query 744 TTTAGAAAATTCTAAACTTTTATA-ATGCATAGATTCTTAAGTTATTTATTTATTTATAA 802

||| |||||||||||||||||| | ||||||||||||||||||||||||||| ||

Sbjct 280 TTTCGAAAATTCTAAACTTTTAAACATGCATAGATTCTTAAGTTATTTATTTTC----AA 335

Query 803 GATCAATAAAA--TATAAGAATGAATACCTAATCAAATTTTATCATAATTTTAGCCTTTT 860

||||| ||||| |||||||||||| ||||||| ||||||||||||||||||||||||||

Sbjct 336 GATCACTAAAAAATATAAGAATGAAAACCTAATTAAATTTTATCATAATTTTAGCCTTTT 395

Query 861 TTTT-AGTTTTAGATACTTTAATTTTAGATCTTAAAATAAACATTATTTTAAAATCTATT 919

|||| |||||||||||||||| |||||| |||||||||||| |||||||| || |

Sbjct 396 TTTTTAGTTTTAGATACTTTATTTTTAGTTCTTAAAATAAATATTATTTTTAA------T 449

Query 920 AAACAATTTTTAAAAATAAAATATCAAA---TTTTCTTAATTCGAAAAAAAAAAAACCAA 976

||||||||||| |||||||||||||||| ||||||||||| || ||||||||||||

Sbjct 450 AAACAATTTTTTAAAATAAAATATCAAAAAATTTTCTTAATTTGAT--AAAAAAAACCAA 507

Query 977 CCCCTTTATTACTTATTGAATAAAAACTATTATTATATTACTTTTACTTATATTAATTGT 1036

|||||||||| |||||||||| |||||||||||| |||||||| ||||||||

Sbjct 508 CCCCTTTATTGCTTATTGAAT---------TATTATATTACTCTTACTTATTTTAATTGT 558

Query 1037 TATCAAAAAATACCAAATTTAATAAGTTTTTATTAATATTATTTATTTATTTTTTAATCA 1096

|||||||||||| ||||||||||||||||||||||| ||||||||||| ||||||||| |

Sbjct 559 TATCAAAAAATATCAAATTTAATAAGTTTTTATTAAAATTATTTATTTGTTTTTTAATAA 618

Query 1097 AATAAAATAATATTTTTAATGTAATTTTTTTATAAAACATCTATAAAATATTAAATAAAA 1156

||||||||||| | || ||| || |||| || ||||| ||| ||||||||||||||||||

Sbjct 619 AATAAAATAATGTGTTGAATTTATTTTTCTTTTAAAATATCAATAAAATATTAAATAAAA 678

Query 1157 ATGGGGAAAAAATTTTATAATTCTAAATTTTA 1188

|| |||||||| ||||||| ||| |||||

Sbjct 679 AT--GGAAAAAAATTTATAAGTCTTTTTTTTA 708

**Alignment of Cbs 1R-37 & 38**

Query= Cbs 1R-37&38 2137 bp

>lcl|99975 Cbs 1R-37&38 2137 bp

Score = 77.0 bits (84), Expect = 3e-17

Identities = 111/150 (74%), Gaps = 14/150 (9%)

Strand=Plus/Plus

Query 1562 AAACAAATTATAATAAAAAAATCATAAAAAGAAAATAAAAACTAAAAATGTTTGTTTAAA 1621

||||||||| | ||| ||| ||||| |||| ||| |||||| ||| || ||

Sbjct 449 AAACAAATTTTTATATT----TCAAAAAAATTAAATCAAA-CTAAAA---TTTATT--AA 498

Query 1622 AAAAACCAACCCCTTTAGTGAAAATAAATT-AATAGAA-AAAATATTATTTTAATTTGAT 1679

||||||||||| |||| |||||||||||| || |||| ||||||||| || ||| ||

Sbjct 499 AAAAACCAACCTCTTTTATGAAAATAAATTAAAGAGAACAAAATATTACTTAGTTTTAAT 558

Query 1680 TAATTGATGAAAAGTTTTAATAAGTTTAAT 1709

||||| | |||| ||| || ||||||

Sbjct 559 TAATTACTTAAAATAATTA--AAATTTAAT 586

**Beween clade alignments:**

Alignments between the two clades (Cbs 1R-35-37 and 36-38) were not statistically significant: Expected value >1E-07; this is likely due to low sequence complexity. Consistent with this, the (rare) nucleotides C and G failed to align with one another, unlike the case of authentic alignments.

**Cbs 1R-35 and 1R-38 alignment**

Query= Cbs 1R-35&36 1493 bp

>lcl|50295 Cbs 1R-37&38 2137 bp

Length=2137

Score = 42.8 bits (46), Expect = 3e-07

Identities = 58/80 (73%), Gaps = 11/80 (14%)

Strand=Plus/Plus

Query 443 TTTTAATAAACAATTTTTTAAAATAAAATATCAA---AAAAT-TTTCTTAATTTGATAAA 498

|| ||||||| || | | |||| |||||| || ||||| ||| || ||||

Sbjct 1569 TTATAATAAAAAAATCATAAAAAGAAAATAAAAACTAAAAATGTTTGTT-------TAAA 1621

Query 499 AAAAACCAACCCCTTTATTG 518

||||||||||||||||| ||

Sbjct 1622 AAAAACCAACCCCTTTAGTG 1641

**Cbs 1R-36 and 1R-38 alignment**

Score = 39.2 bits (42), Expect = 4e-06

Identities = 42/54 (78%), Gaps = 5/54 (9%)

Strand=Plus/Plus

Query 965 AAAAAAAACCAACCCCTTTATT---ACTTATTGAAT--AAAAACTATTATTATA 1013

|||||||||||||||||||| | | | | | ||| ||||| ||||||| ||

Sbjct 1619 AAAAAAAACCAACCCCTTTAGTGAAAATAAATTAATAGAAAAAATATTATTTTA 1672

**Section A 3.**

**Alignment of Cbs 4R-25 and 5’ rDNA end**

Score = 172 bits (190), Expect = 2e-45

Identities = 190/245 (78%), Gaps = 15/245 (6%)

Strand=Plus/Plus

Query 460 ATTATTAATACA--AAATAAATAAAAATAAAAATTATT--ATGAAATAATGAGGTTGGTT 515

||||| |||| | || ||| |||||||||| |||| | ||| || ||||||||||

Sbjct 154 ATTATCAATAAATGAATTAATTAAAAATAAATTATATTTAAATAAACAAAGAGGTTGGTT 213

Query 516 TAATTTTTTAAAA-AAGTTAAAACCCAAAAATCTTATTTTTTATTCATATTTTATTAAAG 574

|||||||| |||| |||||||||||| || | || ||| | || | ||| | ||||

Sbjct 214 TAATTTTTAAAAATAAGTTAAAACCCCAATTTAATAATTTCT-TTGACATTGAGTAAAAG 272

Query 575 TTATTTTTTTATTAGTAATACTTTTATATTTCAGATTGTTTATGAATGATTATGATTAAG 634

|||||| || | | |||||||||| || ||| || || |||||||||||||| ||

Sbjct 273 TTATTTATTGAAT-GTAATACTTTGATTTTT-AG-TTATTTATGAATGATTA------AG 323

Query 635 ATGCTAAACTGTTTAAATTCTATTATATTTTGAAAAGTTTATATAAAAATACTCATAAAT 694

||| |||| |||||||||||||| |||||||||| |||||||||| |||| ||||||

Sbjct 324 ATGTTAAAATGTTTAAATTCTATAATATTTTGAATAGTTTATATATGAATAAACATAAAA 383

Query 695 TATTA 699

|||||

Sbjct 384 TATTA 388

**Alignment of Cbs 4R-24 and 3’ rDNA end**

BLASTN Expected Value threshold = 1E-05; Word size =7; Low complexity filter = OFF

Query= Supercontig 2.7 of *Tetrahymena thermophila* SB210 (MIC) 2230205-2231878

Length=1674 (includes Cbs 4R-24 and 25)

>lcl|35605 MIC rDNA region sequence assembly rev compl 3’-5’ Length=10918

Score = 228 bits (252), Expect = 3e-62

Identities = 490/714 (69%), Gaps = 85/714 (12%)

Strand=Plus/Plus

Query 727 ATTAAATTTATTTATTTTTACTGAAATTAAAGTTTT-AAATTGATAAAATCTTT------ 779

|||||||| |||| |||||| |||||||| | |||| | |||||| | |

Sbjct 10188 ATTAAATTCATTTTAATTTACTAGCATTAAAGTAGTCAAATAGCTAAAATATGTAATATT 10247

Query 780 ---TCATTTTTAATATC-TATTTTTCTTTTAAATTTTTTAATATTACTAATTTTATTTTA 835

|||||||| | | | |||||| ||| |||||| | ||| || |||||||| | |

Sbjct 10248 TTCTCATTTTTTAGAACCTATTTTGCTTGAAAATTTGTAAAT-TTGTAAATTTTATCTAA 10306

Query 836 TTTAAAA--TTATTCAAAATATAAAAGAACACATTATTACCTGTCATACAATATGTTAGT 893

||||||| || | ||| ||| |||| || | ||| || | |||| || || ||||

Sbjct 10307 TTTAAAACATTTTACAACATAAAAAACAA-ATATTTTTGTTTTAAATACTATTTGCTAGT 10365

Query 894 TGAATTAAATCAAAATCATATTGAGTAGCTCC-TTTTCAT-AGTGGAATAAGGAAAATAT 951

| | ||| || ||||| || | | | ||| | |||||| |||| ||| || | |||||

Sbjct 10366 TTA-TTAGATTAAAATAATTTCTATTTGCTACATTTTCACCAGTGAAATTAGCAGAATAT 10424

Query 952 CAACATTTTATGAATTTTTACCCAAATTTTTTTTTCAGT---AAAGATTTCTCAATTCAA 1008

||||| | || | | ||| ||||||| ||| | || ||| ||||||| |

Sbjct 10425 AAACATCTAATTCTTATCTACTCAAATTTAAATTTTAAACCCAATTATTACTCAATTTTA 10484

Query 1009 AGTAAATTTCGAAGATAA--------------GTACTGAAGACCTTAAAACTAACTACAA 1054

||| | |||||||| ||| || |||||| | |||||||||| ||||

Sbjct 10485 AGTGATTTTCGAAGGTAAAATTCAACAACATAGTGCTGAAGGCTATAAAACTAACAACAA 10544

Query 1055 ATAGCAAAAAAA-TAACCTTCAGCACTATTTTATTGAA-TTTATCTTCGAAAATGACTTA 1112

| |||||||||| || ||||||||||||| || ||||| |||| |||||||||| |||

Sbjct 10545 AAAGCAAAAAAACTAGCCTTCAGCACTATGTTGTTGAATTTTACCTTCGAAAATCGCTTT 10604

Query 1113 AAATATTAATAAATAAAC----TTCTTTTATATAAAATTAATTT----TTGAAATAAACC 1164

|||||||||||| ||| |||||| ||||||| ||||||| ||||||||||||

Sbjct 10605 TAATATTAATAAACCAACCTCTTTCTTTAATATAAATTTAATTTTTGATTGAAATAAACC 10664

Query 1165 AACCTCATTTTGAAAATTAAATAGAA--ATTTTTTAAA------------CATCTA--TT 1208

|||||| || | || ||||| || ||| || || ||| || ||

Sbjct 10665 AACCTCTTTCTTTAATATAAATTTAATCATTGATTGAAATAAACCAACCTCATTTATTTT 10724

Query 1209 GAAA----AATAAAAT------TTAAAAATTTTAATTTGTTTGCT-GGATATTAAAGTTG 1257

|||| ||| |||| ||| || ||||||||||||| | || ||| ||

Sbjct 10725 GAAAATCTAATTAAATTAGAAATTACAATATTTAATTTGTTTGTTCTCATTTTA---TTA 10781

Query 1258 TAATGATAAATTTAAATGTTTATTCTT-TTTATTCT---AATTAAAATAATTTTTATTTA 1313

||| |||| ||||||| || ||||| |||||| | |||| ||| || || | |

Sbjct 10782 AAATTATAATTTTAAATATTCTTTCTTATTTATTATCAAAATTTAAAGCCATTATA-TAA 10840

Query 1314 TTTATAAATTTACTTTTTAAATTAATTTTTTATAAAAATTAAATAGAGAAATAT 1367

||| | |||||| |||||||||||| |||| | ||| |||| ||| ||||||

Sbjct 10841 TTTTTGAATTTA-TTTTTAAATTAA---TTTAAATAAAATAAA-AGAAAAATAT 10889

The assembly of the region that includes the rDNA-destined DNA is fragmented in the MIC genome assembly 2. A 10,918-bp assembly of the region was done by hand, by exploiting the overlap of sequence flanking Cbs 1L-16 (supercontig 2.78: 12,656-13,070) and the Cbs 1L-17 clade (supercontig 2.2: 590-1094) with the MAC rDNA palindrome sequence (GenBank sequence gi|10832|emb|X54512.1).

**MIC rDNA sequence assembly (5’end region: bp 9,435 – 10,918)**

TAACTCTTTATTTAATCAAAAAAAATTTATTTTGAATGTAAAATTTAACTAAAATGAATAAATGAAATTGATAAAATGTTTTTTACATATAAAAAATGATATACGCATGCTGTTAAAATTGTTTTATGTTTTTTTATATATGTTTTATTTAAATTTACTAGCATTTAAATGATTAAAAAATATAAAATAAATTTTACTTTTCTATCATTTTTAATTTATAAATTTTCTCTAAGTCTTTTTATCTCTTTTTGATTTTTTCTCTAAGTCTTTTTTTACACTTTTAAAATTTTTTCTAAGTCTTTTTTTCAACTCTCAAAAAAAGTGATTTTTTACTATTTTTGTTTTTTTTTTTGCCAAAAAAATATCTAATGAGCAATTTTCAAAATCAATATTTTTAATTCTAGATAAACTTTTTCTTTAAGTTATCTTTATAAAGTCTTTATTTTACCAAAAAAAAGTTTTTTGTGAATGAAAAATTTAACTAAAATGAATAAATGAAATTGATAAAATGTTTTTTACATAAAAAAAACTATTTACTCATATTCCTAAAACTATTTTACTTTTATTTATAAATGTTTTACTTAAATTTTCTAGCATTTAAATGTATAAAAAAAGGAAATACTTCATTTTTGTCCATTTTTAGATGCTTTTTCTTTAAAATTTAACTAATTTCTTAAATTTACCTCTTAAAAAATATTTTATCATTTTAAAATCATATATTTTATTAAAATAAGTGTTTTAAAGTGAAAATGAATTAAATTCATTTTAATTTACTAGCATTAAAGTAGTCAAATAGCTAAAATATGTAATATTTTCTCATTTTTTAGAACCTATTTTGCTTGAAAATTTGTAAATTTGTAAATTTTATCTAATTTAAAACATTTTACAACATAAAAAACAAATATTTTTGTTTTAAATACTATTTGCTAGTTTATTAGATTAAAATAATTTCTATTTGCTACATTTTCACCAGTGAAATTAGCAGAATATAAACATCTAATTCTTATCTACTCAAATTTAAATTTTAAACCCAATTATTACTCAATTTTAAGTGATTTTCGAAGGTAAAATTCAACAACATAGTGCTGAAGGCTATAAAACTAACAACAAAAAGCAAAAAAACTAGCCTTCAGCACTATGTTGTTGAATTTTACCTTCGAAAATCGCTTTTAATATTAATAAACCAACCTCTTTCTTTAATATAAATTTAATTTTTGATTGAAATAAACCAACCTCTTTCTTTAATATAAATTTAATCATTGATTGAAATAAACCAACCTCATTTATTTTGAAAATCTAATTAAATTAGAAATTACAATATTTAATTTGTTTGTTCTCATTTTATTAAAATTATAATTTTAAATATTCTTTCTTATTTATTATCAAAATTTAAAGCCATTATATAATTTTTGAATTTATTTTTAAATTAATTTAAATAAAATAAAAGAAAAATATTCTTTTACTTAAAGTGCTTATTAATATAT

Yellow: M-repeats; Green: M-repeat spacer; Light gray: Segment (including the Cbs, yellow and green segments) matching Cbs 4R-25 and adjacent sequence

**MIC rDNA sequence assembly (3’end region: bp 1 – 1,068)**

AAAATCTTTATAATTGAAGAATTTGAATAAAAATAGTTTGAACTGAAAGAAGGAATATCACAAAATTAGGATTTAATCAAGCATTCATATTCAAAAGAGTCATATTAGTAACAGAAAATTATCAATAATGTATTAAAAATATGCTACTTATGCATTATCAATAAATGAATTAATTAAAAATAAATTATATTTAAATAAACAAAGAGGTTGGTTTAATTTTTAAAAATAAGTTAAAACCCCAATTTAATAATTTCTTTGACATTGAGTAAAAGTTATTTATTGAATGTAATACTTTGATTTTTAGTTATTTATGAATGATTAAGATGTTAAAATGTTTAAATTCTATAATATTTTGAATAGTTTATATATGAATAAACATAAAATATTAATTAAATCTCAAAAATGACTAAGCTAGTCAAAGATTGAAGTTCTTATCAAAATTATTTTAATTAAAAATTTTATTTTTTGTAGTTTTCATAAATTTTTATAAGTTTTTCATTCAAAAATAAACTTTTAGAAGGGTTTTTTGTTATAAAAGATCAATAAATAAAATGTTTTTCAAGAAATTAATGATGATTAATAAATTTTATCTAAAAATGAAGCTTCCGCGTTAAATTTAAAATTTCTTATAAGCCTTAAATAATGCAAATCTCATAAATTATAGAGATCAACCATTTTTACATATTTATTGAATTAAATTTTAGTGAAATTTTTGCATCAATTTCCACTTAATTTTTGTGTGGGATTTTTATTTTTACGCTTGAAATTAAAGTGGGATAAAGTTAAAATCTAAGCGTAGTTTAGCGCATTTTTACACGCAAAAAAATTTTTATTCAAATCCACTTTAGCAAATTATTCATTGGACAAGCCGTGGTAAAAAAGAGATTTATCTAATAAAGTGGTTTTCTTGAAAAAAGTCAACTTTTTAAATCAAATCTTTAGGTCTTTTGGTTTGGTCCCAAAAAAAATATTGATAAGTGGGTGAAATTTTGAATGAAAAATTTGGTTAAAATACAAAGATTTTTTCATTCATTTTGCTTCTTAATAAAAGTCAACTTTTAGGAAAGA

Dark gray: Segment (including the Cbs) matching Cbs 4R-24 and adjacent sequence

**MIC region including Cbs 4R-24 and 25 (Supercontig 2.7: 2,230,205-2,231,878, 1674 bp)**

TTTACTAATATTTTAGCTTTTATAATTTTTTCAATGAATTTTTTTACAAATATTATTAGAATTCAAATTATTTTTTTAATTAATAAATATATAATATATTTATTTAAAACCAATTAGTTTATTAATTTTAAATTTCCTATAAAAAATTGTAAGTTTGAGGAAAAATTTGGAATTTAATTTGCACATATTTGCTTGGTAGAGTATTTTGATTACAGAAAATGATATAAAATTTATTTGTTTCTATGACTTGTAAAAAAATGAGTAATATTTTATATTAAAGAAATTATCATCCTTATACCTAATTCTCTTATGCTTTAAATAGCTTAAGAGACTTACTTATTTTAAAAAAAAGGCTAGTTTTTTTCGATGAAGACACACCCTAATTATCTTACAACATATGAAATGCAATAATTAAACTTATTAATTATAAATTTTTATAAAATTTCAATTATTTTCAATATTATTAATACAAAATAAATAAAAATAAAAATTATTATGAAATAATGAGGTTGGTTTAATTTTTTAAAAAAGTTAAAACCCAAAAATCTTATTTTTTATTCATATTTTATTAAAGTTATTTTTTTATTAGTAATACTTTTATATTTCAGATTGTTTATGAATGATTATGATTAAGATGCTAAACTGTTTAAATTCTATTATATTTTGAAAAGTTTATATAAAAATACTCATAAATTATTAcaagtcattttaatgaatgattgaagtATTAAATTTATTTATTTTTACTGAAATTAAAGTTTTAAATTGATAAAATCTTTTCATTTTTAATATCTATTTTTCTTTTAAATTTTTTAATATTACTAATTTTATTTTATTTAAAATTATTCAAAATATAAAAGAACACATTATTACCTGTCATACAATATGTTAGTTGAATTAAATCAAAATCATATTGAGTAGCTCCTTTTCATAGTGGAATAAGGAAAATATCAACATTTTATGAATTTTTACCCAAATTTTTTTTTCAGTAAAGATTTCTCAATTCAAAGTAAATTTCGAAGATAAGTACTGAAGACCTTAAAACTAACTACAAATAGCAAAAAAATAACCTTCAGCACTATTTTATTGAATTTATCTTCGAAAATGACTTAAAATATTAATAAATAAACTTCTTTTATATAAAATTAATTTTTGAAATAAACCAACCTCATTTTGAAAATTAAATAGAAATTTTTTAAACATCTATTGAAAAATAAAATTTAAAAATTTTAATTTGTTTGCTGGATATTAAAGTTGTAATGATAAATTTAAATGTTTATTCTTTTTATTCTAATTAAAATAATTTTTATTTATTTATAAATTTACTTTTTAAATTAATTTTTTATAAAAATTAAATAGAGAAATATCCATATTTATTAGACATATTGAAATAAGAATAATAATAATCTATATATCTTTATATTTTAATCTTTTTATAATTAATTTTATTTCTTGAAAACATTTAATTTTTATTATTACTTGCTACTGTTGCTTATGACAATATACAAAATTATAATAAGAATTTTATTTTAATATTATTTTATAATATGGAATTCAAAATTTTTTCATAAATCAATAATCTATTTAAAAATATGTTATTTCAATTATTATAAATTTATAAATAGCTAACTTAACTTTAATAGAGAAATTTAATTAGACTTTAAAATAAATTGA

Light and dark gray: sequences matching the 5’ and 3’ end of the MIC rDNA, respectively. Lower case letters: 27-bp alignment gap between matches to 5’ and 3’ rDNA ends. Yellow and green: matches to rDNA M-repeats and their nonpalindromic spacer, respectively.
